# DNA sequence models of genome-wide *Drosophila melanogaster* Polycomb binding sites improve generalization to independent Polycomb Response Elements

**DOI:** 10.1093/nar/gkz617

**Published:** 2019-07-24

**Authors:** Bjørn André Bredesen, Marc Rehmsmeier

**Affiliations:** 1 Computational Biology Unit, Department of Informatics, University of Bergen, P.O. Box 7803, N-5020 Bergen, Norway; 2 Integrated Research Institute (IRI) for the Life Sciences and Department of Biology, Humboldt-Universität zu Berlin, Unter den Linden 6, 10099 Berlin, Germany

## Abstract

Polycomb Response Elements (PREs) are *cis*-regulatory DNA elements that maintain gene transcription states through DNA replication and mitosis. PREs have little sequence similarity, but are enriched in a number of sequence motifs. Previous methods for modelling *Drosophila melanogaster* PRE sequences (PREdictor and EpiPredictor) have used a set of 7 motifs and a training set of 12 PREs and 16-23 non-PREs. Advances in experimental methods for mapping chromatin binding factors and modifications has led to the publication of several genome-wide sets of Polycomb targets. In addition to the seven motifs previously used, PREs are enriched in the GTGT motif, recently associated with the sequence-specific DNA binding protein Combgap. We investigated whether models trained on genome-wide Polycomb sites generalize to independent PREs when trained with control sequences generated by naive PRE models and including the GTGT motif. We also developed a new PRE predictor: SVM-MOCCA. Training PRE predictors with genome-wide experimental data improves generalization to independent data, and SVM-MOCCA predicts the majority of PREs in three independent experimental sets. We present 2908 candidate PREs enriched in sequence and chromatin signatures. 2412 of these are also enriched in H3K4me1, a mark of Trithorax activated chromatin, suggesting that PREs/TREs have a common sequence code.

## INTRODUCTION

The body plan of the fruit fly, *Drosophila melanogaster*, is genetically determined by transcription factors whose expression patterns are carefully coordinated and localized (1). Some transcription factors are produced early in development, where they gather at initiation elements in DNA that in turn establish the expression states of developmentally important genes (1). Later in development, these initiating factors deteriorate, and a memory of gene transcription states must be maintained (2,3).

Polycomb Response Elements (PREs) are cellular memory elements in DNA that maintain a memory of transcription states of their target genes over cell division (4,5). To accomplish this, PREs recruit Polycomb group (PcG) proteins, which maintain repression, and Trithorax group (TrxG) proteins, which antagonize PcG repression (6,7) (see Materials and Methods for a discussion of response elements nomenclature). PcG proteins were first identified as *Hox* gene regulators in *Drosophila melanogaster*, where PcG mutant flies exhibit ectopic *Hox* gene expression along the anterior-posterior axis (3,8). It has since been discovered that PcG proteins target a much wider range of genes (9–12) and that PcG proteins have mammalian homologs, with important roles in development and with implications in cancer (13,14).

The Polycomb system is best characterized in *Drosophila melanogaster*, where tens of PREs have been experimentally verified (1,15–17) and tens of PcG/TrxG proteins have been identified (13,14). *Drosophila* PREs are several hundred base pairs long, with little sequence homology between them (1). Nonetheless, they are enriched in the binding motifs for several DNA binding factors. PcG proteins in *D. melanogaster* include Pc (Polycomb), Psc (Posterior sex combs), Pho (Pleiohomeotic) and Sfmbt (Scm-related gene containing four mbt domains) (13). Pho is the only PcG protein known to bind DNA with sequence specificity (18). PcG proteins form three major complexes on chromatin: Polycomb Repressive Complex 1 (PRC1) (19), Polycomb Repressive Complex 2 (PRC2) (20–23) and Pleiohomeotic Repressive Complex (PhoRC) (24). Polycomb repressed chromatin is marked by histone 3 lysine 27 trimethylation (H3K27me3) (20–23). Trithorax activated chromatin is marked by histone 3 lysine 4 monomethylation (H3K4me1) (25) or dimethylation (H3K4me2) (26).

*Drosophila* PREs were originally discovered by testing segments of DNA for their ability to maintain previously established transcription states when taken out of their endogenous context (4,5). In 2003, Ringrose *et al.* published a computational method to model PRE sequences, named the PREdictor, which predicted 167 candidate PREs genome-wide in *D. melanogaster* for one expected false-positive prediction (9). The PREdictor scores sequence windows by a linear combination of motif pair occurrence frequencies, weighted by log-odds of occurrence frequencies in a training set of PREs and non-PREs. Ringrose *et al.* trained the PREdictor on a set of 12 PREs (11 PREs from *D. melanogaster* and 1 from *D. virilis*) and 16 non-PREs (promoters that are enriched in PRE sequence motifs but that do not recruit Polycomb), together with a set of seven motifs. Six of these motifs correspond to DNA binding factors (two for GAGA binding factor, three for Pleiohomeotic, one for Zeste), and one is a motif that was identified by conservation between *D. melanogaster* and *D. virilis* in the engrailed PRE and whose deletion abrogates silencing function (EN1) (27). The authors found that paired motif occurrence frequencies can distinguish PREs from non-PREs, whereas single motif occurrence frequencies cannot. This suggests that the sequence criteria for recruiting Polycomb are of a combinatorial nature and that DNA binding factors cooperate on PREs to recruit Polycomb regulatory complexes. Furthermore, Ringrose *et al.* identified several new candidate PRE sequence motifs, including the GTGT motif. Since then, the GTGT motif has been shown to be essential for silencing in the *vg* PRE (28), and it has been shown to be bound by the sequence-specific DNA binding protein Combgap, which is involved in PcG recruitment (29). The GTGT motif has also been rediscovered as the CACA motif in a ChIP-on-chip study of genome-wide binding profiles of PcG and other proteins (30). The PREdictor (9) method was later extended to the jPREdictor (31), a re-implementation in Java, providing a graphical user interface and offering the ability to flexibly define motifs and their combinations.

In 2012, Zeng *et al.* published the EpiPredictor (32), a PRE predictor that uses the machine learning method of Support Vector Machines (SVMs). Support Vector Machines model feature space class boundaries by placing a decision surface between the points of two classes such that the margin to the closest points is maximized, with room for treating data points as noise by use of a soft margin, and with the possibility of non-linear modelling by use of kernel functions (33). The EpiPredictor filters sequence windows using the SVM and a GC-content filter and scores them based on the total number of motif occurrences they contain. The SVM feature space consists of single motif occurrence frequencies. The EpiPredictor was trained on the same set of PREs and with the same motifs as used by Ringrose *et al.* (9). Zeng *et al.* (32) found that non-linear kernels distinguish PREs from non-PREs better than linear kernels, adding further evidence of the importance of motif occurrence combinatorics for PRE sequences.

Recent advances in experimental methods have led to the publication of several sets of candidate PREs genome-wide in *Drosophila* (10,11,30,34–38). These methods include chromatin immunoprecipitation (ChIP) combined with microarray (ChIP-chip) (39), ChIP combined with high-throughput sequencing (ChIP-seq) (40) and DNA adenine methyltransferase identification (DamID) (37).

The published candidate PRE sets vary in the number and identity of candidate PREs they contain (1,12). Several factors may underlie these discrepancies, such as differences in experimental methods (ChIP-chip versus ChIP-seq) or differences in antibodies used. The results of experimental mapping methods also depend on the cells being studied and on their genetic states. Furthermore, PREs physically interact with other genomic loci, forming higher-order structures (41). Experimental mapping methods do not discriminate between recruiting and interacting sites and can as a result capture regions that PREs interact with, in addition to the PREs themselves (1). *In silico* PRE prediction methods have no such limitations and can help us to understand the sequence criteria for what constitutes a PRE.

Sequences that recruit PcG proteins in other organisms are also being studied, though few mammalian PREs have so far been identified (15). PcG recruitment has been modelled in human embryonic stem cells using Support Vector Machines (42). In the frog *Xenopus tropicalis*, Support Vector Machines were able to identify a *k*-mer spectrum that characterizes H3K27me3 nucleation sites that are not CpG islands and that work as repressive elements when taken out of their endogenous context (43). Du *et al.* (44) reported three classes of response elements in human: Polycomb Response Elements (PREs), Trithorax Response Elements (TREs) and Polycomb/Trithorax Response Elements (P/TREs).

Previous publications on modelling *Drosophila* PREs have used small sets of experimentally tested PREs for training the models. The resulting genome-wide predictions have limited overlaps with genome-wide experimentally determined PcG-recruiting chromatin regions. Furthermore, the GTGT motif has not previously been included in *Drosophila* PRE sequence models. We here seek to refine the state of the art in DNA sequence models of *Drosophila* Polycomb Response Elements by investigating whether the training of sequence models on genome-wide experimentally determined PcG-recruiting DNA and including the GTGT motif increases the agreement between *in silico* PRE predictions and independent experimentally determined genome-wide sets of PcG target regions. We further address the question whether a more advanced modelling approach can additionally improve model generalization and present a new method for modelling *cis*-regulatory elements, SVM-MOCCA.

## MATERIALS AND METHODS

### Nomenclature of response elements

The nomenclature of response elements is evolving. Chang *et al.* (45) identified a 440-bp fragment in the *postbithorax/bithoraxoid* region of *Ultrabithorax* that contains both a PRE (Polycomb Response Element) and a TRE (Trithorax Response Element). Tillib *et al.* (46) distinguish TREs and PREs as discrete sequences in a TRE-PRE module. The closeness of PREs and TREs is described by (47) as an ‘intermingling of elements’, and the authors propose that PREs/TREs acquire the new name ‘maintenance elements’, to reflect their dual function. Bloyer *et al.* (48) conclude that (then) recent data strongly suggests that ‘each PRE/TRE is composed of multiple different *cis*-DNA modules, which can be bound by different subsets of PC-G and TRX-G at defined spatial and temporal positions in the embryo’. While some authors consistently use the term PRE/TRE (1), emphasizing the dual nature of these maintenance elements, others primarily use the term PRE and conclude from experimental data that ‘PREs are also TREs’ (34). Enderle *et al.* (35) present a set of ‘PcG binding sites’ that is not only defined on the basis of proteins from the Polycomb group, but also on TRX-C, and also use the term PRE. Kahn *et al.* (36) also use the term PRE for regions defined from overlapping peaks of E(Z), TRX and PC and coinciding with H3K27me3 domains. It thus appears that more recently, the term PRE is universally used for PcG target sites that can also be TrxG target sites and have potential to be both Polycomb and Trithorax Response Elements (with the caveat that the response function of these sites has not been tested). In accordance with this, we primarily use the term PRE (Polycomb Response Element), but mean it to encompass such elements’ potential function as TREs.

### Genome assembly

We used the *D. melanogaster* genome assembly release 6 (2014) (49,50). All published genomic coordinates that we considered that were for a previous genome assembly were converted to release 6 using the FlyBase (51) coordinate converter.

### DNA sequence motifs

We used motifs defined in IUPAC notation (52), as used or reported in Ringrose *et al.* (9): EN 1: GSNMACGCCCC (one mismatch allowed), G10: GAGAGAGAGA (one mismatch allowed), GAF: GAGAG, PF: GCCATHWY, PM: CNGCCATNDNND, PS: GCCAT, Z: YGAGYG, GTGT: GTGT. Throughout the manuscript, when comparing classifiers with and without the GTGT motif, those with have been marked ‘w. GTGT’. SVM-MOCCA always makes use of this motif and has not been marked explicitly.

For comparison experiments, we also used the following motifs, reported in (53): one additional motif for Zeste: BGAGTGV, one for Sp1/KLF: RRGGYG, one for Dsp1: GAAAA, two for Grainyhead: TGTTTTTT and WCHGGTT, and one for ‘site A’: GAACNG.

To investigate how the addition of GTGT to a PRE model compares to adding a random 4-mer, we randomly generated 19 unique 4-mers (unique also when considering reverse complements).

### Sequence-generating nth-order Markov chains

For every *n*-mer *s* (a DNA sequence of length *n*), we obtained the probability of observing each nucleotide $q \in \lbrace \mathtt {A}, \mathtt {T}, \mathtt {G}, \mathtt {C} \rbrace$ next as the fraction of times we observe *q* after *s* versus the total number of observations of *s*. To account for double-strandedness, we also obtained *n*-mer frequencies on the reverse complement of each sequence. We added a pseudocount of 1 for each nucleotide for each *n*-mer to ensure none had zero observations. To generate a sequence, we randomly picked an *n*-mer with the probability of observing this *n*-mer, and generated each subsequent nucleotide based on the nucleotide probability distribution for the last generated *n*-mer.

### Training and validation sequences

We acquired the training set used by Ringrose *et al.* (9), consisting of 12 PREs and 16 non-PREs, henceforth referred to as the T2003 training set.

Additionally, we acquired genome-wide candidate PcG target sites determined by Schwartz *et al.* (34), Enderle *et al.* (35) and Kahn *et al.* (36). We considered including data from Schuettengruber *et al.* (30), but as they did not publish candidate PRE coordinates and we already consider three more recently published PRE sets, we opted not to include their data in our analysis. For the Schwartz *et al.* (34) set, computationally defined PREs were downloaded from the article’s Supplementary Table S6, and coordinates were converted from *D. melanogaster* genome assembly 4 to assembly 6. PcG target regions from the Enderle *et al.* (35) set were acquired from the article’s Supplementary Table 3 and converted from *D. melanogaster* genome assembly 5 to assembly 6. The Kahn *et al.* (36) set of computationally defined PREs was extracted from the article’s Supplementary Table S1 and converted from genome assembly 5 to assembly 6. All coordinate conversions between genome assemblies were performed using the FlyBase (51) coordinate converter. Only regions localized on chromosomes 2L, 2R, 3L, 3R, 4 and X were considered. Heterochromatic regions (‘Het’ chromosomes in the FlyBase annotation) were discarded. After coordinate conversions, in order to account for any distancing between recruited factors and recruiting sequences, all regions were resized to a length of 3 kb each (1.5 kb bidirectionally from each region center), and corresponding sequences were extracted from the assembly 6 genome.

We generated three sets of negative control sequences for training and testing: (a) For each PcG target region set, we generated a set of one hundred times as many 3 kb-long random sequences, using a fourth-order Markov chain trained on the respective set, henceforth referred to as dummy PREs. (b) A fourth-order Markov chain was trained on the *D. melanogaster* genome and used to generate a set of a hundred times as many 3 kb-long random sequences as in the largest Polycomb target set (20 100 sequences in total), henceforth referred to as dummy genomic sequences. Dummy sequences mirror average 5-mer distributions of their set of origin, but are unlikely to retain any higher-order structure such as motif pairing or clustering. (c) Finally, we acquired coding sequences from the FlyBase (51) r6.04 annotation. In order to get a set of uniformly sized coding sequences for training and testing, we concatenated the coding sequences and split the resulting sequence into non-overlapping 3 kb-long fragments, henceforth referred to as coding sequences. Additionally, in order to have a coding sequence region set to check for genomic overlaps with predictions, unlikely to contain gene-proximal PREs, we defined core coding sequences as annotated coding sequences shrunk bi-directionally by 250 bp, with regions too small to shrink omitted.

We refer to training sets consisting of PREs from a genome-wide experimental set and corresponding dummy PREs by the name T2017. For the main figures, T2017 refers to the Schwartz *et al.* (34) set of PREs and of corresponding dummy PREs as controls. For supplementary figures where we train models on the Enderle *et al.* (35) and Kahn *et al.* (36) sets, the meaning of T2017 is modified to refer to the specified PRE set and corresponding dummy PREs.

### Cross-validation

To account for random variation in generalization performance, we cross-validated with 50 repetitions, resulting in 50 sets of independent training and test sequences. Over cross-validation, each sequence set was randomly shuffled, and the first 110 sequences were reserved for training. Of the remainder, the first 50 PRE sequences and 5000 non-PRE sequences of each set were used for testing. This 100:1 ratio of controls to PREs reflects the expected genome-wide context, based on the assumption that the 140 Mb-long *D. melanogaster* genome contains 1400 1 kb-long PREs. Note that the precise number is neither known nor necessary to be known for this analysis, since any number between a few hundred and a few thousand PREs in the *Drosophila* genome will be reflected accurately enough in the performance evaluations.

### Classifier performance evaluation

When testing model generalization, we applied our models using a sliding window across all test sequences, where the maximum window score was taken as the final test sequence score. When visualizing model generalization, we focused on Precision/Recall curves (PRCs), which plot Precision = TP/(TP + FP) in the Y-axis and Recall = TP/(TP + FN) in the X-axis. TP denotes the number of true positives, FP the number of false positives and FN the number of false negatives. PRCs, unlike ROC (Receiver Operating Characteristics) curves, are informative of generalization performance on highly imbalanced datasets, such as genome-wide predictions, where the number of positives is small compared to the number of negatives (54). The area under the Precision/Recall curve (PRC AUC) gives a threshold-independent measure of expected classifier generalization. Note that, as a consequence, PRC AUC does not refer to any particular number of predictions nor to any particular number of true and false positives. Rather, such numbers correspond to a point on the Precision/Recall curve. Depending on requirements, e.g. with respect to an expected precision, a score cutoff can be chosen which will then determine specific numbers such as the number of predictions and true and false positives. We use the mean PRC AUC over cross-validation, with 95% confidence intervals calculated based on normally distributed means.

### CPREdictor

We have reimplemented the PREdictor (9) algorithm in C++, following the formulation given in (9) and in the jPREdictor (31) source code. We henceforth refer to our implementation as the CPREdictor. The CPREdictor has been tested for functional equivalence with PREdictor and jPREdictor, in order to ensure comparability.

### SVM-MOCCA

The Support Vector Machine Motif Occurrence Combinatorics Classification Algorithm (SVM-MOCCA) constructs one Support Vector Machine (SVM) per motif in order to model local sequence composition around motif occurrences in a target class versus one or more negative classes. Given a DNA sequence, a feature vector is constructed for each occurrence of each motif, consisting of occurrence frequencies of motifs and dinucleotides within 250 bp of the occurrence, giving a feature space in |*M*| + 4^2^ dimensions for a set of |*M*| motifs. For a given set of training sequences, each motif SVM is trained on all occurrences of its respective motif in the training sequences, with the view of predicting the sequence class (positive or negative) of a motif occurrence.

Once each SVM has been trained, occurrences of all motifs in the training set are classified by the corresponding SVMs. Let *M* denote a set of motifs, *P* and *N* sets of positive and negative training sequences, respectively, and *f*(*m, s*) the frequency of positively classified occurrences of motif *m* in sequence *s*. For each motif *m* ∈ *M*, a weight is calculated as

\begin{equation*} w_m = \log \frac{ \sum _{p \in P} f(m,p) / |P| }{ \sum _{n \in N} f(m,n) / |N| }. \end{equation*}

Given a sequence to classify, feature vectors are constructed for all motif occurrences in the sequence, which are in turn classified by their corresponding SVM. Frequencies of positively classified motif occurrences, *f*(*m*) for a motif *m*, are weighted and summed, giving a score for the sequence:

\begin{equation*} S = \sum _m w_m f(m). \end{equation*}

We used LibSVM (55) for the Support Vector Machine implementation. SVMs were trained with linear kernels and also with polynomial kernels with degrees 2 and 3 (henceforth referred to as quadratic and cubic kernels, respectively). As SVMs support the use of more than two classes, we used PREs together with all three control classes for training (dummy PREs, dummy genomic sequences and coding sequences).

When more than two classes are used, each SVM models all class boundaries using binary SVM classifiers, and the class of each motif occurrence is predicted by majority vote, as implemented in LibSVM (55). One of the classes is designated as positive and the remaining classes as negative, giving a binary classification.

### Prediction threshold calibration

We considered the model trained for cross-validation fold 1. The test set PREs were taken as positives. For the calibration negatives, we trained a fourth-order Markov chain on the *D. melanogaster* genome, and we generated 44 626 sequences, each 3 kb long, adding up to approximately the size of the *D. melanogaster* genome, at a total of 133.9 Mb. We searched the precision/recall space for the threshold with highest recall for the desired precision, with linear interpolation if necessary. For reasons of stability, we took the mean threshold over 10 repetitions of random-genome construction.

### Genome-wide prediction

We applied each classifier across chromosomes 2L, 2R, 3L, 3R, 4 and X using a sliding window, with a step size of 10 bp, and a window size determined by the classifier. Windows with a score above the classifier threshold were noted as predictions, and overlapping predictions were merged into non-overlapping predicted candidate PREs.

### Chromatin accessibility

We acquired DNaseI-seq data from the Berkeley Drosophila Transcription Network Project (BDTNP) (http://bdtnp.lbl.gov:8080/Fly-Net/access.jsp) for five different developmental stages (embryonic stages 5, 9, 10, 11 and 14). For a given set of regions, we defined accessible regions of the set as the subset of regions that overlap with regions in at least one of the five DNaseI-seq sets.

### Genomic region overlaps

To measure genomic region overlaps between two sets A and B, we took the subset of regions in A that overlap with at least one region in B by at least one base pair. When comparing predictions to published genome-wide data sets, in order to account for potential distancing of recruited factors from recruitment sites, we extended regions in the published sets bi-directionally by 1 kb before checking overlaps (with the exception of modENCODE histone marks).

### ModENCODE data sets

We acquired GFF/GFF3 genomic coordinate files from modENCODE (56) for *D. melanogaster*: H3K27me3 (13 sets); H3K4me1 (10 sets); H3K4me3 (14 sets); Pc (Polycomb) (6 sets); Psc (Posterior sex combs) (3 sets); dSFMBT (2 sets). The full paths from the modENCODE FTP archive are given in Supplementary Table S1. The datasets were downloaded in April 2016, and later datasets were not considered. The sets include data from animals (Adult-Female, Adult-Male, Embryos-0-12-hr, Embryos-0-4-hr, Embryos-12-16-hr, Embryos-14-16-hr-OR, Embryos-16-20-hr, Embryos-2-4-hr-OR, Embryos-20-24-hr, Embryos-4-8-hr, Embryos-8-12-hr, Larvae-3rd-instar, Larvae-L1-stage, Larvae-L2-stage, Larvae-L3-stage, Late-Embryonic-stage), as well as cell-lines (ML-DmBG3-c2, S2-DRSC).

### Extraction of PRE predictions with biologically relevant signals

For each set of predictions by CPREdictor T2017 w. GTGT and SVM-MOCCA (Supplementary Files 1 and 3), we extracted the subsets of predictions that overlapped both with at least one H3K27me3 peak and with at least one peak of Pc, Psc or Sfmbt. For the H3K27me3, Pc, Psc and Sfmbt signals, we used merged sets of peaks from modENCODE as noted above. The resulting sets of candidate PREs are henceforth referred to as CPREdictor T2017 w. GTGT HC (1036 candidate PREs; Supplementary File 2) and SVM-MOCCA HC (2908 candidate PREs; Supplementary File 4), respectively, with ‘HC’ standing for ‘high-confidence’. In addition, we extracted predictions enriched in H3K4me1 as candidate TREs (Supplementary Files 10 and 11).

### Core sequence fragment prediction

From the 3 kb-long (or longer when merged) SVM-MOCCA predictions, we identified the most predictive sub-regions, henceforth referred to as SVM-MOCCA HC Core (Supplementary File 5). We applied SVM-MOCCA to its genome-wide predictions, with an iteratively larger window size from the following sequence of sizes: 500 bp, 600 bp, 750 bp, 1 kb, 1.5 kb, 2 kb, 2.5 kb, 3 kb, and with a step size of 50 bp. The highest-scoring window for each window size was collected, and the overall maximally scoring window (with the score normalized by window length), was defined as the core sequence.

### Target gene prediction

We acquired the FlyBase genome annotation release R6.04. For a given region, any gene overlapping with a region was defined as a candidate target gene. For each region that did not overlap with any gene, the gene closest to the region (as determined by the closest region and gene endpoints) was defined as a candidate target gene.

Candidate PcG target genes were predicted for the complete PRE prediction sets from CPREdictor T2003, CPREdictor T2017, CPREdictor T2017 w. GTGT (Supplementary File 6) and SVM-MOCCA (Supplementary File 7).

### Target genes from other publications

We downloaded published sets of predicted PcG target genes for PREdictor (9) and EpiPredictor (32), and from Schwartz *et al.* (34) and Enderle *et al.* (35).

The Schwartz *et al.* (34) PcG target genes were extracted from Supplementary Tables S2 and S4 from their article (class I and class II high-confidence PcG target genes, respectively), and these two sets were merged. For the Enderle *et al.* (35) set, target genes were extracted from the article’s Supplementary Table 4 (first column). Genes that could not be found in the FlyBase (51) r6.04 annotation were omitted. No further validation was performed on sets, except for the predictions from (9), which we validated using FlyBase, giving higher numbers of genes recognized in the annotation we used. Since no target genes were published in (36), we predicted target genes for that study by proximity, following the same procedure as for our own PRE predictions.

### Gene ontology analysis

A list of all gene names was extracted from the FlyBase (51) r6.04 annotation. For each set of candidate PcG target genes, gene ontology analysis was performed using GOrilla (57) with two unranked lists of genes, where the first was the list of candidate PcG target genes and the second was the list of all annotated genes.

### Software and packages

**Figure 1.**
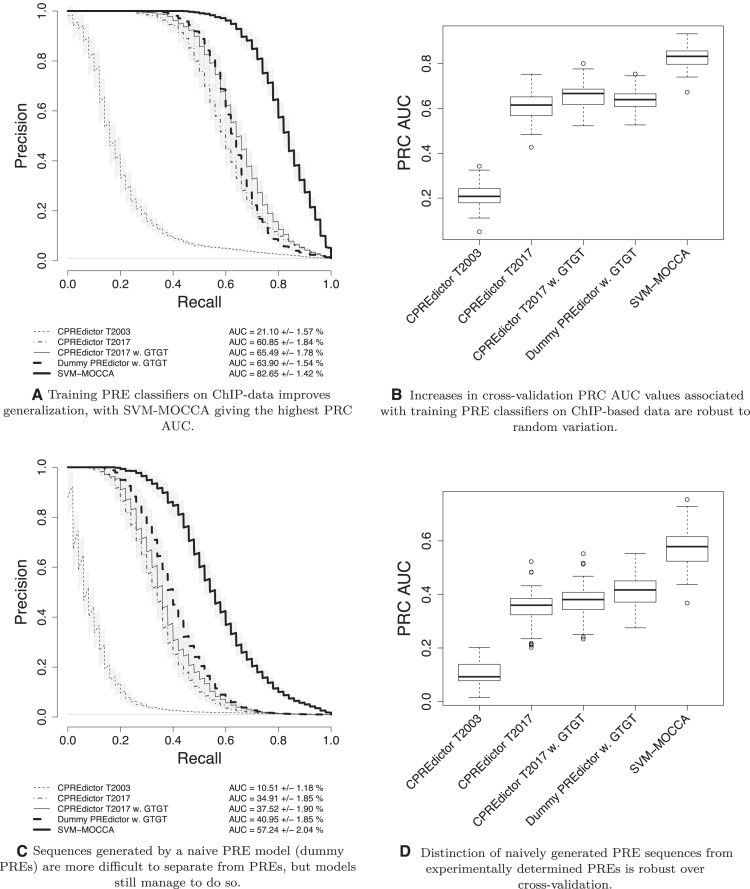
Classifier generalization when trained on genome-wide experimental data for PRE prediction. (**A**) Average Precision/Recall plot for classifiers applied to PREs determined by Schwartz *et al.* (34) (independent from training set PREs) versus 100 times as many control sequences generated by a fourth-order Markov Chain trained genome-wide, as according to the plot legend. Average curves over all 50 folds are shown, together with 95% confidence intervals for the mean precision. AUC values are percentages rounded to two digits. (**B**) PRC AUC box plot for multiple classifiers over all 50 folds. (**C**) Average Precision/Recall plot for PREs determined by Schwartz *et al.* (34) (independent from training set PREs) versus 100 times as many sequences generated randomly using a fourth-order Markov Chain trained on PREs, constituting a naive PRE model (dummy PREs). Average curves over all 50 folds are shown, together with 95% confidence intervals for the mean precision. (**D**) PRC AUC box plot for multiple classifiers over all 50 folds.

**Figure 2.**
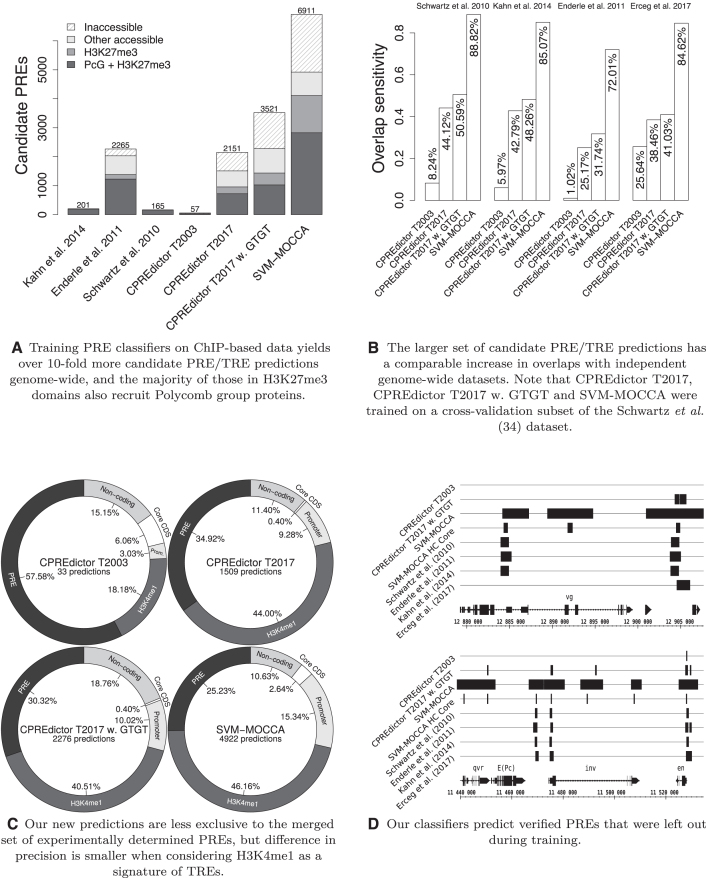
Results of genome-wide candidate PRE/TRE prediction for an expected precision of 80%. (**A**) Numbers of experimentally determined and computationally predicted candidate PREs. Accessible portions in Polycomb repressed domains (H3K27me3) have been marked, as well as the portions of those regions that are enriched in Polycomb. Chromatin accessibility was derived from DNaseI-seq data; see Materials and Methods, also for H3K27me3 and Polycomb datasets. (**B**) Overlap sensitivity of each classifier’s predictions to two genome-wide, experimentally determined candidate PRE sets (35,36) and a set of functionally validated PREs (69) (see Materials and Methods for the definition of these three sets). Overlap sensitivity is defined as the fraction of regions in an experimental set that are overlapped by at least one prediction. (**C**) Proportions of the sets of predictions that overlap with different genomic loci. Only predictions in accessible chromatin are considered. The merged set of experimentally determined PREs by Kahn *et al.* (36), Enderle *et al.* (35) and Schwartz *et al.* (34) are considered first, and from the leftover, H3K4me1, then promoters, then core CDS; the final leftover set of predictions is marked as non-coding. See Materials and Methods for H3K27me3 datasets. Promoters are predicted as 3 kb upstream to 0.5 kb downstream from annotated gene transcription start sites. Core CDS is annotated coding sequence (CDS) shrunk bi-directionally by 250 bp (see Materials and Methods). (**D**) *invected*/*engrailed* and *vestigial* loci, visualized with the Integrated Genome Browser (63).

**Figure 3.**
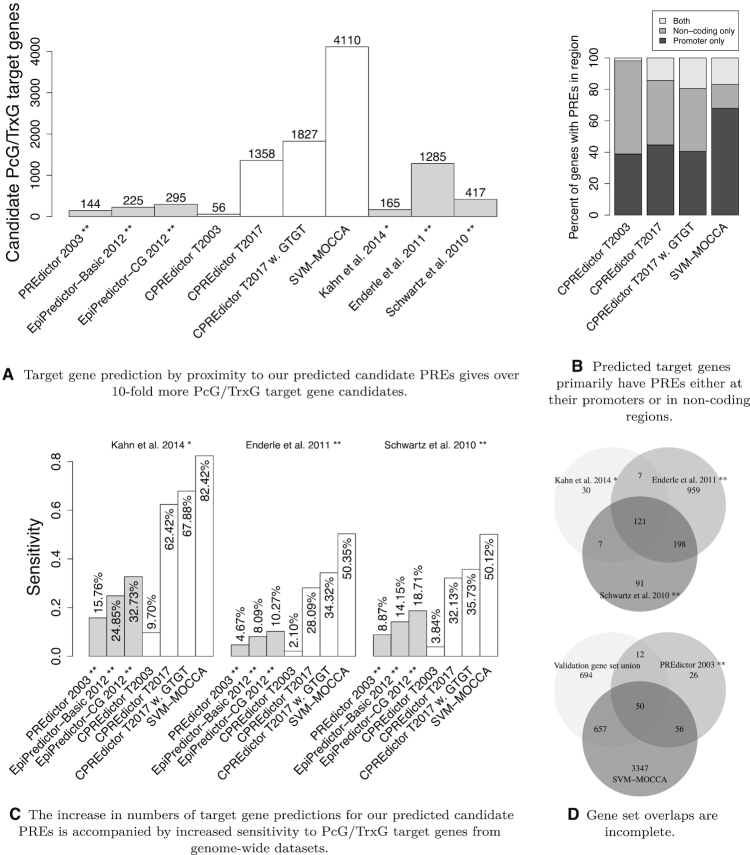
PcG/TrxG target gene prediction results. (**A**) Numbers of target genes predicted by each algorithm, as well as in each experimentally published set. (**B**) Fractions of predicted target genes that have predicted PREs in either promoter regions (TSS −3 kb/+0.5 kb), in non-coding regions (not on promoters or core coding regions) or both. (**C**) Sensitivity of each classifier target gene prediction set to experimentally determined sets. Sensitivity is defined as the fraction of experimentally determined genes that are also predicted. * Kahn *et al.* (36) did not publish a set of genes, so we predicted target genes by proximity. ** Genes that were not found in the current annotation were omitted. (**D**) Venn diagrams of gene set overlaps for validation gene sets and target gene predictions.

All figures except for Figure 2D were generated using R (58). The Precrec (59) library was used for generating average Precision/Recall curves and corresponding confidence intervals (Figure 1A and C). The Plotrix (60) library was used when generating the pie charts in Figure 2C. For generating the Venn diagrams in Figures 3D and Supplementary Figure S11, the VennDiagram (61) library was used. Tomtom (62) was used to search for factors that bind a *k*-mer. Gene ontology analysis was performed using GOrilla (57). The *vestigial, invected* and *engrailed* loci in Figure 2D were visualized using the Integrated Genome Browser (63).

## RESULTS

### Training sequence models on genome-wide PcG target sites improves PRE sequence model generalization

We wanted to see how models trained on genome-wide experimentally determined PcG-enriched regions compare to models trained on the Ringrose *et al.*(9) set of PREs, in terms of their ability to distinguish independent experimentally determined PcG-enriched regions from different classes of background sequences. To this end, we extracted genomic sequences for PcG-enriched regions from three publications (34–36), as described in Materials and Methods. We focus on the (34) set for training. Our models are discriminative, necessitating a set of non-PREs for training. We used three classes of non-PRE sequences for training and testing: (a) dummy PREs, (b) dummy genomic sequences and (c) coding sequences, as described in Materials and Methods. Dummy PREs, due to their motif composition being similar to that of PREs, form the strictest of our control sets, but are also unlikely to retain the characteristic motif occurrence clustering that has been found to be predictive of PREs (9). We thus assume that dummy PREs are unlikely to model functional PREs, and we include this set in the training of all of our models. Core coding sequences have zero or close to zero overlaps with experimentally determined PRE sets when promoter-overlapping PREs are omitted (data not shown). We speculate that any overlaps of PREs with coding sequences are due to promoter-promixal PREs, lack of positional precision for ChIP data, and factor mobility, rather than that PREs occur in coding sequences. With this assumption, coding sequences constitute a set of real genomic sequences that are unlikely to contain PREs. Both dummy genomic sequences and coding sequences share only minimal resemblence with PREs, making them more null than dummy PREs. We thus focused most of our attention on training with dummy PREs, but we include dummy genomic and coding sequences in our model evaluation and when training multi-class models, as independent control sets. This enabled us to investigate any over-fitting to dummy PREs that may occur and to train multi-class models.

In order to test model generalization, we split the PRE and control sets into independent training and test sets, with 50-fold cross-validation to account for random variation, and a 1:100 ratio of PREs to non-PREs to reflect the expected genome-wide context, as described in Materials and Methods.

When training the CPREdictor algorithm on the T2017 set, using the same motifs as Ringrose *et al.* (9), and evaluating the trained classifiers on independent cross-validation PREs versus dummy genomic sequence controls, we observed a 2.9-fold increase in the mean Area Under the Precision Recall Curve (PRC AUC) compared to training with the training set used by Ringrose *et al.* (9) (T2003) (Figure 1A). This increase in PRC AUC is robust over cross-validation (Figure 1A and B), with non-overlapping 95% confidence intervals of the mean PRC AUCs (Figure 1A). We also observed increased PRC AUC for T2017 when evaluating with dummy PRE controls (Figure 1C and D) and coding sequence controls (Supplementary Figure S1).

These results demonstrate that training models on genome-wide experimentally determined PcG target sites, and with controls generated by a fourth-order Markov chain trained on those sites, results in models that better distinguish independent PcG target sites (from the same set) from genomic background and PRE-like non-PRE sequences than models trained on the set compiled by Ringrose *et al.* (9). Training and evaluating PRE sequence models using other published sets of PcG-recruiting regions shows the same trend, where models trained on PcG-recruiting regions generalize better to independent PcG target regions than models trained on the T2003 set (Supplementary Figure S2).

### The improvement in model generalization is independent of training set size

To determine the influence of training set size on generalization performance, we additionally trained the CPREdictor using sets of 12 and sets of 50 PRE and control sequences each. We observed only negligible differences in generalization performance across the sets of 12, 50 and 110 training sequences (mean PRC AUCs, with 95% confidence intervals, were 34.62 ± 1.84%, 34.79 ± 1.82% and 34.91 ± 1.85%, respectively; all values from an evaluation against dummy PREs; compare Figure 1C), demonstrating that training set size does not play a role in generalization performance and suggesting that the T2017 training set is qualitatively different from the T2003 training set.

### The choice of negative training sequences is instrumental in PRE prediction performance

It is interesting to ask how models trained with the training set used by Ringrose *et al.* (9) fare compared with models trained using their set of PREs and randomly generated non-PRE sequences (dummy PREs). To test this, we trained a fourth-order Markov chain on the Ringrose *et al.* (9) training set PREs, and we randomly generated 12 dummy PREs, each with length equal to the mean PRE length (2914 bp). After training CPREdictor on this training set, we observed increased generalization to the Schwartz *et al.* (34) set versus dummy PREs compared to when using the negative training set from Ringrose *et al.*, with PRC AUCs close to those obtained when training with ChIP-based data (Supplementary Figure S3). In summary, combining the 2003 positive training sequences with dummy PREs derived from these as negatives generalizes better to independent PcG targets than models trained with the original 2003 positive and negative training sets, demonstrating that the choice of negative training sequences is instrumental in PRE prediction performance.

### Including the GTGT motif improves PRE sequence model generalization

The accumulating evidence for the GTGT motif being a component of Polycomb regulation (9,28–30) prompted us to investigate whether the inclusion of the GTGT motif in our PRE sequence models improves generalization to independent PREs. When we added the GTGT motif to our CPREdictor T2017 model, we observed an additional 1.1-fold increase in the mean PRC AUC on independent PREs and dummy genomic sequences in comparison to the T2017 model without the GTGT motif (Figure 1A). This increase is robust over cross-validation, different PRE sets and different control classes (Figure 1A–D, and Supplementary Figures S1 and S2). In summary, the inclusion of the GTGT motif in PRE models improves generalization across different training and test sets, providing additional evidence that this motif plays an important role in Polycomb regulation.

### The improvement in model generalization cannot be attributed to increased model complexity, and GTGT performs better than other reported motifs

To assess the degree to which the improvement in generalization performance upon adding the GTGT motif might be explained by the increased model complexity (owing to the inclusion of a motif and the associated parameters), we added random 4-mers to our CPREdictor T2017 model. The inclusion of GTGT resulted in a 1.04-fold to 1.16-fold increase in the mean PRC AUC over the inclusion of 18 out of 19 other unique, randomly generated 4-mers, for Schwartz PREs versus dummy genomic controls (Supplementary Figure S4), demonstrating that the GTGT motif contributes to a performance improvement beyond that expected from increased model complexity. The only 4-mer that gave higher PRC AUC was GGCG. Searching for *D. melanogaster* factors that bind GGCG using Tomtom (62) gave Brinker (Brk) as a match (*P*-value = 7.78e–04), a transcriptional repressor of Dpp target genes (64–66). We also tested six other published motifs that have been associated with PcG recruitment: one additional motif for Zeste, one for Sp1/KLF, one for Dsp1, two for Grainyhead and one for ‘site A’ ((53) and references therein; see also Materials and Methods). The GTGT motif gives the largest improvement in model generalization (1.06-fold to 1.10-fold higher PRC AUC compared with the inclusion of the other motifs, for Schwartz PREs versus dummy genomic sequences), while the other motifs affect model generalization to only a smaller extent and similarly to each other (PRC AUCs range from 59.73% to 61.96% for Schwartz PREs versus dummy genomic controls, and the majority of the confidence intervals overlap with one another) (Supplementary Figure S5), suggesting that the GTGT motif plays a more decisive role in PcG recruitment.

### Genome-wide PcG target sites and Ringrose *et al.* training PREs have different sequence properties

Given that the models trained with the T2017 set and the GTGT motif and those trained with the T2003 set showed highly different generalization abilities to independent PcG target sites, we were interested in how the models differ and what might cause the difference in generalization ability. We thus investigated the weights of CPREdictor models trained with the T2003 set and T2017 set, and also with PREs from the T2003 set and generated non-PREs (Supplementary Figure S6).

We found a moderate negative correspondence between motif pair weights assigned using T2003 versus T2017 (Pearson’s correlation coefficient < –0.5). Weight correlation when using T2003 PREs and generated non-PREs versus when using the T2017 set is low (Pearson’s correlation coefficient < 0.4). For T2003 versus when using T2003 PREs and generated non-PREs, correlation is similarly low (Pearson’s correlation coefficient < 0.4). Whereas the T2003 model has three negatively weighted motif pairs (G10:G10, G10:GAF and GAF:GAF), with all three weights being substantial, the T2017 model has two (PM:PM and PS:GTGT), both with weights close to zero. In fact, the most negatively predictive T2003 motif pair, G10:G10, is the most highly weighted motif pair for the T2017 model. The discrepancy might be due to clusters of GAF motifs in the negative training set in (9) which includes promoters of genes that are regulated by GAF and Z (9). The small size of the T2003 set can result in one or a few more pair occcurrences in the negative training set compared to the positive training set which would have a large influence on the final model weights. The seven highest weighted motif pairs in the T2003 model all include Pho binding site variants (PF:PM, GAF:PF, PM:PS, G10:PM, G10:PF, PF:PF and PM:Z). These weights have approximately been reduced by half or more for the T2017 model, and the top four highest weighted motif pairs for the T2017 model do not include any Pho binding site variants and are instead enriched for G10 (G10:G10, G10:GAF, G10:Z, G10:GTGT). The dominance of G10 in the top T2017 motif pair weights may in part be attributed to properties of control sequences generated by Markov chains of fixed order and the long length of G10. Models trained using the T2003 versus T2017 sets are thus dissimilar, meaning that motif composition is different in the training sets.

### Models trained with genome-wide PcG targets can distinguish Ringrose *et al.* training PREs from background

As the models trained on T2003 and T2017 are so different, we wanted to see how our models score the training set used by Ringrose *et al.* (9). The models that we trained on ChIP data versus dummy PREs have lower PRC AUC to the Ringrose *et al.* (9) training set than does CPREdictor trained on this set, but PRC AUCs are still above random (Supplementary Figure S7). The best generalization to the Ringrose *et al.* (9) training set that we observe for models not trained on this set is for CPREdictor including the GTGT motif, with a mean PRC AUC of $70.20 \pm 0.99 \%$. The lowest is for SVM-MOCCA, with a mean PRC AUC of $61.75 \pm 1.14 \%$. We also investigated the degree to which our models can distinguish the Ringrose *et al.* (9) PREs from dummy PREs. For this case, CPREdictor with GTGT and SVM-MOCCA obtain the highest PRC AUCs, at $81.23 \pm 0.26 \%$ and $98.45 \pm 0.32 \%$, respectively. In conclusion, our models are still able to distinguish the set of PREs and non-PREs used by Ringrose *et al.* (9), though to a lower degree than CPREdictor trained on this set, and our models are better at distinguishing the Ringrose *et al.* (9) PREs from randomly generated controls.

### Uniformly weighted motif pair clustering distinguishes PREs from background

Considering the large differences in model weights obtained when using T2003, T2017 and a set consisting of PREs from T2003 and generated non-PREs, we wanted to see how well a uniformly weighted PREdictor model would distinguish PREs from non-PREs. We thus constructed a PREdictor model with all weights set equal to 1, henceforth referred to as the Dummy PREdictor. We found that the Dummy PREdictor generalizes comparably to CPREdictor trained with T2017 when including the GTGT motif and testing with Schwartz PREs as positives and dummy genomic sequences as negatives (Figure 1A). When we evaluate our models using Schwartz PREs as positives and dummy PREs as negatives, where the CPREdictor has been trained with this set, the Dummy PREdictor outperforms the CPREdictor (Figure 1C ). This was a surprise to us, as we expected a trained model would have an advantage, with weights fitted both to PREs and a randomly generated non-PRE distribution. The Dummy PREdictor corresponds to a uniformly weighted motif pair clustering.

### A more advanced PcG target site sequence model improves generalization

We have developed SVM-MOCCA (see Materials and Methods), a new method for modelling *cis*-regulatory elements, and we wanted to test how such a more advanced modelling method would fare in modelling PcG target sites in comparison to the CPREdictor.

We trained SVM-MOCCA using the T2017 set with all three control classes and with the motifs used by Ringrose *et al.* (9), with the addition of the GTGT motif. The training sequences are 3 kb long. Ringrose *et al.* (9) used a 500 bp window. We thus tested how CPREdictor and SVM-MOCCA models generalize when using windows that are 500 bp or 3 kb long. We found that for SVM-MOCCA, using a 3 kb sequence window gave similar generalization performance to a 500 bp window, and we focus on a 3 kb window due to it potentially capturing more diffuse PREs. For the CPREdictor, a 500 bp sequence window gives the best generalization, so we focus on using this window size (Supplementary Figure S8).

The method of Support Vector Machines supports non-linear classification, which prompted us to test SVM-MOCCA with linear, quadratic and cubic kernels (see Materials and Methods). The best generalization performance was achieved with the quadratic kernel (Supplementary Figure S9). We thus focus on the quadratic kernel in subsequent analyses, referring to the corresponding run as SVM-MOCCA.

When testing with Schwartz PREs versus dummy genomic sequences, we observed a 1.3-fold increase in PRC AUC when using SVM-MOCCA (with a quadratic kernel, trained with T2017 with three control classes, and including the GTGT motif) compared to the best CPREdictor result (trained with T2017 and including GTGT) (Figure 1A). This increase is robust over cross-validation, different PRE sets and different control classes (Figure 1A–D, and Supplementary Figures S1 and S2), and the 95% confidence intervals of the mean PRC AUCs are non-overlapping (Figure 1A and C). SVM-MOCCA is particularly good at distinguishing PREs from dummy PREs, giving a 1.5-fold increase in the mean PRC AUC over CPREdictor (Figure 1C). These results demonstrate that a more advanced modelling approach can substantially contribute to an improved generalization performance.

### Models trained on genome-wide PcG target sites predict more candidate PREs for the same expected precision

Having trained our models, we can predict candidate PREs genome-wide. Previous efforts of modelling PREs (9) have yielded candidate PRE predictions of high reliability, but with only moderate overlap with sets of genome-wide PcG target sites (67). We wanted to see whether training models on genome-wide PcG target sites would result in predictions with higher agreement with independent genome-wide PcG target sites.

We set a score threshold for each model for an expected precision of 80% genome-wide. Having trained CPREdictor with the T2017 set, we predicted over 37 times more candidate PREs genome-wide compared to having trained CPREdictor with the T2003 set (Figure 2A). Including the GTGT motif led to another 1.6-fold increase in predictions (Supplementary File 1). Using SVM-MOCCA gave a further 2-fold increase in predictions over CPREdictor (Supplementary File 3).

CPREdictor trained with T2003 predicts less than half as many PREs as the PREdictor predicted genome-wide (9). This can be explained by differences in the threshold calibration procedure. Ringrose *et al.* (9) calibrated the PREdictor threshold for one expected false positive prediction genome-wide, based on 100 genome-size sequences generated by an i.i.d. genome model. Our method differs in that we find a threshold for which we obtain a desired precision for a set of independent PREs and controls generated by a fourth-order Markov chain trained genome-wide, where the total control sequence length adds up to the size of the genome. Sequences generated by a fourth-order Markov chain are more difficult for our models to distinguish from PREs than are sequences generated by an i.i.d. model (data not shown). As a result, we can expect a reduction in numbers of predictions made using our control sequences for calibration. Also, the ability of a model to positively classify PREs is taken into account by our method, which can affect the numbers of predictions made if precision is only high for low recall, which is the case for CPREdictor trained on T2003. We can expect some further difference in numbers of predictions for these calibration methods on the basis that Ringrose *et al.* (9) use genome-length random sequences, whereas we use sets of PRE-length sequences with total set length equal to that of the genome. The calibration methods are thus not comparable. However, we use our method for calibrating all the classifiers that we consider, where possible.

### SVM-MOCCA motif model weights are heterogeneous and enriched for interacting dinucleotide patterns

Given the improved generalization of SVM-MOCCA with a quadratic kernel, we were interested in what the sequence criteria encoded in the model are. In order to investigate this, we transformed the SVM quadratic kernel into a sum of weighted feature pairs (Supplementary Text 1). Our SVM-MOCCA models are multi-class, giving a large number of weights. We wanted to condense the weights involved in distinguishing PREs from non-PREs into one weight per feature pair. We thus summed up all feature pair weights across all PRE versus non-PRE class boundaries. Duplicate features, due to reverse complements and reversed pairing order were added together, giving a set of 171 unique feature pair weights.

Strikingly, each SVM has different motif pair weighting, even though all of the SVMs have been trained on the same sets of PREs and non-PREs. The only difference lies in the motifs for which each SVM is trained to classify its local sequence landscape. This suggests that PRE sequence criteria may vary per motif, with different local sequence landscapes for different PRE motifs.

For all motifs except the En motif, all weights involving motif pairs are negatively weighted, and positively weighted feature pairs are with dinucleotide pairs. Positively weighted dinucleotides generally include ‘GA’/‘AG’, which likely correspond with GAGA site enrichment, as well as ‘AC’/‘CA’, which may correspond with GTGT sites. ‘AA’ self-pairing is generally positively weighted, as is ‘CC’ self-pairing, but interestingly, ‘AA’ paired with ‘CC’ is negatively weighted.

In conclusion, SVM-MOCCA classifier weights are enriched for patterns in agreement with previous work, such as GAGA, GTGT and poly-A, but also in ‘CC’-dinucleotide self-pairing, and there are weight interactions for the ‘AA’ and ‘CC’ dinucleotides.

### A quarter to half of genome-wide PRE predictions are in chromatin that is inaccessible early in development

ChIP-chip and ChIP-seq can only detect the PcG target regions that are accessible for binding in the cells that are being studied. We were thus interested in how many of our predictions fall in chromatin that is accessible over development. We acquired DNaseI-seq peaks for cells in five different embryonic stages (Materials and Methods). We refer to regions that overlap with peaks in at least one of the DNaseI-seq sets as being in accessible chromatin. The experimentally determined PcG target sets that we consider (34–36) were determined by ChIP-chip and ChIP-seq on ML-DmBG3-c2, ML-DmD23-c4, S2 and Sg4 cell lines, derived from embryonic cells and the developing nervous system. As expected, all regions in these sets overlap with accessible chromatin. One half to three quarters of predictions made by our methods are in accessible chromatin (Figure 2A). Therefore, a quarter to half of our predictions are inaccessible in the five developmental stages we consider, and even if they are *bonafide* PREs, they would likely go undetected in the experiments that determined the PcG targets that we consider. When comparing *in silico* PRE predictions to experimentally determined PcG targets, we thus focus on PREs in accessible chromatin.

### We predict a set of 2908 candidate PREs enriched in biologically relevant signals

To assess the degree to which our predictions recruit PcG proteins and repress or activate chromatin, we acquired genome-wide experimentally determined enrichment signals for three PcG proteins (Pc, Psc and Sfmbt) (13), histone 3 lysine 27 trimethylation (H3K27me3; a mark of Polycomb repressed chromatin) (68), and histone 3 lysine 4 monomethylation (H3K4me1; a mark of Trithorax activated chromatin) (25), from modENCODE (56) (see Materials and Methods).

Of accessible predictions, over half are enriched in H3K27me3 at some point during development, and the majority of these regions are also enriched in at least one PcG protein (Pc, Psc or Sfmbt) (Figure 2A). We extracted the latter subsets for CPREdictor T2017 w. GTGT and SVM-MOCCA (see Materials and Methods), henceforth CPREdictor T2017 w. GTGT HC (1036 high-confidence candidate PREs; Supplementary File 2) and SVM-MOCCA HC (2908 high-confidence candidate PREs; Supplementary Files 4 and 5) respectively. In addition, we extracted predictions enriched in H3K4me1 (1723 candidate TREs for CPREdictor T2017 w. GTGT, 3616 candidate TREs for SVM-MOCCA; Supplementary Files 10 and 11, respectively). The SVM-MOCCA PRE and TRE sets have 2412 candidates in common, supporting the notion of a dual function of PREs as TREs. The four sets constitute collections of candidate PRE/TREs with experimental support in the form of enrichment in biologically relevant signals.

### Models of genome-wide PcG target sites increase the agreement between PRE prediction and genome-wide experiments

For independent evaluation of our predictions, we considered two independent published sets of PcG target regions: one determined using ChIP-chip (36) and one using ChIP-seq (35). The Schwartz *et al.* (34) and Kahn *et al.* (36) sets are both based on Sg4 cells and have related sources in terms of authors and institutions. However, whereas the Schwartz *et al.* (34) set is based on peaks of E(z), Psc and Pc, the Kahn *et al.* (36) set is based on peaks of E(z), Trx, Pc and H3K27me3. The Kahn *et al.* (36) set is also larger than the Schwartz *et al.* (34) set (201 versus 170 candidate PREs, respectively, in *Drosophila* genome assembly R6; 165 in the Schwartz *et al.* set when excluding known PREs around the *invected*/*engrailed* and *vestigial* loci). As a result of their relatedness, the Kahn *et al.* (36) and Schwartz *et al.* (34) sets have a high number of overlaps ($70.65{-}83.53\%$ when considering the full sets).

The Enderle *et al.* (35) set is unrelated to the Kahn *et al.* (36) and Schwartz *et al.* (34) sets, determined using a different experimental method (ChIP-seq), cell culture (S2 cells) and factors (Pc, Ph, Psc and Trx-C). The Enderle *et al.* (35) set is an order of magnitude larger than the other sets, at 2274 regions (2265 euchromatic regions). As a result, the Enderle *et al.* (35) set covers most of the Schwartz *et al.* (34) and Kahn *et al.* (36) sets (91.18% and 89.55% of regions, respectively, when considering the full sets). Additionally, we used a set of functionally tested PREs compiled from the literature (69).

Sequence models trained on genome-wide experimentally determined PcG target sites predict a larger fraction of each of the independent experimental sets, compared to the CPREdictor trained with the T2003 set (Figure 2B). SVM-MOCCA predicts the majority of each of these sets (Figure 2B). Out of our predictions in accessible chromatin, over a quarter overlap with regions from the Schwartz, Enderle and Kahn sets (Figure 2C). Of the remainder, the majority are enriched with histone 3 lysine 4 monomethylation, potentially indicative of TREs/PREs in active states (25).

During training, we left out five PREs from the well-studied *vestigial* (*vg*) (28), *invected* (*inv*) (70) and *engrailed* (*en*) (71,72) loci. Of these PREs, CPREdictor trained with the T2003 set predicts only one, whereas CPREdictor trained with the T2017 set predicts three out of five, and SVM-MOCCA predicts all five (Figure 2D). SVM-MOCCA also predicts several other peaks, with no experimental evidence.

We were interested in the degree to which our final predictions conform to the PREs and non-PREs used for training by Ringrose *et al.* (9). We thus acquired genomic coordinates for the T2003 set by BLAST search, and compared overlaps. CPREdictor T2017 w. GTGT and SVM-MOCCA predict 45.45% and 90.91% of the T2003 PREs, respectively, which is a 1.7–3.3-fold increase over CPREdictor T2003, for which this set was used for training. Whereas CPREdictor T2003 predicts none of the T2003 non-PREs, CPREdictor T2017 w. GTGT and SVM-MOCCA predict 18.75% and 56.25%, respectively. Though SVM-MOCCA predicts many of the T2003 non-PREs, SVM-MOCCA HC Core predicts as many T2003 PREs as SVM-MOCCA, but only 18.75% of T2003 non-PREs, the same number as CPREdictor T2017. See Supplementary Figure S10 for an extended evaluation.

Taken together, these results demonstrate that models of genome-wide PcG target sites have larger agreement with independent genome-wide experimental data and functionally verified PREs than models based on the Ringrose *et al.* (9) training set.

### We predict a large new set of candidate PcG regulated genes, enriched in transcription factor and signalling functions

Given our much larger set of candidate PRE predictions, it is interesting to identify candidate target genes and their functions and to compare them with previously published sets. Target genes for our predictions were assigned as described in Materials and Methods. Target genes for other publications were extracted or defined also as described in Materials and Methods.

Similar to the prediction of PREs, our methods predict many more target genes than previously published methods (Figure 3A). The majority of predicted PcG target genes has associated PRE predictions either at the promoter or in non-coding sequence, but not both (Figure 3B). Our target gene predictions have higher numbers of overlaps with target genes from genome-wide PcG profiling studies than previously published *in silico* methods (Figure 3C). The sensitivities of our predictions to the Schwartz *et al.* (34) and Enderle *et al.* (35) sets are lower when based on genes (Figure 3C), in comparison to when based on PREs (Figure 2B).

We summarized gene set overlaps with Venn diagrams (Figure 3D). For the Schwartz *et al.* (34), Enderle *et al.* (35) and Kahn *et al.* (36) sets, respectively, 21.82%, 74.63% and 18.18% of each is unique. The majority of the Kahn *et al.* (36) set is in consensus with the other sets, whereas the majority of the Schwartz *et al.* (34) set is in agreement with the Enderle *et al.* (35) set but not the Kahn *et al.* (36) set. The largest target gene agreement is observed between the Enderle *et al.* (35) and Schwartz *et al.* (34) sets, at 319 genes, corresponding to 24.82% of the Enderle *et al.* (35) set and 76.50% of the Schwartz *et al.* (34) set. Accordingly, the sets of experimentally determined PcG target genes that we consider have different sizes and incomplete overlaps. Of published PREdictor gene predictions (9), 43.06% correspond to genes in at least one of the experimentally determined sets. The ratio of SVM-MOCCA predictions that correspond to experimentally determined PcG target genes is smaller, at 17.20%. There are only 12 validated genes that only the PREdictor predicts and SVM-MOCCA does not, and SVM-MOCCA predicts an additional 657 validated PcG target genes that the PREdictor does not. As such, SVM-MOCCA predicts many PcG target genes with experimental support, as well as a large new set of candidate PcG target genes that await experimental verification.

We analyzed PcG target gene predictions for enriched gene ontologies using GOrilla (57). Target genes predicted by SVM-MOCCA are highly enriched in transcription factor functions (Supplementary Figure S11). We compared gene ontology terms enriched in predictions made by SVM-MOCCA with terms enriched in the PREdictor, EpiPredictor (basic) and EpiPredictor (CG) predictions, the Schwartz *et al.* (34) HC Class I and II sets, and the Enderle *et al.* (35) set. The top three terms are enriched in all sets considered and are all related to transcription factor activities. The fourth term, ‘Protein binding’, is enriched for one of the experimental sets. Six terms are enriched in zero or one other set and comprise functions unrelated to transcription factor activities: ‘Calcium ion binding’, ‘Potassium ion transmembrane transporter activity’, ‘Cytoskeletal protein binding’, ‘Actin binding’, ‘Cell adhesion molecule binding’ and ‘Protein kinase activity’. The remaining enriched terms correspond to transcription factor and signalling activities (see Supplementary File 9 for complete lists of enriched terms in all sets).

## DISCUSSION

Previous approaches to modelling *Drosophila* PREs have used comparatively small sets of functionally characterized PREs and non-PREs for training binary classifiers (9,31,32). Here, we trained models on published genome-wide sets of PcG-recruiting chromatin regions. Negatives were generated by fourth-order Markov chains trained either on the same set of PcG-recruiting sequences or the entire genome and also taken from coding sequence.

Genome-wide sets of experimentally determined PcG-recruiting regions can be expected to contain false positives, due both to physical chromatin interactions and to experimental conditions. PREs have been observed to make long-range chromatin contacts with promoters, with ChIP signals at both contact points, where then one signal may be only a shadow of the interaction (1,73). A recent Hi-C study by Eagen *et al.* (74) found PRC1 enriched at 26% of chromatin loop anchors, and for loops where not both anchors correspond to PREs, there could thus be additional shadow signals. Furthermore, the majority of PRE ChIP studies rely on cell cultures, and even if assuming optimal experimental conditions and choice of antibodies, cultured cells are not normal cells (75), and genome-wide epigenetic states are likely to differ from those *in vivo*. Furthermore, ChIP only captures protein binding at a certain time in a certain population of cells, and results are thus unlikely to reflect the epigenetic diversity in the entire animal. Additionally, the PcG-recruiting regions we consider are large (3 kb after expansion to account for potential distancing between recruiting sequences and recruited factors). Nonetheless, models trained on PcG-recruiting regions and automatically generated controls generalize well to independent PcG-recruiting regions over cross-validation, with substantially higher PRC AUC than the CPREdictor trained on the set used by Ringrose *et al.* (9) (2.88-fold increase). Thus, our modelling methods are robust against any non-PRE signals that the ChIP-data used for training may contain, and they manage to pick out general features predictive of PcG-recruiting sequences.

Identifying a large, definitive set of genomic non-PREs that is sufficiently PRE-like to use for training sequence models is challenging. We circumvented this problem by automatically generating non-PRE sequences by use of naive PRE models (fourth-order Markov chains), making use of the knowledge that motif pair occurrences are predictive of PREs, while individual motif occurrences are only marginally predictive (9). Thus, the probability of these models generating *bona fide* PREs can be expected to be low, but the sequences they generate have highly similar motif composition to that of PREs. Despite this similarity, our models are able to distinguish them from published PcG target regions, showing that these genome-wide experimentally determined regions are enriched in motif co-occurrence patterns.

We developed a new method for modelling *cis*-regulatory elements, called SVM-MOCCA. SVM-MOCCA distinguishes itself from other PRE-modelling methods by modelling the local motif and dinucleotide occurrence landscape around motif occurrences. Across the board, SVM-MOCCA gave the best generalization to independent PcG-recruiting regions over cross-validation.

The models we trained on genome-wide experimental data and randomly generated controls predict many more PREs genome-wide than previous methods, for the same expected precision of 80%. This is accompanied by our methods predicting a much larger number of experimentally determined PcG target regions than previous methods. We excluded five well-studied PREs at the *vestigial*, *engrailed* and *invected* loci from our training data, both during model testing and for genome-wide prediction, and we predict the majority of these PREs. Our computational approach allowed us to study the importance of the GTGT motif and of other motifs in a genome-wide manner. Adding the GTGT motif results both in increased model generalization and in a higher number of predictions genome-wide, adding to the growing body of evidence that this motif plays an important role in Polycomb recruitment. The inclusion of other published motifs had only little impact on model generalization.

Counterintuitively, models trained using our methods predict more of the PREs used for training by Ringrose *et al.* (9) than does the CPREdictor trained on that very set, for an expected precision of $80\%$ genome-wide (Supplementary Figure S10). A possible explanation for this is that our models have been trained on large sets of non-PRE sequences, and that this makes the models better at distinguishing PcG target sites from genomic background. Models trained with the T2017 set also predict a minimal number of sequences from the non-PRE set used by Ringrose *et al.* (9). SVM-MOCCA predicts over half of the non-PREs used by Ringrose *et al.* (9), but filtering by biological signals and predicting the core predictive regions of the SVM-MOCCA predictions lowers the number of non-PREs predicted to a fifth.

Despite the much larger number of predictions that our models make, and though we predict a large fraction of the PREs in the experimental sets that we consider, none of our sets of predictions completely cover any of the experimentally determined PRE sets. There may be several reasons for this. Our models may lack the sequence features needed in order to accurately model the remaining PREs, such as additional motifs, higher-order motif occurrence combinatorics, strandedness and positioning, or taking local or distal sequence elements into consideration. The experimental sets may also contain regions that are not in fact PREs, but are instead marked by PcG proteins due to physical interactions with PREs, or are enriched due to experimental noise.

As the SVM-MOCCA predictions are 3 kb long, we predicted core PRE fragments. It is interesting to note that the core fragments have fewer overlaps with experimental sets. This means that PcG-enriched regions are close by, and it is possible that experimental signals in some cases have been displaced due to factor mobility. Our observation is also in agreement with the suggestion of Schuettengruber *et al.* (30) that the genome uses ‘not only local sequence (high-affinity transcription factor binding sites located at the binding peaks) information to determine PREs, but also integration of regional sequence information [...]’ and that the use of such information to predict PREs ‘may break the current specificity and sensitivity barriers.’ A corollary to this latter notion is the possibility that previous evaluations of PRE prediction have taken regional information (recruitment versus enrichment) into account only insufficiently.

Multiple weaker PREs functioning together has been observed for the *engrailed* gene locus (76). Our core PRE prediction method only finds the sub-region with the strongest sequence signal enrichment. It may be that some SVM-MOCCA predictions are enriched in multiple weak sequence signals that add up to a significant prediction. If so, ChIP-signals that do not overlap with a predicted core may instead coincide with a separate, weaker PRE sequence signal. It could also be that the position of the final ChIP-peak depends on the structure of the complex of weak PREs and PcG proteins.

We present two high-confidence sets of *D. melanogaster* candidate PRE predictions, based on filtering predictions for enrichment of histone 3 lysine 27 trimethylation and at least one of three PcG proteins (Pc, Psc or Sfmbt). This filtering procedure provides a form of experimental validation of predicted PRE candidates on the basis of previously published ChIP enrichment datasets and is comparable to experimental definitions of PREs from such datasets (34–36). However, our procedure does not define PRE candidates from ChIP enrichment datasets alone, but starts with a set of candidates that were predicted by a well-designed machine-learning model and that share sequence characteristics that have been established to be relevant, both here and in previous work (9,29,30). Furthermore, since with our filtering procedure we treat any type of ChIP enrichment as a necessary but not as a sufficient criterion for PRE-ness, our high-confidence candidates are less prone to potential looping, spreading and displacement artefacts. In fact, one could argue that the presence of a PRE prediction in a region of ChIP enrichment gives credence to that enrichment and indicates the initial Polycomb recruitment site. Even though the high-confidence prediction sets are smaller than the complete prediction sets (1036 versus 3521 predictions for CPREdictor and 2908 versus 6911 for SVM-MOCCA), they have almost as high numbers of overlaps with the experimental sets that we consider (Supplementary Figure S10). As such, we increase precision to the experimentally determined PcG target region sets with low loss of recall. It is worth noting that we used merged ChIP peaks from multiple experiments per factor and that the factors we considered are not only enriched at PREs, making this a modest filtering step. Both high-confidence PRE sets are larger than the Schwartz *et al.* (34) set that the models were trained on, despite the filtering for biologically relevant chromatin signatures. These high-confidence candidate PREs remain to be tested for whether they can maintain target gene transcription states.

Additionally, we predict many PREs outside of the high-confidence sets. A large number of candidate PREs do not overlap with chromatin that is accessible in the developmental stages that we consider. Inaccessible PRE predictions may be functional PREs that recruit PcG/TrxG when chromatin is made accessible. A large number of PRE predictions that do not overlap with experimentally determined PRE sets but are nonetheless in accessible chromatin are enriched for histone 3 lysine 4 monomethylation (H3K4me1). It is possible that these predictions are PRE/TREs in an activated state (25) and that they recruit Polycomb in other contexts. A large proportion (over 82%) of high-confidence PRE candidates are also enriched in H3K4me1, supporting the notion of a dual function of PREs as TREs. Furthermore, the fact that all candidates were predicted by a single machine learning model suggests that PREs and TREs have a common sequence code. The remaining predictions may be false positives, due both to a threshold calibration for an expected precision of 80% (corresponding to an expected 20% of false positives among the positive predictions) and to imperfections in our training sets and models.

An extended overlap analysis (Supplementary Table S2) showed only small differences in high-confidence PRE candidate enrichment between H3K4me1 and H3K4me3, the latter of which has previously been reported to be methylated by TRX but was later shown to be mostly methylated by SET1/COMPASS (reviewed in (77)).

In correspondence with our larger numbers of *D. melanogaster* PRE predictions compared to previously published *in silico* methods, we predict a larger set of candidate PcG/TrxG target genes, with higher numbers of overlaps with published experimentally determined PcG/TrxG target genes. We speculate that, like our predicted PREs themselves, predicted targets that have not previously been identified on the basis of ChIP enrichment, might recruit Polycomb or Trithorax group proteins and associated histone modifications in cell types or in conditions that so far have not been studied with respect to their epigenetic regulatory landscape. Our target gene predictions are highly enriched for transcription factor functions and also for novel potential PcG target gene functions. The sensitivities of predictions to experimentally determined sets are lower when considering PcG target genes than for candidate PREs. This can be attributed to different methods being employed for predicting target genes from regions, as well as different genome annotations used while predicting target genes. Schwartz *et al.* (34) used the Dm2 assembly and Enderle *et al.* (35) used Dm3. Both Schwartz *et al.* (34) and Enderle *et al.* (35) determined PcG target genes based on enrichment of PcG signals proximal to the TSS, rather than based on gene proximity to candidate PREs. Overall, our genome-wide PcG target gene predictions are more sensitive to experimentally determined PcG target genes than are published predictions from previous *in silico* PcG target gene prediction methods.

Although we devoted most of our attention to training with the Schwartz *et al.* (34) candidate PREs, we obtain similar results when training with the Enderle *et al.* (35) and Kahn *et al.* (36) sets (Supplementary File 8), demonstrating that our results are general. Training SVM-MOCCA with the Schwartz *et al.* (34) candidate PREs resulted in 6911 predictions genome-wide, training with the Enderle *et al.* (35) set resulted in 5910 predictions genome-wide, and 5294 of the Schwartz *et al.* (34)–based predictions overlap with Enderle *et al.* (35)–based predictions (CPREdictor results are similar, at lower total numbers of predictions, 3521, 2775 and 2768, respectively). This high overlap indicates the robustness of our approach and might also suggest a potential saturation of PRE prediction.

There are multiple ways in which our work can be expanded upon. The majority of the steps have been written as a computational pipeline, aiding not only the reproducibility of our results, but also the application to other problems. Our methods can be adapted to the modelling of other classes of regulatory sequences and for use in other genomes, given appropriate sets of motifs and genome-wide experimental data. Our high-confidence PRE predictions are a rich source of candidates for the further study of PRE function, architecture and dynamic behaviour during development.

## Supplementary Material

gkz617_Supplemental_Files
