# Supplementary material for: DNA sequence models of genome-wide Drosophila melanogaster Polycomb binding sites improve generalization to independent Polycomb Response Elements
Source: Nucleic Acids Res. 2019 Jul 24;47(15):7781–97. doi: 10.1093/nar/gkz617 (PMC6735708; doi:10.1093/nar/gkz617)
Supplement: gkz617_Supplemental_Files [file gkz617_supplemental_files.zip › SFile8_AnalysisProfile.pdf]

Benchmark profile

Bjørn André Bredesen, December 23, 2018

Contents

1 Varied training set sizes (training set PREs: Schwartz 2010 PREs) 4

1.1 Varied training set sizes - PRE training set: Schwartz 2010 PREs - Validation set: validation_PREsSchwartz vs. validation_R5mers . . . 4

1.2 Varied training set sizes - PRE training set: Schwartz 2010 PREs - Validation set: validation_PREsSchwartz vs. validation_CDS . . . 6

1.3 Varied training set sizes - PRE training set: Schwartz 2010 PREs - Validation set: validation_PREsSchwartz vs. validation_D5merPREsSchwartz . . . 8

2 Exclusion/inclusion of GTGT (training set PREs: Schwartz 2010 PREs) 10

2.1 Exclusion/inclusion of GTGT - PRE training set: Schwartz 2010 PREs - Validation set: validation_PREsSchwartz vs. validation_R5mers . . . 11

2.2 Exclusion/inclusion of GTGT - PRE training set: Schwartz 2010 PREs - Validation set: validation_PREsSchwartz vs. validation_CDS . . . 13

2.3 Exclusion/inclusion of GTGT - PRE training set: Schwartz 2010 PREs - Validation set: validation_PREsSchwartz vs. validation_D5merPREsSchwartz . . . 15

3 GTGT versus random 4-mers (training set PREs: Schwartz 2010 PREs) 17

3.1 GTGT versus random 4-mers - PRE training set: Schwartz 2010 PREs - Validation set: validation_PREsSchwartz vs. validation_R5mers . . . 18

3.2 GTGT versus random 4-mers - PRE training set: Schwartz 2010 PREs - Validation set: validation_PREsSchwartz vs. validation_CDS . . . 19

3.3 GTGT versus random 4-mers - PRE training set: Schwartz 2010 PREs - Validation set: validation_PREsSchwartz vs. validation_D5merPREsSchwartz . . . 20

4 Tests of extra published motifs (training set PREs Schwartz 2010 PREs) 21

4.1 Tests of extra published motifs - PRE training set: Schwartz 2010 PREs - Validation set: validation_PREsSchwartz vs. validation_R5mers . . . 22

4.2 Tests of extra published motifs - PRE training set: Schwartz 2010 PREs - Validation set: validation_PREsSchwartz vs. validation_CDS . . . 23

4.3 Tests of extra published motifs - PRE training set: Schwartz 2010 PREs - Validation set: validation_PREsSchwartz vs. validation_D5merPREsSchwartz . . . 24

5 Classifier window size (training set PREs: Schwartz 2010 PREs) 25

5.1 Classifier window size - PRE training set: Schwartz 2010 PREs - Validation set: validation_PREsSchwartz vs. validation_R5mers . . . 25

5.2 Classifier window size - PRE training set: Schwartz 2010 PREs - Validation set: validation_PREsSchwartz vs. validation_CDS . . . 27

5.3 Classifier window size - PRE training set: Schwartz 2010 PREs - Validation set: validation_PREsSchwartz vs. validation_D5merPREsSchwartz . . . 29

6 SVM-MOCCA kernel functions (training set PREs: Schwartz 2010 PREs) - 3kb window 31

6.1 SVM-MOCCA kernel functions - PRE training set: Schwartz 2010 PREs - Validation set: validation_PREsSchwartz vs. validation_R5mers . . . 32

6.2 SVM-MOCCA kernel functions - PRE training set: Schwartz 2010 PREs - Validation set: validation_PREsSchwartz vs. validation_CDS . . . 33

6.3 SVM-MOCCA kernel functions - PRE training set: Schwartz 2010 PREs - Validation set: validation_PREsSchwartz vs. validation_D5merPREsSchwartz . . . 34

7 CPREdictor trained with T2003 PREs versus non-PREs or dummy PREs 35

7.1 Classifier comparison - T2003, Markov chain controls - Validation set: validation PREsSchwartz vs. validation R5mers . . . 36

7.2 Classifier comparison - T2003, Markov chain controls - Validation set: validation PREsSchwartz vs. validation CDS . . . 38

7.3 Classifier comparison - T2003, Markov chain controls - Validation set: validation PREsSchwartz vs. validation D5merPREsSchwartz . . . 40

8 T2003 PREs versus non-PREs (training set PREs: Schwartz 2010 PREs) 42
8.1 T2003 tests - PRE training set: Schwartz 2010 PREs - Validation set: T2003 PREs vs. T2003 NonPREs . . . 43
8.2 T2003 tests - PRE training set: Schwartz 2010 PREs - Validation set: T2003 PREs vs. T2003 D5merPREs . . . 45

9 Classifier comparison, coloured (training set PREs: Schwartz 2010 PREs) 47
9.1 Classifier comparison, coloured - training set PREs: Schwartz 2010 PREs - Validation set: validation_PREsSchwartz vs. validation_R5mers . . . 48
9.2 Classifier comparison, coloured - training set PREs: Schwartz 2010 PREs - Validation set: validation_PREsSchwartz vs. validation_CDS . . . 50
9.3 Classifier comparison, coloured - training set PREs: Schwartz 2010 PREs - Validation set: validation_PREsSchwartz vs. validation_D5merPREsSchwartz . . . 52

10 Training set PREs: Schwartz 2010 PREs 54
10.1 Classifier comparison (training set PREs: Schwartz 2010 PREs) . . . 54
10.2 Classifier comparison - training set PREs: Schwartz 2010 PREs - Validation set: validation_PREsSchwartz vs. validation_R5mers . . . 55
10.3 Classifier comparison - training set PREs: Schwartz 2010 PREs - Validation set: validation_PREsSchwartz vs. validation_CDS . . . 57
10.4 Classifier comparison - training set PREs: Schwartz 2010 PREs - Validation set: validation_PREsSchwartz vs. validation_D5merPREsSchwartz . . . 59
10.5 Numbers of candidate PREs (training set PREs: Schwartz 2010 PREs) . . . 61
10.6 Repressed PRE Polycomb recruitment (training set PREs: Schwartz 2010 PREs) . . . 61
10.7 Repressed PRE Polycomb recruitment (training set PREs: Schwartz 2010 PREs) . . . 62
10.8 PRE set overlap sensitivity (training set PREs: Schwartz 2010 PREs) . . . 63
10.9 PRE set overlap sensitivity (training set PREs: Schwartz 2010 PREs) . . . 64
10.10 PcG/TrxG target genes (training set PREs: Schwartz 2010 PREs) . . . 65
10.11 PcG/TrxG target gene sensitivity (training set PREs: Schwartz 2010 PREs) . . . 65
10.12 PcG/TrxG target gene precision (training set PREs: Schwartz 2010 PREs) . . . 66
10.13 PcG/TrxG target gene P-values (training set PREs: Schwartz 2010 PREs) . . . 66
10.14 Genomic loci of PREs per predicted target gene (training set PREs: Schwartz 2010 PREs) . . . 67
10.15 Gene Venn diagrams (training set PREs: Schwartz 2010 PREs) . . . 67

11 Training set PREs: Kahn 2014 PREs 68
11.1 Classifier comparison (training set PREs: Kahn 2014 PREs) . . . 68
11.2 Classifier comparison - training set PREs: Kahn 2014 PREs - Validation set: validation_PREsKahn vs. validation_R5mers . . . 69
11.3 Classifier comparison - training set PREs: Kahn 2014 PREs - Validation set: validation_PREsKahn vs. validation_CDS . . . 71
11.4 Classifier comparison - training set PREs: Kahn 2014 PREs - Validation set: validation_PREsKahn vs. validation_D5merPREsKahn . . . 73
11.5 Numbers of candidate PREs (training set PREs: Kahn 2014 PREs) . . . 75
11.6 Repressed PRE Polycomb recruitment (training set PREs: Kahn 2014 PREs) . . . 75
11.7 PRE set overlap sensitivity (training set PREs: Kahn 2014 PREs) . . . 76
11.8 PRE set overlap sensitivity (training set PREs: Kahn 2014 PREs) . . . 77
11.9 PcG/TrxG target genes (training set PREs: Kahn 2014 PREs) . . . 78
11.10 PcG/TrxG target gene sensitivity (training set PREs: Kahn 2014 PREs) . . . 78
11.11 PcG/TrxG target gene precision (training set PREs: Kahn 2014 PREs) . . . 79
11.12 PcG/TrxG target gene P-values (training set PREs: Kahn 2014 PREs) . . . 79
11.13 Genomic loci of PREs per predicted target gene (training set PREs: Kahn 2014 PREs) . . . 80

12 Training set PREs: Enderle 2011 PREs 81
12.1 Classifier comparison (training set PREs: Enderle 2011 PREs) . . . 81
12.2 Classifier comparison - training set PREs: Enderle 2011 PREs - Validation set: validation_PREsEnderle vs. validation_R5mers . . . 82
12.3 Classifier comparison - training set PREs: Enderle 2011 PREs - Validation set: validation_PREsEnderle vs. validation_CDS . . . 84
12.4 Classifier comparison - training set PREs: Enderle 2011 PREs - Validation set: validation_PREsEnderle vs. validation_D5merPREsEnderle . . . 86
12.5 Numbers of candidate PREs (training set PREs: Enderle 2011 PREs) . . . 88

12.6 Repressed PRE Polycomb recruitment (training set PREs: Enderle 2011 PREs) 88
12.7 PRE set overlap sensitivity (training set PREs: Enderle 2011 PREs) 89
12.8 PRE set overlap sensitivity (training set PREs: Enderle 2011 PREs) 90
12.9 PcG/TrxG target genes (training set PREs: Enderle 2011 PREs) 91
12.10 PcG/TrxG target gene sensitivity (training set PREs: Enderle 2011 PREs) 91
12.11 PcG/TrxG target gene precision (training set PREs: Enderle 2011 PREs) 92
12.12 PcG/TrxG target gene P-values (training set PREs: Enderle 2011 PREs) 92
12.13 Genomic loci of PREs per predicted target gene (training set PREs: Enderle 2011 PREs) . . . 93

Varied training set sizes (training set PREs: Schwartz 2010 PREs)

- - - - T2003 (12 PREs)
—— T2017 x12
▬ T2017 x50
━━ T2017 x110

Classifier	Path
T2003 (12 PREs)	CPREdictor_M2003_T2003_mdBetween_wmPREdictor
T2017 x12	CPREdictor_M2003_CPPREsSchwartz_CND5merPREsSchwartz_T12_mdBetween_wmPREdictor
T2017 x50	CPREdictor_M2003_CPPREsSchwartz_CND5merPREsSchwartz_T50_mdBetween_wmPREdictor
T2017 x110	CPREdictor_M2003_CPPREsSchwartz_CND5merPREsSchwartz_T110_mdBetween_wmPREdictor

Varied training set sizes - PRE training set: Schwartz 2010 PREs - Validation set: validation_PREsSchwartz vs. validation_R5mers

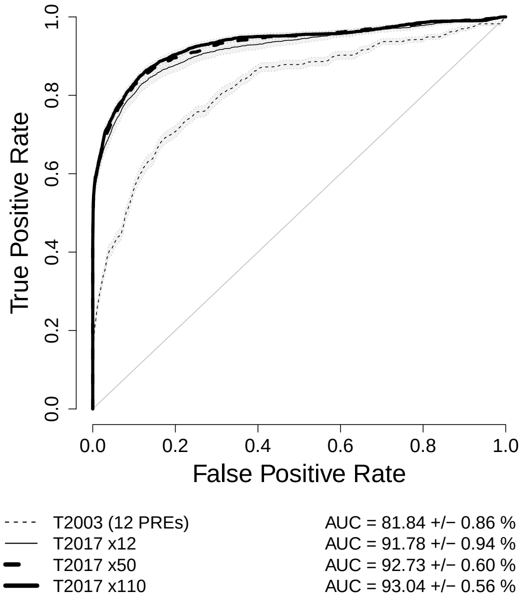

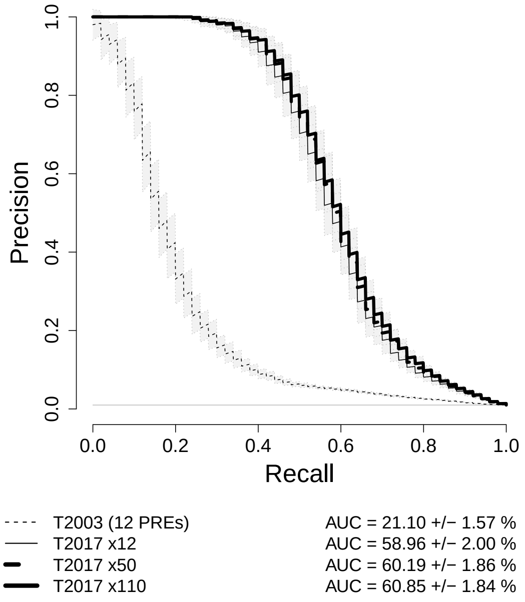

Classifier	ROC AUC
T2003 (12 PREs)	81.84 +/- 0.86 %
T2017 x12	91.78 +/- 0.94 %
T2017 x50	92.73 +/- 0.60 %
T2017 x110	93.04 +/- 0.56 %

Classifier	PRC AUC
T2003 (12 PREs)	21.10 +/- 1.57 %
T2017 x12	58.96 +/- 2.00 %
T2017 x50	60.19 +/- 1.86 %
T2017 x110	60.85 +/- 1.84 %

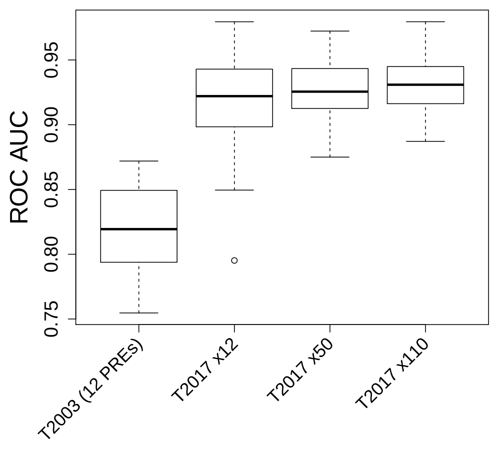

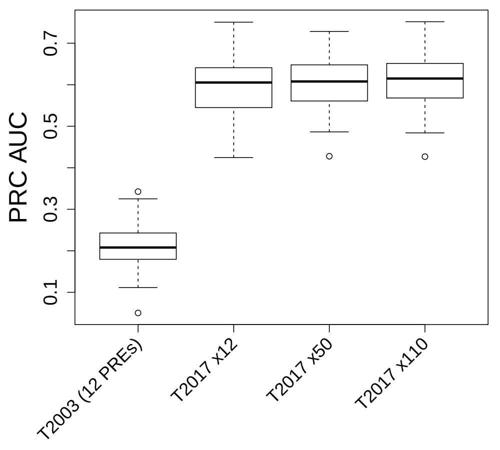

Classifier 1	Classifier 2	$p(H_0 : ROC1 <= ROC2)$	t	$p(H_0 : PRC1 <= PRC2)$	t
T2003 (12 PREs)	T2003 (12 PREs)	-	-	-	-
T2003 (12 PREs)	T2017 x12	1.000000E+00	-1.813830E+01	1.000000E+00	-4.370766E+01
T2003 (12 PREs)	T2017 x50	1.000000E+00	-2.550434E+01	1.000000E+00	-5.187992E+01
T2003 (12 PREs)	T2017 x110	1.000000E+00	-2.669258E+01	1.000000E+00	-5.230428E+01
T2017 x12	T2003 (12 PREs)	1.106109E-23	1.813830E+01	3.250031E-41	4.370766E+01
T2017 x12	T2017 x12	-	-	-	-
T2017 x12	T2017 x50	9.981789E-01	-3.054321E+00	9.979151E-01	-3.005686E+00
T2017 x12	T2017 x110	9.998831E-01	-3.971256E+00	9.999849E-01	-4.597455E+00
T2017 x50	T2003 (12 PREs)	3.031562E-30	2.550434E+01	8.714279E-45	5.187992E+01
T2017 x50	T2017 x12	1.821061E-03	3.054321E+00	2.084871E-03	3.005686E+00
T2017 x50	T2017 x50	-	-	-	-
T2017 x50	T2017 x110	9.999626E-01	-4.324484E+00	9.999917E-01	-4.775582E+00
T2017 x110	T2003 (12 PREs)	3.770779E-31	2.669258E+01	5.886922E-45	5.230428E+01
T2017 x110	T2017 x12	1.169411E-04	3.971256E+00	1.514482E-05	4.597455E+00
T2017 x110	T2017 x50	3.741929E-05	4.324484E+00	8.312924E-06	4.775582E+00
T2017 x110	T2017 x110	-	-	-	-

Varied training set sizes - PRE training set: Schwartz 2010 PREs - Validation set: validation_PREsSchwartz vs. validation_CDS

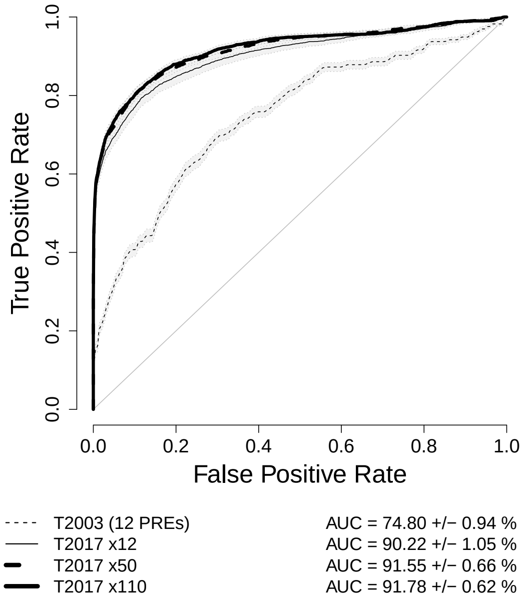

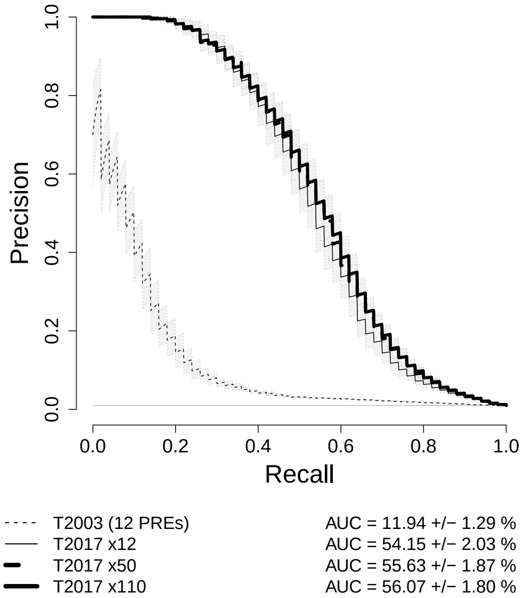

Classifier	ROC AUC
T2003 (12 PREs)	74.80 +/- 0.94 %
T2017 x12	90.22 +/- 1.05 %
T2017 x50	91.55 +/- 0.66 %
T2017 x110	91.78 +/- 0.62 %

Classifier	PRC AUC
T2003 (12 PREs)	11.94 +/- 1.29 %
T2017 x12	54.15 +/- 2.03 %
T2017 x50	55.63 +/- 1.87 %
T2017 x110	56.07 +/- 1.80 %

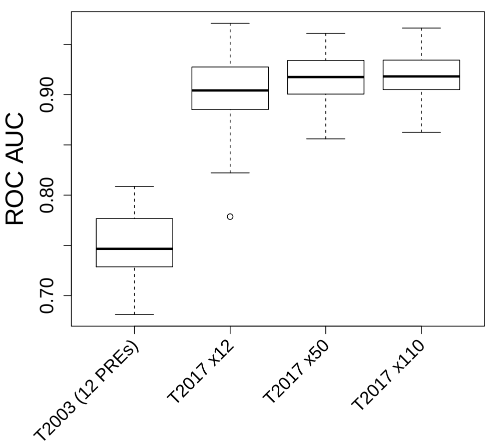

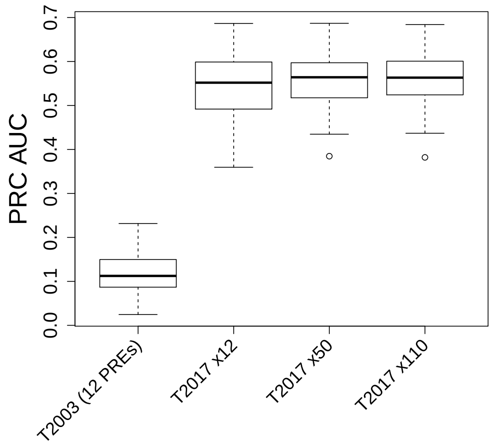

Classifier 1	Classifier 2	$p(H_0 : ROC1 <= ROC2)$	t	$p(H_0 : PRC1 <= PRC2)$	t
T2003 (12 PREs)	T2003 (12 PREs)	-	-	-	-
T2003 (12 PREs)	T2017 x12	1.000000E+00	-2.480272E+01	1.000000E+00	-4.444688E+01
T2003 (12 PREs)	T2017 x50	1.000000E+00	-3.616181E+01	1.000000E+00	-5.570959E+01
T2003 (12 PREs)	T2017 x110	1.000000E+00	-3.732274E+01	1.000000E+00	-5.922155E+01
T2017 x12	T2003 (12 PREs)	1.080062E-29	2.480272E+01	1.457446E-41	4.444688E+01
T2017 x12	T2017 x12	-	-	-	-
T2017 x12	T2017 x50	9.992314E-01	-3.355395E+00	9.979414E-01	-3.010256E+00
T2017 x12	T2017 x110	9.998162E-01	-3.827470E+00	9.998225E-01	-3.838754E+00
T2017 x50	T2003 (12 PREs)	2.663183E-37	3.616181E+01	2.814634E-46	5.570959E+01
T2017 x50	T2017 x12	7.686397E-04	3.355395E+00	2.058638E-03	3.010256E+00
T2017 x50	T2017 x50	-	-	-	-
T2017 x50	T2017 x110	9.751495E-01	-2.012310E+00	9.832607E-01	-2.187864E+00
T2017 x110	T2003 (12 PREs)	5.971102E-38	3.732274E+01	1.469455E-47	5.922155E+01
T2017 x110	T2017 x12	1.838332E-04	3.827470E+00	1.774678E-04	3.838754E+00
T2017 x110	T2017 x50	2.485050E-02	2.012310E+00	1.673931E-02	2.187864E+00
T2017 x110	T2017 x110	-	-	-	-

Varied training set sizes - PRE training set: Schwartz 2010 PREs - Validation set: validation_PREsSchwartz vs. validation_D5merPREsSchwartz

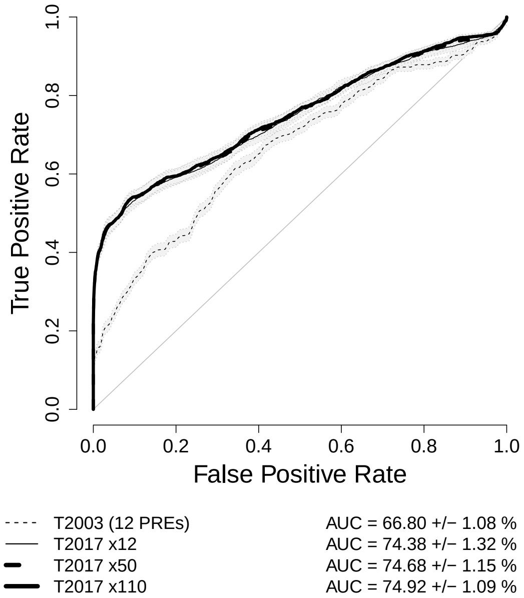

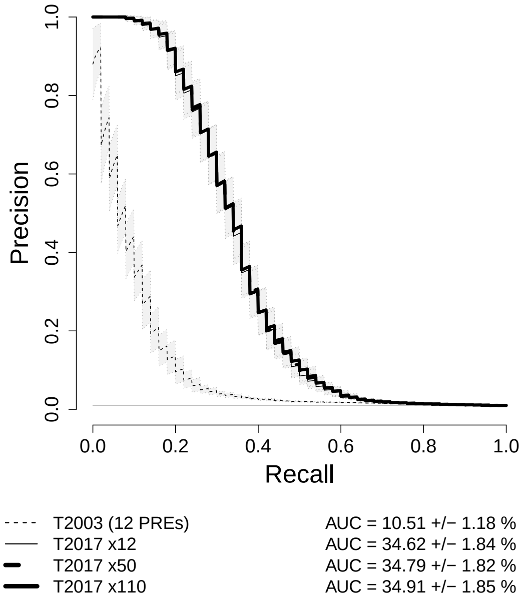

Classifier	ROC AUC
T2003 (12 PREs)	66.80 +/- 1.08 %
T2017 x12	74.38 +/- 1.32 %
T2017 x50	74.68 +/- 1.15 %
T2017 x110	74.92 +/- 1.09 %

Classifier	PRC AUC
T2003 (12 PREs)	10.51 +/- 1.18 %
T2017 x12	34.62 +/- 1.84 %
T2017 x50	34.79 +/- 1.82 %
T2017 x110	34.91 +/- 1.85 %

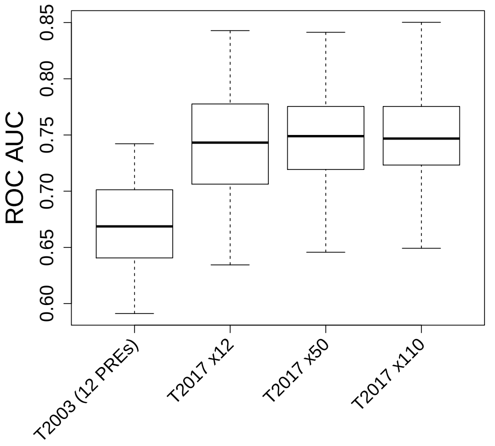

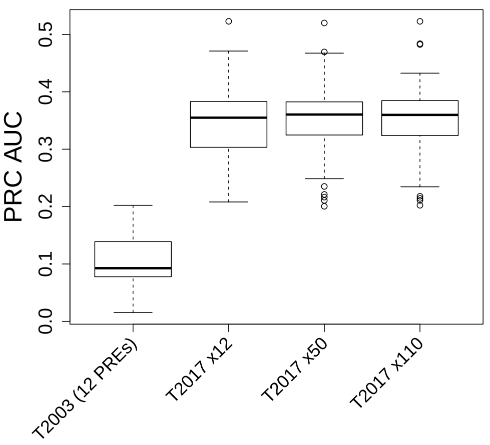

Classifier 1	Classifier 2	$p(H_0 : ROC1 <= ROC2)$	t	$p(H_0 : PRC1 <= PRC2)$	t
T2003 (12 PREs)	T2003 (12 PREs)	-	-	-	-
T2003 (12 PREs)	T2017 x12	1.000000E+00	-1.071171E+01	1.000000E+00	-2.847630E+01
T2003 (12 PREs)	T2017 x50	1.000000E+00	-1.286616E+01	1.000000E+00	-2.990521E+01
T2003 (12 PREs)	T2017 x110	1.000000E+00	-1.357770E+01	1.000000E+00	-2.935681E+01
T2017 x12	T2003 (12 PREs)	9.777145E-15	1.071171E+01	1.912756E-32	2.847630E+01
T2017 x12	T2017 x12	-	-	-	-
T2017 x12	T2017 x50	8.474387E-01	-1.036358E+00	8.666740E-01	-1.123613E+00
T2017 x12	T2017 x110	9.547615E-01	-1.726947E+00	9.620578E-01	-1.813481E+00
T2017 x50	T2003 (12 PREs)	1.245543E-17	1.286616E+01	1.974037E-33	2.990521E+01
T2017 x50	T2017 x12	1.525613E-01	1.036358E+00	1.333260E-01	1.123613E+00
T2017 x50	T2017 x50	-	-	-	-
T2017 x50	T2017 x110	9.622423E-01	-1.815844E+00	9.403356E-01	-1.585312E+00
T2017 x110	T2003 (12 PREs)	1.570290E-18	1.357770E+01	4.664484E-33	2.935681E+01
T2017 x110	T2017 x12	4.523852E-02	1.726947E+00	3.794223E-02	1.813481E+00
T2017 x110	T2017 x50	3.775768E-02	1.815844E+00	5.966435E-02	1.585312E+00
T2017 x110	T2017 x110	-	-	-	-

Exclusion/inclusion of GTGT (training set PREs: Schwartz 2010 PREs)

CPREdictor T2003
CPREdictor T2003 w. GTGT
CPREdictor T2017
CPREdictor T2017 w. GTGT
Dummy PREdictor w. GTGT

Classifier	Path
CPREdictor T2003	CPREdictor_M2003_T2003_mdBetween_wmPREdictor
CPREdictor T2003 w. GTGT	CPREdictor_M2003_GTGT_T2003_mdBetween_wmPREdictor
CPREdictor T2017	CPREdictor_M2003_CPPREsSchwartz_CND5merPREsSchwartz_T110_mdBetween_wmPREdictor
CPREdictor T2017 w. GTGT	CPREdictor_M2003_GTGT_CPPREsSchwartz_CND5merPREsSchwartz_T110_mdBetween_wmPREdictor
Dummy PREdictor w. GTGT	DummyPREdictor_M2003_GTGT_T2003_mdBetween_wmPREdictor

Exclusion/inclusion of GTGT - PRE training set: Schwartz 2010 PREs - Validation set: validation_PREsSchwartz vs. validation_R5mers

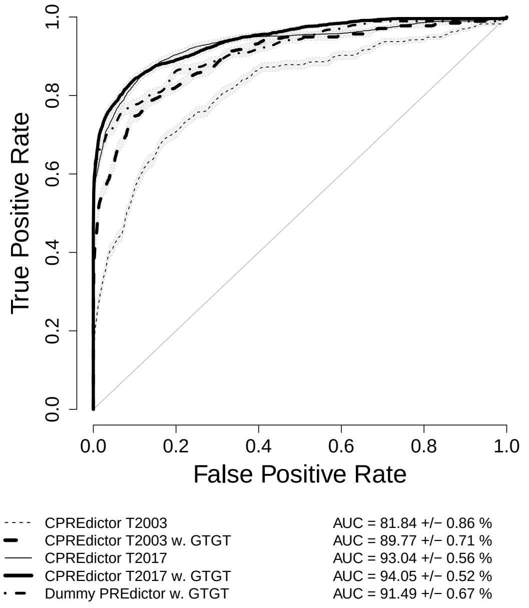

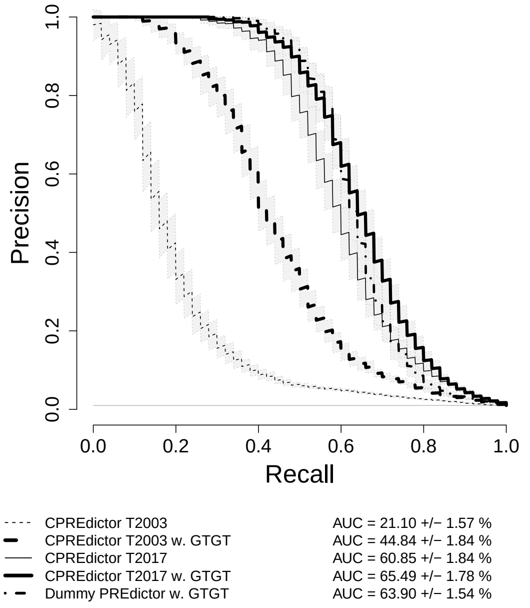

Classifier	ROC AUC
CPREdictor T2003	81.84 +/- 0.86 %
CPREdictor T2003 w. GTGT	89.77 +/- 0.71 %
CPREdictor T2017	93.04 +/- 0.56 %
CPREdictor T2017 w. GTGT	94.05 +/- 0.52 %
Dummy PREdictor w. GTGT	91.49 +/- 0.67 %

Classifier	PRC AUC
CPREdictor T2003	21.10 +/- 1.57 %
CPREdictor T2003 w. GTGT	44.84 +/- 1.84 %
CPREdictor T2017	60.85 +/- 1.84 %
CPREdictor T2017 w. GTGT	65.49 +/- 1.78 %
Dummy PREdictor w. GTGT	63.90 +/- 1.54 %

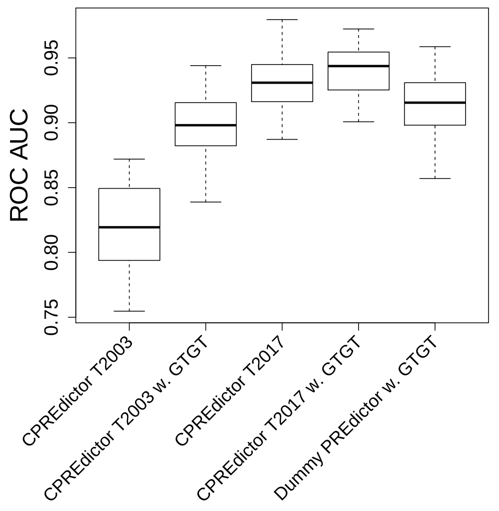

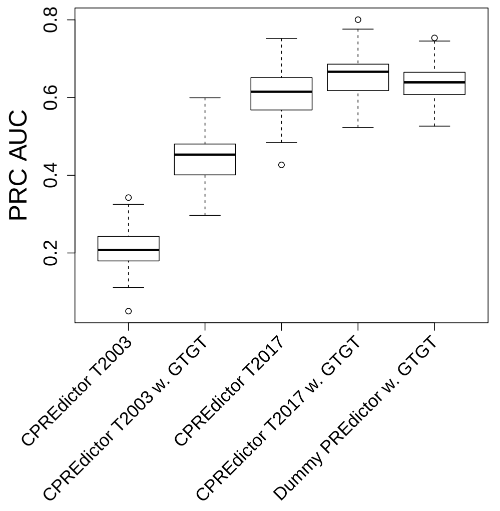

Classifier 1	Classifier 2	$p(H_0 : ROC1 <= ROC2)$	t	$p(H_0 : PRC1 <= PRC2)$	
CPREdictor T2003	CPREdictor T2003	-	-	-	
CPREdictor T2003	CPREdictor T2003 w. GTGT	1.000000E+00	-2.372866E+01	1.000000E+00	-3[illegible]
CPREdictor T2003	CPREdictor T2017	1.000000E+00	-2.669258E+01	1.000000E+00	-5[illegible]
CPREdictor T2003	CPREdictor T2017 w. GTGT	1.000000E+00	-2.760384E+01	1.000000E+00	-5[illegible]
CPREdictor T2003	Dummy PREdictor w. GTGT	1.000000E+00	-2.429137E+01	1.000000E+00	-5[illegible]
CPREdictor T2003 w. GTGT	CPREdictor T2003	8.026321E-29	2.372866E+01	1.358707E-36	3[illegible]
CPREdictor T2003 w. GTGT	CPREdictor T2003 w. GTGT	-	-	-	
CPREdictor T2003 w. GTGT	CPREdictor T2017	1.000000E+00	-9.082298E+00	1.000000E+00	-2[illegible]
CPREdictor T2003 w. GTGT	CPREdictor T2017 w. GTGT	1.000000E+00	-1.671251E+01	1.000000E+00	-2[illegible]
CPREdictor T2003 w. GTGT	Dummy PREdictor w. GTGT	1.000000E+00	-1.039286E+01	1.000000E+00	-3[illegible]
CPREdictor T2017	CPREdictor T2003	3.770779E-31	2.669258E+01	5.886922E-45	5[illegible]
CPREdictor T2017	CPREdictor T2003 w. GTGT	2.223317E-12	9.082298E+00	2.520094E-27	2[illegible]
CPREdictor T2017	CPREdictor T2017	-	-	-	
CPREdictor T2017	CPREdictor T2017 w. GTGT	9.997900E-01	-3.784664E+00	1.000000E+00	-1[illegible]
CPREdictor T2017	Dummy PREdictor w. GTGT	7.861661E-06	4.792053E+00	9.999748E-01	-4[illegible]
CPREdictor T2017 w. GTGT	CPREdictor T2003	8.049101E-32	2.760384E+01	5.393157E-45	5[illegible]
CPREdictor T2017 w. GTGT	CPREdictor T2003 w. GTGT	3.532833E-22	1.671251E+01	6.069041E-33	2[illegible]
CPREdictor T2017 w. GTGT	CPREdictor T2017	2.100401E-04	3.784664E+00	1.258567E-14	1[illegible]
CPREdictor T2017 w. GTGT	CPREdictor T2017 w. GTGT	-	-	-	
CPREdictor T2017 w. GTGT	Dummy PREdictor w. GTGT	7.262424E-20	1.467609E+01	2.262641E-03	2[illegible]
Dummy PREdictor w. GTGT	CPREdictor T2003	2.780187E-29	2.429137E+01	6.652054E-47	5[illegible]
Dummy PREdictor w. GTGT	CPREdictor T2003 w. GTGT	2.757511E-14	1.039286E+01	4.560255E-37	3[illegible]
Dummy PREdictor w. GTGT	CPREdictor T2017	9.999921E-01	-4.792053E+00	2.515157E-05	4[illegible]
Dummy PREdictor w. GTGT	CPREdictor T2017 w. GTGT	1.000000E+00	-1.467609E+01	9.977374E-01	-2[illegible]
Dummy PREdictor w. GTGT	Dummy PREdictor w. GTGT	-	-	-	

Exclusion/inclusion of GTGT - PRE training set: Schwartz 2010 PREs - Validation set: validation_PREsSchwartz vs. validation_CDS

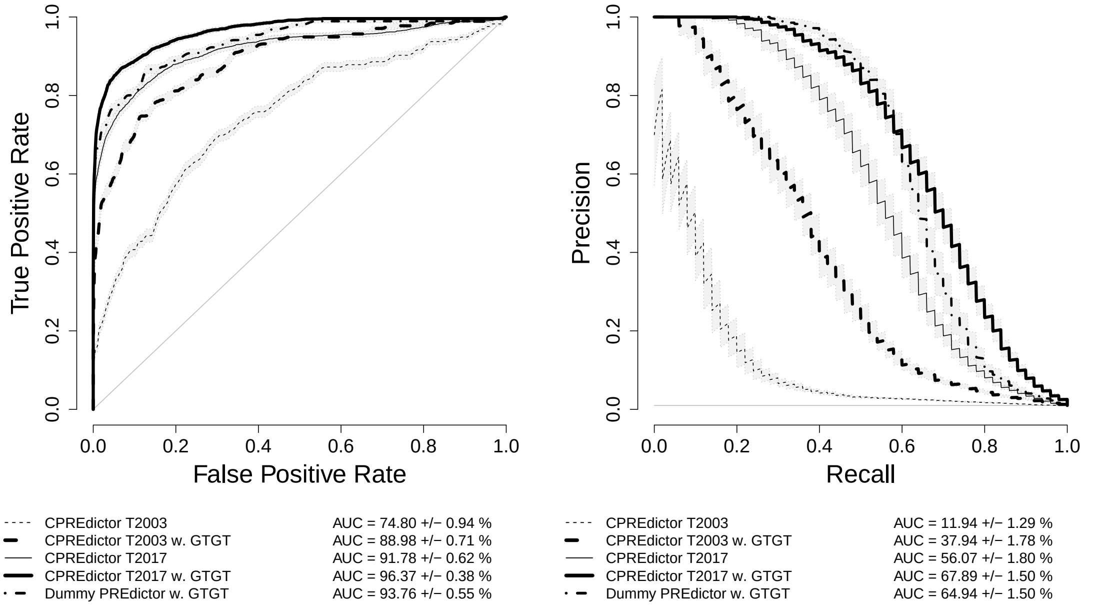

Classifier	ROC AUC
CPREdictor T2003	74.80 +/- 0.94 %
CPREdictor T2003 w. GTGT	88.98 +/- 0.71 %
CPREdictor T2017	91.78 +/- 0.62 %
CPREdictor T2017 w. GTGT	96.37 +/- 0.38 %
Dummy PREdictor w. GTGT	93.76 +/- 0.55 %

Classifier	PRC AUC
CPREdictor T2003	11.94 +/- 1.29 %
CPREdictor T2003 w. GTGT	37.94 +/- 1.78 %
CPREdictor T2017	56.07 +/- 1.80 %
CPREdictor T2017 w. GTGT	67.89 +/- 1.50 %
Dummy PREdictor w. GTGT	64.94 +/- 1.50 %

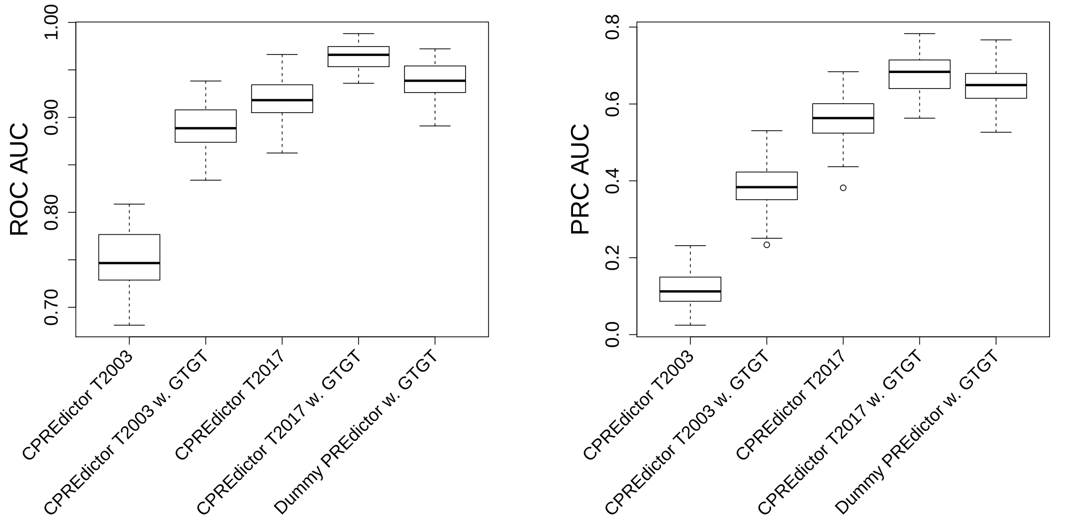

Classifier 1	Classifier 2	$p(H_0 : ROC1 <= ROC2)$	t	$p(H_0 : PRC1 <= PRC2)$	
CPREdictor T2003	CPREdictor T2003	-	-	-	
CPREdictor T2003	CPREdictor T2003 w. GTGT	1.000000E+00	-3.919928E+01	1.000000E+00	-3[illegible]
CPREdictor T2003	CPREdictor T2017	1.000000E+00	-3.732274E+01	1.000000E+00	-5[illegible]
CPREdictor T2003	CPREdictor T2017 w. GTGT	1.000000E+00	-4.607221E+01	1.000000E+00	-7[illegible]
CPREdictor T2003	Dummy PREdictor w. GTGT	1.000000E+00	-4.451863E+01	1.000000E+00	-7[illegible]
CPREdictor T2003 w. GTGT	CPREdictor T2003	5.825198E-39	3.919928E+01	1.608606E-37	3[illegible]
CPREdictor T2003 w. GTGT	CPREdictor T2003 w. GTGT	-	-	-	
CPREdictor T2003 w. GTGT	CPREdictor T2017	1.000000E+00	-7.743889E+00	1.000000E+00	-2[illegible]
CPREdictor T2003 w. GTGT	CPREdictor T2017 w. GTGT	1.000000E+00	-2.831699E+01	1.000000E+00	-4[illegible]
CPREdictor T2003 w. GTGT	Dummy PREdictor w. GTGT	1.000000E+00	-2.808669E+01	1.000000E+00	-4[illegible]
CPREdictor T2017	CPREdictor T2003	5.971102E-38	3.732274E+01	1.469455E-47	5[illegible]
CPREdictor T2017	CPREdictor T2003 w. GTGT	2.358164E-10	7.743889E+00	2.347277E-31	2[illegible]
CPREdictor T2017	CPREdictor T2017	-	-	-	
CPREdictor T2017	CPREdictor T2017 w. GTGT	1.000000E+00	-1.753386E+01	1.000000E+00	-2[illegible]
CPREdictor T2017	Dummy PREdictor w. GTGT	1.000000E+00	-6.426571E+00	1.000000E+00	-1[illegible]
CPREdictor T2017 w. GTGT	CPREdictor T2003	2.611054E-42	4.607221E+01	1.875927E-53	7[illegible]
CPREdictor T2017 w. GTGT	CPREdictor T2003 w. GTGT	2.479346E-32	2.831699E+01	6.380772E-41	4[illegible]
CPREdictor T2017 w. GTGT	CPREdictor T2017	4.683991E-23	1.753386E+01	6.043936E-31	2[illegible]
CPREdictor T2017 w. GTGT	CPREdictor T2017 w. GTGT	-	-	-	
CPREdictor T2017 w. GTGT	Dummy PREdictor w. GTGT	7.848617E-24	1.828423E+01	1.327323E-09	7[illegible]
Dummy PREdictor w. GTGT	CPREdictor T2003	1.349215E-41	4.451863E+01	2.452816E-52	7[illegible]
Dummy PREdictor w. GTGT	CPREdictor T2003 w. GTGT	3.616015E-32	2.808669E+01	1.065472E-43	4[illegible]
Dummy PREdictor w. GTGT	CPREdictor T2017	2.565824E-08	6.426571E+00	3.945098E-19	1[illegible]
Dummy PREdictor w. GTGT	CPREdictor T2017 w. GTGT	1.000000E+00	-1.828423E+01	1.000000E+00	-7[illegible]
Dummy PREdictor w. GTGT	Dummy PREdictor w. GTGT	-	-	-	

Exclusion/inclusion of GTGT - PRE training set: Schwartz 2010 PREs - Validation set: validation_PREsSchwartz vs. validation_D5merPREsSchwartz

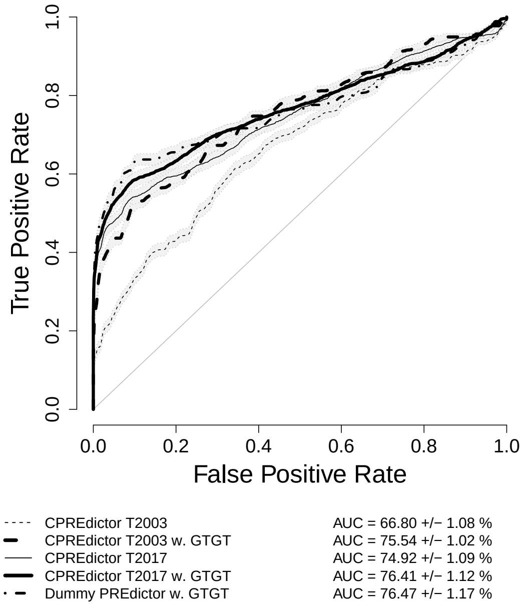

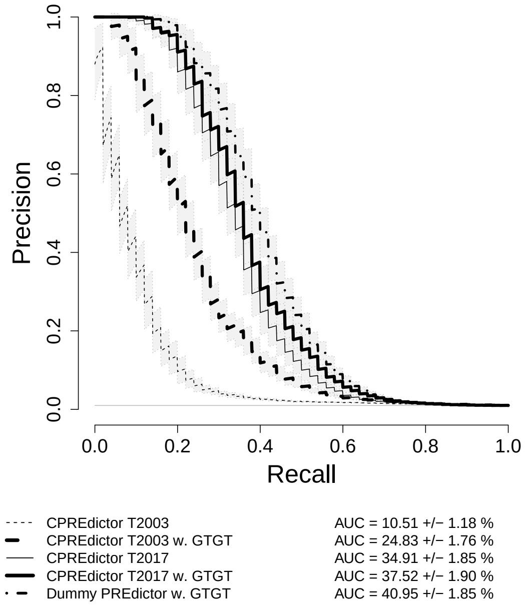

Classifier	ROC AUC
CPREdictor T2003	66.80 +/- 1.08 %
CPREdictor T2003 w. GTGT	75.54 +/- 1.02 %
CPREdictor T2017	74.92 +/- 1.09 %
CPREdictor T2017 w. GTGT	76.41 +/- 1.12 %
Dummy PREdictor w. GTGT	76.47 +/- 1.17 %

Classifier	PRC AUC
CPREdictor T2003	10.51 +/- 1.18 %
CPREdictor T2003 w. GTGT	24.83 +/- 1.76 %
CPREdictor T2017	34.91 +/- 1.85 %
CPREdictor T2017 w. GTGT	37.52 +/- 1.90 %
Dummy PREdictor w. GTGT	40.95 +/- 1.85 %

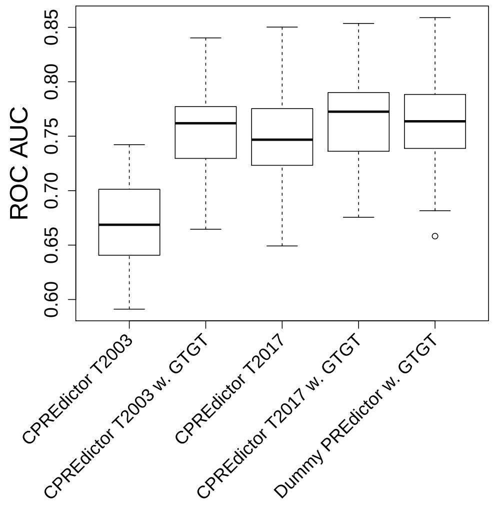

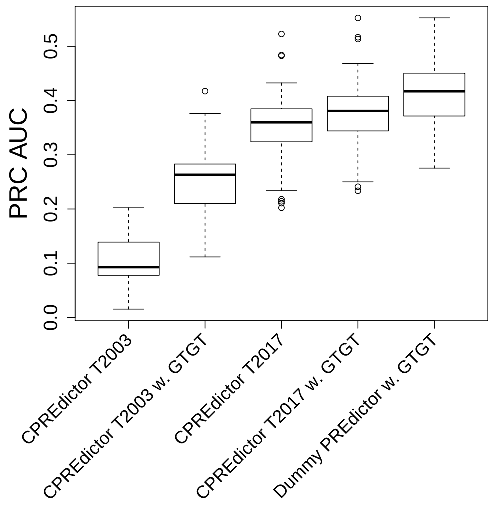

Classifier 1	Classifier 2	$p(H_0 : ROC1 <= ROC2)$	t	$p(H_0 : PRC1 <= PRC2)$	
CPREdictor T2003	CPREdictor T2003	-	-	-	
CPREdictor T2003	CPREdictor T2003 w. GTGT	1.000000E+00	-1.985943E+01	1.000000E+00	-2
CPREdictor T2003	CPREdictor T2017	1.000000E+00	-1.357770E+01	1.000000E+00	-2
CPREdictor T2003	CPREdictor T2017 w. GTGT	1.000000E+00	-1.567490E+01	1.000000E+00	-3
CPREdictor T2003	Dummy PREdictor w. GTGT	1.000000E+00	-1.758233E+01	1.000000E+00	-3
CPREdictor T2003 w. GTGT	CPREdictor T2003	2.194621E-25	1.985943E+01	1.022709E-26	2
CPREdictor T2003 w. GTGT	CPREdictor T2003 w. GTGT	-	-	-	
CPREdictor T2003 w. GTGT	CPREdictor T2017	1.210747E-01	1.183938E+00	1.000000E+00	-1
CPREdictor T2003 w. GTGT	CPREdictor T2017 w. GTGT	9.837843E-01	-2.201615E+00	1.000000E+00	-2
CPREdictor T2003 w. GTGT	Dummy PREdictor w. GTGT	9.994986E-01	-3.499498E+00	1.000000E+00	-3
CPREdictor T2017	CPREdictor T2003	1.570290E-18	1.357770E+01	4.664484E-33	2
CPREdictor T2017	CPREdictor T2003 w. GTGT	8.789253E-01	-1.183938E+00	1.130833E-23	1
CPREdictor T2017	CPREdictor T2017	-	-	-	
CPREdictor T2017	CPREdictor T2017 w. GTGT	9.999424E-01	-4.192038E+00	1.000000E+00	-1
CPREdictor T2017	Dummy PREdictor w. GTGT	9.991435E-01	-3.318416E+00	1.000000E+00	-1
CPREdictor T2017 w. GTGT	CPREdictor T2003	5.026969E-21	1.567490E+01	2.984238E-34	3
CPREdictor T2017 w. GTGT	CPREdictor T2003 w. GTGT	1.621575E-02	2.201615E+00	1.686946E-27	2
CPREdictor T2017 w. GTGT	CPREdictor T2017	5.761909E-05	4.192038E+00	1.188161E-15	1
CPREdictor T2017 w. GTGT	CPREdictor T2017 w. GTGT	-	-	-	
CPREdictor T2017 w. GTGT	Dummy PREdictor w. GTGT	5.896643E-01	-2.279011E-01	1.000000E+00	-8
Dummy PREdictor w. GTGT	CPREdictor T2003	4.166453E-23	1.758233E+01	1.154506E-37	3
Dummy PREdictor w. GTGT	CPREdictor T2003 w. GTGT	5.014163E-04	3.499498E+00	1.377062E-35	3
Dummy PREdictor w. GTGT	CPREdictor T2017	8.564522E-04	3.318416E+00	4.532895E-17	1
Dummy PREdictor w. GTGT	CPREdictor T2017 w. GTGT	4.103357E-01	2.279011E-01	4.827804E-11	8
Dummy PREdictor w. GTGT	Dummy PREdictor w. GTGT	-	-	-	

GTGT versus random 4-mers (training set PREs: Schwartz 2010 PREs)

Classifier	Path
M2003	CPREdictor_M2003_CPPREsSchwartz_CND5merPREsSchwartz_T110_mdBetween_wmPREdictor
M2003 + ACGG	CPREdictor_M2003_ACGG_CPPREsSchwartz_CND5merPREsSchwartz_T110_mdBetween_wmPREdictor
M2003 + GGCG	CPREdictor_M2003_GGCG_CPPREsSchwartz_CND5merPREsSchwartz_T110_mdBetween_wmPREdictor
M2003 + GCTC	CPREdictor_M2003_GCTC_CPPREsSchwartz_CND5merPREsSchwartz_T110_mdBetween_wmPREdictor
M2003 + ATGT	CPREdictor_M2003_ATGT_CPPREsSchwartz_CND5merPREsSchwartz_T110_mdBetween_wmPREdictor
M2003 + TAAG	CPREdictor_M2003_TAAG_CPPREsSchwartz_CND5merPREsSchwartz_T110_mdBetween_wmPREdictor
M2003 + CGTT	CPREdictor_M2003_CGTT_CPPREsSchwartz_CND5merPREsSchwartz_T110_mdBetween_wmPREdictor
M2003 + TGGC	CPREdictor_M2003_TGGC_CPPREsSchwartz_CND5merPREsSchwartz_T110_mdBetween_wmPREdictor
M2003 + GTCG	CPREdictor_M2003_GTCG_CPPREsSchwartz_CND5merPREsSchwartz_T110_mdBetween_wmPREdictor
M2003 + TGCA	CPREdictor_M2003_TGCA_CPPREsSchwartz_CND5merPREsSchwartz_T110_mdBetween_wmPREdictor
M2003 + CCCC	CPREdictor_M2003_CCCC_CPPREsSchwartz_CND5merPREsSchwartz_T110_mdBetween_wmPREdictor
M2003 + TGAG	CPREdictor_M2003_TGAG_CPPREsSchwartz_CND5merPREsSchwartz_T110_mdBetween_wmPREdictor
M2003 + TGAC	CPREdictor_M2003_TGAC_CPPREsSchwartz_CND5merPREsSchwartz_T110_mdBetween_wmPREdictor
M2003 + ATAG	CPREdictor_M2003_ATAG_CPPREsSchwartz_CND5merPREsSchwartz_T110_mdBetween_wmPREdictor
M2003 + CACG	CPREdictor_M2003_CACG_CPPREsSchwartz_CND5merPREsSchwartz_T110_mdBetween_wmPREdictor
M2003 + ATGG	CPREdictor_M2003_ATGG_CPPREsSchwartz_CND5merPREsSchwartz_T110_mdBetween_wmPREdictor
M2003 + TTAT	CPREdictor_M2003_TTAT_CPPREsSchwartz_CND5merPREsSchwartz_T110_mdBetween_wmPREdictor
M2003 + GGAA	CPREdictor_M2003_GGAA_CPPREsSchwartz_CND5merPREsSchwartz_T110_mdBetween_wmPREdictor
M2003 + GGGA	CPREdictor_M2003_GGGA_CPPREsSchwartz_CND5merPREsSchwartz_T110_mdBetween_wmPREdictor
M2003 + GAGT	CPREdictor_M2003_GAGT_CPPREsSchwartz_CND5merPREsSchwartz_T110_mdBetween_wmPREdictor
M2003 + GTGT	CPREdictor_M2003_GTGT_CPPREsSchwartz_CND5merPREsSchwartz_T110_mdBetween_wmPREdictor

GTGT versus random 4-mers - PRE training set: Schwartz 2010 PREs - Validation set: validation_PREsSchwartz vs. validation_R5mers

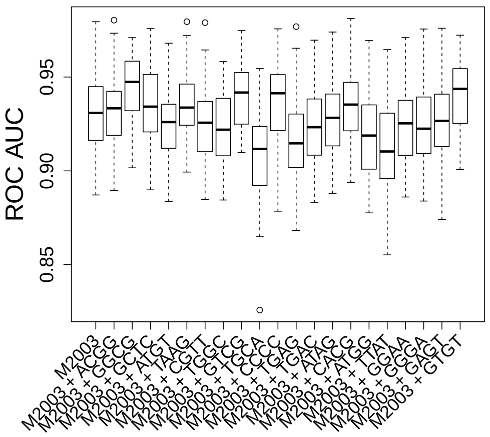

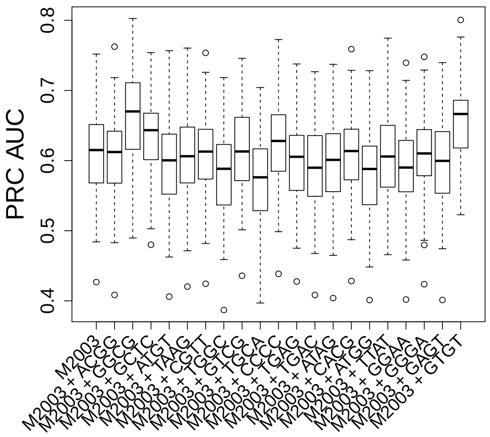

Run	Mean ROC AUC	ROC AUC rank	Mean PRC AUC	PRC AUC rank
M2003	93.04±0.56%	9	60.85±1.84%	6
M2003 + ACGG	93.15±0.55%	8	60.28±1.87%	12
M2003 + GGCG	94.57±0.45%	1	66.09±1.70%	1
M2003 + GCTC	93.36±0.57%	7	63.06±1.72%	3
M2003 + ATGT	92.55±0.57%	12	59.04±1.91%	16
M2003 + TAAG	93.64±0.50%	5	60.40±1.92%	11
M2003 + CGTT	92.51±0.56%	13	60.53±1.83%	8
M2003 + TGGC	92.28±0.53%	17	58.07±1.94%	19
M2003 + GTCG	94.05±0.48%	3	61.20±1.81%	5
M2003 + TGCA	90.81±0.68%	21	56.53±1.92%	21
M2003 + CCCC	93.74±0.55%	4	62.41±1.87%	4
M2003 + TGAG	91.61±0.65%	19	59.75±1.83%	13
M2003 + TGAC	92.33±0.55%	16	58.80±1.87%	17
M2003 + ATAG	92.82±0.54%	10	59.51±1.92%	15
M2003 + CACG	93.42±0.52%	6	60.73±1.87%	7
M2003 + ATGG	91.94±0.59%	18	57.95±1.93%	20
M2003 + TTAT	91.01±0.65%	20	60.44±1.83%	10
M2003 + GGAA	92.44±0.54%	15	58.76±1.89%	18
M2003 + GGGA	92.48±0.59%	14	60.49±1.85%	9
M2003 + GAGT	92.70±0.62%	11	59.54±1.91%	14
M2003 + GTGT	94.05±0.52%	2	65.49±1.78%	2

GTGT versus random 4-mers - PRE training set: Schwartz 2010 PREs - Validation set: validation_PREsSchwartz vs. validation_CDS

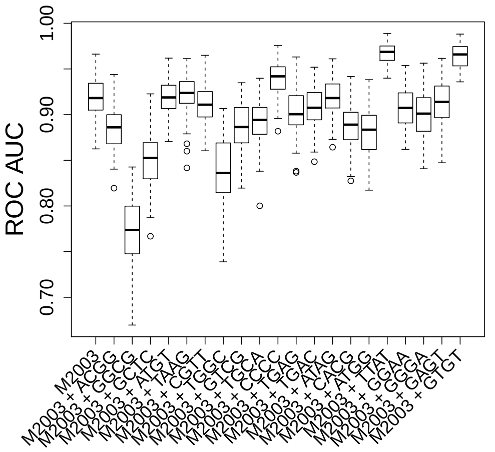

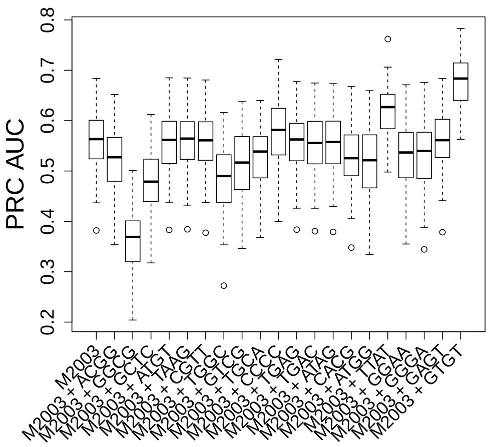

Run	Mean ROC AUC	ROC AUC rank	Mean PRC AUC	PRC AUC rank
M2003	91.78±0.62%	7	56.07±1.80%	5
M2003 + ACGG	88.48±0.70%	17	52.13±1.78%	16
M2003 + GGCG	77.07±1.19%	21	35.91±1.86%	21
M2003 + GCTC	85.14±0.89%	19	47.55±1.92%	20
M2003 + ATGT	91.89±0.62%	5	55.59±1.79%	9
M2003 + TAAG	92.13±0.69%	4	55.70±1.79%	7
M2003 + CGTT	91.17±0.61%	9	55.63±1.78%	8
M2003 + TGGC	83.64±1.09%	20	47.97±1.97%	19
M2003 + GTCG	88.60±0.74%	16	51.62±1.81%	17
M2003 + TGCA	89.16±0.75%	14	52.99±1.82%	14
M2003 + CCCC	93.90±0.53%	3	57.57±1.83%	3
M2003 + TGAG	90.19±0.73%	12	55.87±1.81%	6
M2003 + TGAC	90.77±0.63%	10	55.00±1.78%	11
M2003 + ATAG	91.80±0.59%	6	55.46±1.79%	10
M2003 + CACG	88.80±0.73%	15	52.72±1.84%	15
M2003 + ATGG	88.26±0.78%	18	51.47±1.97%	18
M2003 + TTAT	96.78±0.30%	1	61.79±1.61%	2
M2003 + GGAA	90.65±0.59%	11	53.06±1.84%	13
M2003 + GGGA	89.95±0.72%	13	53.08±1.86%	12
M2003 + GAGT	91.49±0.67%	8	56.18±1.82%	4
M2003 + GTGT	96.37±0.38%	2	67.89±1.50%	1

GTGT versus random 4-mers - PRE training set: Schwartz 2010 PREs - Validation set: validation_PREsSchwartz vs. validation_D5merPREsSchwartz

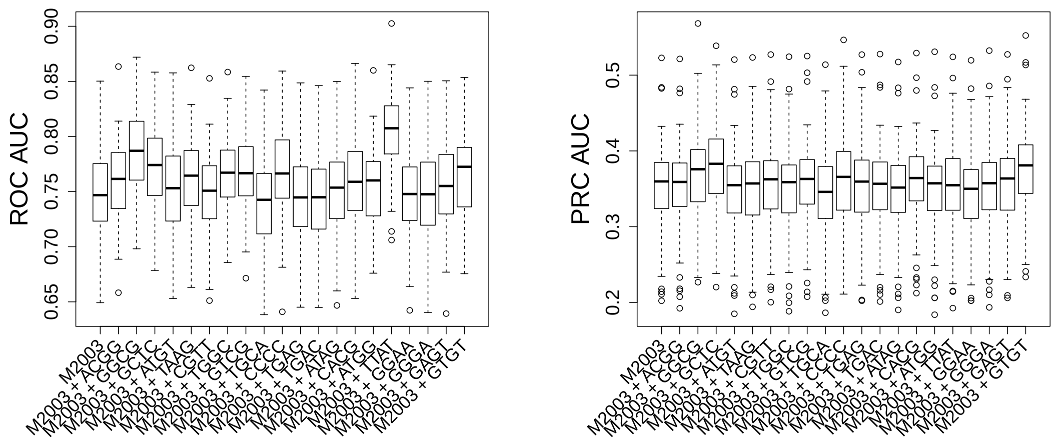

Run	Mean ROC AUC	ROC AUC rank	Mean PRC AUC	PRC AUC rank
M2003	74.92±1.09%	15	34.91±1.85%	13
M2003 + ACGG	75.99±1.03%	9	34.83±1.86%	14
M2003 + GGCG	78.49±1.01%	2	36.80±1.86%	3
M2003 + GCTC	76.95±1.08%	3	37.55±1.89%	1
M2003 + ATGT	75.21±1.13%	13	34.50±1.87%	18
M2003 + TAAG	76.21±1.13%	8	34.69±1.86%	17
M2003 + CGTT	74.89±1.07%	16	35.47±1.84%	8
M2003 + TGGC	76.52±1.02%	6	34.78±1.85%	15
M2003 + GTCG	76.71±1.04%	4	35.63±1.82%	6
M2003 + TGCA	74.05±1.14%	21	34.14±1.87%	21
M2003 + CCCC	76.70±1.23%	5	35.99±1.89%	4
M2003 + TGAG	74.49±1.12%	19	35.34±1.92%	9
M2003 + TGAC	74.24±1.11%	20	34.93±1.86%	12
M2003 + ATAG	75.17±1.11%	14	34.48±1.85%	19
M2003 + CACG	75.79±1.13%	10	35.76±1.83%	5
M2003 + ATGG	75.43±1.07%	11	34.69±1.88%	16
M2003 + TTAT	80.30±1.07%	1	35.10±1.92%	10
M2003 + GGAA	74.80±1.08%	18	34.20±1.82%	20
M2003 + GGGA	74.87±1.13%	17	34.94±1.86%	11
M2003 + GAGT	75.33±1.13%	12	35.52±1.89%	7
M2003 + GTGT	76.41±1.12%	7	37.52±1.90%	2

Tests of extra published motifs (training set PREs Schwartz 2010 PREs)

Classifier	Path
M2003	CPREdictor_M2003_CPPREsSchwartz_CND5merPREsSchwartz_T110_mdBetween_wmPREdictor
M2003 + Zeste2	CPREdictor_M2003_Zeste2_CPPREsSchwartz_CND5merPREsSchwartz_T110_mdBetween_wmPREdictor
M2003 + Sp1Klf	CPREdictor_M2003_Sp1Klf_CPPREsSchwartz_CND5merPREsSchwartz_T110_mdBetween_wmPREdictor
M2003 + Dsp1	CPREdictor_M2003_Dsp1_CPPREsSchwartz_CND5merPREsSchwartz_T110_mdBetween_wmPREdictor
M2003 + Grh1	CPREdictor_M2003_Grh1_CPPREsSchwartz_CND5merPREsSchwartz_T110_mdBetween_wmPREdictor
M2003 + Grh2	CPREdictor_M2003_Grh2_CPPREsSchwartz_CND5merPREsSchwartz_T110_mdBetween_wmPREdictor
M2003 + SiteA	CPREdictor_M2003_SiteA_CPPREsSchwartz_CND5merPREsSchwartz_T110_mdBetween_wmPREdictor
M2003 + GTGT	CPREdictor_M2003_GTGT_CPPREsSchwartz_CND5merPREsSchwartz_T110_mdBetween_wmPREdictor
M2003 + all extra	CPREdictor_M2003_GTGT_extraAll_CPPREsSchwartz_CND5merPREsSchwartz_T110_mdBetween_wmPREdictor

Tests of extra published motifs - PRE training set: Schwartz 2010 PREs - Validation set: validation_PREsSchwartz vs. validation_R5mers

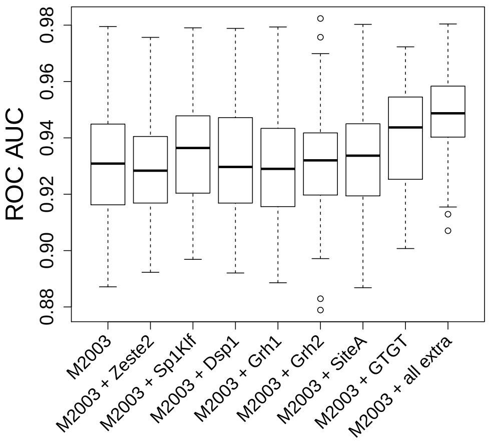

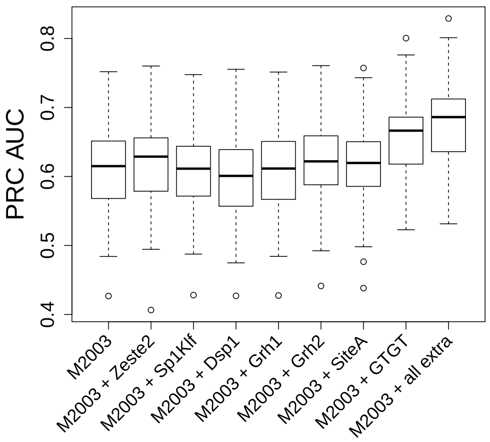

Run	Mean ROC AUC	ROC AUC rank	Mean PRC AUC	PRC AUC rank
M2003	93.04±0.56%	7	60.85±1.84%	6
M2003 + Zeste2	92.85±0.53%	9	61.78±1.88%	4
M2003 + Sp1Klf	93.57±0.50%	3	60.82±1.82%	7
M2003 + Dsp1	93.05±0.54%	6	59.73±1.81%	9
M2003 + Grh1	92.96±0.55%	8	60.73±1.84%	8
M2003 + Grh2	93.10±0.59%	5	61.96±1.78%	3
M2003 + SiteA	93.17±0.56%	4	61.47±1.84%	5
M2003 + GTGT	94.05±0.52%	2	65.49±1.78%	2
M2003 + all extra	94.80±0.48%	1	67.56±1.78%	1

Tests of extra published motifs - PRE training set: Schwartz 2010 PREs - Validation set: validation_PREsSchwartz vs. validation_CDS

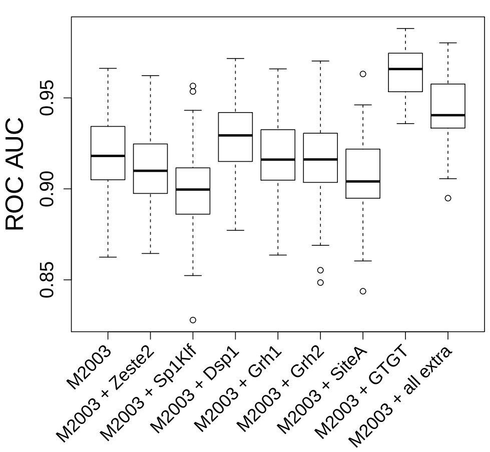

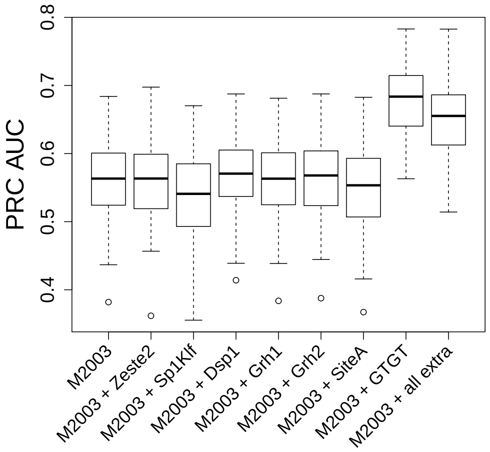

Run	Mean ROC AUC	ROC AUC rank	Mean PRC AUC	PRC AUC rank
M2003	91.78±0.62%	4	56.07±1.80%	6
M2003 + Zeste2	91.11±0.61%	7	56.50±1.83%	4
M2003 + Sp1Klf	89.90±0.68%	9	53.85±1.84%	9
M2003 + Dsp1	92.88±0.55%	3	56.79±1.66%	3
M2003 + Grh1	91.64±0.62%	5	56.06±1.78%	7
M2003 + Grh2	91.52±0.68%	6	56.36±1.74%	5
M2003 + SiteA	90.46±0.67%	8	54.78±1.82%	8
M2003 + GTGT	96.37±0.38%	1	67.89±1.50%	1
M2003 + all extra	94.26±0.55%	2	65.23±1.57%	2

Tests of extra published motifs - PRE training set: Schwartz 2010 PREs - Validation set: validation_PREsSchwartz vs. validation_D5merPREsSchwartz

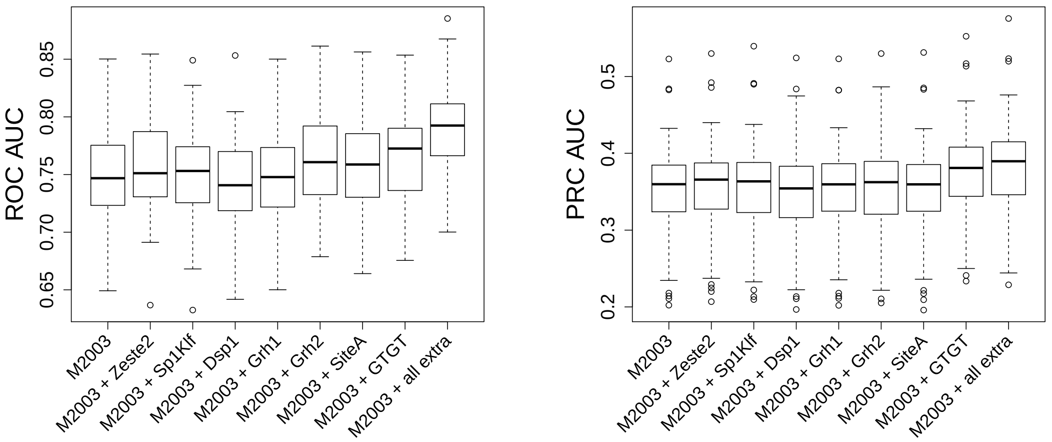

Run	Mean ROC AUC	ROC AUC rank	Mean PRC AUC	PRC AUC rank
M2003	74.92±1.09%	7	34.91±1.85%	8
M2003 + Zeste2	75.68±1.09%	5	35.62±1.87%	3
M2003 + Sp1Klf	75.31±1.12%	6	35.43±1.86%	4
M2003 + Dsp1	74.31±1.13%	9	34.76±1.84%	9
M2003 + Grh1	74.82±1.09%	8	34.91±1.85%	7
M2003 + Grh2	76.06±1.05%	3	35.36±1.87%	5
M2003 + SiteA	75.89±1.06%	4	34.97±1.87%	6
M2003 + GTGT	76.41±1.12%	2	37.52±1.90%	2
M2003 + all extra	79.00±1.07%	1	38.34±1.92%	1

Classifier window size (training set PREs: Schwartz 2010 PREs)

- - - - CPREdictor 500bp
—— CPREdictor 3k
▬ SVM−MOCCA 500bp
━━ SVM−MOCCA 3k

Classifier	Path
CPREdictor 500bp	CPREdictor_M2003_GTGT_CPPREsSchwartz_CND5merPREsSchwartz_T110_mdBetween_wmPREdictor
CPREdictor 3k	CPREdictor_M2003_GTGT_CPPREsSchwartz_CND5merPREsSchwartz_T110_ws3000_mdBetween_wmPREdictor
SVM-MOCCA 500bp	SVMMOCCA_fnOcc_fDNT_M2003_GTGT_CPPREsSchwartz_CNR5mers_CNCDS_CND5merPREsSchwartz_T110_mdBetween_wmPREdictor
SVM-MOCCA 3k	SVMMOCCA_klinear_fnOcc_fDNT_M2003_GTGT_CPPREsSchwartz_CNR5mers_CNCDS_CND5merPREsSchwartz_T110_ws3000_mdBetween_wmPREdictor

Classifier window size - PRE training set: Schwartz 2010 PREs - Validation set: validation_PREsSchwartz vs. validation_R5mers

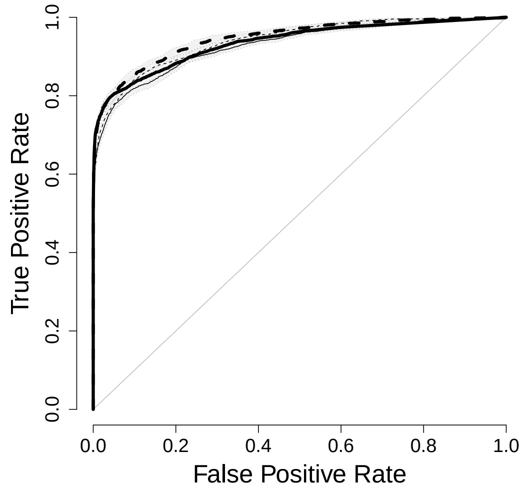

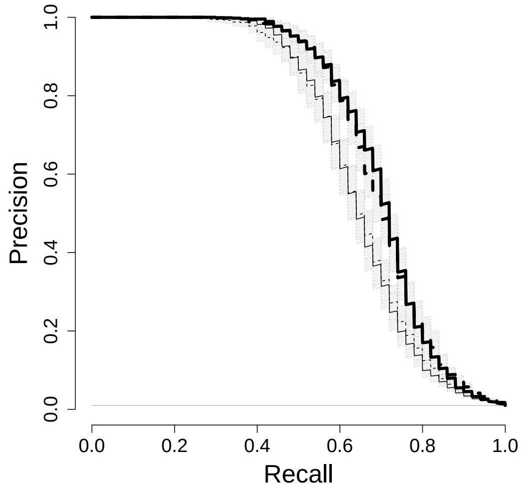

- - - -	CPREdictor 500bp	AUC = 94.05 +/− 0.52 %
——	CPREdictor 3k	AUC = 92.85 +/− 0.62 %
▬	SVM−MOCCA 500bp	AUC = 94.75 +/− 0.55 %
━━	SVM−MOCCA 3k	AUC = 93.56 +/− 0.60 %

- - - -	CPREdictor 500bp	AUC = 65.49 +/− 1.78 %
——	CPREdictor 3k	AUC = 65.25 +/− 1.75 %
▬	SVM−MOCCA 500bp	AUC = 70.31 +/− 1.62 %
━━	SVM−MOCCA 3k	AUC = 70.70 +/− 1.59 %

Classifier	ROC AUC
CPREdictor 500bp	94.05 +/- 0.52 %
CPREdictor 3k	92.85 +/- 0.62 %
SVM-MOCCA 500bp	94.75 +/- 0.55 %
SVM-MOCCA 3k	93.56 +/- 0.60 %

Classifier	PRC AUC
CPREdictor 500bp	65.49 +/- 1.78 %
CPREdictor 3k	65.25 +/- 1.75 %
SVM-MOCCA 500bp	70.31 +/- 1.62 %
SVM-MOCCA 3k	70.70 +/- 1.59 %

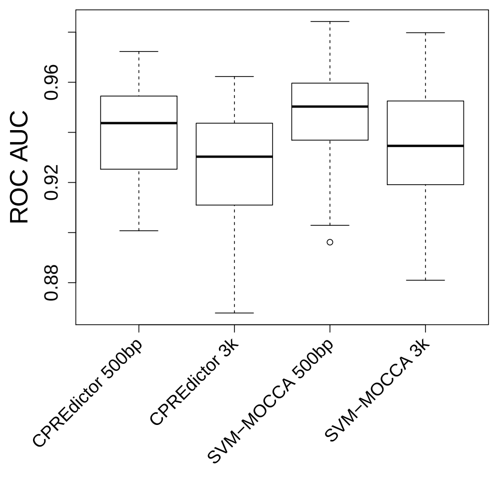

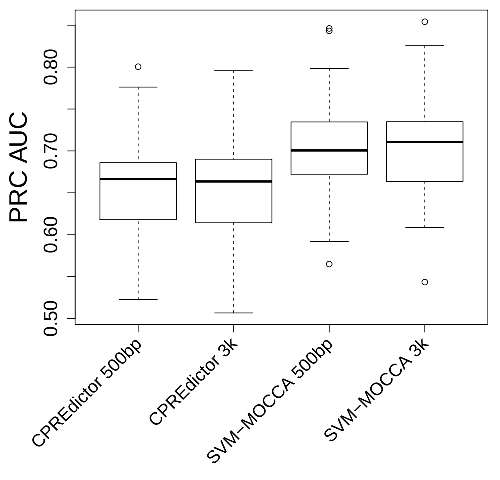

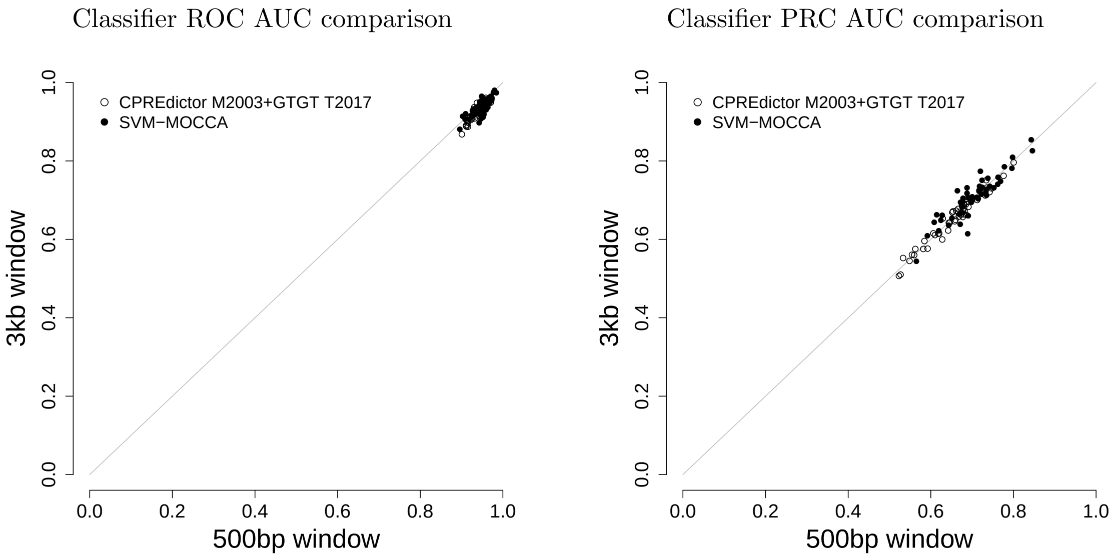
Classifier ROC AUC comparison
CPREdictor M2003+GTGT T2017
SVM−MOCCA
3kb window
500bp window
0.0
0.2
0.4
0.6
0.8
1.0
Classifier PRC AUC comparison
CPREdictor M2003+GTGT T2017
SVM−MOCCA
3kb window
500bp window

Classifier window size - PRE training set: Schwartz 2010 PREs - Validation set: validation_PREsSchwartz vs. validation_CDS

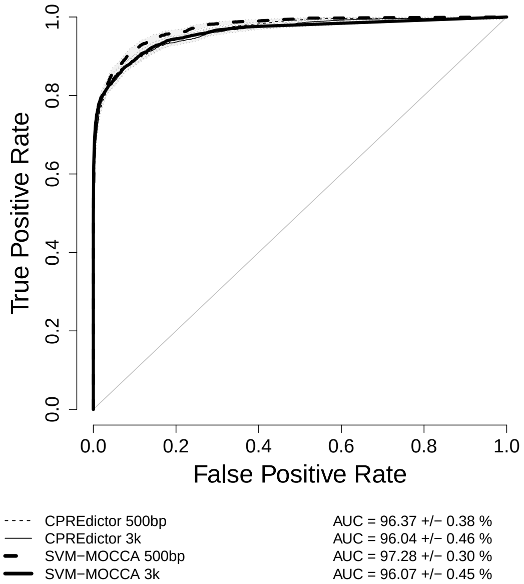

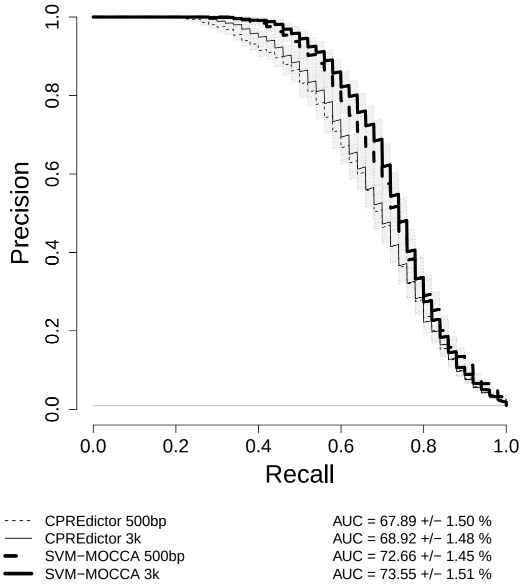

Classifier	ROC AUC
CPREdictor 500bp	96.37 +/- 0.38 %
CPREdictor 3k	96.04 +/- 0.46 %
SVM-MOCCA 500bp	97.28 +/- 0.30 %
SVM-MOCCA 3k	96.07 +/- 0.45 %

Classifier	PRC AUC
CPREdictor 500bp	67.89 +/- 1.50 %
CPREdictor 3k	68.92 +/- 1.48 %
SVM-MOCCA 500bp	72.66 +/- 1.45 %
SVM-MOCCA 3k	73.55 +/- 1.51 %

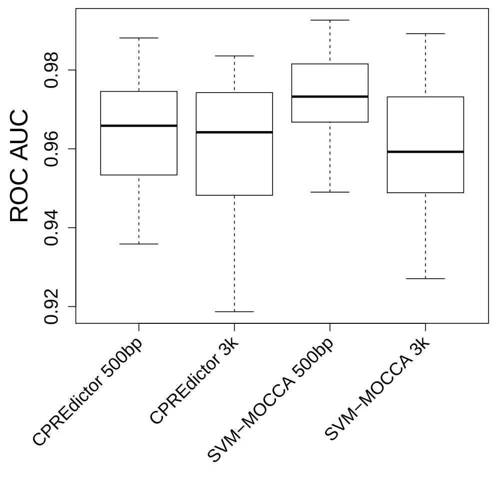

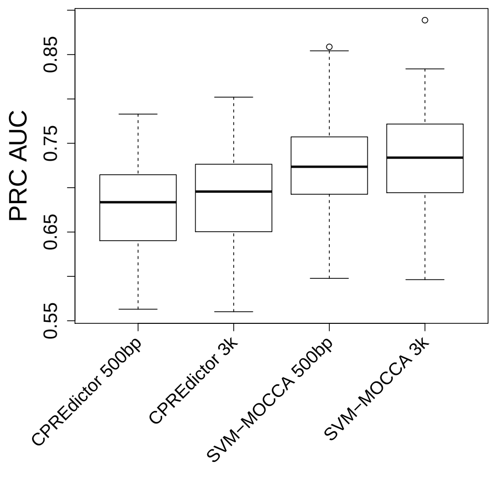

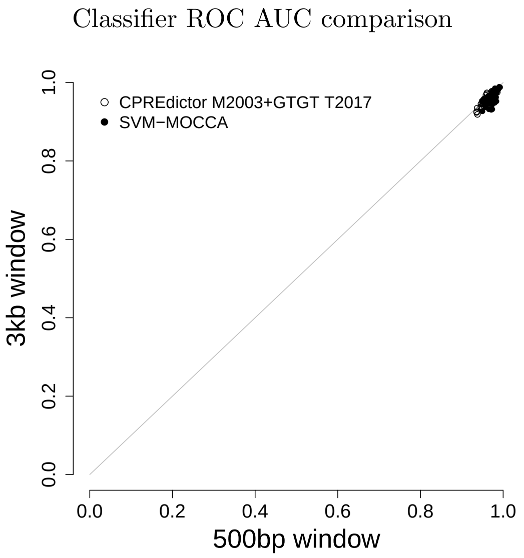

Classifier ROC AUC comparison
CPREdictor M2003+GTGT T2017
SVM−MOCCA
3kb window
500bp window
0.0
0.2
0.4
0.6
0.8
1.0

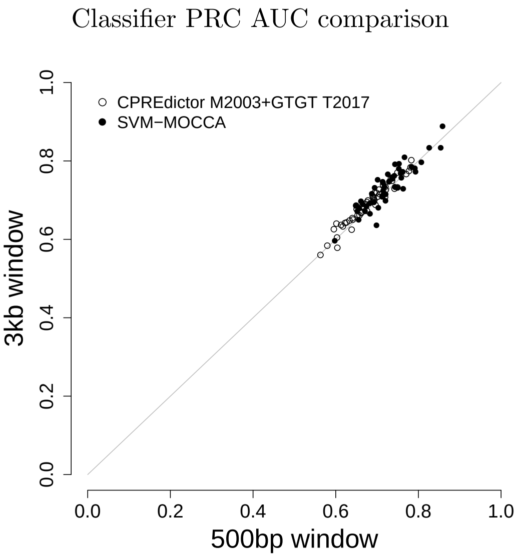

Classifier PRC AUC comparison
CPREdictor M2003+GTGT T2017
SVM−MOCCA
3kb window
500bp window
0.0
0.2
0.4
0.6
0.8
1.0

Classifier window size - PRE training set: Schwartz 2010 PREs - Validation set: validation_PREsSchwartz vs. validation_D5merPREsSchwartz

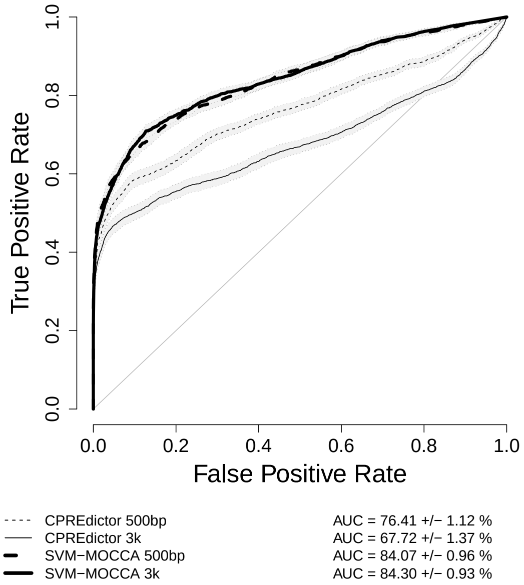

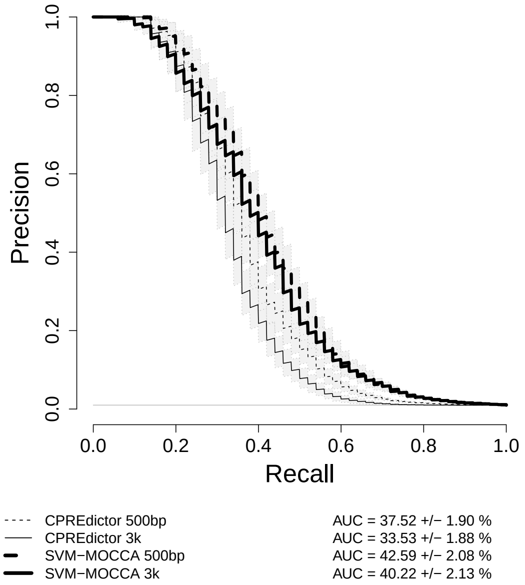

Classifier	ROC AUC
CPREdictor 500bp	76.41 +/- 1.12 %
CPREdictor 3k	67.72 +/- 1.37 %
SVM-MOCCA 500bp	84.07 +/- 0.96 %
SVM-MOCCA 3k	84.30 +/- 0.93 %

Classifier	PRC AUC
CPREdictor 500bp	37.52 +/- 1.90 %
CPREdictor 3k	33.53 +/- 1.88 %
SVM-MOCCA 500bp	42.59 +/- 2.08 %
SVM-MOCCA 3k	40.22 +/- 2.13 %

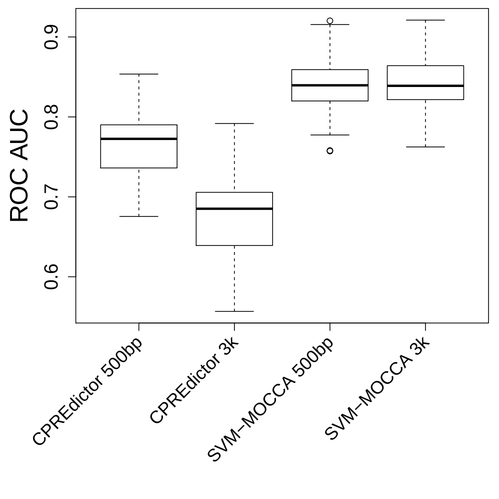

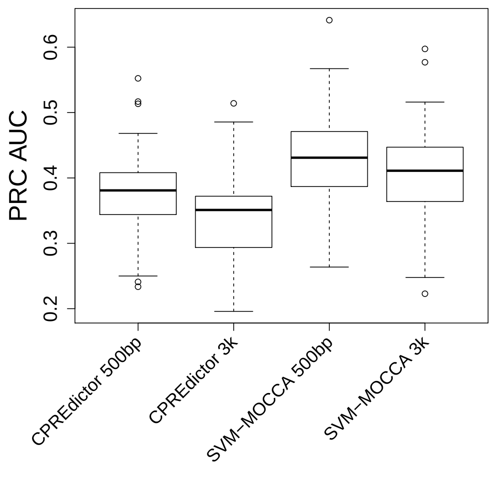

Classifier ROC AUC comparison

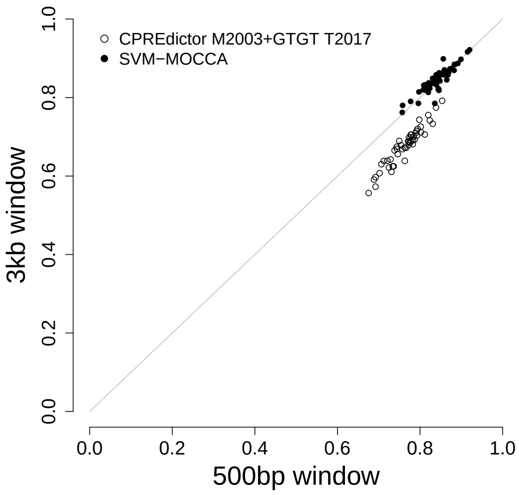

Classifier PRC AUC comparison

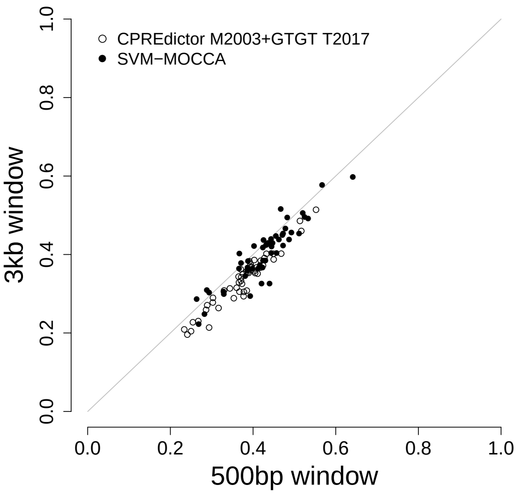

SVM-MOCCA kernel functions (training set PREs: Schwartz 2010 PREs) - 3kb window

---- SVM−MOCCA linear
SVM−MOCCA quadratic
SVM−MOCCA cubic

Classifier	Path
SVM-MOCCA linear	SVMMOCCA_klinear_fnOcc_fDNT_M2003_GTGT_CPPREsSchwartz_CNR5mers_CNCDS_CND5merPREsSchwartz_T110_ws3000_mdBetween_wmPREdic
SVM-MOCCA quadratic	SVMMOCCA_kquadratic_fnOcc_fDNT_M2003_GTGT_CPPREsSchwartz_CNR5mers_CNCDS_CND5merPREsSchwartz_T110_ws3000_mdBetween_wmPRE
SVM-MOCCA cubic	SVMMOCCA_kcubic_fnOcc_fDNT_M2003_GTGT_CPPREsSchwartz_CNR5mers_CNCDS_CND5merPREsSchwartz_T110_ws3000_mdBetween_wmPREdict

SVM-MOCCA kernel functions - PRE training set: Schwartz 2010 PREs - Validation set: validation_PREsSchwartz vs. validation_R5mers

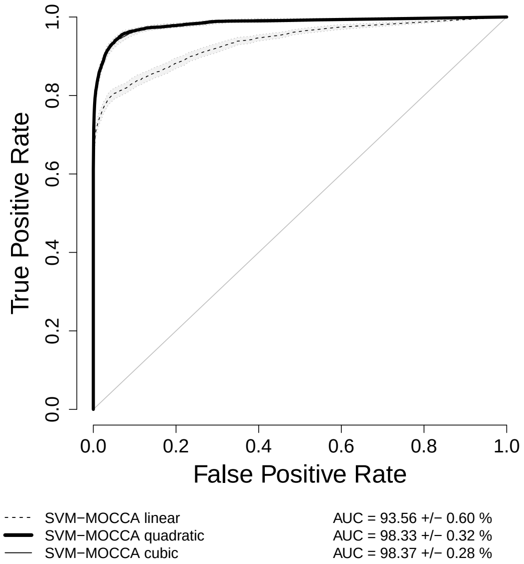

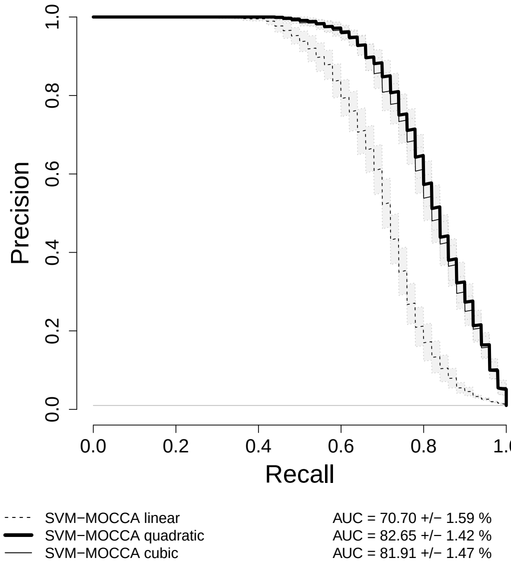

Classifier	ROC AUC
SVM-MOCCA linear	93.56 +/- 0.60 %
SVM-MOCCA quadratic	98.33 +/- 0.32 %
SVM-MOCCA cubic	98.37 +/- 0.28 %

Classifier	PRC AUC
SVM-MOCCA linear	70.70 +/- 1.59 %
SVM-MOCCA quadratic	82.65 +/- 1.42 %
SVM-MOCCA cubic	81.91 +/- 1.47 %

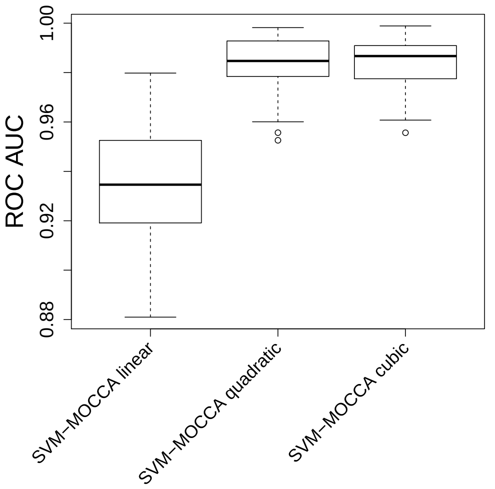

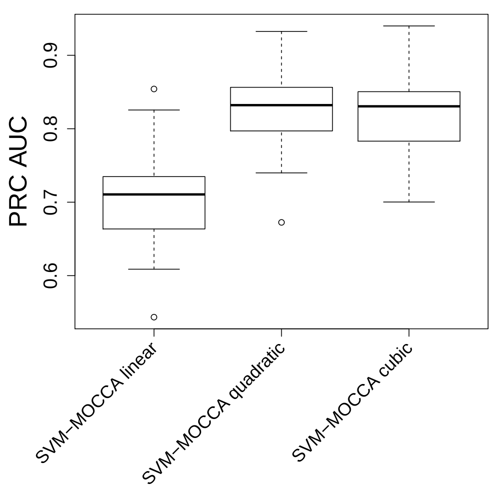

Classifier 1	Classifier 2	$p(H_0 : ROC1 <= ROC2)$	t	$p(H_0 : PRC1 <= PRC2)$	
SVM-MOCCA linear	SVM-MOCCA linear	-	-	-	
SVM-MOCCA linear	SVM-MOCCA quadratic	1.000000E+00	-1.771821E+01	1.000000E+00	-2.054930
SVM-MOCCA linear	SVM-MOCCA cubic	1.000000E+00	-1.847835E+01	1.000000E+00	-1.849810
SVM-MOCCA quadratic	SVM-MOCCA linear	3.004512E-23	1.771821E+01	4.909801E-26	2.054930
SVM-MOCCA quadratic	SVM-MOCCA quadratic	-	-	-	
SVM-MOCCA quadratic	SVM-MOCCA cubic	6.396879E-01	-3.596905E-01	5.167015E-04	3.489465
SVM-MOCCA cubic	SVM-MOCCA linear	4.988459E-24	1.847835E+01	4.764646E-24	1.849810
SVM-MOCCA cubic	SVM-MOCCA quadratic	3.603121E-01	3.596905E-01	9.994833E-01	-3.489465
SVM-MOCCA cubic	SVM-MOCCA cubic	-	-	-	

SVM-MOCCA kernel functions - PRE training set: Schwartz 2010 PREs - Validation set: validation_PREsSchwartz vs. validation_CDS

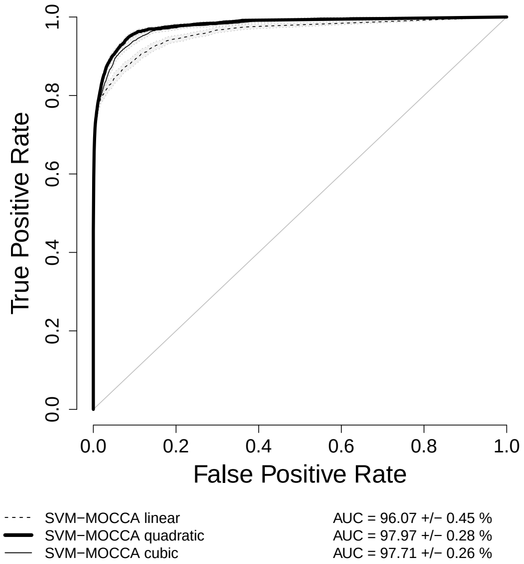

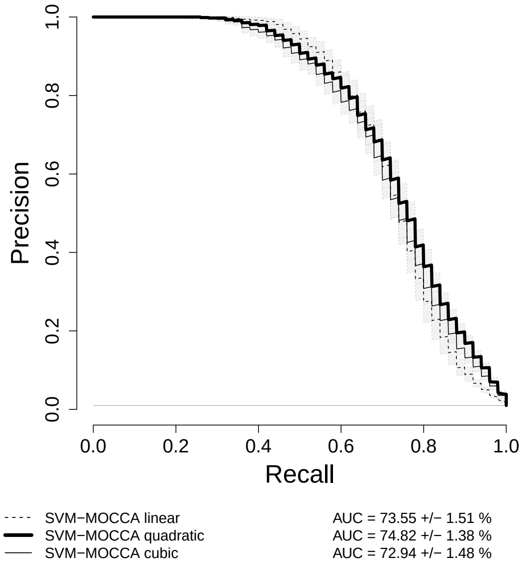

Classifier	ROC AUC
SVM-MOCCA linear	96.07 +/- 0.45 %
SVM-MOCCA quadratic	97.97 +/- 0.28 %
SVM-MOCCA cubic	97.71 +/- 0.26 %

Classifier	PRC AUC
SVM-MOCCA linear	73.55 +/- 1.51 %
SVM-MOCCA quadratic	74.82 +/- 1.38 %
SVM-MOCCA cubic	72.94 +/- 1.48 %

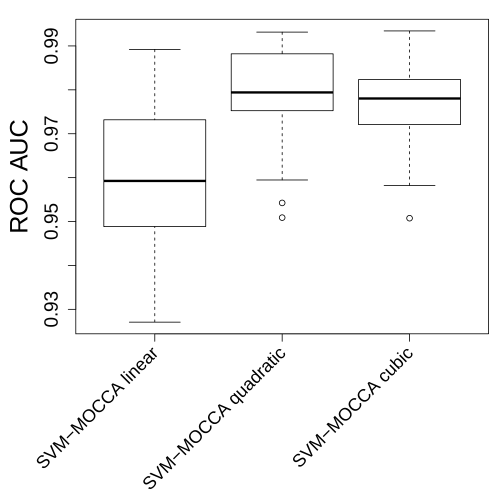

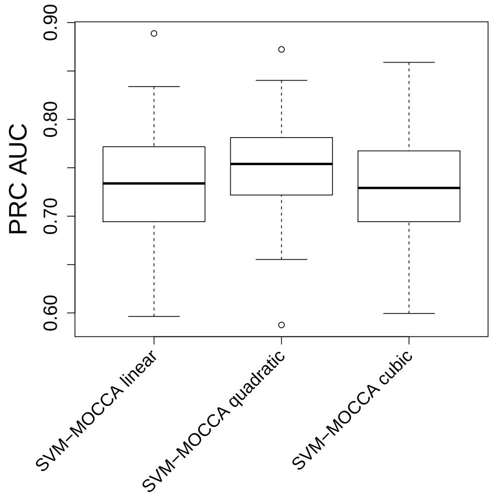

Classifier 1	Classifier 2	$p(H_0 : ROC1 <= ROC2)$	t	$p(H_0 : PRC1 <= PRC2)$	[illegible]
SVM-MOCCA linear	SVM-MOCCA linear	-	-	-	
SVM-MOCCA linear	SVM-MOCCA quadratic	1.000000E+00	-8.662432E+00	9.997814E-01	-3.771752[illegible]
SVM-MOCCA linear	SVM-MOCCA cubic	1.000000E+00	-7.669591E+00	5.388263E-02	1.638301[illegible]
SVM-MOCCA quadratic	SVM-MOCCA linear	9.442117E-12	8.662432E+00	2.186267E-04	3.771752[illegible]
SVM-MOCCA quadratic	SVM-MOCCA quadratic	-	-	-	
SVM-MOCCA quadratic	SVM-MOCCA cubic	2.253108E-03	2.977596E+00	6.274187E-12	8.780590[illegible]
SVM-MOCCA cubic	SVM-MOCCA linear	3.067293E-10	7.669591E+00	9.461174E-01	-1.638301[illegible]
SVM-MOCCA cubic	SVM-MOCCA quadratic	9.977469E-01	-2.977596E+00	1.000000E+00	-8.780590[illegible]
SVM-MOCCA cubic	SVM-MOCCA cubic	-	-	-	

SVM-MOCCA kernel functions - PRE training set: Schwartz 2010 PREs - Validation set: validation_PREsSchwartz vs. validation_D5merPREsSchwartz

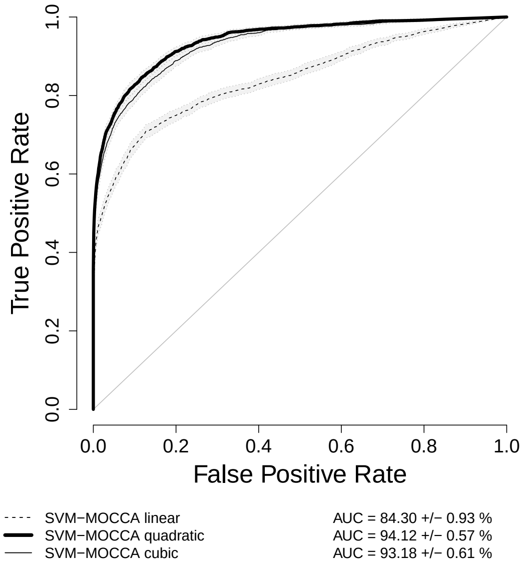

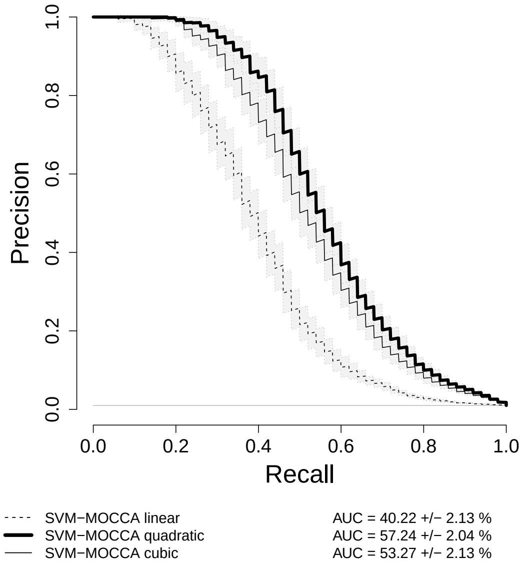

Classifier	ROC AUC
SVM-MOCCA linear	84.30 +/- 0.93 %
SVM-MOCCA quadratic	94.12 +/- 0.57 %
SVM-MOCCA cubic	93.18 +/- 0.61 %

Classifier	PRC AUC
SVM-MOCCA linear	40.22 +/- 2.13 %
SVM-MOCCA quadratic	57.24 +/- 2.04 %
SVM-MOCCA cubic	53.27 +/- 2.13 %

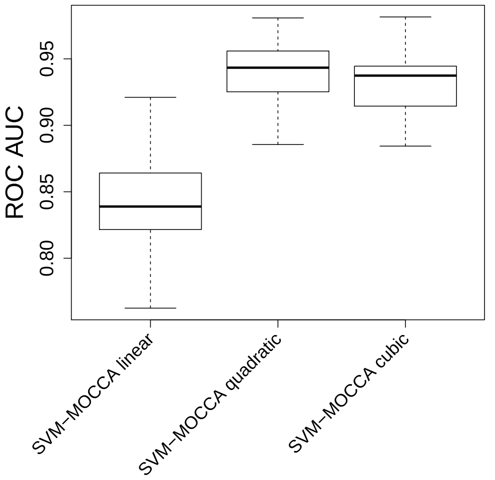

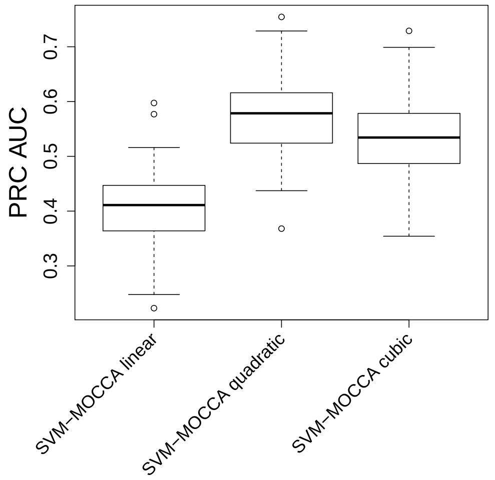

Classifier 1	Classifier 2	$p(H_0 : ROC1 <= ROC2)$	t	$p(H_0 : PRC1 <= PRC2)$	[illegible]
SVM-MOCCA linear	SVM-MOCCA linear	-	-	-	
SVM-MOCCA linear	SVM-MOCCA quadratic	1.000000E+00	-3.118331E+01	1.000000E+00	-3.174699[illegible]
SVM-MOCCA linear	SVM-MOCCA cubic	1.000000E+00	-3.105093E+01	1.000000E+00	-2.134326[illegible]
SVM-MOCCA quadratic	SVM-MOCCA linear	2.807764E-34	3.118331E+01	1.215494E-34	3.174699[illegible]
SVM-MOCCA quadratic	SVM-MOCCA quadratic	-	-	-	
SVM-MOCCA quadratic	SVM-MOCCA cubic	2.935673E-10	7.681979E+00	8.262088E-17	1.223459[illegible]
SVM-MOCCA cubic	SVM-MOCCA linear	3.424678E-34	3.105093E+01	9.201194E-27	2.134326[illegible]
SVM-MOCCA cubic	SVM-MOCCA quadratic	1.000000E+00	-7.681979E+00	1.000000E+00	-1.223459[illegible]
SVM-MOCCA cubic	SVM-MOCCA cubic	-	-	-	

CPREdictor trained with T2003 PREs versus non-PREs or dummy PREs

- - - - CPREdictor T2003
—— CPREdictor T2003 M.C.
CPREdictor T2003 w. GTGT
CPREdictor T2003 M.C. w. GTGT

Classifier	Path
CPREdictor T2003	CPREdictor_M2003_T2003_mdBetween_wmPREdictor
CPREdictor T2003 M.C.	CPREdictor_M2003_T2003Markov_mdBetween_wmPREdictor
CPREdictor T2003 w. GTGT	CPREdictor_M2003_GTGT_T2003_mdBetween_wmPREdictor
CPREdictor T2003 M.C. w. GTGT	CPREdictor_M2003_GTGT_T2003Markov_mdBetween_wmPREdictor

Classifier comparison - T2003, Markov chain controls - Validation set: validation PREsSchwartz vs. validation R5mers

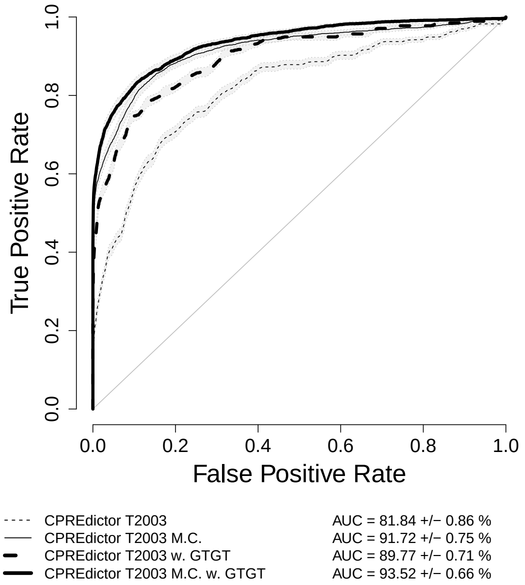

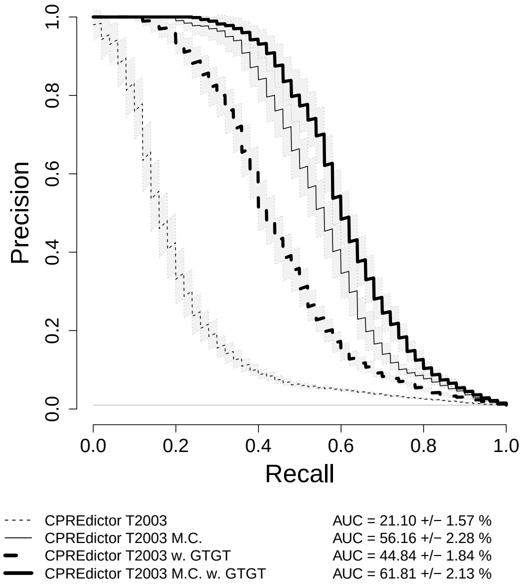

Classifier	ROC AUC
CPREdictor T2003	81.84 +/- 0.86 %
CPREdictor T2003 M.C.	91.72 +/- 0.75 %
CPREdictor T2003 w. GTGT	89.77 +/- 0.71 %
CPREdictor T2003 M.C. w. GTGT	93.52 +/- 0.66 %

Classifier	PRC AUC
CPREdictor T2003	21.10 +/- 1.57 %
CPREdictor T2003 M.C.	56.16 +/- 2.28 %
CPREdictor T2003 w. GTGT	44.84 +/- 1.84 %
CPREdictor T2003 M.C. w. GTGT	61.81 +/- 2.13 %

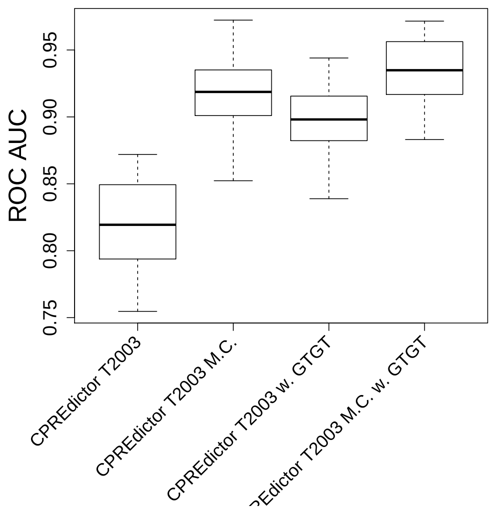

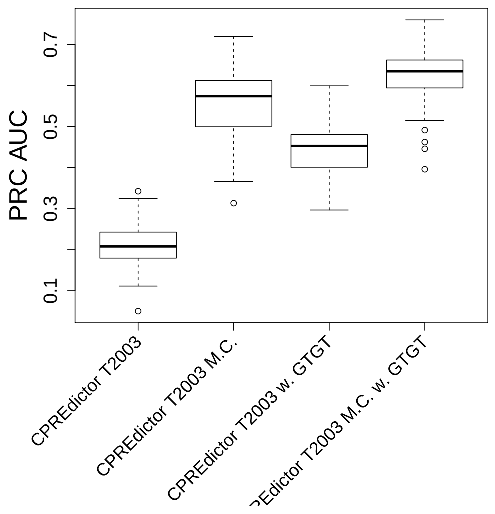

Classifier 1	Classifier 2	$p(H_0 : ROC1 <= ROC2)$	t	$p(H_0 : PRC1 <= PR$[illegible]
CPREdictor T2003	CPREdictor T2003	-	-	
CPREdictor T2003	CPREdictor T2003 M.C.	1.000000E+00	-2.430013E+01	1.000000E[illegible]
CPREdictor T2003	CPREdictor T2003 w. GTGT	1.000000E+00	-2.372866E+01	1.000000E[illegible]
CPREdictor T2003	CPREdictor T2003 M.C. w. GTGT	1.000000E+00	-2.793545E+01	1.000000E[illegible]
CPREdictor T2003 M.C.	CPREdictor T2003	2.735101E-29	2.430013E+01	8.325364E[illegible]
CPREdictor T2003 M.C.	CPREdictor T2003 M.C.	-	-	
CPREdictor T2003 M.C.	CPREdictor T2003 w. GTGT	1.160804E-05	4.676691E+00	5.380019E[illegible]
CPREdictor T2003 M.C.	CPREdictor T2003 M.C. w. GTGT	1.000000E+00	-6.717717E+00	1.000000E[illegible]
CPREdictor T2003 w. GTGT	CPREdictor T2003	8.026321E-29	2.372866E+01	1.358707E[illegible]
CPREdictor T2003 w. GTGT	CPREdictor T2003 M.C.	9.999884E-01	-4.676691E+00	1.000000E[illegible]
CPREdictor T2003 w. GTGT	CPREdictor T2003 w. GTGT	-	-	
CPREdictor T2003 w. GTGT	CPREdictor T2003 M.C. w. GTGT	1.000000E+00	-1.327194E+01	1.000000E[illegible]
CPREdictor T2003 M.C. w. GTGT	CPREdictor T2003	4.639898E-32	2.793545E+01	1.129624E[illegible]
CPREdictor T2003 M.C. w. GTGT	CPREdictor T2003 M.C.	9.081165E-09	6.717717E+00	4.454486E[illegible]
CPREdictor T2003 M.C. w. GTGT	CPREdictor T2003 w. GTGT	3.793717E-18	1.327194E+01	1.135106E[illegible]
CPREdictor T2003 M.C. w. GTGT	CPREdictor T2003 M.C. w. GTGT	-	-	

Classifier comparison - T2003, Markov chain controls - Validation set: validation PREsSchwartz vs. validation CDS

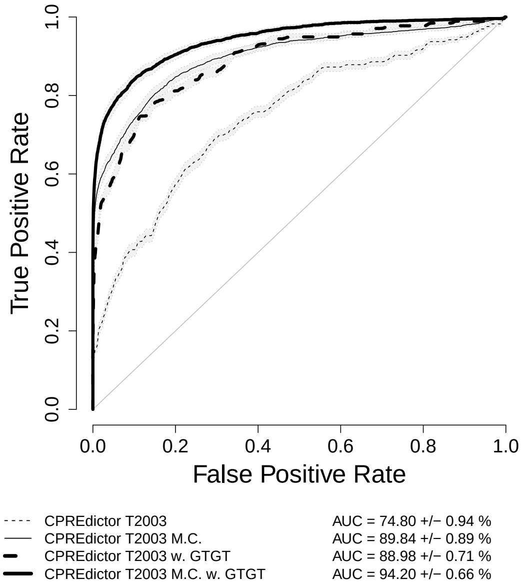

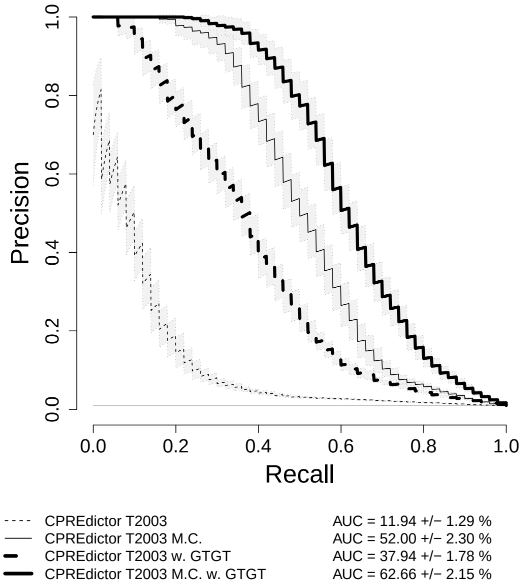

Classifier	ROC AUC
CPREdictor T2003	74.80 +/- 0.94 %
CPREdictor T2003 M.C.	89.84 +/- 0.89 %
CPREdictor T2003 w. GTGT	88.98 +/- 0.71 %
CPREdictor T2003 M.C. w. GTGT	94.20 +/- 0.66 %

Classifier	PRC AUC
CPREdictor T2003	11.94 +/- 1.29 %
CPREdictor T2003 M.C.	52.00 +/- 2.30 %
CPREdictor T2003 w. GTGT	37.94 +/- 1.78 %
CPREdictor T2003 M.C. w. GTGT	62.66 +/- 2.15 %

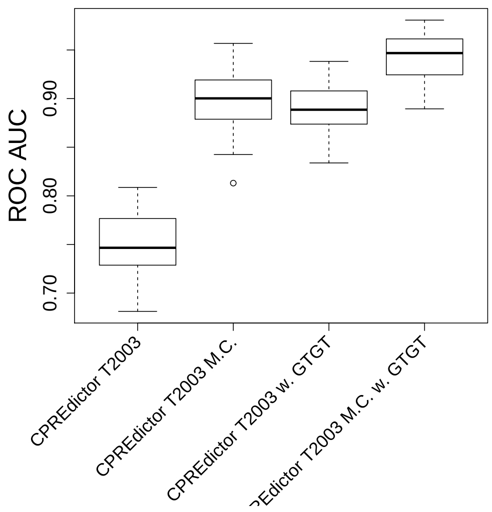

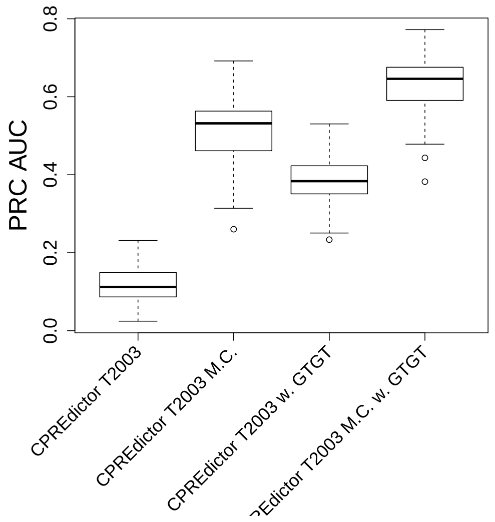

Classifier 1	Classifier 2	$p(H_0 : ROC1 <= ROC2)$	t	$p(H_0 : PRC1 <= PR$[illegible]
CPREdictor T2003	CPREdictor T2003	-	-	
CPREdictor T2003	CPREdictor T2003 M.C.	1.000000E+00	-3.241688E+01	1.000000E[illegible]
CPREdictor T2003	CPREdictor T2003 w. GTGT	1.000000E+00	-3.919928E+01	1.000000E[illegible]
CPREdictor T2003	CPREdictor T2003 M.C. w. GTGT	1.000000E+00	-4.146346E+01	1.000000E[illegible]
CPREdictor T2003 M.C.	CPREdictor T2003	4.572911E-35	3.241688E+01	3.953958E[illegible]
CPREdictor T2003 M.C.	CPREdictor T2003 M.C.	-	-	
CPREdictor T2003 M.C.	CPREdictor T2003 w. GTGT	3.425484E-02	1.862668E+00	8.312488E[illegible]
CPREdictor T2003 M.C.	CPREdictor T2003 M.C. w. GTGT	1.000000E+00	-1.411441E+01	1.000000E[illegible]
CPREdictor T2003 w. GTGT	CPREdictor T2003	5.825198E-39	3.919928E+01	1.608606E[illegible]
CPREdictor T2003 w. GTGT	CPREdictor T2003 M.C.	9.657452E-01	-1.862668E+00	1.000000E[illegible]
CPREdictor T2003 w. GTGT	CPREdictor T2003 w. GTGT	-	-	
CPREdictor T2003 w. GTGT	CPREdictor T2003 M.C. w. GTGT	1.000000E+00	-1.713008E+01	1.000000E[illegible]
CPREdictor T2003 M.C. w. GTGT	CPREdictor T2003	4.023745E-40	4.146346E+01	1.229970E[illegible]
CPREdictor T2003 M.C. w. GTGT	CPREdictor T2003 M.C.	3.433439E-19	1.411441E+01	3.776369E[illegible]
CPREdictor T2003 M.C. w. GTGT	CPREdictor T2003 w. GTGT	1.253762E-22	1.713008E+01	1.227597E[illegible]
CPREdictor T2003 M.C. w. GTGT	CPREdictor T2003 M.C. w. GTGT	-	-	

Classifier comparison - T2003, Markov chain controls - Validation set: validation PREsSchwartz vs. validation D5merPREsSchwartz

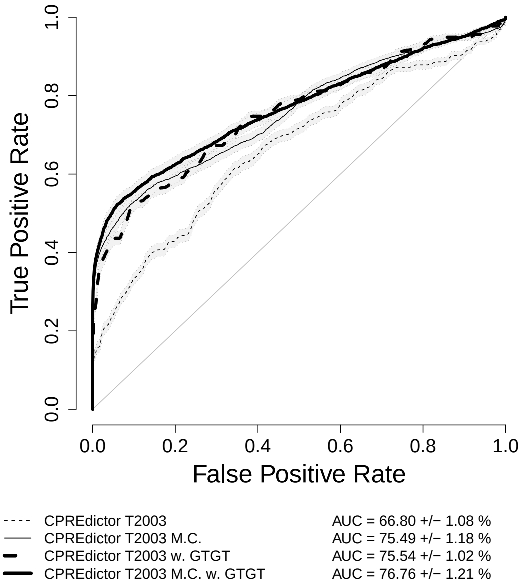

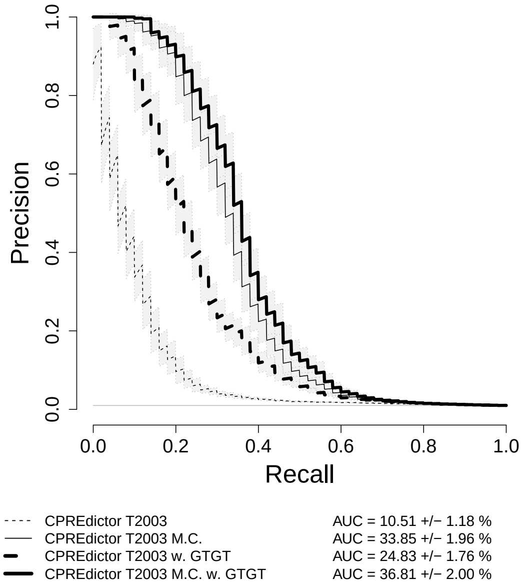

Classifier	ROC AUC
CPREdictor T2003	66.80 +/- 1.08 %
CPREdictor T2003 M.C.	75.49 +/- 1.18 %
CPREdictor T2003 w. GTGT	75.54 +/- 1.02 %
CPREdictor T2003 M.C. w. GTGT	76.76 +/- 1.21 %

Classifier	PRC AUC
CPREdictor T2003	10.51 +/- 1.18 %
CPREdictor T2003 M.C.	33.85 +/- 1.96 %
CPREdictor T2003 w. GTGT	24.83 +/- 1.76 %
CPREdictor T2003 M.C. w. GTGT	36.81 +/- 2.00 %

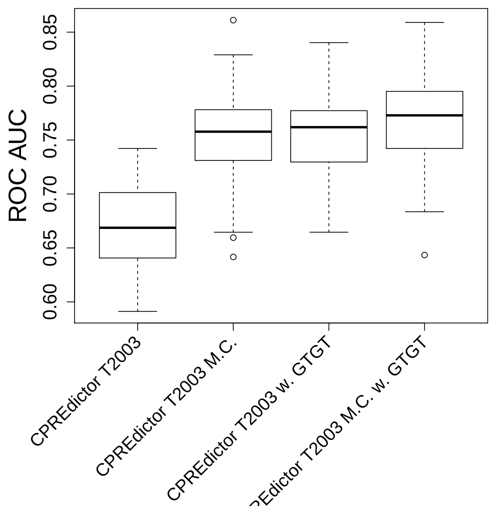

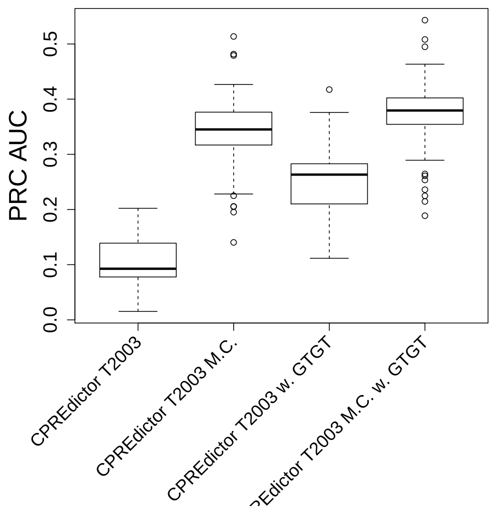

Classifier 1	Classifier 2	$p(H_0 : ROC1 <= ROC2)$	t	$p(H_0 : PRC1 <= PR$ [illegible]
CPREdictor T2003	CPREdictor T2003	-	-	
CPREdictor T2003	CPREdictor T2003 M.C.	1.000000E+00	-1.641222E+01	1.000000E- [illegible]
CPREdictor T2003	CPREdictor T2003 w. GTGT	1.000000E+00	-1.985943E+01	1.000000E- [illegible]
CPREdictor T2003	CPREdictor T2003 M.C. w. GTGT	1.000000E+00	-1.632728E+01	1.000000E- [illegible]
CPREdictor T2003 M.C.	CPREdictor T2003	7.527083E-22	1.641222E+01	2.368706E [illegible]
CPREdictor T2003 M.C.	CPREdictor T2003 M.C.	-	-	
CPREdictor T2003 M.C.	CPREdictor T2003 w. GTGT	5.365490E-01	-9.221662E-02	6.220110E [illegible]
CPREdictor T2003 M.C.	CPREdictor T2003 M.C. w. GTGT	9.997274E-01	-3.700310E+00	1.000000E- [illegible]
CPREdictor T2003 w. GTGT	CPREdictor T2003	2.194621E-25	1.985943E+01	1.022709E [illegible]
CPREdictor T2003 w. GTGT	CPREdictor T2003 M.C.	4.634510E-01	9.221662E-02	1.000000E- [illegible]
CPREdictor T2003 w. GTGT	CPREdictor T2003 w. GTGT	-	-	
CPREdictor T2003 w. GTGT	CPREdictor T2003 M.C. w. GTGT	9.967469E-01	-2.842616E+00	1.000000E- [illegible]
CPREdictor T2003 M.C. w. GTGT	CPREdictor T2003	9.338977E-22	1.632728E+01	9.159692E [illegible]
CPREdictor T2003 M.C. w. GTGT	CPREdictor T2003 M.C.	2.725854E-04	3.700310E+00	1.053632E [illegible]
CPREdictor T2003 M.C. w. GTGT	CPREdictor T2003 w. GTGT	3.253075E-03	2.842616E+00	3.957484E [illegible]
CPREdictor T2003 M.C. w. GTGT	CPREdictor T2003 M.C. w. GTGT	-	-	

T2003 PREs versus non-PREs (training set PREs: Schwartz 2010 PREs)

CPREdictor T2003
CPREdictor T2017
CPREdictor T2017 w. GTGT
SVM−MOCCA

Classifier	Path
CPREdictor T2003	CPREdictor_M2003_T2003_mdBetween_wmPREdictor
CPREdictor T2017	CPREdictor_M2003_CPPREsSchwartz_CND5merPREsSchwartz_T110_mdBetween_wmPREdictor
CPREdictor T2017 w. GTGT	CPREdictor_M2003_GTGT_CPPREsSchwartz_CND5merPREsSchwartz_T110_mdBetween_wmPREdictor
SVM-MOCCA	SVMMOCCA_kquadratic_fnOcc_fDNT_M2003_GTGT_CPPREsSchwartz_CNR5mers_CNCDS_CND5merPREsSchwartz_T110_ws3000_mdBetween_w[illegible]

T2003 tests - PRE training set: Schwartz 2010 PREs - Validation set: T2003 PREs vs. T2003 NonPREs

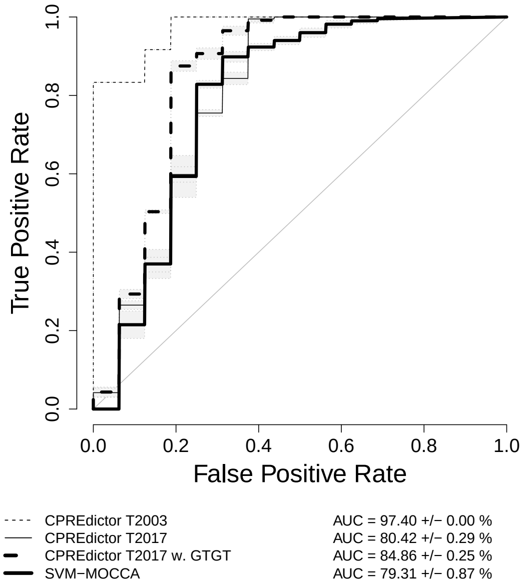

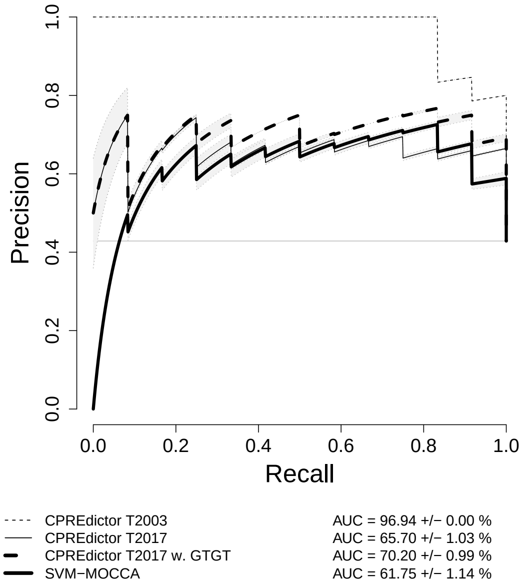

Classifier	ROC AUC
CPREdictor T2003	97.40 +/- 0.00 %
CPREdictor T2017	80.42 +/- 0.29 %
CPREdictor T2017 w. GTGT	84.86 +/- 0.25 %
SVM-MOCCA	79.31 +/- 0.87 %

Classifier	PRC AUC
CPREdictor T2003	96.94 +/- 0.00 %
CPREdictor T2017	65.70 +/- 1.03 %
CPREdictor T2017 w. GTGT	70.20 +/- 0.99 %
SVM-MOCCA	61.75 +/- 1.14 %

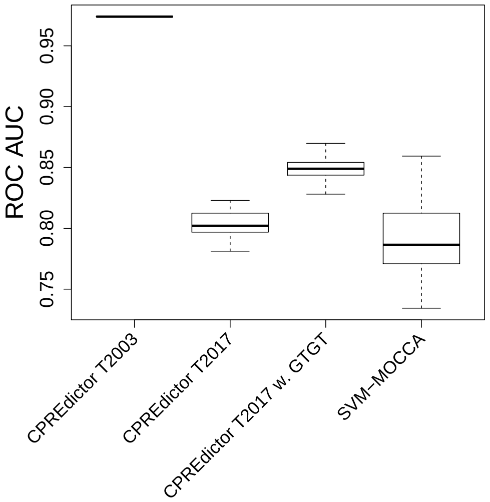

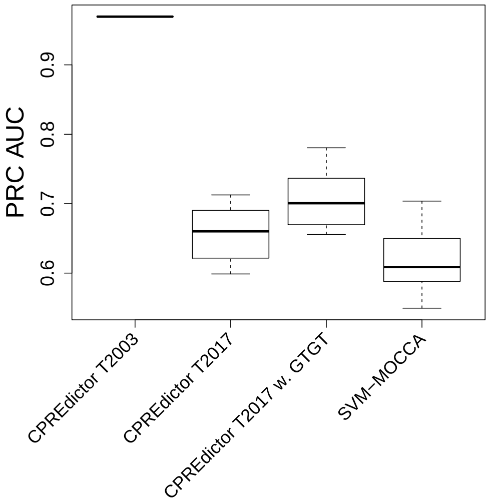

Classifier 1	Classifier 2	$p(H_0 : ROC1 <= ROC2)$	t	$p(H_0 : PRC1 <= PRC2)$	
CPREdictor T2003	CPREdictor T2003	-	-	-	
CPREdictor T2003	CPREdictor T2017	9.869524E-62	1.158510E+02	1.080615E-47	5[illegible]
CPREdictor T2003	CPREdictor T2017 w. GTGT	3.011639E-58	9.828473E+01	3.027723E-45	5[illegible]
CPREdictor T2003	SVM-MOCCA	8.958382E-40	4.077277E+01	4.335608E-48	6[illegible]
CPREdictor T2017	CPREdictor T2003	1.000000E+00	-1.158510E+02	1.000000E+00	-5[illegible]
CPREdictor T2017	CPREdictor T2017	-	-	-	
CPREdictor T2017	CPREdictor T2017 w. GTGT	1.000000E+00	-3.469722E+01	1.000000E+00	-1[illegible]
CPREdictor T2017	SVM-MOCCA	6.369973E-03	2.585796E+00	8.951263E-07	5[illegible]
CPREdictor T2017 w. GTGT	CPREdictor T2003	1.000000E+00	-9.828473E+01	1.000000E+00	-5[illegible]
CPREdictor T2017 w. GTGT	CPREdictor T2017	1.874085E-36	3.469722E+01	1.875037E-25	1[illegible]
CPREdictor T2017 w. GTGT	CPREdictor T2017 w. GTGT	-	-	-	
CPREdictor T2017 w. GTGT	SVM-MOCCA	4.044711E-18	1.324989E+01	5.067143E-16	1[illegible]
SVM-MOCCA	CPREdictor T2003	1.000000E+00	-4.077277E+01	1.000000E+00	-6[illegible]
SVM-MOCCA	CPREdictor T2017	9.936300E-01	-2.585796E+00	9.999991E-01	-5[illegible]
SVM-MOCCA	CPREdictor T2017 w. GTGT	1.000000E+00	-1.324989E+01	1.000000E+00	-1[illegible]
SVM-MOCCA	SVM-MOCCA	-	-	-	

T2003 tests - PRE training set: Schwartz 2010 PREs - Validation set: T2003 PREs vs. T2003 D5merPREs

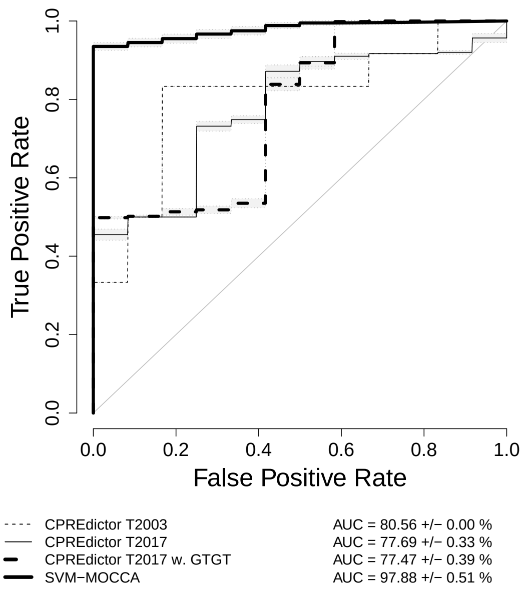

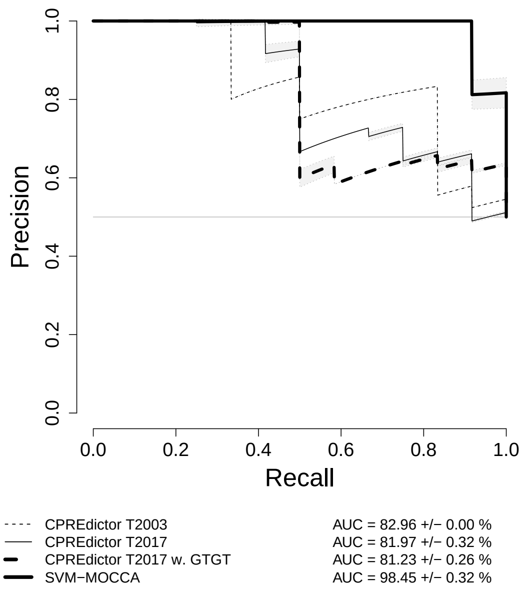

Classifier	ROC AUC
CPREdictor T2003	80.56 +/- 0.00 %
CPREdictor T2017	77.69 +/- 0.33 %
CPREdictor T2017 w. GTGT	77.47 +/- 0.39 %
SVM-MOCCA	97.88 +/- 0.51 %

Classifier	PRC AUC
CPREdictor T2003	82.96 +/- 0.00 %
CPREdictor T2017	81.97 +/- 0.32 %
CPREdictor T2017 w. GTGT	81.23 +/- 0.26 %
SVM-MOCCA	98.45 +/- 0.32 %

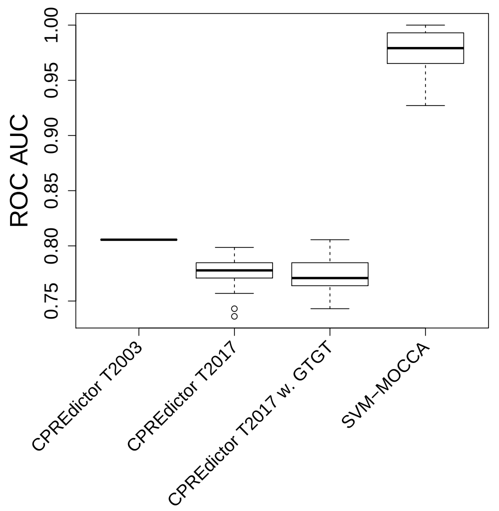

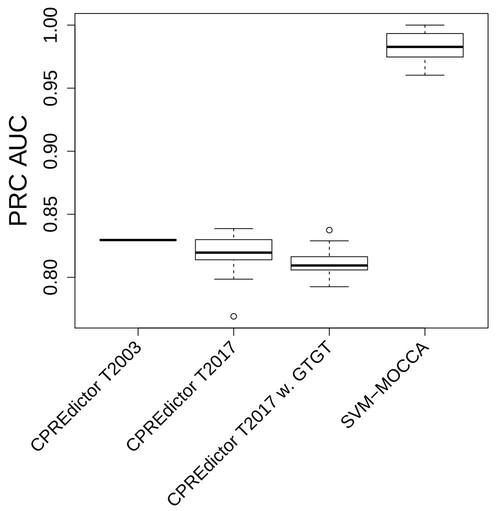

Classifier 1	Classifier 2	$p(H_0 : ROC1 <= ROC2)$	t	$p(H_0 : PRC1 <= PRC2)$	
CPREdictor T2003	CPREdictor T2003	-	-	-	
CPREdictor T2003	CPREdictor T2017	2.111240E-22	1.691910E+01	8.818024E-08	6
CPREdictor T2003	CPREdictor T2017 w. GTGT	9.064700E-21	1.545064E+01	7.515371E-18	1
CPREdictor T2003	SVM-MOCCA	1.000000E+00	-6.671816E+01	1.000000E+00	-9
CPREdictor T2017	CPREdictor T2003	1.000000E+00	-1.691910E+01	9.999999E-01	-6
CPREdictor T2017	CPREdictor T2017	-	-	-	
CPREdictor T2017	CPREdictor T2017 w. GTGT	1.754724E-01	9.417419E-01	7.790880E-06	4
CPREdictor T2017	SVM-MOCCA	1.000000E+00	-6.537007E+01	1.000000E+00	-7
CPREdictor T2017 w. GTGT	CPREdictor T2003	1.000000E+00	-1.545064E+01	1.000000E+00	-1
CPREdictor T2017 w. GTGT	CPREdictor T2017	8.245276E-01	-9.417419E-01	9.999922E-01	-4
CPREdictor T2017 w. GTGT	CPREdictor T2017 w. GTGT	-	-	-	
CPREdictor T2017 w. GTGT	SVM-MOCCA	1.000000E+00	-1.030856E+02	1.000000E+00	-1
SVM-MOCCA	CPREdictor T2003	4.583922E-50	6.671816E+01	9.034312E-58	9
SVM-MOCCA	CPREdictor T2017	1.232811E-49	6.537007E+01	1.061425E-53	7
SVM-MOCCA	CPREdictor T2017 w. GTGT	2.942469E-59	1.030856E+02	2.558337E-64	1
SVM-MOCCA	SVM-MOCCA	-	-	-	

Classifier comparison, coloured (training set PREs: Schwartz 2010 PREs)

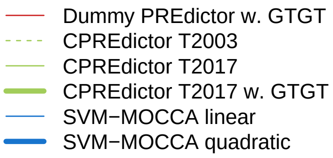

Classifier	Path
Dummy PREdictor w. GTGT	DummyPREdictor_M2003_GTGT_T2003_mdBetween_wmPREdictor
CPREdictor T2003	CPREdictor_M2003_T2003_mdBetween_wmPREdictor
CPREdictor T2017	CPREdictor_M2003_CPPREsSchwartz_CND5merPREsSchwartz_T110_mdBetween_wmPREdictor
CPREdictor T2017 w. GTGT	CPREdictor_M2003_GTGT_CPPREsSchwartz_CND5merPREsSchwartz_T110_mdBetween_wmPREdictor
SVM-MOCCA linear	SVMMOCCA_klinear_fnOcc_fDNT_M2003_GTGT_CPPREsSchwartz_CNR5mers_CNCDS_CND5merPREsSchwartz_T110_ws3000_mdBetween_wmP[illegible]
SVM-MOCCA quadratic	SVMMOCCA_kquadratic_fnOcc_fDNT_M2003_GTGT_CPPREsSchwartz_CNR5mers_CNCDS_CND5merPREsSchwartz_T110_ws3000_mdBetween_w[illegible]

Classifier comparison, coloured - training set PREs: Schwartz 2010 PREs - Validation set: validation_PREsSchwartz vs. validation_R5mers

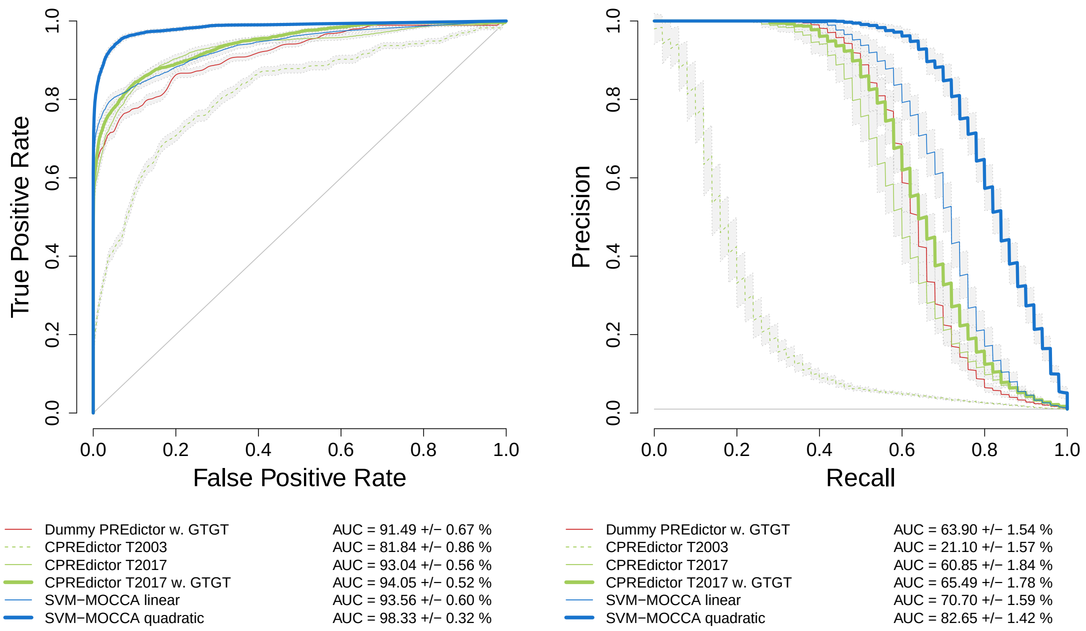

Classifier	ROC AUC
Dummy PREdictor w. GTGT	91.49 +/- 0.67 %
CPREdictor T2003	81.84 +/- 0.86 %
CPREdictor T2017	93.04 +/- 0.56 %
CPREdictor T2017 w. GTGT	94.05 +/- 0.52 %
SVM-MOCCA linear	93.56 +/- 0.60 %
SVM-MOCCA quadratic	98.33 +/- 0.32 %

Classifier	PRC AUC
Dummy PREdictor w. GTGT	63.90 +/- 1.54 %
CPREdictor T2003	21.10 +/- 1.57 %
CPREdictor T2017	60.85 +/- 1.84 %
CPREdictor T2017 w. GTGT	65.49 +/- 1.78 %
SVM-MOCCA linear	70.70 +/- 1.59 %
SVM-MOCCA quadratic	82.65 +/- 1.42 %

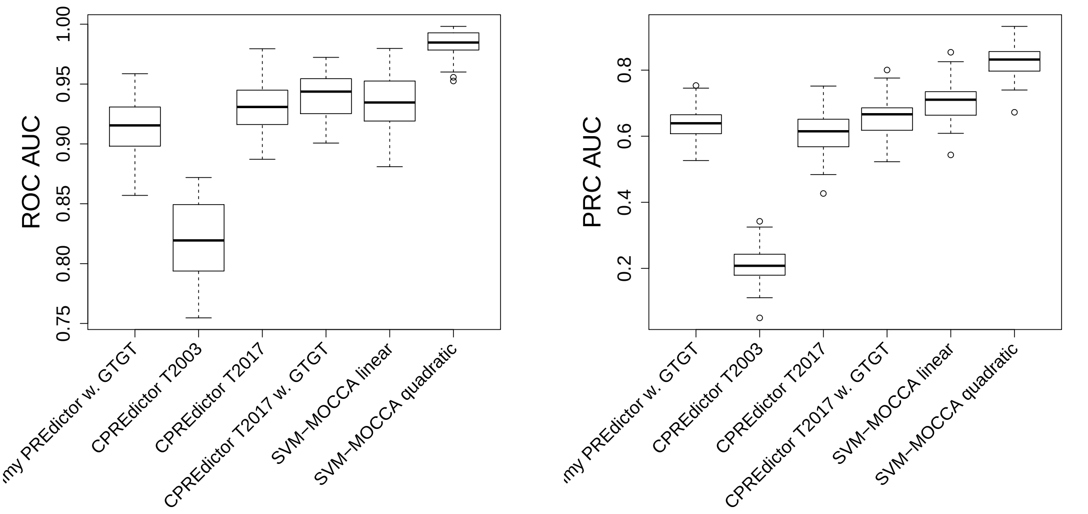

Classifier 1	Classifier 2	$p(H_0 : ROC1 <= ROC2)$	t	$p(H_0 : PRC1 <= PRC2)$	
Dummy PREdictor w. GTGT	Dummy PREdictor w. GTGT	-	-	-	
Dummy PREdictor w. GTGT	CPREdictor T2003	2.780187E-29	2.429137E+01	6.652054E-47	5
Dummy PREdictor w. GTGT	CPREdictor T2017	9.999921E-01	-4.792053E+00	2.515157E-05	4
Dummy PREdictor w. GTGT	CPREdictor T2017 w. GTGT	1.000000E+00	-1.467609E+01	9.977374E-01	-2
Dummy PREdictor w. GTGT	SVM-MOCCA linear	1.000000E+00	-6.313961E+00	1.000000E+00	-1
Dummy PREdictor w. GTGT	SVM-MOCCA quadratic	1.000000E+00	-1.968374E+01	1.000000E+00	-2
CPREdictor T2003	Dummy PREdictor w. GTGT	1.000000E+00	-2.429137E+01	1.000000E+00	-5
CPREdictor T2003	CPREdictor T2003	-	-	-	
CPREdictor T2003	CPREdictor T2017	1.000000E+00	-2.669258E+01	1.000000E+00	-5
CPREdictor T2003	CPREdictor T2017 w. GTGT	1.000000E+00	-2.760384E+01	1.000000E+00	-5
CPREdictor T2003	SVM-MOCCA linear	1.000000E+00	-2.561996E+01	1.000000E+00	-5
CPREdictor T2003	SVM-MOCCA quadratic	1.000000E+00	-3.508461E+01	1.000000E+00	-6
CPREdictor T2017	Dummy PREdictor w. GTGT	7.861661E-06	4.792053E+00	9.999748E-01	-4
CPREdictor T2017	CPREdictor T2003	3.770779E-31	2.669258E+01	5.886922E-45	5
CPREdictor T2017	CPREdictor T2017	-	-	-	
CPREdictor T2017	CPREdictor T2017 w. GTGT	9.997900E-01	-3.784664E+00	1.000000E+00	-1
CPREdictor T2017	SVM-MOCCA linear	9.130674E-01	-1.379954E+00	1.000000E+00	-1
CPREdictor T2017	SVM-MOCCA quadratic	1.000000E+00	-1.607915E+01	1.000000E+00	-2
CPREdictor T2017 w. GTGT	Dummy PREdictor w. GTGT	7.262424E-20	1.467609E+01	2.262641E-03	2
CPREdictor T2017 w. GTGT	CPREdictor T2003	8.049101E-32	2.760384E+01	5.393157E-45	5
CPREdictor T2017 w. GTGT	CPREdictor T2017	2.100401E-04	3.784664E+00	1.258567E-14	1
CPREdictor T2017 w. GTGT	CPREdictor T2017 w. GTGT	-	-	-	
CPREdictor T2017 w. GTGT	SVM-MOCCA linear	4.213860E-02	1.762174E+00	1.000000E+00	-9
CPREdictor T2017 w. GTGT	SVM-MOCCA quadratic	1.000000E+00	-1.567812E+01	1.000000E+00	-1
SVM-MOCCA linear	Dummy PREdictor w. GTGT	3.832733E-08	6.313961E+00	1.020061E-17	1
SVM-MOCCA linear	CPREdictor T2003	2.466046E-30	2.561996E+01	4.814939E-47	5
SVM-MOCCA linear	CPREdictor T2017	8.693259E-02	1.379954E+00	1.195940E-18	1
SVM-MOCCA linear	CPREdictor T2017 w. GTGT	9.578614E-01	-1.762174E+00	1.207380E-12	9
SVM-MOCCA linear	SVM-MOCCA linear	-	-	-	
SVM-MOCCA linear	SVM-MOCCA quadratic	1.000000E+00	-1.771821E+01	1.000000E+00	-2
SVM-MOCCA quadratic	Dummy PREdictor w. GTGT	3.234484E-25	1.968374E+01	4.344156E-31	2
SVM-MOCCA quadratic	CPREdictor T2003	1.110388E-36	3.508461E+01	4.613400E-50	6
SVM-MOCCA quadratic	CPREdictor T2017	1.761682E-21	1.607915E+01	3.061086E-29	2
SVM-MOCCA quadratic	CPREdictor T2017 w. GTGT	4.984804E-21	1.567812E+01	2.061671E-25	1
SVM-MOCCA quadratic	SVM-MOCCA linear	3.004512E-23	1.771821E+01	4.909801E-26	2
SVM-MOCCA quadratic	SVM-MOCCA quadratic	-	-	-	

Classifier comparison, coloured - training set PREs: Schwartz 2010 PREs - Validation set: validation_PREsSchwartz vs. validation_CDS

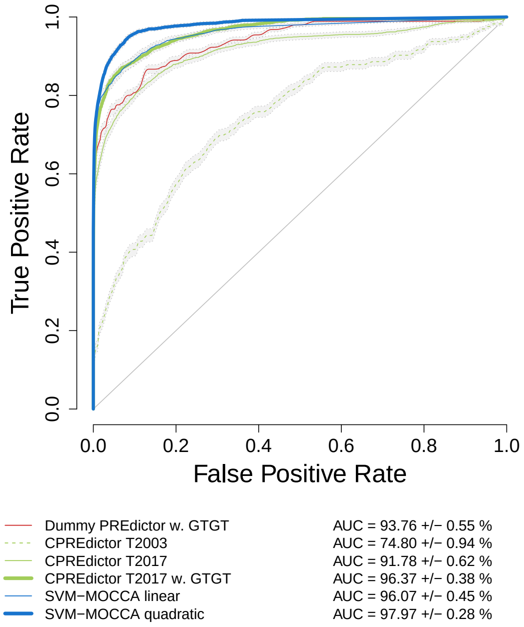

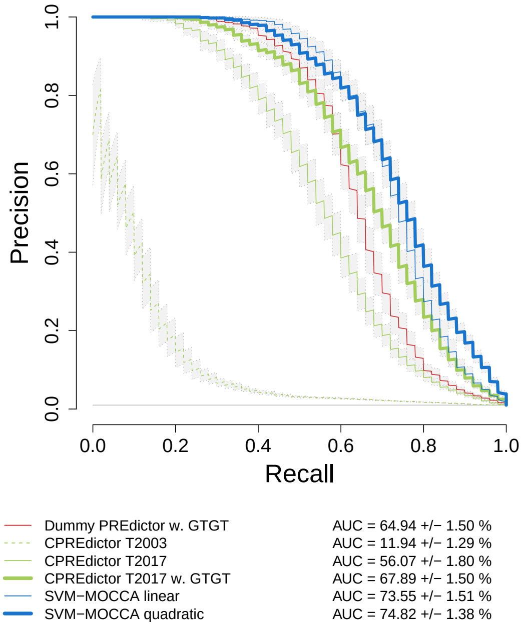

Classifier	ROC AUC
Dummy PREdictor w. GTGT	93.76 +/- 0.55 %
CPREdictor T2003	74.80 +/- 0.94 %
CPREdictor T2017	91.78 +/- 0.62 %
CPREdictor T2017 w. GTGT	96.37 +/- 0.38 %
SVM-MOCCA linear	96.07 +/- 0.45 %
SVM-MOCCA quadratic	97.97 +/- 0.28 %

Classifier	PRC AUC
Dummy PREdictor w. GTGT	64.94 +/- 1.50 %
CPREdictor T2003	11.94 +/- 1.29 %
CPREdictor T2017	56.07 +/- 1.80 %
CPREdictor T2017 w. GTGT	67.89 +/- 1.50 %
SVM-MOCCA linear	73.55 +/- 1.51 %
SVM-MOCCA quadratic	74.82 +/- 1.38 %

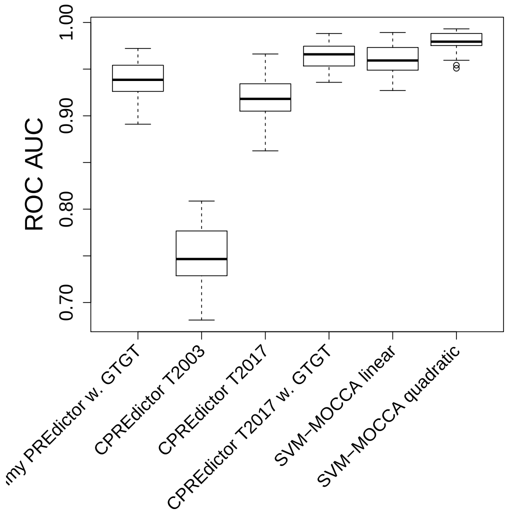

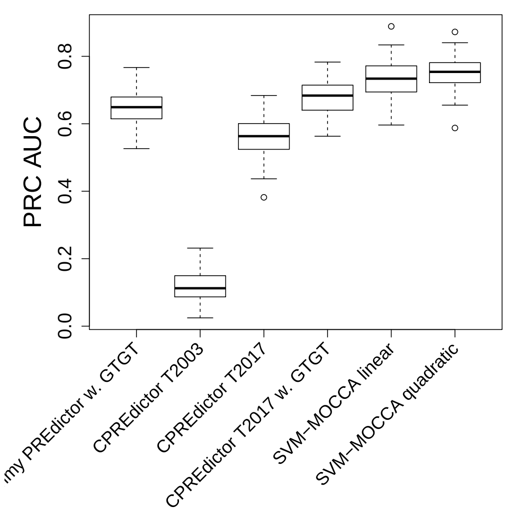

Classifier 1	Classifier 2	$p(H_0 : ROC1 <= ROC2)$	t	$p(H_0 : PRC1 <= PRC2)$	
Dummy PREdictor w. GTGT	Dummy PREdictor w. GTGT	-	-	-	
Dummy PREdictor w. GTGT	CPREdictor T2003	1.349215E-41	4.451863E+01	2.452816E-52	7[illegible]
Dummy PREdictor w. GTGT	CPREdictor T2017	2.565824E-08	6.426571E+00	3.945098E-19	1[illegible]
Dummy PREdictor w. GTGT	CPREdictor T2017 w. GTGT	1.000000E+00	-1.828423E+01	1.000000E+00	-7[illegible]
Dummy PREdictor w. GTGT	SVM-MOCCA linear	1.000000E+00	-8.465319E+00	1.000000E+00	-1[illegible]
Dummy PREdictor w. GTGT	SVM-MOCCA quadratic	1.000000E+00	-1.518366E+01	1.000000E+00	-2[illegible]
CPREdictor T2003	Dummy PREdictor w. GTGT	1.000000E+00	-4.451863E+01	1.000000E+00	-7[illegible]
CPREdictor T2003	CPREdictor T2003	-	-	-	
CPREdictor T2003	CPREdictor T2017	1.000000E+00	-3.732274E+01	1.000000E+00	-5[illegible]
CPREdictor T2003	CPREdictor T2017 w. GTGT	1.000000E+00	-4.607221E+01	1.000000E+00	-7[illegible]
CPREdictor T2003	SVM-MOCCA linear	1.000000E+00	-4.713404E+01	1.000000E+00	-7[illegible]
CPREdictor T2003	SVM-MOCCA quadratic	1.000000E+00	-4.553518E+01	1.000000E+00	-8[illegible]
CPREdictor T2017	Dummy PREdictor w. GTGT	1.000000E+00	-6.426571E+00	1.000000E+00	-1[illegible]
CPREdictor T2017	CPREdictor T2003	5.971102E-38	3.732274E+01	1.469455E-47	5[illegible]
CPREdictor T2017	CPREdictor T2017	-	-	-	
CPREdictor T2017	CPREdictor T2017 w. GTGT	1.000000E+00	-1.753386E+01	1.000000E+00	-2[illegible]
CPREdictor T2017	SVM-MOCCA linear	1.000000E+00	-1.230758E+01	1.000000E+00	-2[illegible]
CPREdictor T2017	SVM-MOCCA quadratic	1.000000E+00	-1.848367E+01	1.000000E+00	-2[illegible]
CPREdictor T2017 w. GTGT	Dummy PREdictor w. GTGT	7.848617E-24	1.828423E+01	1.327323E-09	7[illegible]
CPREdictor T2017 w. GTGT	CPREdictor T2003	2.611054E-42	4.607221E+01	1.875927E-53	7[illegible]
CPREdictor T2017 w. GTGT	CPREdictor T2017	4.683991E-23	1.753386E+01	6.043936E-31	2[illegible]
CPREdictor T2017 w. GTGT	CPREdictor T2017 w. GTGT	-	-	-	
CPREdictor T2017 w. GTGT	SVM-MOCCA linear	9.971664E-02	1.300735E+00	1.000000E+00	-1[illegible]
CPREdictor T2017 w. GTGT	SVM-MOCCA quadratic	1.000000E+00	-7.478324E+00	1.000000E+00	-1[illegible]
SVM-MOCCA linear	Dummy PREdictor w. GTGT	1.872625E-11	8.465319E+00	3.095786E-23	1[illegible]
SVM-MOCCA linear	CPREdictor T2003	8.758373E-43	4.713404E+01	1.565743E-52	7[illegible]
SVM-MOCCA linear	CPREdictor T2017	6.621999E-17	1.230758E+01	7.816657E-28	2[illegible]
SVM-MOCCA linear	CPREdictor T2017 w. GTGT	9.002834E-01	-1.300735E+00	5.019437E-15	1[illegible]
SVM-MOCCA linear	SVM-MOCCA linear	-	-	-	
SVM-MOCCA linear	SVM-MOCCA quadratic	1.000000E+00	-8.662432E+00	9.997814E-01	-3[illegible]
SVM-MOCCA quadratic	Dummy PREdictor w. GTGT	1.842680E-20	1.518366E+01	2.616900E-28	2[illegible]
SVM-MOCCA quadratic	CPREdictor T2003	4.578701E-42	4.553518E+01	2.415136E-55	8[illegible]
SVM-MOCCA quadratic	CPREdictor T2017	4.927108E-24	1.848367E+01	2.443005E-32	2[illegible]
SVM-MOCCA quadratic	CPREdictor T2017 w. GTGT	6.043538E-10	7.478324E+00	8.787213E-19	1[illegible]
SVM-MOCCA quadratic	SVM-MOCCA linear	9.442117E-12	8.662432E+00	2.186267E-04	3[illegible]
SVM-MOCCA quadratic	SVM-MOCCA quadratic	-	-	-	

Classifier comparison, coloured - training set PREs: Schwartz 2010 PREs - Validation set: validation_PREsSchwartz vs. validation_D5merPREsSchwartz

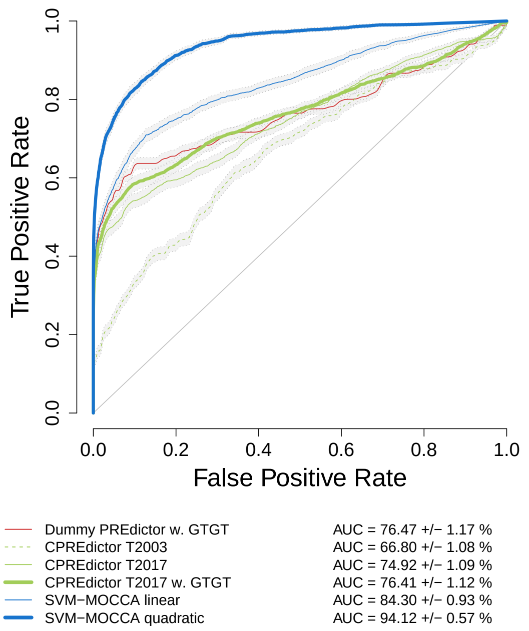

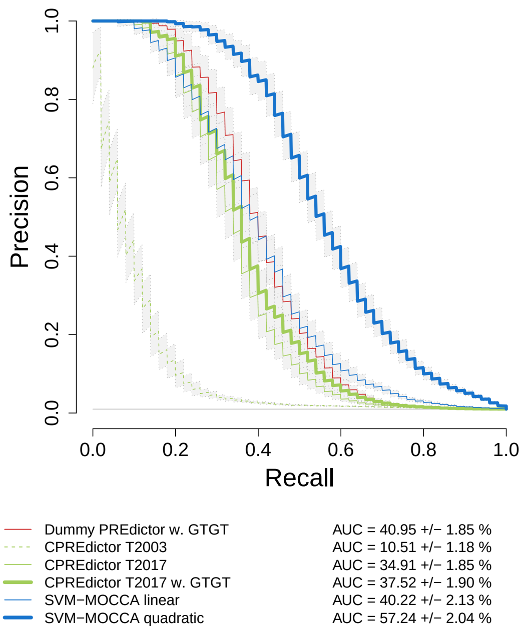

Classifier	ROC AUC
Dummy PREdictor w. GTGT	76.47 +/- 1.17 %
CPREdictor T2003	66.80 +/- 1.08 %
CPREdictor T2017	74.92 +/- 1.09 %
CPREdictor T2017 w. GTGT	76.41 +/- 1.12 %
SVM-MOCCA linear	84.30 +/- 0.93 %
SVM-MOCCA quadratic	94.12 +/- 0.57 %

Classifier	PRC AUC
Dummy PREdictor w. GTGT	40.95 +/- 1.85 %
CPREdictor T2003	10.51 +/- 1.18 %
CPREdictor T2017	34.91 +/- 1.85 %
CPREdictor T2017 w. GTGT	37.52 +/- 1.90 %
SVM-MOCCA linear	40.22 +/- 2.13 %
SVM-MOCCA quadratic	57.24 +/- 2.04 %

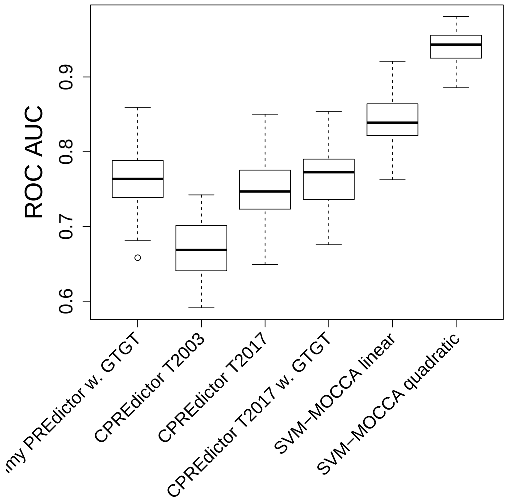

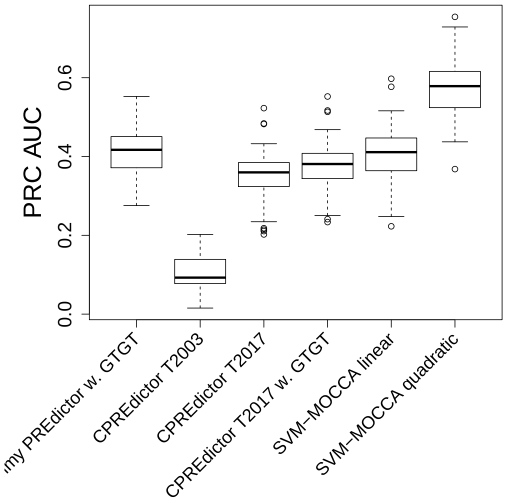

Classifier 1	Classifier 2	$p(H_0 : ROC1 <= ROC2)$	t	$p(H_0 : PRC1 <= PRC2)$	
Dummy PREdictor w. GTGT	Dummy PREdictor w. GTGT	-	-	-	
Dummy PREdictor w. GTGT	CPREdictor T2003	4.166453E-23	1.758233E+01	1.154506E-37	3[illegible]
Dummy PREdictor w. GTGT	CPREdictor T2017	8.564522E-04	3.318416E+00	4.532895E-17	1[illegible]
Dummy PREdictor w. GTGT	CPREdictor T2017 w. GTGT	4.103357E-01	2.279011E-01	4.827804E-11	8[illegible]
Dummy PREdictor w. GTGT	SVM-MOCCA linear	1.000000E+00	-1.801492E+01	9.352137E-02	1[illegible]
Dummy PREdictor w. GTGT	SVM-MOCCA quadratic	1.000000E+00	-3.442788E+01	1.000000E+00	-2[illegible]
CPREdictor T2003	Dummy PREdictor w. GTGT	1.000000E+00	-1.758233E+01	1.000000E+00	-3[illegible]
CPREdictor T2003	CPREdictor T2003	-	-	-	
CPREdictor T2003	CPREdictor T2017	1.000000E+00	-1.357770E+01	1.000000E+00	-2[illegible]
CPREdictor T2003	CPREdictor T2017 w. GTGT	1.000000E+00	-1.567490E+01	1.000000E+00	-3[illegible]
CPREdictor T2003	SVM-MOCCA linear	1.000000E+00	-2.917760E+01	1.000000E+00	-2[illegible]
CPREdictor T2003	SVM-MOCCA quadratic	1.000000E+00	-4.680712E+01	1.000000E+00	-4[illegible]
CPREdictor T2017	Dummy PREdictor w. GTGT	9.991435E-01	-3.318416E+00	1.000000E+00	-1[illegible]
CPREdictor T2017	CPREdictor T2003	1.570290E-18	1.357770E+01	4.664484E-33	2[illegible]
CPREdictor T2017	CPREdictor T2017	-	-	-	
CPREdictor T2017	CPREdictor T2017 w. GTGT	9.999424E-01	-4.192038E+00	1.000000E+00	-1[illegible]
CPREdictor T2017	SVM-MOCCA linear	1.000000E+00	-1.774064E+01	1.000000E+00	-1[illegible]
CPREdictor T2017	SVM-MOCCA quadratic	1.000000E+00	-3.672911E+01	1.000000E+00	-3[illegible]
CPREdictor T2017 w. GTGT	Dummy PREdictor w. GTGT	5.896643E-01	-2.279011E-01	1.000000E+00	-8[illegible]
CPREdictor T2017 w. GTGT	CPREdictor T2003	5.026969E-21	1.567490E+01	2.984238E-34	3[illegible]
CPREdictor T2017 w. GTGT	CPREdictor T2017	5.761909E-05	4.192038E+00	1.188161E-15	1[illegible]
CPREdictor T2017 w. GTGT	CPREdictor T2017 w. GTGT	-	-	-	
CPREdictor T2017 w. GTGT	SVM-MOCCA linear	1.000000E+00	-1.834856E+01	9.999966E-01	-5[illegible]
CPREdictor T2017 w. GTGT	SVM-MOCCA quadratic	1.000000E+00	-3.549485E+01	1.000000E+00	-2[illegible]
SVM-MOCCA linear	Dummy PREdictor w. GTGT	1.480730E-23	1.801492E+01	9.064786E-01	-1[illegible]
SVM-MOCCA linear	CPREdictor T2003	6.197191E-33	2.917760E+01	6.748490E-33	2[illegible]
SVM-MOCCA linear	CPREdictor T2017	2.847159E-23	1.774064E+01	5.777162E-14	1[illegible]
SVM-MOCCA linear	CPREdictor T2017 w. GTGT	6.751507E-24	1.834856E+01	3.412952E-06	5[illegible]
SVM-MOCCA linear	SVM-MOCCA linear	-	-	-	
SVM-MOCCA linear	SVM-MOCCA quadratic	1.000000E+00	-3.118331E+01	1.000000E+00	-3[illegible]
SVM-MOCCA quadratic	Dummy PREdictor w. GTGT	2.705340E-36	3.442788E+01	2.477113E-30	2[illegible]
SVM-MOCCA quadratic	CPREdictor T2003	1.222876E-42	4.680712E+01	2.043927E-43	4[illegible]
SVM-MOCCA quadratic	CPREdictor T2017	1.275574E-37	3.672911E+01	1.116145E-35	3[illegible]
SVM-MOCCA quadratic	CPREdictor T2017 w. GTGT	6.416161E-37	3.549485E+01	6.702903E-32	2[illegible]
SVM-MOCCA quadratic	SVM-MOCCA linear	2.807764E-34	3.118331E+01	1.215494E-34	3[illegible]
SVM-MOCCA quadratic	SVM-MOCCA quadratic	-	-	-	

Training set PREs: Schwartz 2010 PREs

Classifier comparison (training set PREs: Schwartz 2010 PREs)

CPREdictor T2003
CPREdictor T2017
CPREdictor T2017 w. GTGT
Dummy PREdictor w. GTGT
SVM−MOCCA

Classifier	Path
CPREdictor T2003	CPREdictor_M2003_T2003_mdBetween_wmPREdictor
CPREdictor T2017	CPREdictor_M2003_CPPREsSchwartz_CND5merPREsSchwartz_T110_mdBetween_wmPREdictor
CPREdictor T2017 w. GTGT	CPREdictor_M2003_GTGT_CPPREsSchwartz_CND5merPREsSchwartz_T110_mdBetween_wmPREdictor
Dummy PREdictor w. GTGT	DummyPREdictor_M2003_GTGT_T2003_mdBetween_wmPREdictor
SVM-MOCCA	SVMMOCCA_kquadratic_fnOcc_fDNT_M2003_GTGT_CPPREsSchwartz_CNR5mers_CNCDS_CND5merPREsSchwartz_T110_ws3000_mdBetween_w

Classifier comparison - training set PREs: Schwartz 2010 PREs - Validation set: validation_PREsSchwartz vs. validation_R5mers

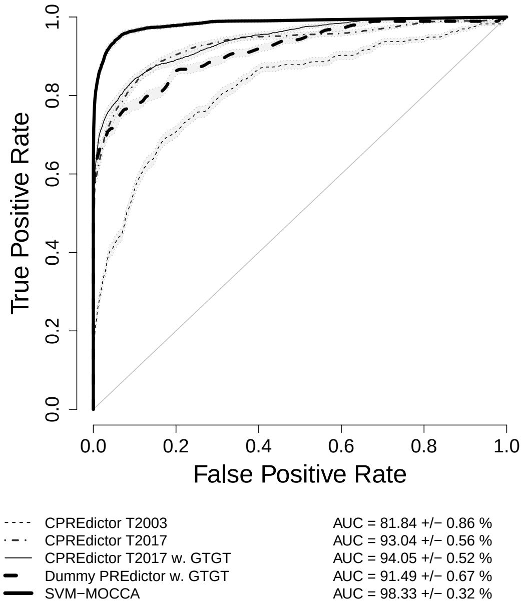

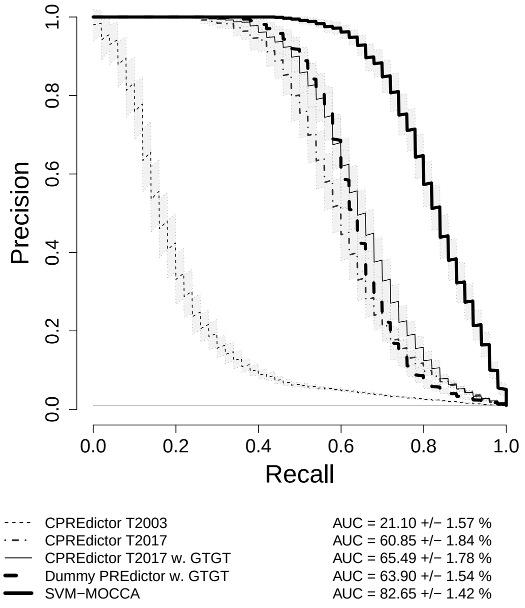

Classifier	ROC AUC
CPREdictor T2003	81.84 +/- 0.86 %
CPREdictor T2017	93.04 +/- 0.56 %
CPREdictor T2017 w. GTGT	94.05 +/- 0.52 %
Dummy PREdictor w. GTGT	91.49 +/- 0.67 %
SVM-MOCCA	98.33 +/- 0.32 %

Classifier	PRC AUC
CPREdictor T2003	21.10 +/- 1.57 %
CPREdictor T2017	60.85 +/- 1.84 %
CPREdictor T2017 w. GTGT	65.49 +/- 1.78 %
Dummy PREdictor w. GTGT	63.90 +/- 1.54 %
SVM-MOCCA	82.65 +/- 1.42 %

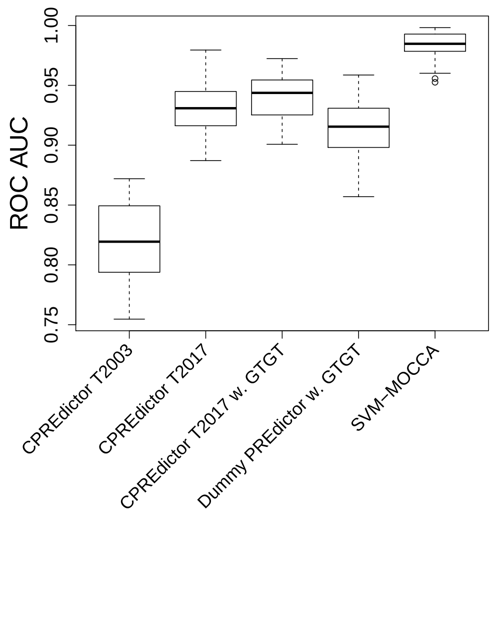

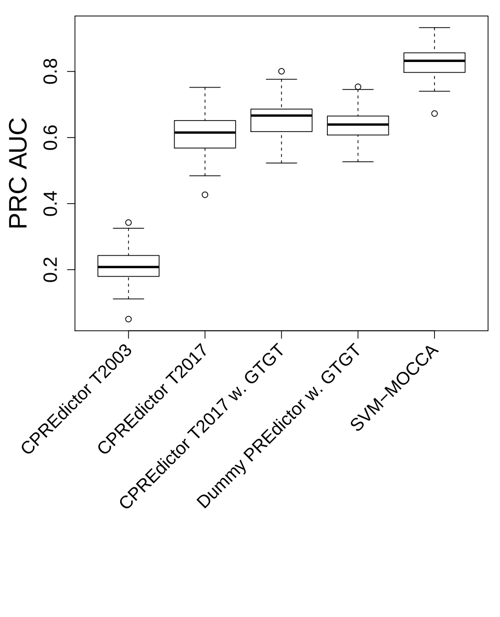

Classifier 1	Classifier 2	$p(H_0 : ROC1 <= ROC2)$	t	$p(H_0 : PRC1 <= PRC2)$	
CPREdictor T2003	CPREdictor T2003	-	-	-	
CPREdictor T2003	CPREdictor T2017	1.000000E+00	-2.669258E+01	1.000000E+00	-5[illegible]
CPREdictor T2003	CPREdictor T2017 w. GTGT	1.000000E+00	-2.760384E+01	1.000000E+00	-5[illegible]
CPREdictor T2003	Dummy PREdictor w. GTGT	1.000000E+00	-2.429137E+01	1.000000E+00	-5[illegible]
CPREdictor T2003	SVM-MOCCA	1.000000E+00	-3.508461E+01	1.000000E+00	-6[illegible]
CPREdictor T2017	CPREdictor T2003	3.770779E-31	2.669258E+01	5.886922E-45	5[illegible]
CPREdictor T2017	CPREdictor T2017	-	-	-	
CPREdictor T2017	CPREdictor T2017 w. GTGT	9.997900E-01	-3.784664E+00	1.000000E+00	-1[illegible]
CPREdictor T2017	Dummy PREdictor w. GTGT	7.861661E-06	4.792053E+00	9.999748E-01	-4[illegible]
CPREdictor T2017	SVM-MOCCA	1.000000E+00	-1.607915E+01	1.000000E+00	-2[illegible]
CPREdictor T2017 w. GTGT	CPREdictor T2003	8.049101E-32	2.760384E+01	5.393157E-45	5[illegible]
CPREdictor T2017 w. GTGT	CPREdictor T2017	2.100401E-04	3.784664E+00	1.258567E-14	1[illegible]
CPREdictor T2017 w. GTGT	CPREdictor T2017 w. GTGT	-	-	-	
CPREdictor T2017 w. GTGT	Dummy PREdictor w. GTGT	7.262424E-20	1.467609E+01	2.262641E-03	2[illegible]
CPREdictor T2017 w. GTGT	SVM-MOCCA	1.000000E+00	-1.567812E+01	1.000000E+00	-1[illegible]
Dummy PREdictor w. GTGT	CPREdictor T2003	2.780187E-29	2.429137E+01	6.652054E-47	5[illegible]
Dummy PREdictor w. GTGT	CPREdictor T2017	9.999921E-01	-4.792053E+00	2.515157E-05	4[illegible]
Dummy PREdictor w. GTGT	CPREdictor T2017 w. GTGT	1.000000E+00	-1.467609E+01	9.977374E-01	-2[illegible]
Dummy PREdictor w. GTGT	Dummy PREdictor w. GTGT	-	-	-	
Dummy PREdictor w. GTGT	SVM-MOCCA	1.000000E+00	-1.968374E+01	1.000000E+00	-2[illegible]
SVM-MOCCA	CPREdictor T2003	1.110388E-36	3.508461E+01	4.613400E-50	6[illegible]
SVM-MOCCA	CPREdictor T2017	1.761682E-21	1.607915E+01	3.061086E-29	2[illegible]
SVM-MOCCA	CPREdictor T2017 w. GTGT	4.984804E-21	1.567812E+01	2.061671E-25	1[illegible]
SVM-MOCCA	Dummy PREdictor w. GTGT	3.234484E-25	1.968374E+01	4.344156E-31	2[illegible]
SVM-MOCCA	SVM-MOCCA	-	-	-	

Classifier comparison - training set PREs: Schwartz 2010 PREs - Validation set: validation_PREsSchwartz vs. validation_CDS

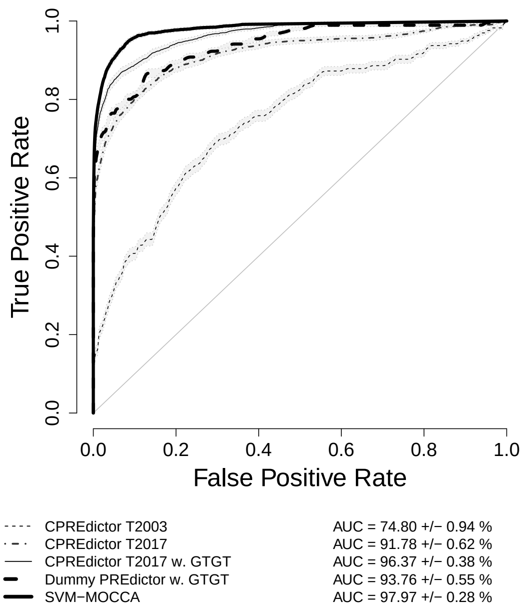

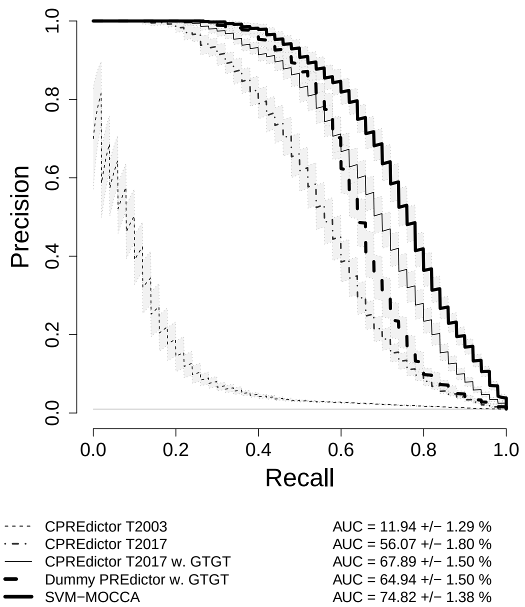

Classifier	ROC AUC
CPREdictor T2003	74.80 +/- 0.94 %
CPREdictor T2017	91.78 +/- 0.62 %
CPREdictor T2017 w. GTGT	96.37 +/- 0.38 %
Dummy PREdictor w. GTGT	93.76 +/- 0.55 %
SVM-MOCCA	97.97 +/- 0.28 %

Classifier	PRC AUC
CPREdictor T2003	11.94 +/- 1.29 %
CPREdictor T2017	56.07 +/- 1.80 %
CPREdictor T2017 w. GTGT	67.89 +/- 1.50 %
Dummy PREdictor w. GTGT	64.94 +/- 1.50 %
SVM-MOCCA	74.82 +/- 1.38 %

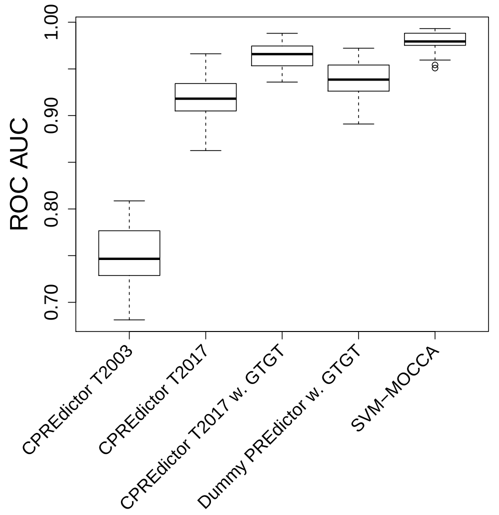

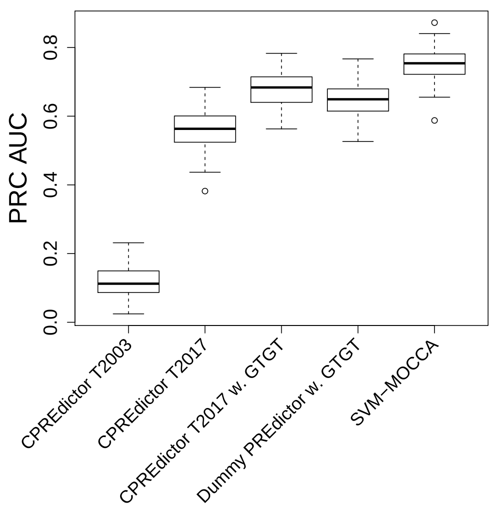

Classifier 1	Classifier 2	$p(H_0 : ROC1 <= ROC2)$	t	$p(H_0 : PRC1 <= PRC2)$	
CPREdictor T2003	CPREdictor T2003	-	-	-	
CPREdictor T2003	CPREdictor T2017	1.000000E+00	-3.732274E+01	1.000000E+00	-5[illegible]
CPREdictor T2003	CPREdictor T2017 w. GTGT	1.000000E+00	-4.607221E+01	1.000000E+00	-7[illegible]
CPREdictor T2003	Dummy PREdictor w. GTGT	1.000000E+00	-4.451863E+01	1.000000E+00	-7[illegible]
CPREdictor T2003	SVM-MOCCA	1.000000E+00	-4.553518E+01	1.000000E+00	-8[illegible]
CPREdictor T2017	CPREdictor T2003	5.971102E-38	3.732274E+01	1.469455E-47	5[illegible]
CPREdictor T2017	CPREdictor T2017	-	-	-	
CPREdictor T2017	CPREdictor T2017 w. GTGT	1.000000E+00	-1.753386E+01	1.000000E+00	-2[illegible]
CPREdictor T2017	Dummy PREdictor w. GTGT	1.000000E+00	-6.426571E+00	1.000000E+00	-1[illegible]
CPREdictor T2017	SVM-MOCCA	1.000000E+00	-1.848367E+01	1.000000E+00	-2[illegible]
CPREdictor T2017 w. GTGT	CPREdictor T2003	2.611054E-42	4.607221E+01	1.875927E-53	7[illegible]
CPREdictor T2017 w. GTGT	CPREdictor T2017	4.683991E-23	1.753386E+01	6.043936E-31	2[illegible]
CPREdictor T2017 w. GTGT	CPREdictor T2017 w. GTGT	-	-	-	
CPREdictor T2017 w. GTGT	Dummy PREdictor w. GTGT	7.848617E-24	1.828423E+01	1.327323E-09	7[illegible]
CPREdictor T2017 w. GTGT	SVM-MOCCA	1.000000E+00	-7.478324E+00	1.000000E+00	-1[illegible]
Dummy PREdictor w. GTGT	CPREdictor T2003	1.349215E-41	4.451863E+01	2.452816E-52	7[illegible]
Dummy PREdictor w. GTGT	CPREdictor T2017	2.565824E-08	6.426571E+00	3.945098E-19	1[illegible]
Dummy PREdictor w. GTGT	CPREdictor T2017 w. GTGT	1.000000E+00	-1.828423E+01	1.000000E+00	-7[illegible]
Dummy PREdictor w. GTGT	Dummy PREdictor w. GTGT	-	-	-	
Dummy PREdictor w. GTGT	SVM-MOCCA	1.000000E+00	-1.518366E+01	1.000000E+00	-2[illegible]
SVM-MOCCA	CPREdictor T2003	4.578701E-42	4.553518E+01	2.415136E-55	8[illegible]
SVM-MOCCA	CPREdictor T2017	4.927108E-24	1.848367E+01	2.443005E-32	2[illegible]
SVM-MOCCA	CPREdictor T2017 w. GTGT	6.043538E-10	7.478324E+00	8.787213E-19	1[illegible]
SVM-MOCCA	Dummy PREdictor w. GTGT	1.842680E-20	1.518366E+01	2.616900E-28	2[illegible]
SVM-MOCCA	SVM-MOCCA	-	-	-	

Classifier comparison - training set PREs: Schwartz 2010 PREs - Validation set: validation_PREsSchwartz vs. validation_D5merPREsSchwartz

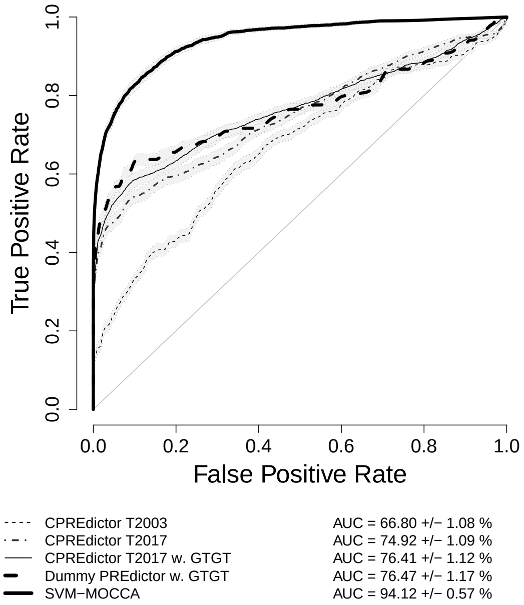

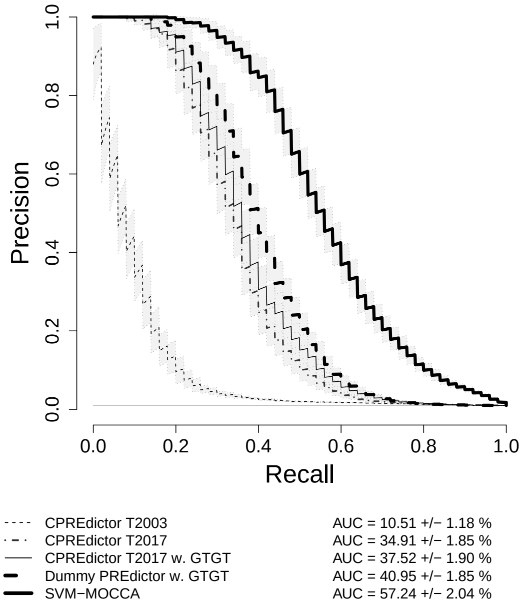

Classifier	ROC AUC
CPREdictor T2003	66.80 +/- 1.08 %
CPREdictor T2017	74.92 +/- 1.09 %
CPREdictor T2017 w. GTGT	76.41 +/- 1.12 %
Dummy PREdictor w. GTGT	76.47 +/- 1.17 %
SVM-MOCCA	94.12 +/- 0.57 %

Classifier	PRC AUC
CPREdictor T2003	10.51 +/- 1.18 %
CPREdictor T2017	34.91 +/- 1.85 %
CPREdictor T2017 w. GTGT	37.52 +/- 1.90 %
Dummy PREdictor w. GTGT	40.95 +/- 1.85 %
SVM-MOCCA	57.24 +/- 2.04 %

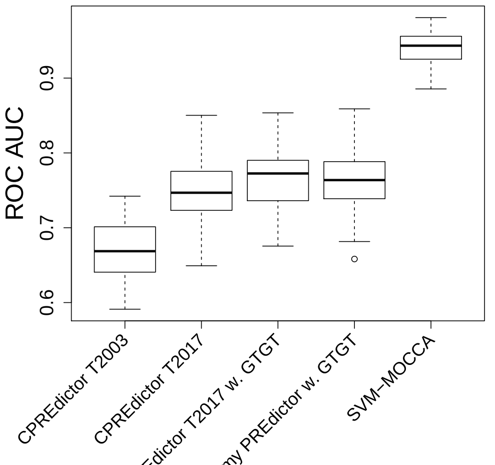

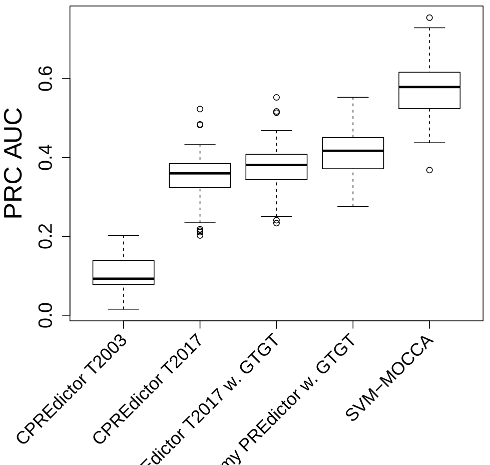

Classifier 1	Classifier 2	$p(H_0 : ROC1 <= ROC2)$	t	$p(H_0 : PRC1 <= PRC2)$	
CPREdictor T2003	CPREdictor T2003	-	-	-	
CPREdictor T2003	CPREdictor T2017	1.000000E+00	-1.357770E+01	1.000000E+00	-2
CPREdictor T2003	CPREdictor T2017 w. GTGT	1.000000E+00	-1.567490E+01	1.000000E+00	-3
CPREdictor T2003	Dummy PREdictor w. GTGT	1.000000E+00	-1.758233E+01	1.000000E+00	-3
CPREdictor T2003	SVM-MOCCA	1.000000E+00	-4.680712E+01	1.000000E+00	-4
CPREdictor T2017	CPREdictor T2003	1.570290E-18	1.357770E+01	4.664484E-33	2
CPREdictor T2017	CPREdictor T2017	-	-	-	
CPREdictor T2017	CPREdictor T2017 w. GTGT	9.999424E-01	-4.192038E+00	1.000000E+00	-1
CPREdictor T2017	Dummy PREdictor w. GTGT	9.991435E-01	-3.318416E+00	1.000000E+00	-1
CPREdictor T2017	SVM-MOCCA	1.000000E+00	-3.672911E+01	1.000000E+00	-3
CPREdictor T2017 w. GTGT	CPREdictor T2003	5.026969E-21	1.567490E+01	2.984238E-34	3
CPREdictor T2017 w. GTGT	CPREdictor T2017	5.761909E-05	4.192038E+00	1.188161E-15	1
CPREdictor T2017 w. GTGT	CPREdictor T2017 w. GTGT	-	-	-	
CPREdictor T2017 w. GTGT	Dummy PREdictor w. GTGT	5.896643E-01	-2.279011E-01	1.000000E+00	-8
CPREdictor T2017 w. GTGT	SVM-MOCCA	1.000000E+00	-3.549485E+01	1.000000E+00	-2
Dummy PREdictor w. GTGT	CPREdictor T2003	4.166453E-23	1.758233E+01	1.154506E-37	3
Dummy PREdictor w. GTGT	CPREdictor T2017	8.564522E-04	3.318416E+00	4.532895E-17	1
Dummy PREdictor w. GTGT	CPREdictor T2017 w. GTGT	4.103357E-01	2.279011E-01	4.827804E-11	8
Dummy PREdictor w. GTGT	Dummy PREdictor w. GTGT	-	-	-	
Dummy PREdictor w. GTGT	SVM-MOCCA	1.000000E+00	-3.442788E+01	1.000000E+00	-2
SVM-MOCCA	CPREdictor T2003	1.222876E-42	4.680712E+01	2.043927E-43	4
SVM-MOCCA	CPREdictor T2017	1.275574E-37	3.672911E+01	1.116145E-35	3
SVM-MOCCA	CPREdictor T2017 w. GTGT	6.416161E-37	3.549485E+01	6.702903E-32	2
SVM-MOCCA	Dummy PREdictor w. GTGT	2.705340E-36	3.442788E+01	2.477113E-30	2
SVM-MOCCA	SVM-MOCCA	-	-	-	

Numbers of candidate PREs (training set PREs: Schwartz 2010 PREs)

All the following predictions are for an expected precision of 0.8.

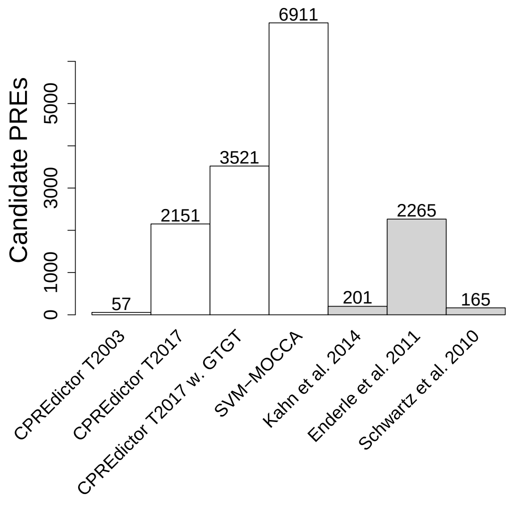

Set	Candidate PREs
CPREdictor T2003	57
CPREdictor T2017	2151
CPREdictor T2017 w. GTGT	3521
SVM-MOCCA	6911
Kahn et al. 2014	201
Enderle et al. 2011	2265
Schwartz et al. 2010	165

Repressed PRE Polycomb recruitment (training set PREs: Schwartz 2010 PREs)

All the following are for an expected precision of 0.8.

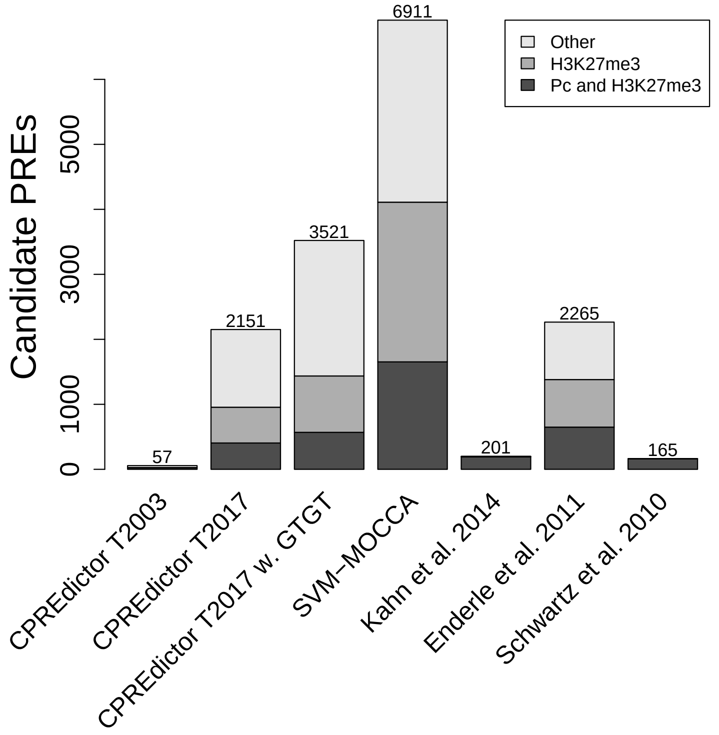

Repressed PRE Polycomb recruitment (training set PREs: Schwartz 2010 PREs)

All the following are for an expected precision of 0.8.

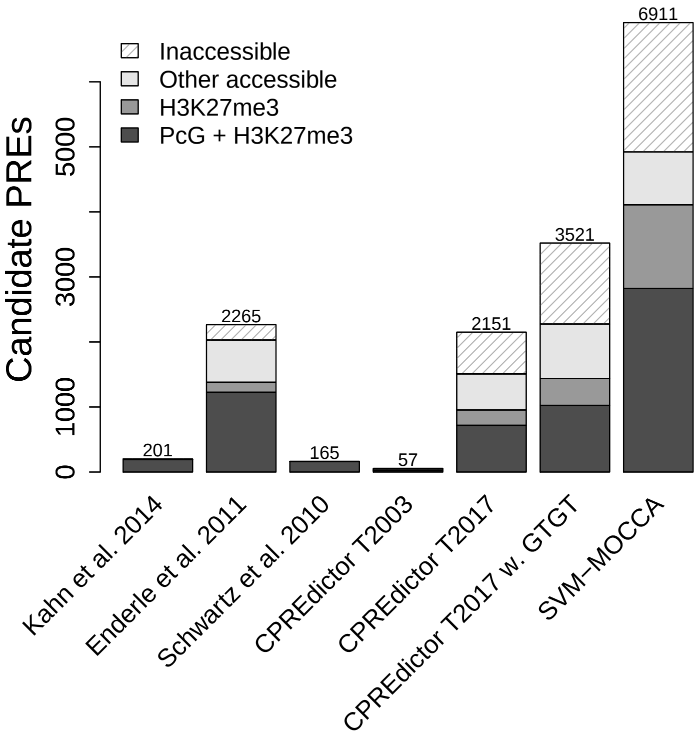

PRE set overlap sensitivity (training set PREs: Schwartz 2010 PREs)

All the following are for an expected precision of 0.8.

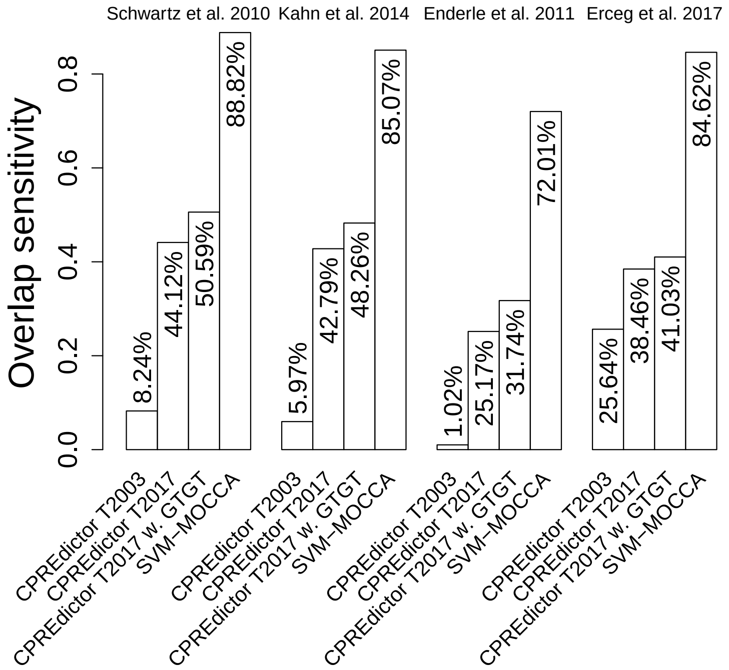

Overlap sensitivity	Kahn et al. 2014	Enderle et al. 2011	Schwartz et al. 2010
CPREdictor T2003	5.97 %	1.02 %	7.88 %
CPREdictor T2017	42.79 %	25.17 %	44.24 %
CPREdictor T2017 w. GTGT	48.26 %	31.74 %	50.30 %
SVM-MOCCA	85.07 %	72.01 %	88.48 %

PRE set overlap sensitivity (training set PREs: Schwartz 2010 PREs)

All the following are for an expected precision of 0.8.

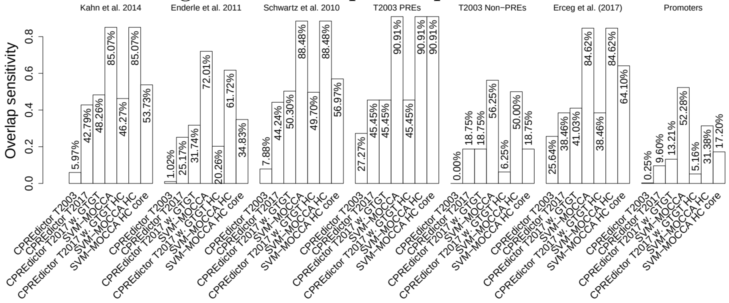

Overlap sensitivity	Kahn et al. 2014	Enderle et al. 2011	Schwartz et al. 2010	T2003 PREs	T200
CPREdictor T2003	5.97 %	1.02 %	7.88 %	27.27 %	
CPREdictor T2017	42.79 %	25.17 %	44.24 %	45.45 %	
CPREdictor T2017 w. GTGT	48.26 %	31.74 %	50.30 %	45.45 %	
SVM-MOCCA	85.07 %	72.01 %	88.48 %	90.91 %	
CPREdictor T2017 w. GTGT HC	46.27 %	20.26 %	49.70 %	45.45 %	
SVM-MOCCA HC	85.07 %	61.72 %	88.48 %	90.91 %	
SVM-MOCCA HC core	53.73 %	34.83 %	56.97 %	90.91 %	

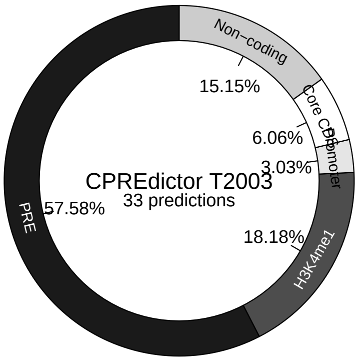

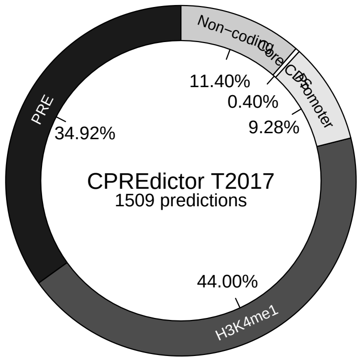

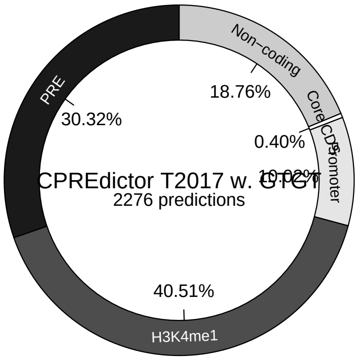

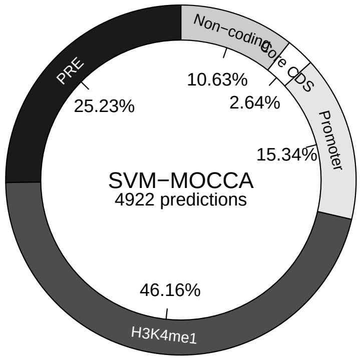

PcG/TrxG target genes (training set PREs: Schwartz 2010 PREs)

All the following are for an expected precision of 0.8. * Kahn et al had not published any gene lists, so we predicted target genes ourselves based on computationally determined PREs. ** Where published sets of genes contained genes not found in the latest FlyBase annotation, we left those out.

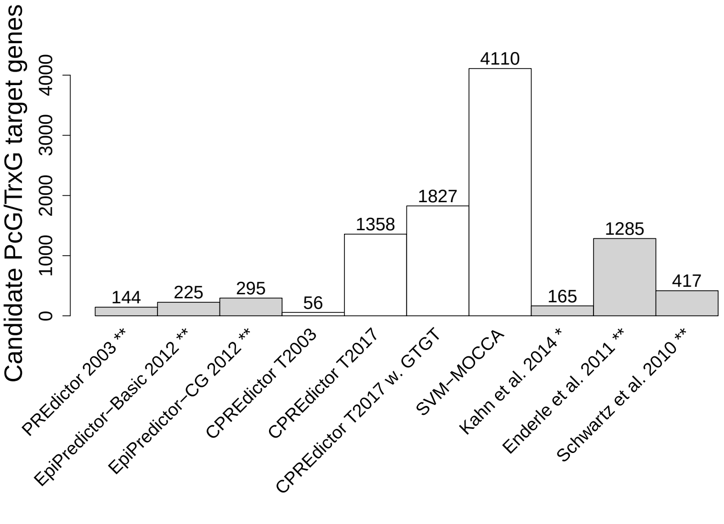

Set	Candidate PcG/TrxG target genes
PREdictor 2003 **	144
EpiPredictor-Basic 2012 **	225
EpiPredictor-CG 2012 **	295
CPREdictor T2003	56
CPREdictor T2017	1358
CPREdictor T2017 w. GTGT	1827
SVM-MOCCA	4110
Kahn et al. 2014 *	165
Enderle et al. 2011 **	1285
Schwartz et al. 2010 **	417

PcG/TrxG target gene sensitivity (training set PREs: Schwartz 2010 PREs)

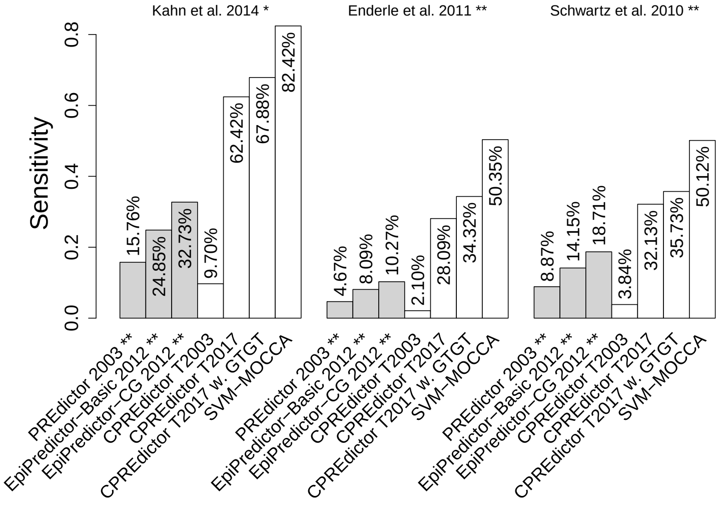

Sensitivity	Kahn et al. 2014 *	Enderle et al. 2011 **	Schwartz et al. 2010 **
PREdictor 2003 **	15.76 %	4.67 %	8.87 %
EpiPredictor-Basic 2012 **	24.85 %	8.09 %	14.15 %
EpiPredictor-CG 2012 **	32.73 %	10.27 %	18.71 %
CPREdictor T2003	9.70 %	2.10 %	3.84 %
CPREdictor T2017	62.42 %	28.09 %	32.13 %
CPREdictor T2017 w. GTGT	67.88 %	34.32 %	35.73 %
SVM-MOCCA	82.42 %	50.35 %	50.12 %

PcG/TrxG target gene precision (training set PREs: Schwartz 2010 PREs)

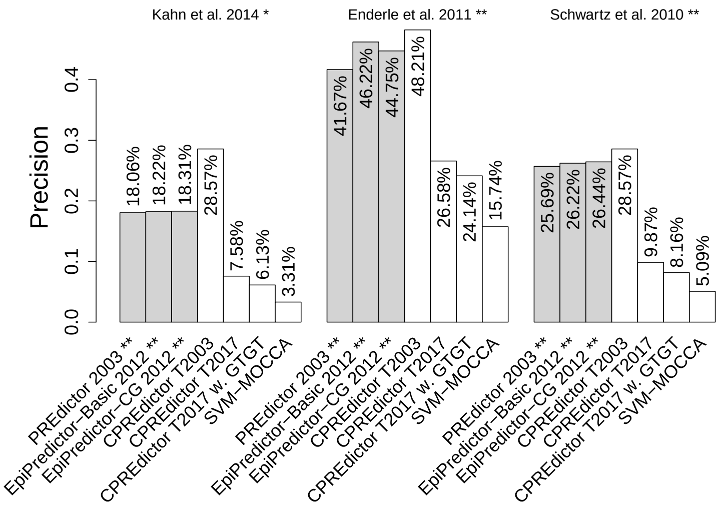

Precision	Kahn et al. 2014 *	Enderle et al. 2011 **	Schwartz et al. 2010 **
PREdictor 2003 **	18.06 %	41.67 %	25.69 %
EpiPredictor-Basic 2012 **	18.22 %	46.22 %	26.22 %
EpiPredictor-CG 2012 **	18.31 %	44.75 %	26.44 %
CPREdictor T2003	28.57 %	48.21 %	28.57 %
CPREdictor T2017	7.58 %	26.58 %	9.87 %
CPREdictor T2017 w. GTGT	6.13 %	24.14 %	8.16 %
SVM-MOCCA	3.31 %	15.74 %	5.09 %

PcG/TrxG target gene P-values (training set PREs: Schwartz 2010 PREs)

P-value	Kahn et al. 2014 *	Enderle et al. 2011 **	Schwartz et al. 2010 **
PREdictor 2003 **	3.32e-26	1.24e-30	6.09e-28
EpiPredictor-Basic 2012 **	1.96e-41	7.25e-58	9.20e-45
EpiPredictor-CG 2012 **	3.27e-55	2.73e-71	1.13e-59
CPREdictor T2003	5.65e-20	1.73e-16	1.43e-13
CPREdictor T2017	2.15e-72	1.49e-117	7.87e-49
CPREdictor T2017 w. GTGT	1.62e-70	1.21e-130	1.67e-44
SVM-MOCCA	4.91e-58	1.34e-105	5.79e-33

Genomic loci of PREs per predicted target gene (training set PREs: Schwartz 2010 PREs)

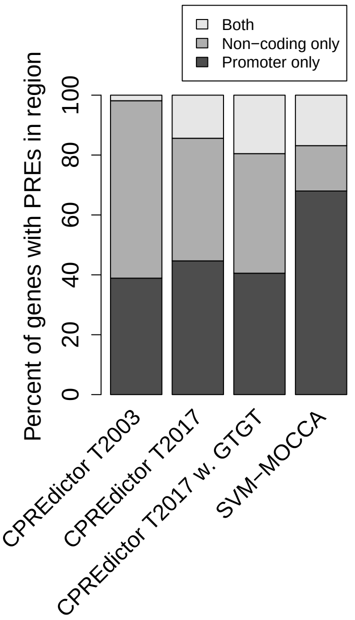

Gene Venn diagrams (training set PREs: Schwartz 2010 PREs)

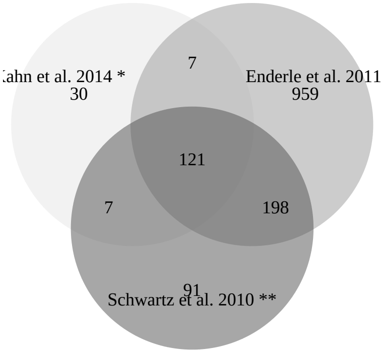

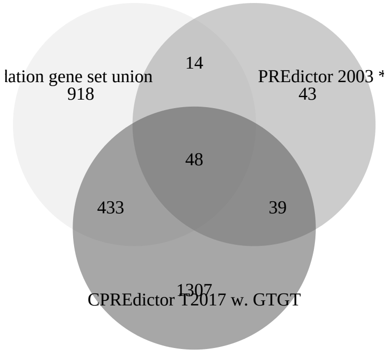

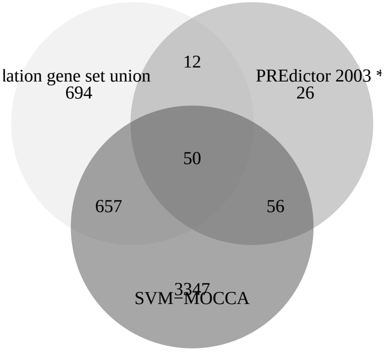

Training set PREs: Kahn 2014 PREs

Classifier comparison (training set PREs: Kahn 2014 PREs)

CPREdictor T2003
CPREdictor T2017
CPREdictor T2017 w. GTGT
Dummy PREdictor w. GTGT
SVM−MOCCA

Classifier	Path
CPREdictor T2003	CPREdictor_M2003_T2003_mdBetween_wmPREdictor
CPREdictor T2017	CPREdictor_M2003_CPPREsKahn_CND5merPREsKahn_T110_mdBetween_wmPREdictor
CPREdictor T2017 w. GTGT	CPREdictor_M2003_GTGT_CPPREsKahn_CND5merPREsKahn_T110_mdBetween_wmPREdictor
Dummy PREdictor w. GTGT	DummyPREdictor_M2003_GTGT_T2003_mdBetween_wmPREdictor
SVM-MOCCA	SVMMOCCA_kquadratic_fnOcc_fDNT_M2003_GTGT_CPPREsKahn_CNR5mers_CNCDS_CND5merPREsKahn_T110_ws3000_mdBetween_wmPREd

Classifier comparison - training set PREs: Kahn 2014 PREs - Validation set: validation_PREsKahn vs. validation_R5mers

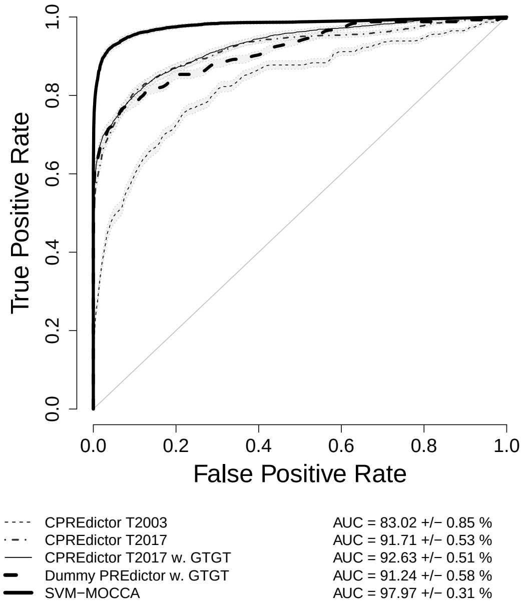

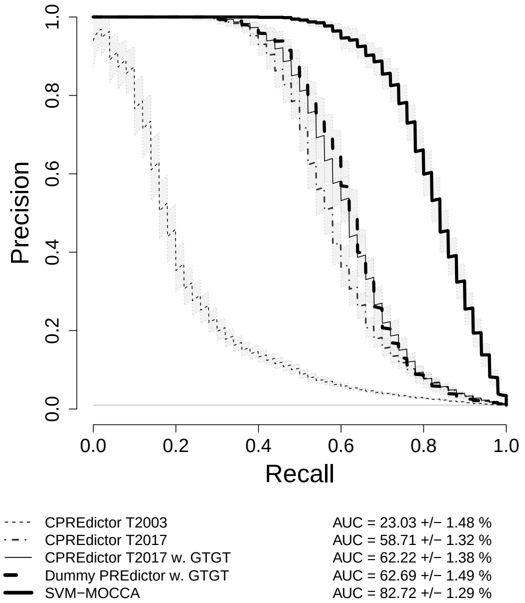

Classifier	ROC AUC
CPREdictor T2003	83.02 +/- 0.85 %
CPREdictor T2017	91.71 +/- 0.53 %
CPREdictor T2017 w. GTGT	92.63 +/- 0.51 %
Dummy PREdictor w. GTGT	91.24 +/- 0.58 %
SVM-MOCCA	97.97 +/- 0.31 %

Classifier	PRC AUC
CPREdictor T2003	23.03 +/- 1.48 %
CPREdictor T2017	58.71 +/- 1.32 %
CPREdictor T2017 w. GTGT	62.22 +/- 1.38 %
Dummy PREdictor w. GTGT	62.69 +/- 1.49 %
SVM-MOCCA	82.72 +/- 1.29 %

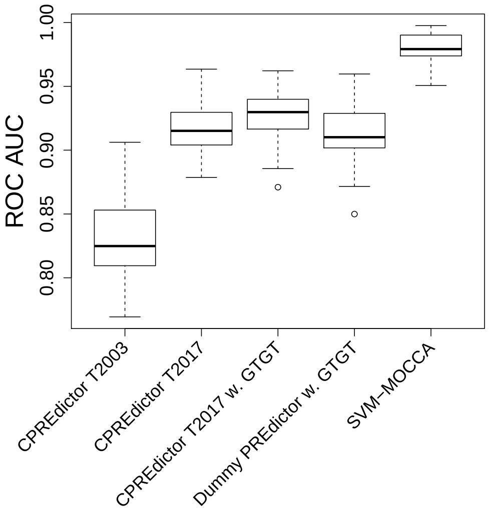

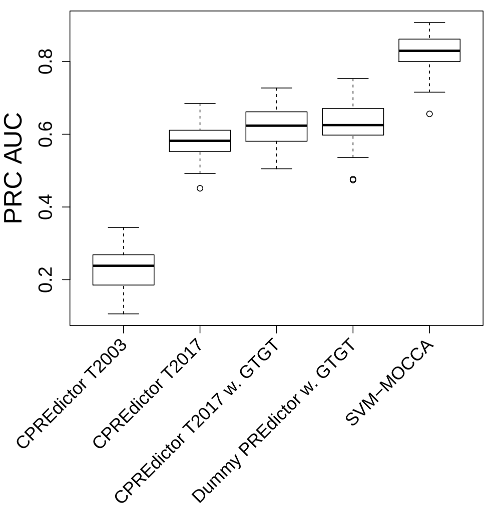

Classifier 1	Classifier 2	$p(H_0 : ROC1 <= ROC2)$	t	$p(H_0 : PRC1 <= PRC2)$	
CPREdictor T2003	CPREdictor T2003	-	-	-	
CPREdictor T2003	CPREdictor T2017	1.000000E+00	-2.793018E+01	1.000000E+00	[illegible]
CPREdictor T2003	CPREdictor T2017 w. GTGT	1.000000E+00	-2.234396E+01	1.000000E+00	[illegible]
CPREdictor T2003	Dummy PREdictor w. GTGT	1.000000E+00	-2.275688E+01	1.000000E+00	[illegible]
CPREdictor T2003	SVM-MOCCA	1.000000E+00	-3.495166E+01	1.000000E+00	[illegible]
CPREdictor T2017	CPREdictor T2003	4.680448E-32	2.793018E+01	6.432387E-45	[illegible]
CPREdictor T2017	CPREdictor T2017	-	-	-	
CPREdictor T2017	CPREdictor T2017 w. GTGT	9.989567E-01	-3.250426E+00	1.000000E+00	[illegible]
CPREdictor T2017	Dummy PREdictor w. GTGT	5.805044E-02	1.599669E+00	1.000000E+00	[illegible]
CPREdictor T2017	SVM-MOCCA	1.000000E+00	-2.137597E+01	1.000000E+00	[illegible]
CPREdictor T2017 w. GTGT	CPREdictor T2003	1.196399E-27	2.234396E+01	6.476837E-42	[illegible]
CPREdictor T2017 w. GTGT	CPREdictor T2017	1.043256E-03	3.250426E+00	7.903684E-12	[illegible]
CPREdictor T2017 w. GTGT	CPREdictor T2017 w. GTGT	-	-	-	
CPREdictor T2017 w. GTGT	Dummy PREdictor w. GTGT	1.076867E-10	7.965970E+00	8.709585E-01	[illegible]
CPREdictor T2017 w. GTGT	SVM-MOCCA	1.000000E+00	-1.700256E+01	1.000000E+00	[illegible]
Dummy PREdictor w. GTGT	CPREdictor T2003	5.269571E-28	2.275688E+01	1.870334E-41	[illegible]
Dummy PREdictor w. GTGT	CPREdictor T2017	9.419496E-01	-1.599669E+00	2.262760E-08	[illegible]
Dummy PREdictor w. GTGT	CPREdictor T2017 w. GTGT	1.000000E+00	-7.965970E+00	1.290415E-01	[illegible]
Dummy PREdictor w. GTGT	Dummy PREdictor w. GTGT	-	-	-	
Dummy PREdictor w. GTGT	SVM-MOCCA	1.000000E+00	-1.993336E+01	1.000000E+00	[illegible]
SVM-MOCCA	CPREdictor T2003	1.328091E-36	3.495166E+01	7.925811E-51	[illegible]
SVM-MOCCA	CPREdictor T2017	8.597194E-27	2.137597E+01	5.332143E-37	[illegible]
SVM-MOCCA	CPREdictor T2017 w. GTGT	1.716983E-22	1.700256E+01	1.278974E-33	[illegible]
SVM-MOCCA	Dummy PREdictor w. GTGT	1.865624E-25	1.993336E+01	3.885152E-34	[illegible]
SVM-MOCCA	SVM-MOCCA	-	-	-	

Classifier comparison - training set PREs: Kahn 2014 PREs - Validation set: validation_PREsKahn vs. validation_CDS

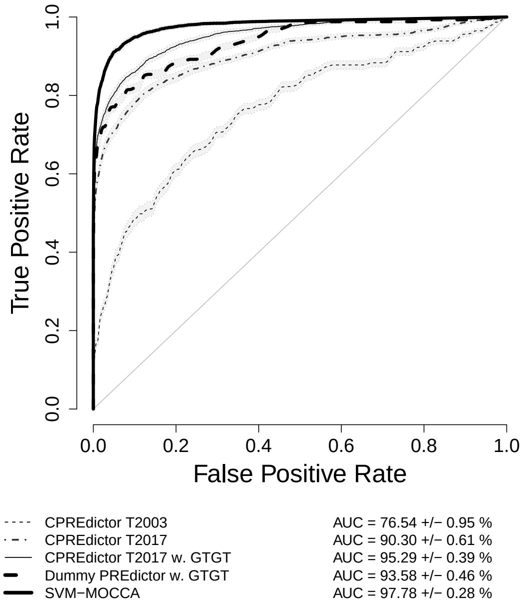

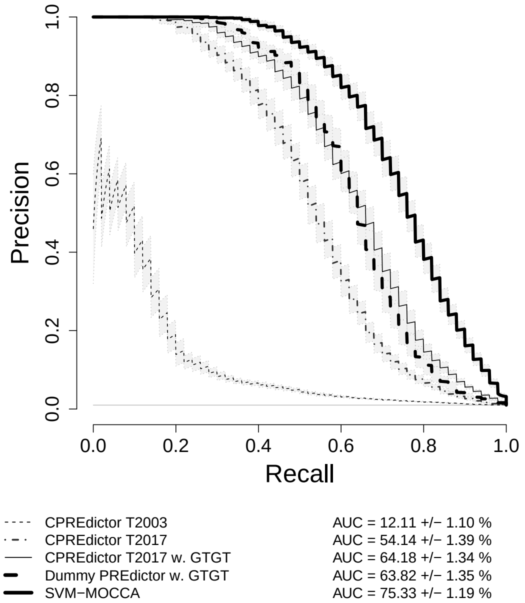

Classifier	ROC AUC
CPREdictor T2003	76.54 +/- 0.95 %
CPREdictor T2017	90.30 +/- 0.61 %
CPREdictor T2017 w. GTGT	95.29 +/- 0.39 %
Dummy PREdictor w. GTGT	93.58 +/- 0.46 %
SVM-MOCCA	97.78 +/- 0.28 %

Classifier	PRC AUC
CPREdictor T2003	12.11 +/- 1.10 %
CPREdictor T2017	54.14 +/- 1.39 %
CPREdictor T2017 w. GTGT	64.18 +/- 1.34 %
Dummy PREdictor w. GTGT	63.82 +/- 1.35 %
SVM-MOCCA	75.33 +/- 1.19 %

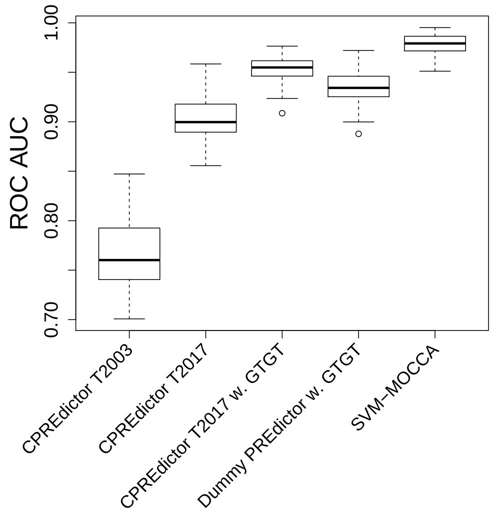

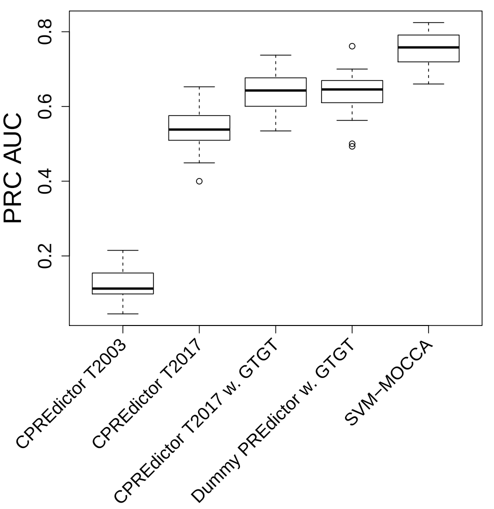

Classifier 1	Classifier 2	$p(H_0 : ROC1 <= ROC2)$	t	$p(H_0 : PRC1 <= PRC2)$	
CPREdictor T2003	CPREdictor T2003	-	-	-	
CPREdictor T2003	CPREdictor T2017	1.000000E+00	-3.813990E+01	1.000000E+00	-5
CPREdictor T2003	CPREdictor T2017 w. GTGT	1.000000E+00	-3.938282E+01	1.000000E+00	-6
CPREdictor T2003	Dummy PREdictor w. GTGT	1.000000E+00	-4.198324E+01	1.000000E+00	-6
CPREdictor T2003	SVM-MOCCA	1.000000E+00	-4.433700E+01	1.000000E+00	-7
CPREdictor T2017	CPREdictor T2003	2.138939E-38	3.813990E+01	4.548979E-46	5
CPREdictor T2017	CPREdictor T2017	-	-	-	
CPREdictor T2017	CPREdictor T2017 w. GTGT	1.000000E+00	-1.804125E+01	1.000000E+00	-2
CPREdictor T2017	Dummy PREdictor w. GTGT	1.000000E+00	-1.134006E+01	1.000000E+00	-1
CPREdictor T2017	SVM-MOCCA	1.000000E+00	-2.304111E+01	1.000000E+00	-3
CPREdictor T2017 w. GTGT	CPREdictor T2003	4.665576E-39	3.938282E+01	1.222646E-48	6
CPREdictor T2017 w. GTGT	CPREdictor T2017	1.391198E-23	1.804125E+01	1.459154E-28	2
CPREdictor T2017 w. GTGT	CPREdictor T2017 w. GTGT	-	-	-	
CPREdictor T2017 w. GTGT	Dummy PREdictor w. GTGT	2.629938E-15	1.112128E+01	2.035498E-01	
CPREdictor T2017 w. GTGT	SVM-MOCCA	1.000000E+00	-1.017615E+01	1.000000E+00	-2
Dummy PREdictor w. GTGT	CPREdictor T2003	2.221437E-40	4.198324E+01	3.731285E-49	6
Dummy PREdictor w. GTGT	CPREdictor T2017	1.315656E-15	1.134006E+01	5.848838E-22	1
Dummy PREdictor w. GTGT	CPREdictor T2017 w. GTGT	1.000000E+00	-1.112128E+01	7.964502E-01	-
Dummy PREdictor w. GTGT	Dummy PREdictor w. GTGT	-	-	-	
Dummy PREdictor w. GTGT	SVM-MOCCA	1.000000E+00	-1.565241E+01	1.000000E+00	-2
SVM-MOCCA	CPREdictor T2003	1.640637E-41	4.433700E+01	3.262996E-53	7
SVM-MOCCA	CPREdictor T2017	3.018328E-28	2.304111E+01	5.117057E-37	3
SVM-MOCCA	CPREdictor T2017 w. GTGT	5.619503E-14	1.017615E+01	1.882862E-28	2
SVM-MOCCA	Dummy PREdictor w. GTGT	5.331717E-21	1.565241E+01	2.927806E-28	2
SVM-MOCCA	SVM-MOCCA	-	-	-	

Classifier comparison - training set PREs: Kahn 2014 PREs - Validation set: validation_PREsKahn vs. validation_D5merPREsKahn

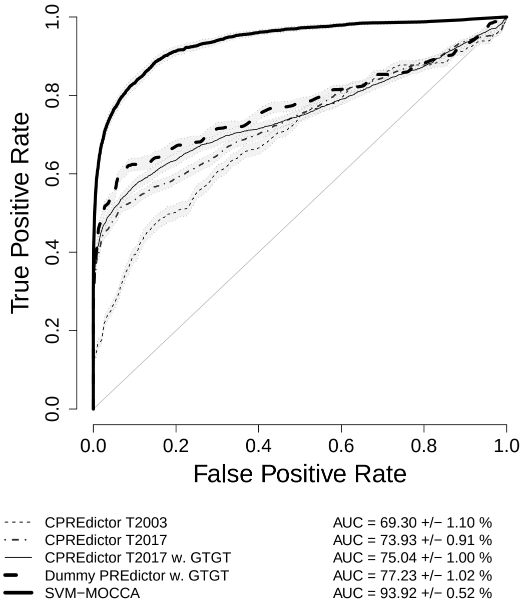

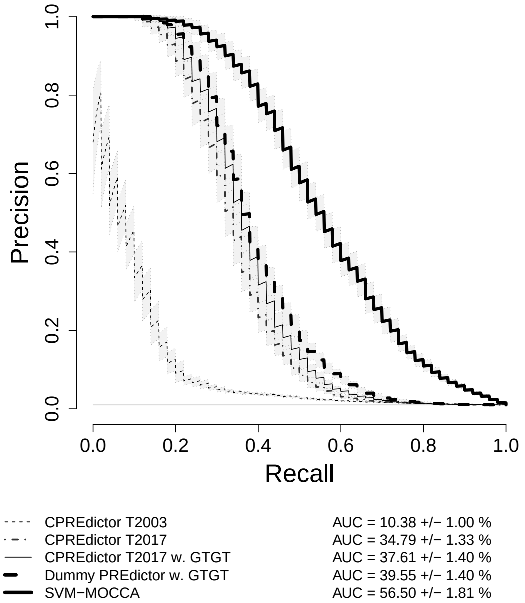

Classifier	ROC AUC
CPREdictor T2003	69.30 +/- 1.10 %
CPREdictor T2017	73.93 +/- 0.91 %
CPREdictor T2017 w. GTGT	75.04 +/- 1.00 %
Dummy PREdictor w. GTGT	77.23 +/- 1.02 %
SVM-MOCCA	93.92 +/- 0.52 %

Classifier	PRC AUC
CPREdictor T2003	10.38 +/- 1.00 %
CPREdictor T2017	34.79 +/- 1.33 %
CPREdictor T2017 w. GTGT	37.61 +/- 1.40 %
Dummy PREdictor w. GTGT	39.55 +/- 1.40 %
SVM-MOCCA	56.50 +/- 1.81 %

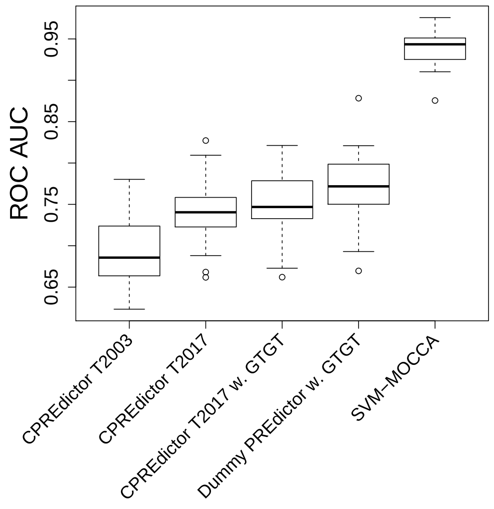

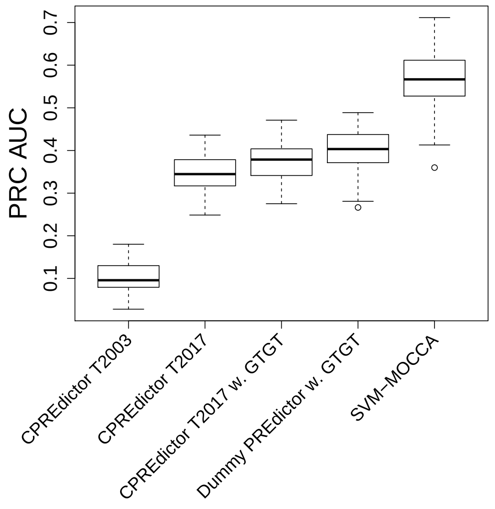

Classifier 1	Classifier 2	$p(H_0 : ROC1 <= ROC2)$	t	$p(H_0 : PRC1 <= PRC2)$	
CPREdictor T2003	CPREdictor T2003	-	-	-	
CPREdictor T2003	CPREdictor T2017	1.000000E+00	-9.734138E+00	1.000000E+00	-3[illegible]
CPREdictor T2003	CPREdictor T2017 w. GTGT	1.000000E+00	-9.252291E+00	1.000000E+00	-3[illegible]
CPREdictor T2003	Dummy PREdictor w. GTGT	1.000000E+00	-1.396887E+01	1.000000E+00	-3[illegible]
CPREdictor T2003	SVM-MOCCA	1.000000E+00	-4.411119E+01	1.000000E+00	-5[illegible]
CPREdictor T2017	CPREdictor T2003	2.443222E-13	9.734138E+00	3.084483E-35	3[illegible]
CPREdictor T2017	CPREdictor T2017	-	-	-	
CPREdictor T2017	CPREdictor T2017 w. GTGT	9.987427E-01	-3.185425E+00	1.000000E+00	-1[illegible]
CPREdictor T2017	Dummy PREdictor w. GTGT	1.000000E+00	-7.510577E+00	1.000000E+00	-9[illegible]
CPREdictor T2017	SVM-MOCCA	1.000000E+00	-4.503066E+01	1.000000E+00	-3[illegible]
CPREdictor T2017 w. GTGT	CPREdictor T2003	1.244346E-12	9.252291E+00	3.890337E-36	3[illegible]
CPREdictor T2017 w. GTGT	CPREdictor T2017	1.257345E-03	3.185425E+00	1.502398E-16	1[illegible]
CPREdictor T2017 w. GTGT	CPREdictor T2017 w. GTGT	-	-	-	
CPREdictor T2017 w. GTGT	Dummy PREdictor w. GTGT	1.000000E+00	-7.313479E+00	9.999725E-01	-4[illegible]
CPREdictor T2017 w. GTGT	SVM-MOCCA	1.000000E+00	-3.810830E+01	1.000000E+00	-2[illegible]
Dummy PREdictor w. GTGT	CPREdictor T2003	5.167036E-19	1.396887E+01	2.027744E-38	3[illegible]
Dummy PREdictor w. GTGT	CPREdictor T2017	5.389748E-10	7.510577E+00	8.772444E-13	9[illegible]
Dummy PREdictor w. GTGT	CPREdictor T2017 w. GTGT	1.085769E-09	7.313479E+00	2.751174E-05	4[illegible]
Dummy PREdictor w. GTGT	Dummy PREdictor w. GTGT	-	-	-	
Dummy PREdictor w. GTGT	SVM-MOCCA	1.000000E+00	-3.423962E+01	1.000000E+00	-2[illegible]
SVM-MOCCA	CPREdictor T2003	2.094441E-41	4.411119E+01	1.607860E-44	5[illegible]
SVM-MOCCA	CPREdictor T2017	7.805746E-42	4.503066E+01	6.039010E-34	3[illegible]
SVM-MOCCA	CPREdictor T2017 w. GTGT	2.224717E-38	3.810830E+01	7.902770E-32	2[illegible]
SVM-MOCCA	Dummy PREdictor w. GTGT	3.502211E-36	3.423962E+01	2.519045E-30	2[illegible]
SVM-MOCCA	SVM-MOCCA	-	-	-	

Numbers of candidate PREs (training set PREs: Kahn 2014 PREs)

All the following predictions are for an expected precision of 0.8.

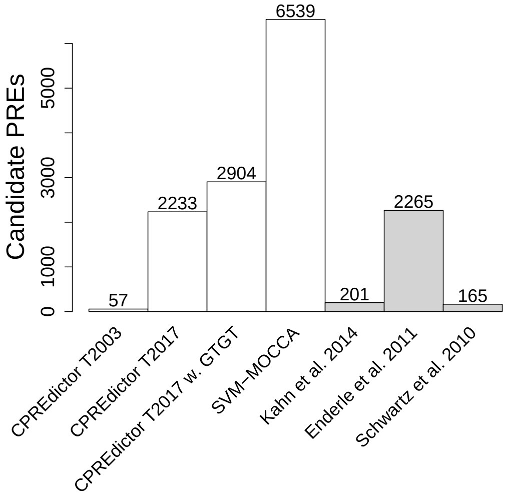

Set	Candidate PREs
CPREdictor T2003	57
CPREdictor T2017	2233
CPREdictor T2017 w. GTGT	2904
SVM-MOCCA	6539
Kahn et al. 2014	201
Enderle et al. 2011	2265
Schwartz et al. 2010	165

Repressed PRE Polycomb recruitment (training set PREs: Kahn 2014 PREs)

All the following are for an expected precision of 0.8.

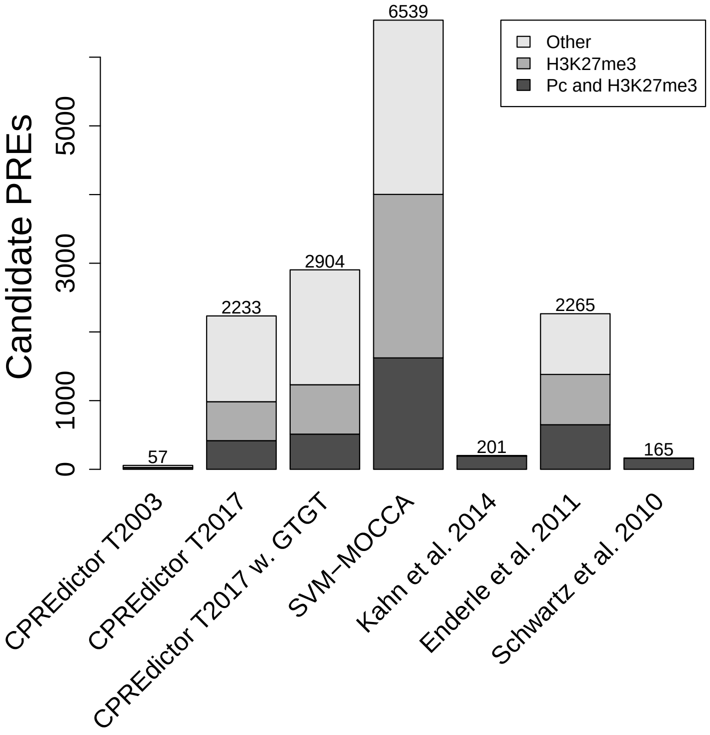

PRE set overlap sensitivity (training set PREs: Kahn 2014 PREs)

All the following are for an expected precision of 0.8.

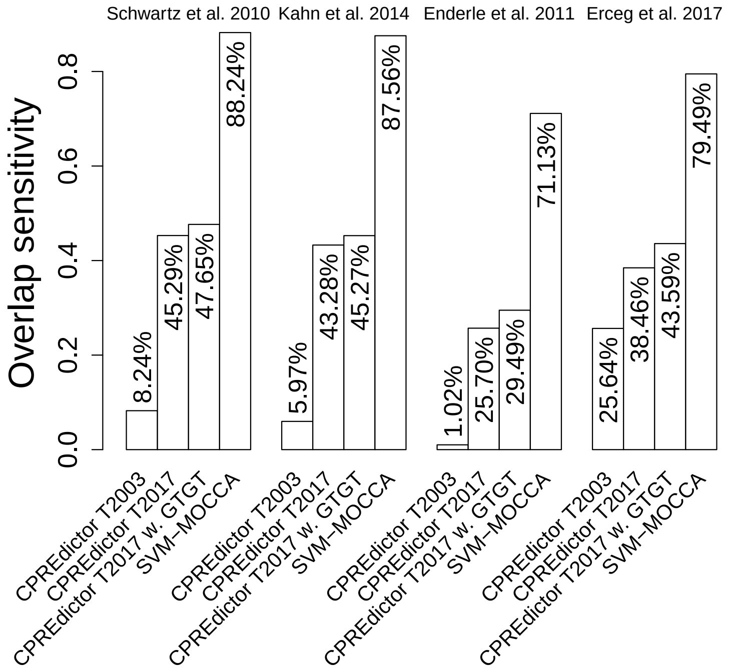

Overlap sensitivity	Kahn et al. 2014	Enderle et al. 2011	Schwartz et al. 2010
CPREdictor T2003	5.97 %	1.02 %	7.88 %
CPREdictor T2017	43.28 %	25.70 %	45.45 %
CPREdictor T2017 w. GTGT	45.27 %	29.49 %	47.27 %
SVM-MOCCA	87.56 %	71.13 %	87.88 %

PRE set overlap sensitivity (training set PREs: Kahn 2014 PREs)

All the following are for an expected precision of 0.8.

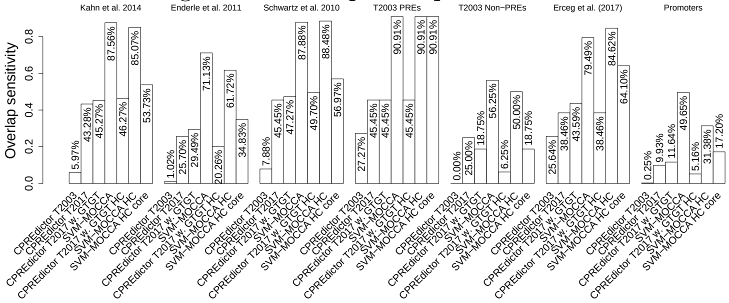

Overlap sensitivity	Kahn et al. 2014	Enderle et al. 2011	Schwartz et al. 2010	T2003 PREs	T200
CPREdictor T2003	5.97 %	1.02 %	7.88 %	27.27 %	
CPREdictor T2017	43.28 %	25.70 %	45.45 %	45.45 %	
CPREdictor T2017 w. GTGT	45.27 %	29.49 %	47.27 %	45.45 %	
SVM-MOCCA	87.56 %	71.13 %	87.88 %	90.91 %	
CPREdictor T2017 w. GTGT HC	46.27 %	20.26 %	49.70 %	45.45 %	
SVM-MOCCA HC	85.07 %	61.72 %	88.48 %	90.91 %	
SVM-MOCCA HC core	53.73 %	34.83 %	56.97 %	90.91 %	

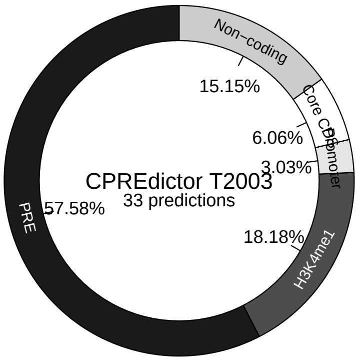

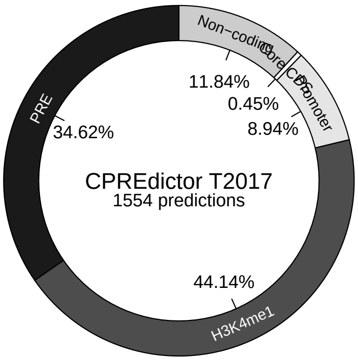

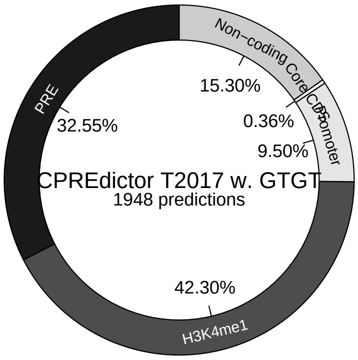

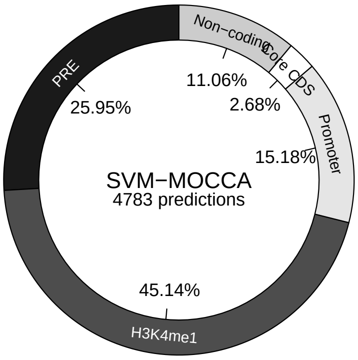

PcG/TrxG target genes (training set PREs: Kahn 2014 PREs)

All the following are for an expected precision of 0.8. * Kahn et al had not published any gene lists, so we predicted target genes ourselves based on computationally determined PREs. ** Where published sets of genes contained genes not found in the latest FlyBase annotation, we left those out.

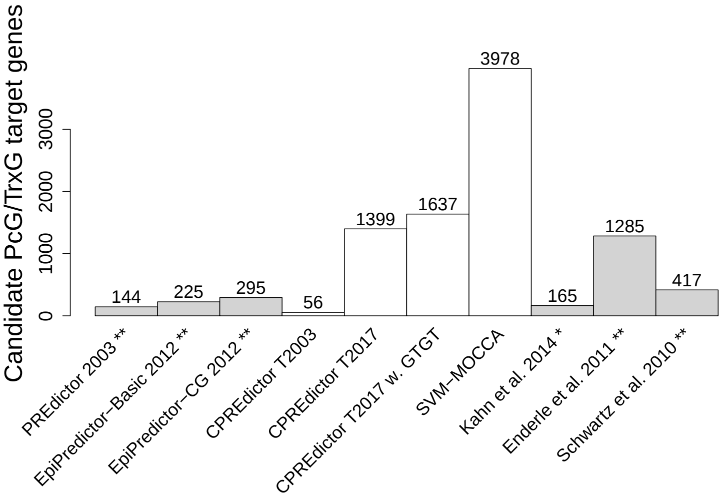

Set	Candidate PcG/TrxG target genes
PREdictor 2003 **	144
EpiPredictor-Basic 2012 **	225
EpiPredictor-CG 2012 **	295
CPREdictor T2003	56
CPREdictor T2017	1399
CPREdictor T2017 w. GTGT	1637
SVM-MOCCA	3978
Kahn et al. 2014 *	165
Enderle et al. 2011 **	1285
Schwartz et al. 2010 **	417

PcG/TrxG target gene sensitivity (training set PREs: Kahn 2014 PREs)

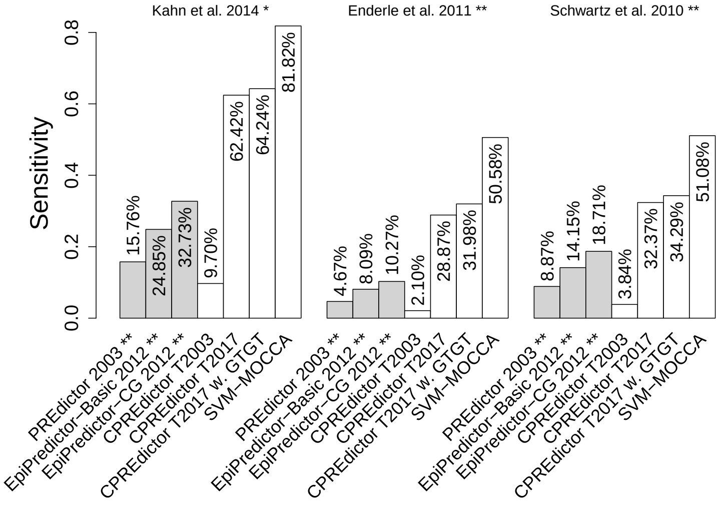

Sensitivity	Kahn et al. 2014 *	Enderle et al. 2011 **	Schwartz et al. 2010 **
PREdictor 2003 **	15.76 %	4.67 %	8.87 %
EpiPredictor-Basic 2012 **	24.85 %	8.09 %	14.15 %
EpiPredictor-CG 2012 **	32.73 %	10.27 %	18.71 %
CPREdictor T2003	9.70 %	2.10 %	3.84 %
CPREdictor T2017	62.42 %	28.87 %	32.37 %
CPREdictor T2017 w. GTGT	64.24 %	31.98 %	34.29 %
SVM-MOCCA	81.82 %	50.58 %	51.08 %

PcG/TrxG target gene precision (training set PREs: Kahn 2014 PREs)

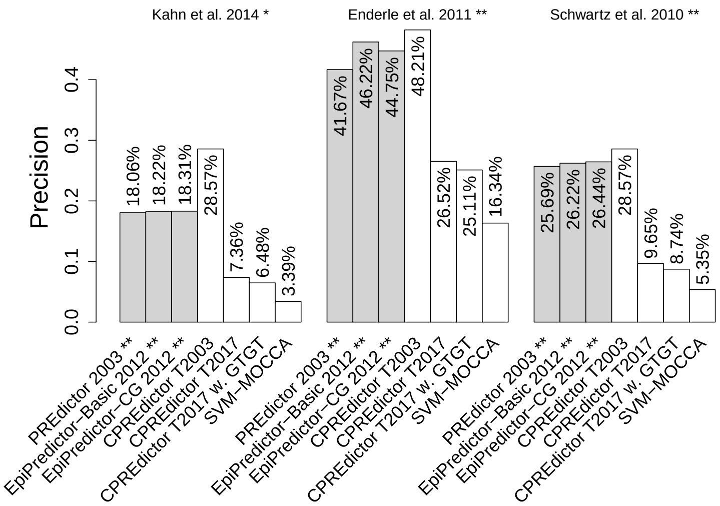

Precision	Kahn et al. 2014 *	Enderle et al. 2011 **	Schwartz et al. 2010 **
PREdictor 2003 **	18.06 %	41.67 %	25.69 %
EpiPredictor-Basic 2012 **	18.22 %	46.22 %	26.22 %
EpiPredictor-CG 2012 **	18.31 %	44.75 %	26.44 %
CPREdictor T2003	28.57 %	48.21 %	28.57 %
CPREdictor T2017	7.36 %	26.52 %	9.65 %
CPREdictor T2017 w. GTGT	6.48 %	25.11 %	8.74 %
SVM-MOCCA	3.39 %	16.34 %	5.35 %

PcG/TrxG target gene P-values (training set PREs: Kahn 2014 PREs)

P-value	Kahn et al. 2014 *	Enderle et al. 2011 **	Schwartz et al. 2010 **
PREdictor 2003 **	3.32e-26	1.24e-30	6.09e-28
EpiPredictor-Basic 2012 **	1.96e-41	7.25e-58	9.20e-45
EpiPredictor-CG 2012 **	3.27e-55	2.73e-71	1.13e-59
CPREdictor T2003	5.65e-20	1.73e-16	1.43e-13
CPREdictor T2017	4.45e-71	7.13e-121	4.24e-48
CPREdictor T2017 w. GTGT	6.00e-68	9.85e-127	5.11e-46
SVM-MOCCA	1.14e-58	1.33e-114	2.33e-37

Genomic loci of PREs per predicted target gene (training set PREs: Kahn 2014 PREs)

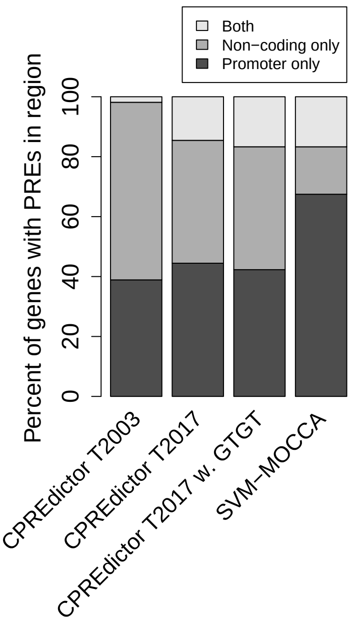

Training set PREs: Enderle 2011 PREs

Classifier comparison (training set PREs: Enderle 2011 PREs)

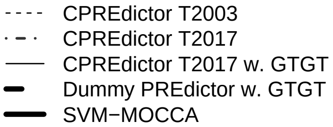

Classifier	Path
CPREdictor T2003	CPREdictor_M2003_T2003_mdBetween_wmPREdictor
CPREdictor T2017	CPREdictor_M2003_CPPREsEnderle_CND5merPREsEnderle_T110_mdBetween_wmPREdictor
CPREdictor T2017 w. GTGT	CPREdictor_M2003_GTGT_CPPREsEnderle_CND5merPREsEnderle_T110_mdBetween_wmPREdictor
Dummy PREdictor w. GTGT	DummyPREdictor_M2003_GTGT_T2003_mdBetween_wmPREdictor
SVM-MOCCA	SVMMOCCA_kquadratic_fnOcc_fDNT_M2003_GTGT_CPPREsEnderle_CNR5mers_CNCDS_CND5merPREsEnderle_T110_ws3000_mdBetween_wm[illegible]

Classifier comparison - training set PREs: Enderle 2011 PREs - Validation set: validation_PREsEnderle vs. validation_R5mers

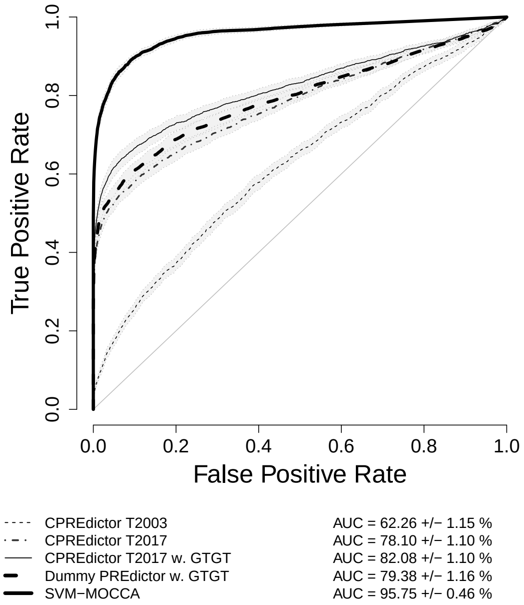

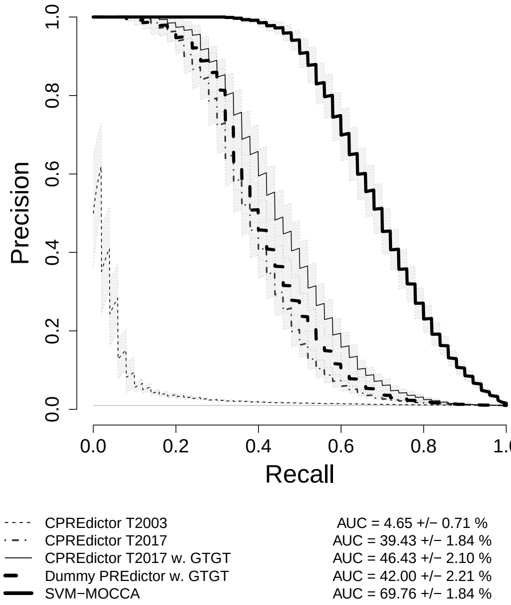

Classifier	ROC AUC
CPREdictor T2003	62.26 +/- 1.15 %
CPREdictor T2017	78.10 +/- 1.10 %
CPREdictor T2017 w. GTGT	82.08 +/- 1.10 %
Dummy PREdictor w. GTGT	79.38 +/- 1.16 %
SVM-MOCCA	95.75 +/- 0.46 %

Classifier	PRC AUC
CPREdictor T2003	4.65 +/- 0.71 %
CPREdictor T2017	39.43 +/- 1.84 %
CPREdictor T2017 w. GTGT	46.43 +/- 2.10 %
Dummy PREdictor w. GTGT	42.00 +/- 2.21 %
SVM-MOCCA	69.76 +/- 1.84 %

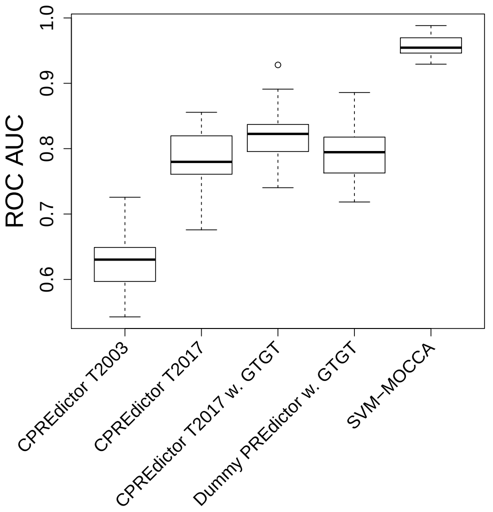

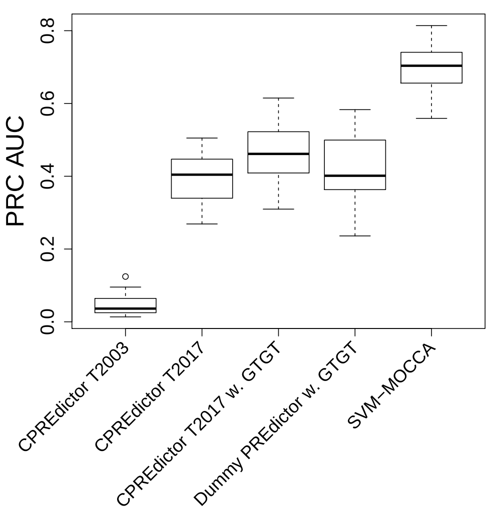

Classifier 1	Classifier 2	$p(H_0 : ROC1 <= ROC2)$	t	$p(H_0 : PRC1 <= PRC2)$	
CPREdictor T2003	CPREdictor T2003	-	-	-	
CPREdictor T2003	CPREdictor T2017	1.000000E+00	-2.581523E+01	1.000000E+00	-3
CPREdictor T2003	CPREdictor T2017 w. GTGT	1.000000E+00	-2.669433E+01	1.000000E+00	-3
CPREdictor T2003	Dummy PREdictor w. GTGT	1.000000E+00	-2.577178E+01	1.000000E+00	-3
CPREdictor T2003	SVM-MOCCA	1.000000E+00	-5.217079E+01	1.000000E+00	-6
CPREdictor T2017	CPREdictor T2003	1.743188E-30	2.581523E+01	1.727879E-36	3
CPREdictor T2017	CPREdictor T2017	-	-	-	
CPREdictor T2017	CPREdictor T2017 w. GTGT	1.000000E+00	-1.021228E+01	1.000000E+00	-1
CPREdictor T2017	Dummy PREdictor w. GTGT	9.972555E-01	-2.905522E+00	9.969462E-01	-2
CPREdictor T2017	SVM-MOCCA	1.000000E+00	-3.291903E+01	1.000000E+00	-3
CPREdictor T2017 w. GTGT	CPREdictor T2003	3.759482E-31	2.669433E+01	2.287931E-38	3
CPREdictor T2017 w. GTGT	CPREdictor T2017	4.988616E-14	1.021228E+01	1.448846E-16	1
CPREdictor T2017 w. GTGT	CPREdictor T2017 w. GTGT	-	-	-	
CPREdictor T2017 w. GTGT	Dummy PREdictor w. GTGT	3.726899E-10	7.614603E+00	3.725103E-09	6
CPREdictor T2017 w. GTGT	SVM-MOCCA	1.000000E+00	-2.774411E+01	1.000000E+00	-2
Dummy PREdictor w. GTGT	CPREdictor T2003	1.882710E-30	2.577178E+01	2.939501E-35	3
Dummy PREdictor w. GTGT	CPREdictor T2017	2.744498E-03	2.905522E+00	3.053839E-03	2
Dummy PREdictor w. GTGT	CPREdictor T2017 w. GTGT	1.000000E+00	-7.614603E+00	1.000000E+00	-6
Dummy PREdictor w. GTGT	Dummy PREdictor w. GTGT	-	-	-	
Dummy PREdictor w. GTGT	SVM-MOCCA	1.000000E+00	-3.018682E+01	1.000000E+00	-3
SVM-MOCCA	CPREdictor T2003	6.657781E-45	5.217079E+01	5.093567E-50	6
SVM-MOCCA	CPREdictor T2017	2.224113E-35	3.291903E+01	6.076326E-36	3
SVM-MOCCA	CPREdictor T2017 w. GTGT	6.371513E-32	2.774411E+01	1.175984E-30	2
SVM-MOCCA	Dummy PREdictor w. GTGT	1.276339E-33	3.018682E+01	7.684924E-34	3
SVM-MOCCA	SVM-MOCCA	-	-	-	

Classifier comparison - training set PREs: Enderle 2011 PREs - Validation set: validation_PREsEnderle vs. validation_CDS

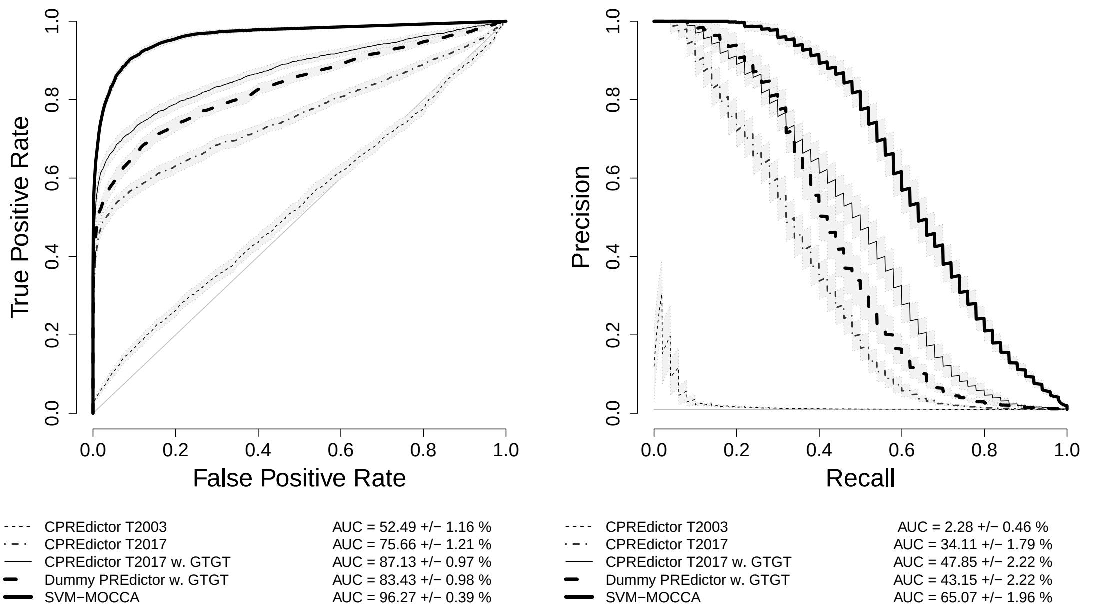

Classifier	ROC AUC
CPREdictor T2003	52.49 +/- 1.16 %
CPREdictor T2017	75.66 +/- 1.21 %
CPREdictor T2017 w. GTGT	87.13 +/- 0.97 %
Dummy PREdictor w. GTGT	83.43 +/- 0.98 %
SVM-MOCCA	96.27 +/- 0.39 %

Classifier	PRC AUC
CPREdictor T2003	2.28 +/- 0.46 %
CPREdictor T2017	34.11 +/- 1.79 %
CPREdictor T2017 w. GTGT	47.85 +/- 2.22 %
Dummy PREdictor w. GTGT	43.15 +/- 2.22 %
SVM-MOCCA	65.07 +/- 1.96 %

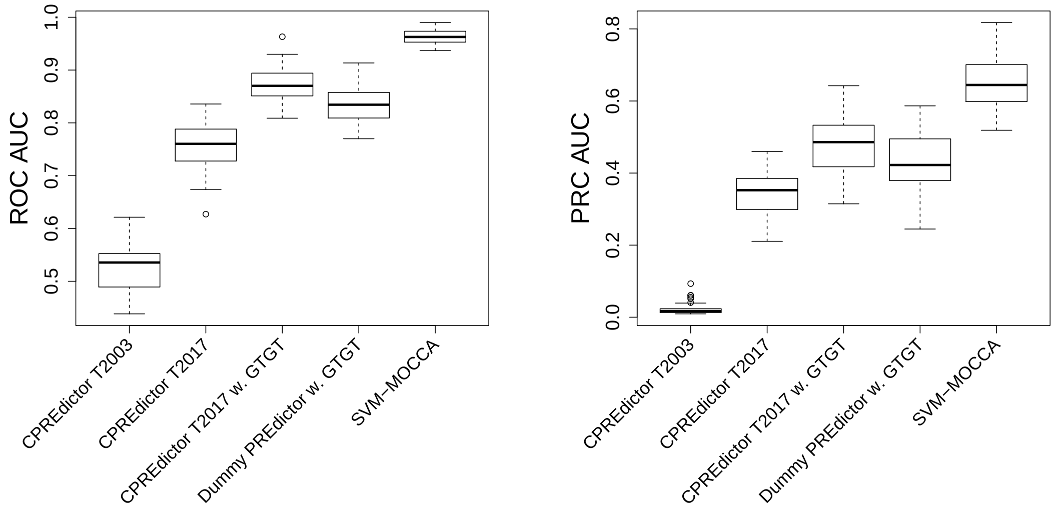

Classifier 1	Classifier 2	$p(H_0 : ROC1 <= ROC2)$	t	$p(H_0 : PRC1 <= PRC2)$	
CPREdictor T2003	CPREdictor T2003	-	-	-	
CPREdictor T2003	CPREdictor T2017	1.000000E+00	-3.515931E+01	1.000000E+00	-3
CPREdictor T2003	CPREdictor T2017 w. GTGT	1.000000E+00	-4.752368E+01	1.000000E+00	-3
CPREdictor T2003	Dummy PREdictor w. GTGT	1.000000E+00	-5.042644E+01	1.000000E+00	-3
CPREdictor T2003	SVM-MOCCA	1.000000E+00	-6.756786E+01	1.000000E+00	-6
CPREdictor T2017	CPREdictor T2003	1.004406E-36	3.515931E+01	5.522791E-36	3
CPREdictor T2017	CPREdictor T2017	-	-	-	
CPREdictor T2017	CPREdictor T2017 w. GTGT	1.000000E+00	-2.380013E+01	1.000000E+00	-2
CPREdictor T2017	Dummy PREdictor w. GTGT	1.000000E+00	-1.575899E+01	1.000000E+00	-1
CPREdictor T2017	SVM-MOCCA	1.000000E+00	-3.439950E+01	1.000000E+00	-3
CPREdictor T2017 w. GTGT	CPREdictor T2003	5.900720E-43	4.752368E+01	5.824459E-39	3
CPREdictor T2017 w. GTGT	CPREdictor T2017	7.006923E-29	2.380013E+01	2.666820E-27	2
CPREdictor T2017 w. GTGT	CPREdictor T2017 w. GTGT	-	-	-	
CPREdictor T2017 w. GTGT	Dummy PREdictor w. GTGT	2.595991E-14	1.041132E+01	1.160615E-08	6
CPREdictor T2017 w. GTGT	SVM-MOCCA	1.000000E+00	-2.069363E+01	1.000000E+00	-2
Dummy PREdictor w. GTGT	CPREdictor T2003	3.420092E-44	5.042644E+01	6.736878E-37	3
Dummy PREdictor w. GTGT	CPREdictor T2017	4.035713E-21	1.575899E+01	4.160677E-15	1
Dummy PREdictor w. GTGT	CPREdictor T2017 w. GTGT	1.000000E+00	-1.041132E+01	1.000000E+00	-6
Dummy PREdictor w. GTGT	Dummy PREdictor w. GTGT	-	-	-	
Dummy PREdictor w. GTGT	SVM-MOCCA	1.000000E+00	-2.853003E+01	1.000000E+00	-2
SVM-MOCCA	CPREdictor T2003	2.481809E-50	6.756786E+01	4.769452E-48	6
SVM-MOCCA	CPREdictor T2017	2.812448E-36	3.439950E+01	4.240463E-37	3
SVM-MOCCA	CPREdictor T2017 w. GTGT	3.607499E-26	2.069363E+01	1.755154E-28	2
SVM-MOCCA	Dummy PREdictor w. GTGT	1.753001E-32	2.853003E+01	2.986331E-33	2
SVM-MOCCA	SVM-MOCCA	-	-	-	

Classifier comparison - training set PREs: Enderle 2011 PREs - Validation set: validation_PREsEnderle vs. validation_D5merPREsEnderle

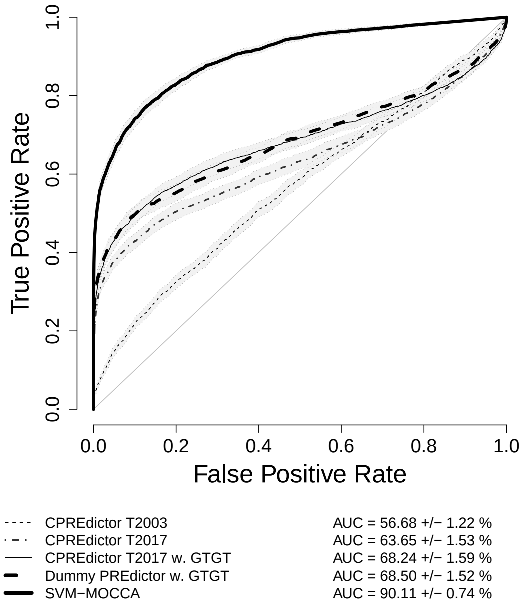

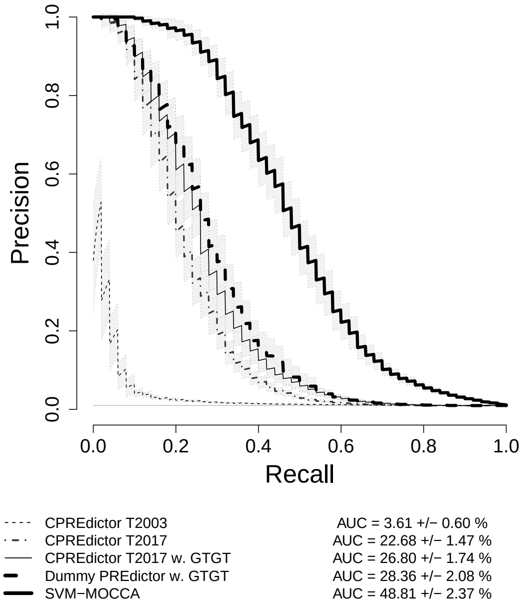

Classifier	ROC AUC
CPREdictor T2003	56.68 +/- 1.22 %
CPREdictor T2017	63.65 +/- 1.53 %
CPREdictor T2017 w. GTGT	68.24 +/- 1.59 %
Dummy PREdictor w. GTGT	68.50 +/- 1.52 %
SVM-MOCCA	90.11 +/- 0.74 %

Classifier	PRC AUC
CPREdictor T2003	3.61 +/- 0.60 %
CPREdictor T2017	22.68 +/- 1.47 %
CPREdictor T2017 w. GTGT	26.80 +/- 1.74 %
Dummy PREdictor w. GTGT	28.36 +/- 2.08 %
SVM-MOCCA	48.81 +/- 2.37 %

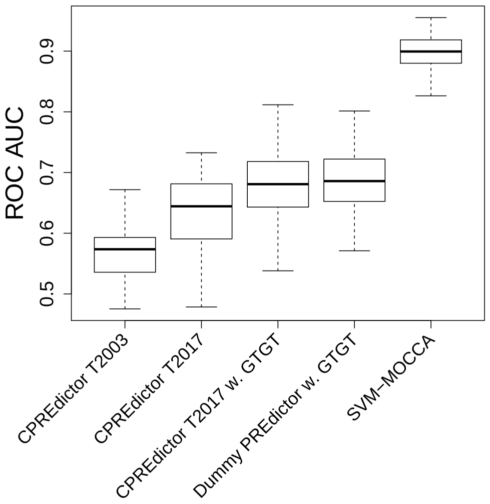

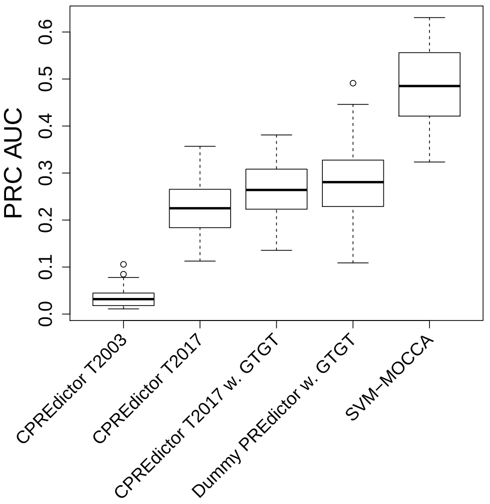

Classifier 1	Classifier 2	$p(H_0 : ROC1 <= ROC2)$	t	$p(H_0 : PRC1 <= PRC2)$	
CPREdictor T2003	CPREdictor T2003	-	-	-	
CPREdictor T2003	CPREdictor T2017	1.000000E+00	-9.059721E+00	1.000000E+00	-2
CPREdictor T2003	CPREdictor T2017 w. GTGT	1.000000E+00	-1.317518E+01	1.000000E+00	-2
CPREdictor T2003	Dummy PREdictor w. GTGT	1.000000E+00	-1.492384E+01	1.000000E+00	-2
CPREdictor T2003	SVM-MOCCA	1.000000E+00	-5.027716E+01	1.000000E+00	-3
CPREdictor T2017	CPREdictor T2003	2.402028E-12	9.059721E+00	4.715059E-29	2
CPREdictor T2017	CPREdictor T2017	-	-	-	
CPREdictor T2017	CPREdictor T2017 w. GTGT	1.000000E+00	-9.651591E+00	1.000000E+00	-1
CPREdictor T2017	Dummy PREdictor w. GTGT	1.000000E+00	-8.137244E+00	1.000000E+00	-7
CPREdictor T2017	SVM-MOCCA	1.000000E+00	-3.762940E+01	1.000000E+00	-2
CPREdictor T2017 w. GTGT	CPREdictor T2003	5.027455E-18	1.317518E+01	8.130997E-30	2
CPREdictor T2017 w. GTGT	CPREdictor T2017	3.223051E-13	9.651591E+00	2.733135E-15	1
CPREdictor T2017 w. GTGT	CPREdictor T2017 w. GTGT	-	-	-	
CPREdictor T2017 w. GTGT	Dummy PREdictor w. GTGT	7.643264E-01	-7.259098E-01	9.936873E-01	-2
CPREdictor T2017 w. GTGT	SVM-MOCCA	1.000000E+00	-3.216710E+01	1.000000E+00	-2
Dummy PREdictor w. GTGT	CPREdictor T2003	3.704281E-20	1.492384E+01	1.242518E-28	2
Dummy PREdictor w. GTGT	CPREdictor T2017	5.897036E-11	8.137244E+00	2.394364E-09	7
Dummy PREdictor w. GTGT	CPREdictor T2017 w. GTGT	2.356736E-01	7.259098E-01	6.312727E-03	2
Dummy PREdictor w. GTGT	Dummy PREdictor w. GTGT	-	-	-	
Dummy PREdictor w. GTGT	SVM-MOCCA	1.000000E+00	-3.327178E+01	1.000000E+00	-2
SVM-MOCCA	CPREdictor T2003	3.944189E-44	5.027716E+01	3.694824E-37	3
SVM-MOCCA	CPREdictor T2017	4.052051E-38	3.762940E+01	8.986204E-33	2
SVM-MOCCA	CPREdictor T2017 w. GTGT	6.569832E-35	3.216710E+01	4.561513E-31	2
SVM-MOCCA	Dummy PREdictor w. GTGT	1.348523E-35	3.327178E+01	5.327491E-28	2
SVM-MOCCA	SVM-MOCCA	-	-	-	

Numbers of candidate PREs (training set PREs: Enderle 2011 PREs)

All the following predictions are for an expected precision of 0.8.

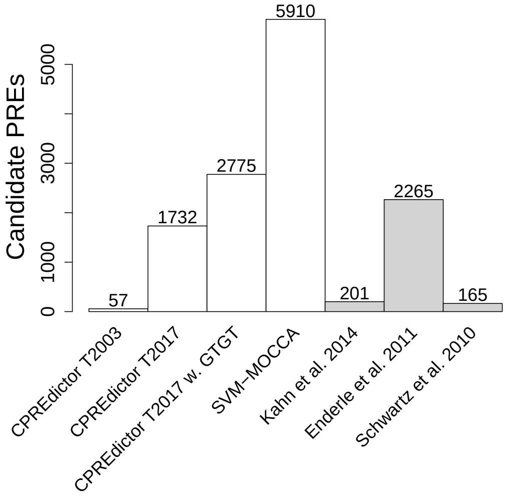

Set	Candidate PREs
CPREdictor T2003	57
CPREdictor T2017	1732
CPREdictor T2017 w. GTGT	2775
SVM-MOCCA	5910
Kahn et al. 2014	201
Enderle et al. 2011	2265
Schwartz et al. 2010	165

Repressed PRE Polycomb recruitment (training set PREs: Enderle 2011 PREs)

All the following are for an expected precision of 0.8.

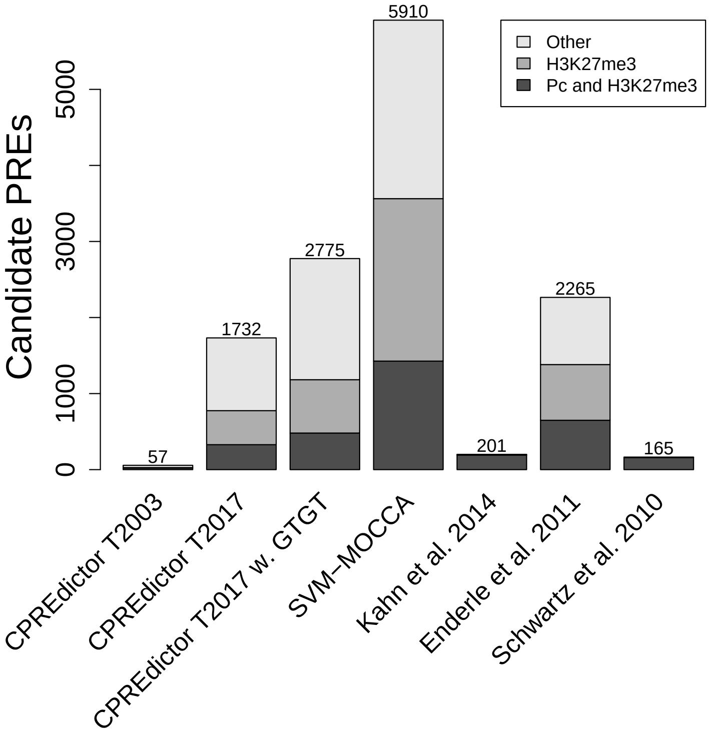

PRE set overlap sensitivity (training set PREs: Enderle 2011 PREs)

All the following are for an expected precision of 0.8.

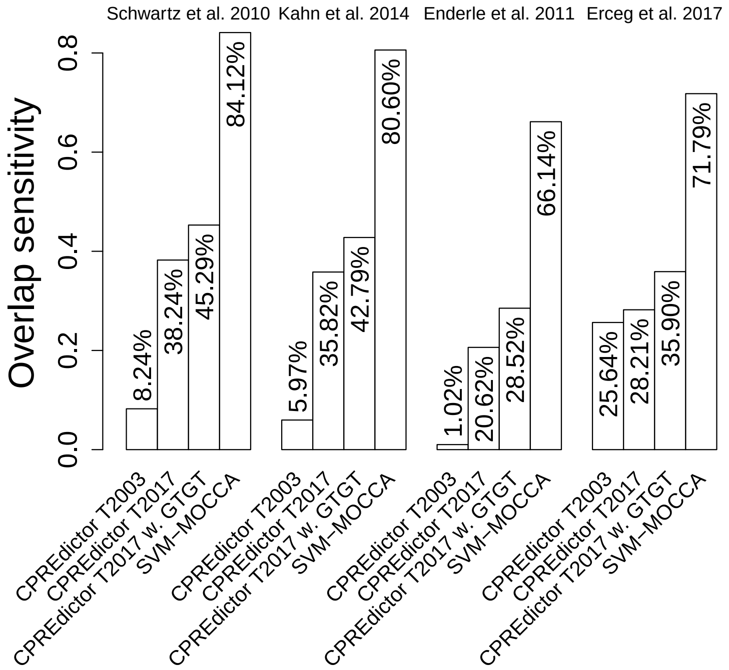

Overlap sensitivity	Kahn et al. 2014	Enderle et al. 2011	Schwartz et al. 2010
CPREdictor T2003	5.97 %	1.02 %	7.88 %
CPREdictor T2017	35.82 %	20.62 %	38.18 %
CPREdictor T2017 w. GTGT	42.79 %	28.52 %	45.45 %
SVM-MOCCA	80.60 %	66.14 %	83.64 %

PRE set overlap sensitivity (training set PREs: Enderle 2011 PREs)

All the following are for an expected precision of 0.8.

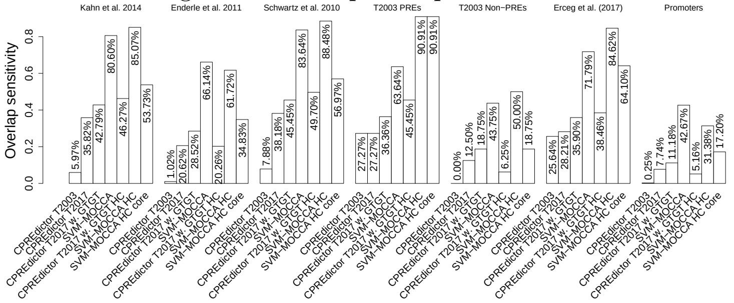

Overlap sensitivity	Kahn et al. 2014	Enderle et al. 2011	Schwartz et al. 2010	T2003 PREs	T200
CPREdictor T2003	5.97 %	1.02 %	7.88 %	27.27 %	
CPREdictor T2017	35.82 %	20.62 %	38.18 %	27.27 %	
CPREdictor T2017 w. GTGT	42.79 %	28.52 %	45.45 %	36.36 %	
SVM-MOCCA	80.60 %	66.14 %	83.64 %	63.64 %	
CPREdictor T2017 w. GTGT HC	46.27 %	20.26 %	49.70 %	45.45 %	
SVM-MOCCA HC	85.07 %	61.72 %	88.48 %	90.91 %	
SVM-MOCCA HC core	53.73 %	34.83 %	56.97 %	90.91 %	

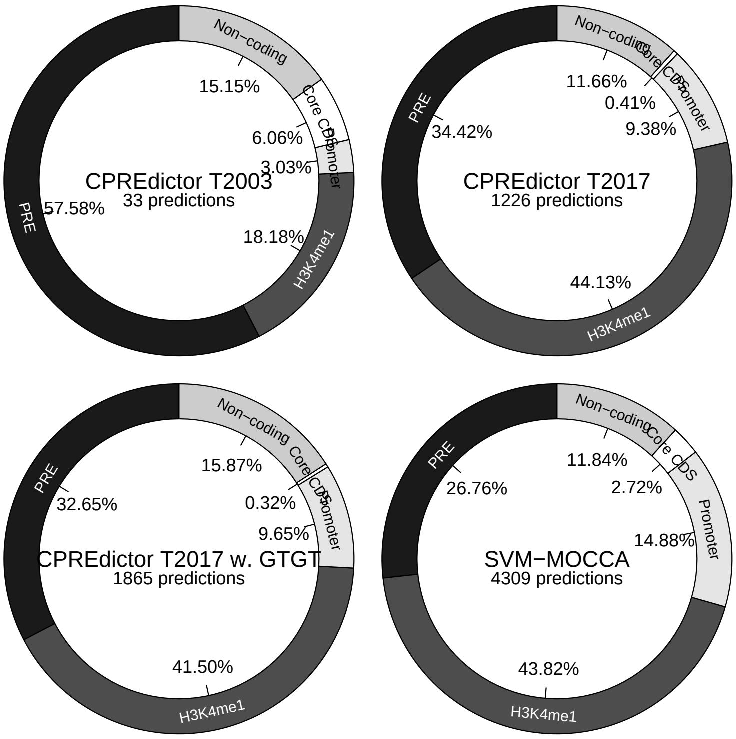

PcG/TrxG target genes (training set PREs: Enderle 2011 PREs)

All the following are for an expected precision of 0.8. * Kahn et al had not published any gene lists, so we predicted target genes ourselves based on computationally determined PREs. ** Where published sets of genes contained genes not found in the latest FlyBase annotation, we left those out.

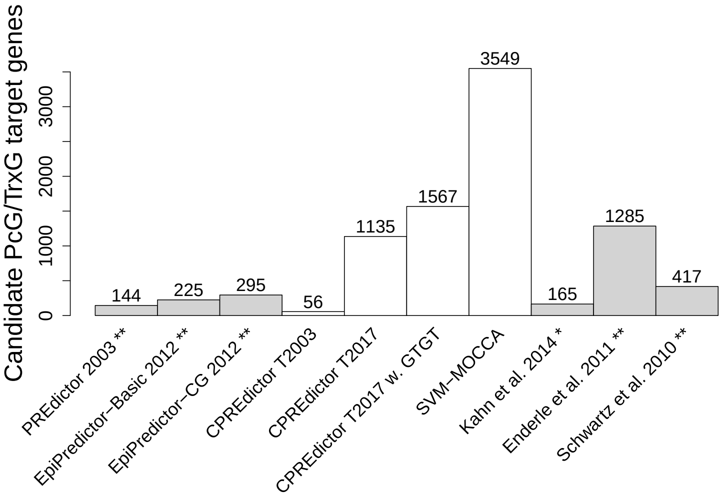

Set	Candidate PcG/TrxG target genes
PREdictor 2003 **	144
EpiPredictor-Basic 2012 **	225
EpiPredictor-CG 2012 **	295
CPREdictor T2003	56
CPREdictor T2017	1135
CPREdictor T2017 w. GTGT	1567
SVM-MOCCA	3549
Kahn et al. 2014 *	165
Enderle et al. 2011 **	1285
Schwartz et al. 2010 **	417

PcG/TrxG target gene sensitivity (training set PREs: Enderle 2011 PREs)

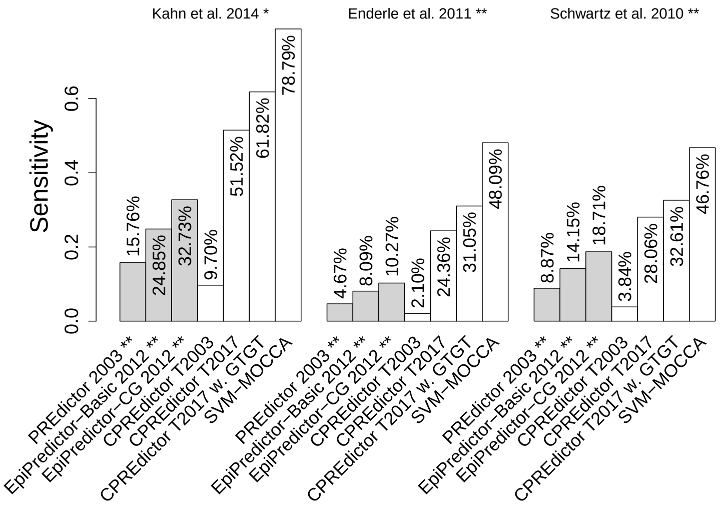

Sensitivity	Kahn et al. 2014 *	Enderle et al. 2011 **	Schwartz et al. 2010 **
PREdictor 2003 **	15.76 %	4.67 %	8.87 %
EpiPredictor-Basic 2012 **	24.85 %	8.09 %	14.15 %
EpiPredictor-CG 2012 **	32.73 %	10.27 %	18.71 %
CPREdictor T2003	9.70 %	2.10 %	3.84 %
CPREdictor T2017	51.52 %	24.36 %	28.06 %
CPREdictor T2017 w. GTGT	61.82 %	31.05 %	32.61 %
SVM-MOCCA	78.79 %	48.09 %	46.76 %

PcG/TrxG target gene precision (training set PREs: Enderle 2011 PREs)

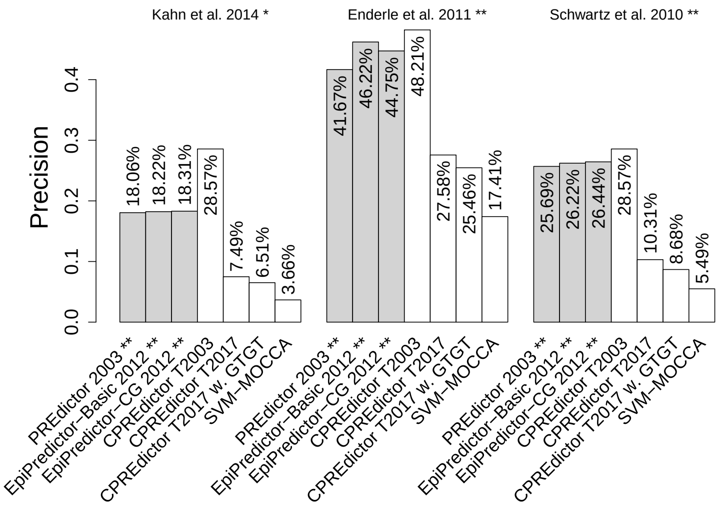

Precision	Kahn et al. 2014 *	Enderle et al. 2011 **	Schwartz et al. 2010 **
PREdictor 2003 **	18.06 %	41.67 %	25.69 %
EpiPredictor-Basic 2012 **	18.22 %	46.22 %	26.22 %
EpiPredictor-CG 2012 **	18.31 %	44.75 %	26.44 %
CPREdictor T2003	28.57 %	48.21 %	28.57 %
CPREdictor T2017	7.49 %	27.58 %	10.31 %
CPREdictor T2017 w. GTGT	6.51 %	25.46 %	8.68 %
SVM-MOCCA	3.66 %	17.41 %	5.49 %

PcG/TrxG target gene P-values (training set PREs: Enderle 2011 PREs)

P-value	Kahn et al. 2014 *	Enderle et al. 2011 **	Schwartz et al. 2010 **
PREdictor 2003 **	3.32e-26	1.24e-30	6.09e-28
EpiPredictor-Basic 2012 **	1.96e-41	7.25e-58	9.20e-45
EpiPredictor-CG 2012 **	3.27e-55	2.73e-71	1.13e-59
CPREdictor T2003	5.65e-20	1.73e-16	1.43e-13
CPREdictor T2017	1.04e-56	5.79e-105	5.89e-44
CPREdictor T2017 w. GTGT	7.46e-65	1.01e-124	3.89e-43
SVM-MOCCA	5.35e-59	1.95e-120	9.88e-35

Genomic loci of PREs per predicted target gene (training set PREs: Enderle 2011 PREs)

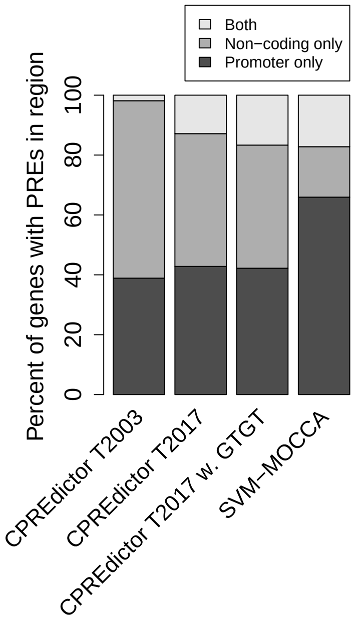

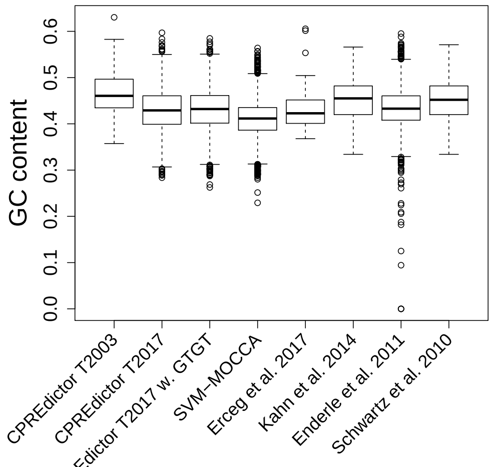

CPREdictor T2003	47.08	+/- 1.38 %
CPREdictor T2017	43.04	+/- 0.20 %
CPREdictor T2017 w. GTGT	43.16	+/- 0.15 %
SVM-MOCCA	41.01	+/- 0.09 %
Erceg et al. 2017	43.54	+/- 1.72 %
Kahn et al. 2014	45.13	+/- 0.64 %
Enderle et al. 2011	43.34	+/- 0.20 %
Schwartz et al. 2010	45.09	+/- 0.70 %
