# Supplementary material for: DNA sequence models of genome-wide Drosophila melanogaster Polycomb binding sites improve generalization to independent Polycomb Response Elements
Source: Nucleic Acids Res. 2019 Jul 24;47(15):7781–97. doi: 10.1093/nar/gkz617 (PMC6735708; doi:10.1093/nar/gkz617)

Gene ontology analysis

Bjørn André Bredesen, August 13, 2018

Contents

1 Gene ontology sets **2**
1.1 Schwartz2010HCClassI 2
1.2 Schwartz2010HCClassII 3
1.3 Enderle2011 4
1.4 PREdictor2003 6
1.5 EpiPredictorBasic2012 7
1.6 EpiPredictorCG2012 9
1.7 CPREdictor_T2017Schwartz_M2003_GTGT 10
1.8 SVMMOCCA_T2017SchwartzMC_M2003_GTGT 13

2 Gene ontology - Article **14**

3 Gene ontology - Article - CPREdictor T2017 M2003+GTGT (term in ? experimental/prediction sets) - Top 20 **15**

4 Gene ontology - Article - CPREdictor T2017 M2003+GTGT (term in ? experimental/prediction sets) - All **15**

5 Gene ontology - Article - SVM-MOCCA (term in ? experimental/prediction sets) - Top 20 **18**

6 Gene ontology - Article - SVM-MOCCA (term in ? experimental/prediction sets) - All **18**

Gene ontology sets

Schwartz2010HCClassI

Significantly enriched terms: 33

Name	p-value
Seq.-spec. DNA binding	2.150000E-73
DNA-binding T.F. activity	5.770000E-72
Transcription regulator activity	1.790000E-62
DNA-binding T.F. activity, RNA pol. II-specific	2.240000E-62
DNA binding	9.910000E-58
T.F. activity, RNA pol. II distal enhancer seq.-spec. binding	8.850000E-42
Reg. region nucleic acid binding	3.680000E-36
Transcription reg. region DNA binding	3.680000E-36
Transcription reg. region seq.-spec. DNA binding	6.220000E-35
Seq.-spec. double-stranded DNA binding	5.110000E-34
RNA pol. II reg. region seq.-spec. DNA binding	1.200000E-33
Nucleic acid binding	2.700000E-33
RNA pol. II reg. region DNA binding	5.010000E-33
Double-stranded DNA binding	9.610000E-32
Heterocyclic compound binding	2.340000E-23
Organic cyclic compound binding	4.060000E-23
Enhancer binding	1.940000E-21
DNA-binding transcription activator activity, RNA pol. II-specific	1.280000E-20
Enhancer seq.-spec. DNA binding	3.390000E-19
T.F. activity, RNA pol. II prox. promoter seq.-spec. DNA binding	6.420000E-19
Prox. promoter DNA-binding transcription activator activity, RNA pol. II-specific	5.100000E-14
RNA pol. II distal enhancer seq.-spec. DNA binding	2.730000E-12
Binding	2.980000E-12
RNA pol. II prox. promoter seq.-spec. DNA binding	5.790000E-08
Prox. promoter seq.-spec. DNA binding	9.710000E-08
DNA-binding transcription repressor activity, RNA pol. II-specific	1.750000E-07
Prox. promoter DNA-binding transcription repressor activity, RNA pol. II-specific	2.530000E-07
Morphogen activity	2.760000E-06
T.F. binding	1.460000E-05
Repressing T.F. binding	7.710000E-05
Imaginal disc growth factor receptor binding	1.750000E-04
Protein homodimerization activity	5.200000E-04
Distal enhancer DNA-binding transcription activator activity, RNA pol. II-specific	7.000000E-04

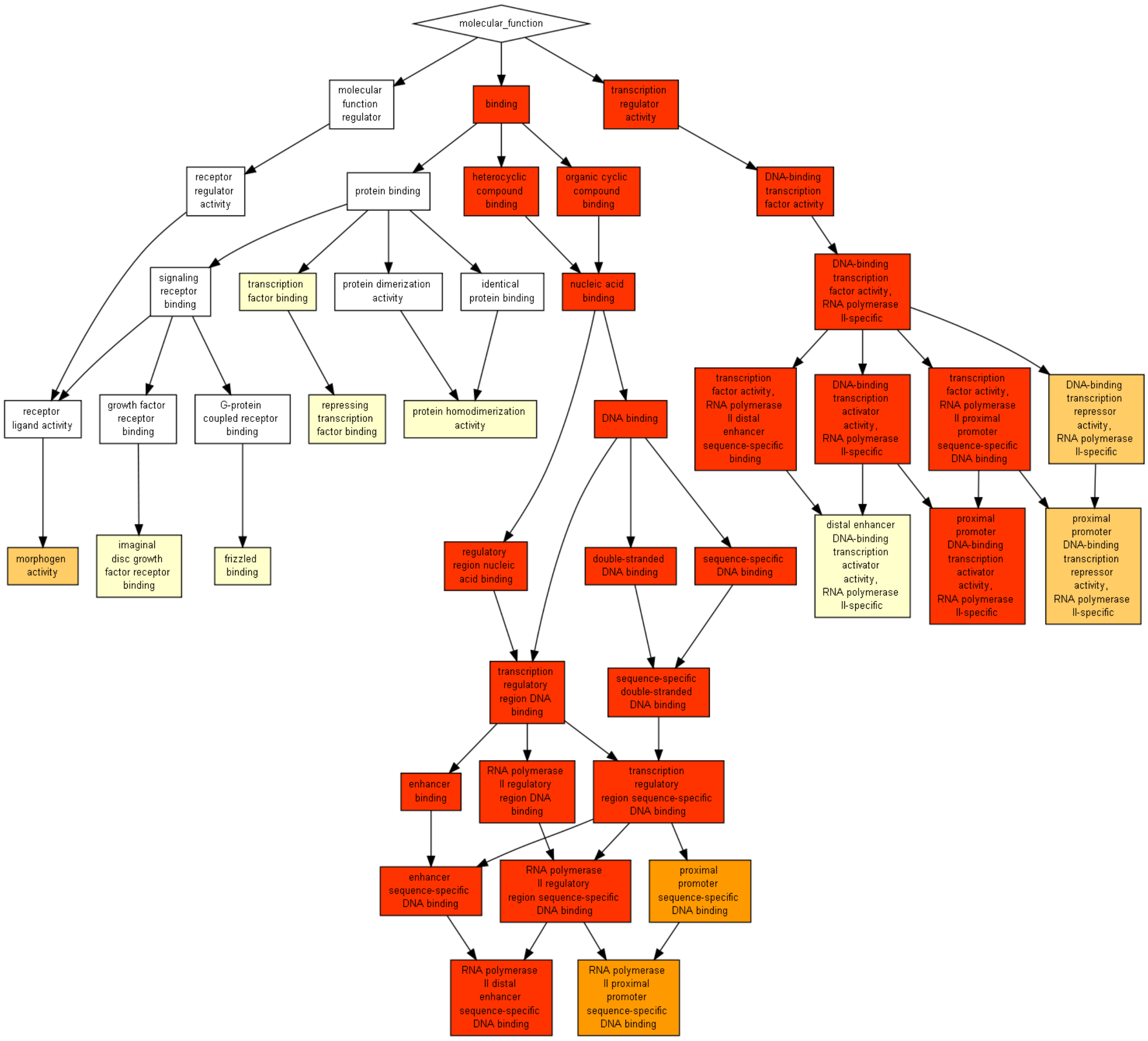

Schwartz2010HCClassII

Significantly enriched terms: 20

Name	p-value
Seq.-spec. DNA binding	1.570000E-10
DNA-binding T.F. activity	7.730000E-10
Transcription regulator activity	4.870000E-09
DNA-binding T.F. activity, RNA pol. II-specific	1.650000E-08
DNA binding	3.520000E-08
RNA pol. II reg. region seq.-spec. DNA binding	1.610000E-07
RNA pol. II reg. region DNA binding	2.210000E-07
Seq.-spec. double-stranded DNA binding	1.490000E-06
Reg. region nucleic acid binding	2.450000E-06
Transcription reg. region DNA binding	2.450000E-06
DNA-binding transcription activator activity, RNA pol. II-specific	3.350000E-06
Transcription reg. region seq.-spec. DNA binding	4.110000E-06
Double-stranded DNA binding	4.290000E-06
Cytokine activity	8.130000E-06
Peroxiredoxin activity	3.250000E-05
Phosphoenolpyruvate carboxykinase (GTP) activity	5.490000E-05
Phosphoenolpyruvate carboxykinase activity	5.490000E-05
T.F. activity, RNA pol. II distal enhancer seq.-spec. binding	8.780000E-05
T.F. activity, RNA pol. II prox. promoter seq.-spec. DNA binding	3.470000E-04
Peroxidase activity	8.170000E-04

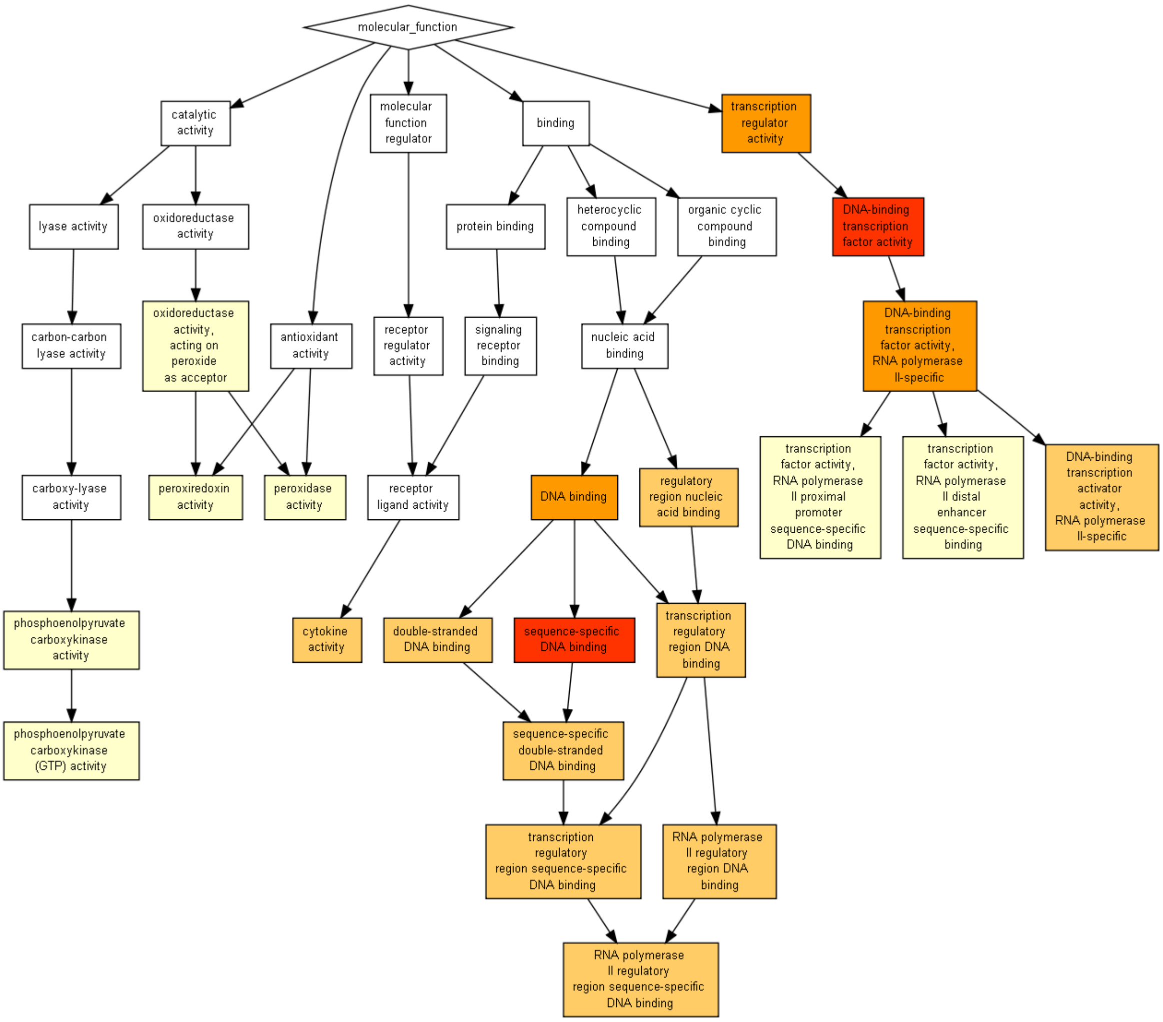

Enderle2011

Significantly enriched terms: 68

Name	p-value
DNA-binding T.F. activity	9.210000E-72
Seq.-spec. DNA binding	1.630000E-70
Transcription regulator activity	2.130000E-61
DNA-binding T.F. activity, RNA pol. II-specific	6.450000E-58
DNA binding	6.180000E-46
T.F. activity, RNA pol. II distal enhancer seq.-spec. binding	2.210000E-42
Binding	3.130000E-41
Protein binding	1.460000E-33
Reg. region nucleic acid binding	1.640000E-33
Transcription reg. region DNA binding	1.640000E-33
Seq.-spec. double-stranded DNA binding	9.820000E-33
Transcription reg. region seq.-spec. DNA binding	3.240000E-32
RNA pol. II reg. region seq.-spec. DNA binding	4.310000E-31
RNA pol. II reg. region DNA binding	9.260000E-31
Double-stranded DNA binding	9.720000E-30
Heterocyclic compound binding	3.620000E-28
Organic cyclic compound binding	7.010000E-28
Nucleic acid binding	8.380000E-24
DNA-binding transcription activator activity, RNA pol. II-specific	5.070000E-23
Enhancer binding	2.530000E-21
T.F. activity, RNA pol. II prox. promoter seq.-spec. DNA binding	4.910000E-21
Enhancer seq.-spec. DNA binding	1.180000E-20

Prox. promoter DNA-binding transcription activator activity, RNA pol. II-specific	1.620000E-17
RNA pol. II distal enhancer seq.-spec. DNA binding	8.580000E-15
T.F. binding	1.160000E-12
Signaling receptor binding	2.730000E-10
Identical protein binding	7.940000E-09
Protein homodimerization activity	6.000000E-08
Prox. promoter seq.-spec. DNA binding	7.310000E-08
RNA pol. II prox. promoter seq.-spec. DNA binding	3.930000E-07
Prox. promoter DNA-binding transcription repressor activity, RNA pol. II-specific	5.410000E-07
MRNA binding	5.900000E-07
DNA-binding transcription repressor activity, RNA pol. II-specific	9.800000E-07
Morphogen activity	1.270000E-06
Growth factor receptor binding	1.430000E-06
Protein domain specific binding	1.690000E-06
Repressing T.F. binding	3.580000E-06
RNA pol. II T.F. binding	6.900000E-06
Misfolded protein binding	2.300000E-05
Protein kinase binding	2.330000E-05
Protein dimerization activity	2.950000E-05
Anion binding	3.710000E-05
Ion binding	4.140000E-05
Steroid hormone receptor activity	4.470000E-05
Kinase binding	4.970000E-05
Receptor ligand activity	4.980000E-05
Distal enhancer DNA-binding transcription activator activity, RNA pol. II-specific	8.050000E-05
Receptor regulator activity	8.570000E-05
Heat shock protein binding	9.590000E-05
Purine ribonucleoside triphosphate binding	1.090000E-04
Enzyme binding	1.260000E-04
MRNA 3'-UTR binding	1.320000E-04
Nuclear receptor activity	1.390000E-04
T.F. activity, direct ligand regulated seq.-spec. DNA binding	1.390000E-04
Purine nucleotide binding	1.910000E-04
Actin binding	2.180000E-04
Ribonucleotide binding	2.500000E-04
Purine ribonucleotide binding	2.800000E-04
Nucleoside phosphate binding	3.140000E-04
Nucleotide binding	3.140000E-04
Protein binding involved in protein folding	3.580000E-04
Small molecule binding	3.930000E-04
Activating T.F. binding	3.970000E-04
ATP binding	3.990000E-04
Kinase activity	4.480000E-04
Cytoskeletal protein binding	5.020000E-04
Protein tyrosine kinase binding	5.380000E-04
Adenyl nucleotide binding	6.820000E-04

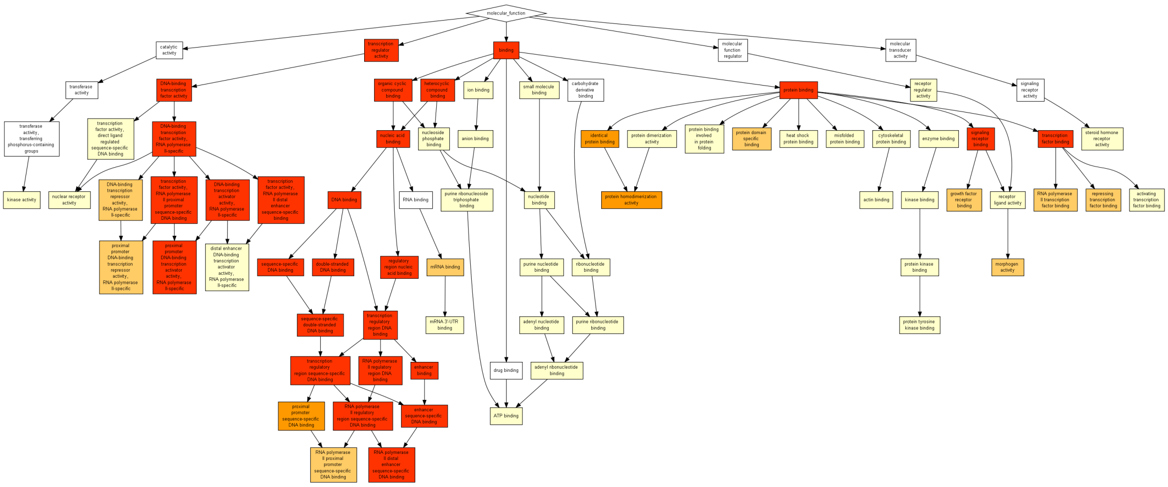

PREdictor2003

Significantly enriched terms: 26

Name	p-value
Seq.-spec. DNA binding	1.150000E-15
Transcription regulator activity	3.330000E-15
DNA-binding T.F. activity	4.680000E-15
DNA-binding T.F. activity, RNA pol. II-specific	3.830000E-13
T.F. activity, RNA pol. II distal enhancer seq.-spec. binding	6.280000E-13
Enhancer seq.-spec. DNA binding	1.820000E-12
Enhancer binding	5.670000E-12
DNA binding	4.000000E-09
RNA pol. II reg. region seq.-spec. DNA binding	5.640000E-09
RNA pol. II distal enhancer seq.-spec. DNA binding	6.140000E-09
Transcription reg. region seq.-spec. DNA binding	7.230000E-09
Reg. region nucleic acid binding	7.520000E-09
Transcription reg. region DNA binding	7.520000E-09
RNA pol. II reg. region DNA binding	8.420000E-09
DNA-binding transcription activator activity, RNA pol. II-specific	1.380000E-08
Double-stranded DNA binding	1.700000E-08
Seq.-spec. double-stranded DNA binding	2.610000E-08
T.F. activity, RNA pol. II prox. promoter seq.-spec. DNA binding	5.170000E-07
Nucleic acid binding	6.120000E-07
Binding	7.560000E-07
Prox. promoter DNA-binding transcription activator activity, RNA pol. II-specific	6.400000E-06
T.F. binding	7.320000E-06
Organic cyclic compound binding	9.050000E-06
Heterocyclic compound binding	1.810000E-05
Signaling receptor binding	6.270000E-05
Protein binding	4.990000E-04

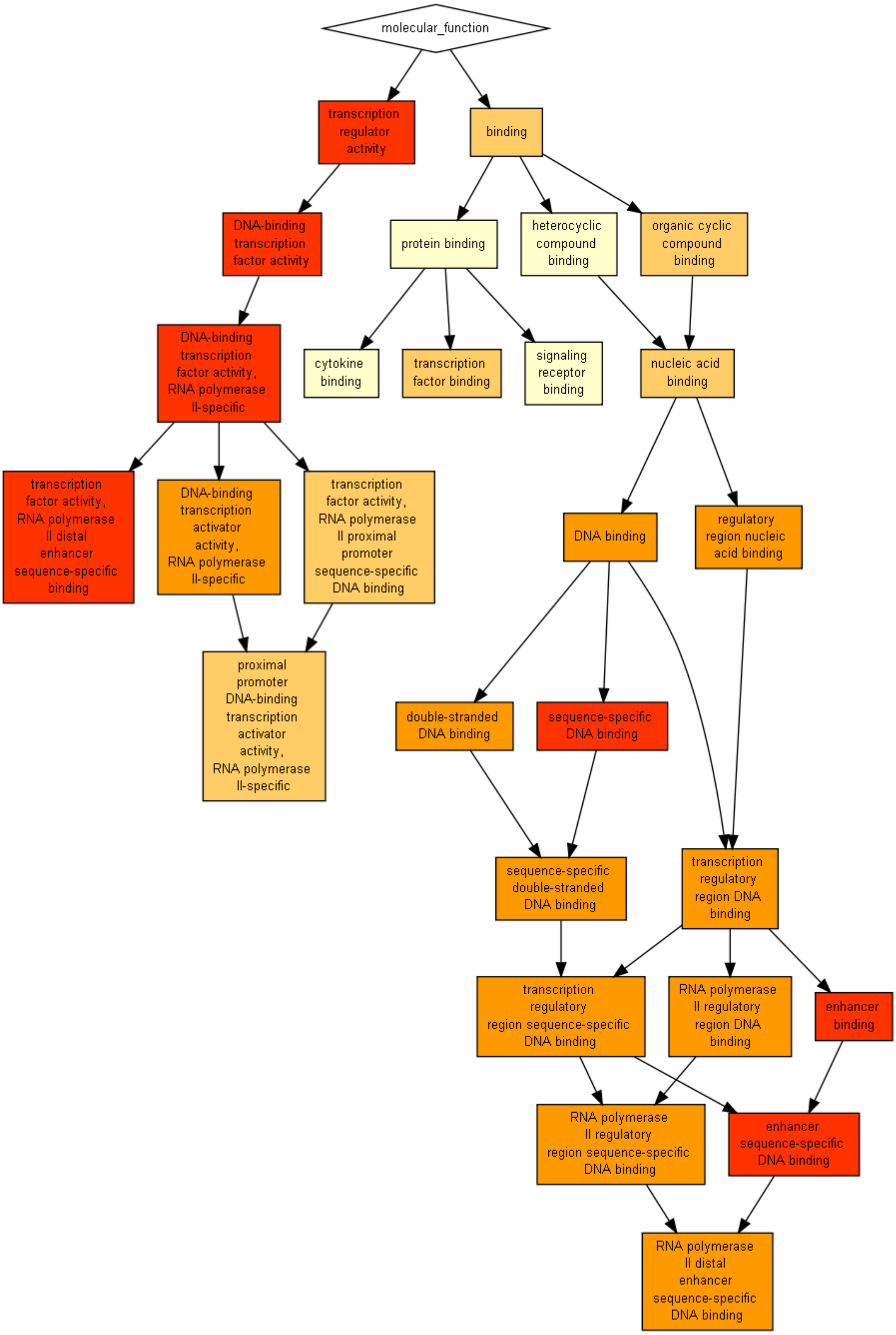

EpiPredictorBasic2012

Significantly enriched terms: 41

Name	p-value
Seq.-spec. DNA binding	1.560000E-25
DNA-binding T.F. activity	3.980000E-25
Transcription regulator activity	1.110000E-21
DNA binding	2.990000E-20
DNA-binding T.F. activity, RNA pol. II-specific	2.750000E-18
Nucleic acid binding	1.260000E-15
Reg. region nucleic acid binding	7.190000E-13
Transcription reg. region DNA binding	7.190000E-13
Enhancer seq.-spec. DNA binding	9.460000E-13
Transcription reg. region seq.-spec. DNA binding	1.170000E-12
T.F. activity, RNA pol. II distal enhancer seq.-spec. binding	2.300000E-12
RNA pol. II reg. region seq.-spec. DNA binding	2.790000E-12
Enhancer binding	3.790000E-12
RNA pol. II reg. region DNA binding	5.250000E-12
Binding	7.250000E-12
Seq.-spec. double-stranded DNA binding	9.370000E-12
Double-stranded DNA binding	1.620000E-11
RNA pol. II distal enhancer seq.-spec. DNA binding	1.700000E-11
DNA-binding transcription activator activity, RNA pol. II-specific	3.130000E-10
Organic cyclic compound binding	3.160000E-10
Heterocyclic compound binding	5.760000E-10
T.F. activity, RNA pol. II prox. promoter seq.-spec. DNA binding	5.070000E-08
Prox. promoter DNA-binding transcription activator activity, RNA pol. II-specific	4.100000E-07
Acetylcholine-gated cation-selective channel activity	5.590000E-06
Protein binding	1.820000E-05
Excitatory extracellular ligand-gated ion channel activity	3.530000E-05
Cytokine binding	5.440000E-05
Cation channel activity	1.090000E-04
Ion gated channel activity	1.490000E-04
Gated channel activity	1.780000E-04
Molecular transducer activity	2.750000E-04
Signaling receptor activity	3.390000E-04
Ion channel activity	3.660000E-04
Substrate-specific channel activity	3.990000E-04
Ligand-gated cation channel activity	5.340000E-04
Transmembrane signaling receptor activity	6.360000E-04
Passive transmembrane transporter activity	6.550000E-04
Channel activity	6.550000E-04
T.F. binding	9.350000E-04
Heparan sulfate proteoglycan binding	9.380000E-04
Proteoglycan binding	9.380000E-04

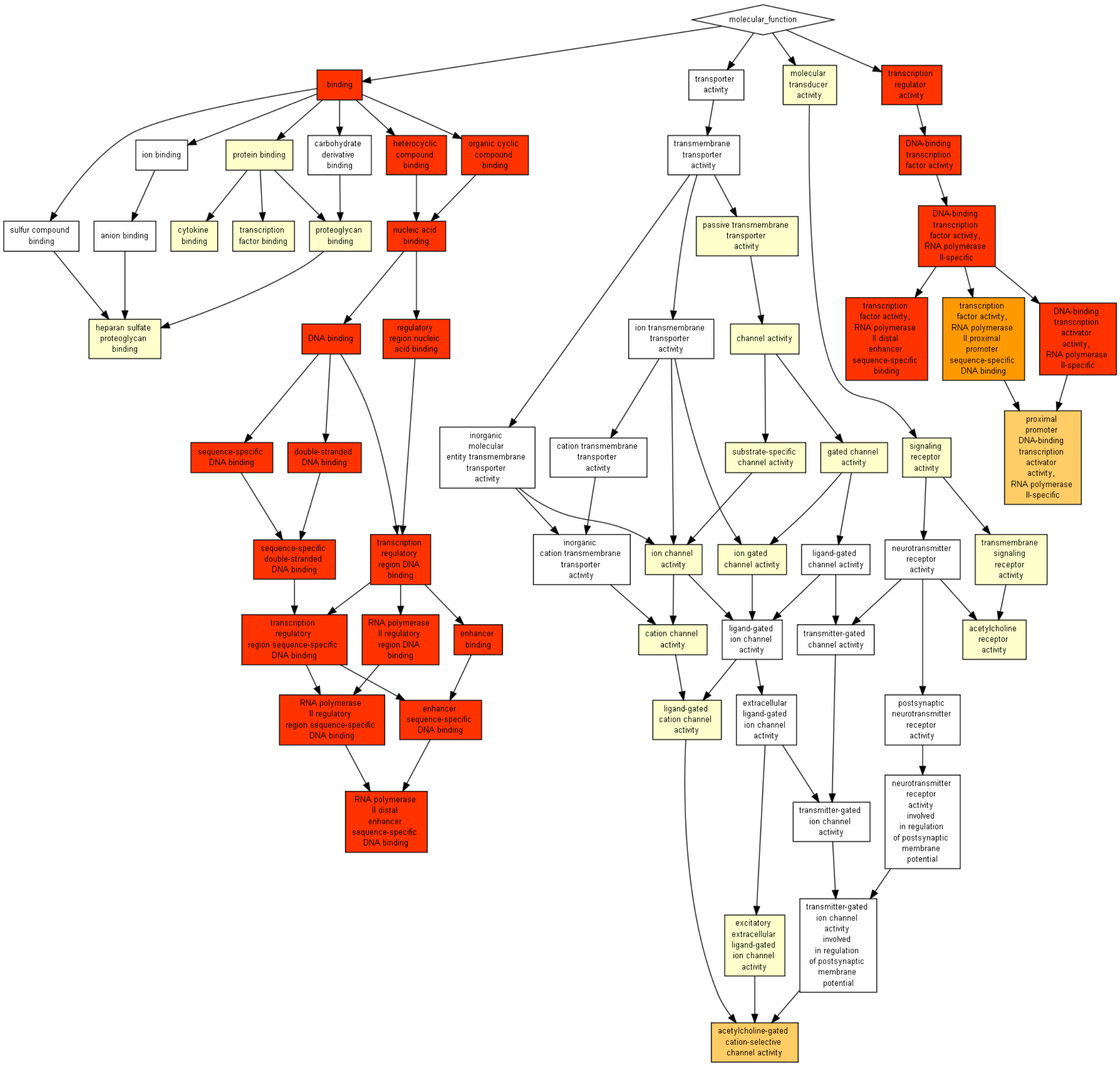

EpiPredictorCG2012

Significantly enriched terms: 52

Name	p-value
DNA-binding T.F. activity	7.750000E-31
Seq.-spec. DNA binding	3.570000E-30
Transcription regulator activity	4.050000E-26
DNA-binding T.F. activity, RNA pol. II-specific	6.400000E-24
DNA binding	6.430000E-22
Reg. region nucleic acid binding	8.910000E-15
Transcription reg. region DNA binding	8.910000E-15
T.F. activity, RNA pol. II distal enhancer seq.-spec. binding	4.000000E-14
DNA-binding transcription activator activity, RNA pol. II-specific	1.270000E-13
Transcription reg. region seq.-spec. DNA binding	2.150000E-13
Seq.-spec. double-stranded DNA binding	4.430000E-13
Nucleic acid binding	4.850000E-13
RNA pol. II reg. region seq.-spec. DNA binding	5.860000E-13
RNA pol. II reg. region DNA binding	1.220000E-12
Double-stranded DNA binding	1.380000E-12
Enhancer seq.-spec. DNA binding	1.490000E-12
T.F. activity, RNA pol. II prox. promoter seq.-spec. DNA binding	2.220000E-12
Binding	3.000000E-12
Enhancer binding	6.760000E-12

Prox. promoter DNA-binding transcription activator activity, RNA pol. II-specific	8.130000E-11
RNA pol. II distal enhancer seq.-spec. DNA binding	3.300000E-10
Organic cyclic compound binding	1.410000E-08
Heterocyclic compound binding	2.100000E-08
Protein binding	2.410000E-07
T.F. binding	3.390000E-06
G-protein coupled amine receptor activity	3.860000E-06
Molecular transducer activity	4.560000E-06
Signaling receptor activity	1.230000E-05
Acetylcholine-gated cation-selective channel activity	1.380000E-05
Protein homodimerization activity	1.890000E-05
Activating T.F. binding	1.930000E-05
G-protein coupled receptor activity	2.760000E-05
Transmembrane signaling receptor activity	3.760000E-05
Wnt-protein binding	5.190000E-05
Dopamine neurotransmitter receptor activity	5.430000E-05
Acetylcholine receptor activity	5.430000E-05
Cation channel activity	6.950000E-05
Inorganic cation transmembrane transporter activity	7.460000E-05
Identical protein binding	7.710000E-05
Ion gated channel activity	1.000000E-04
Neurotransmitter receptor activity	1.000000E-04
Gated channel activity	1.240000E-04
Cation transmembrane transporter activity	1.310000E-04
MRNA 3'-UTR binding	1.410000E-04
Excitatory extracellular ligand-gated ion channel activity	1.470000E-04
RNA pol. II T.F. binding	1.960000E-04
Morphogen activity	2.620000E-04
Wnt-activated receptor activity	4.500000E-04
Ion channel activity	5.380000E-04
Phosphorelay sensor kinase activity	5.780000E-04
Protein histidine kinase activity	5.780000E-04
Substrate-specific channel activity	5.920000E-04

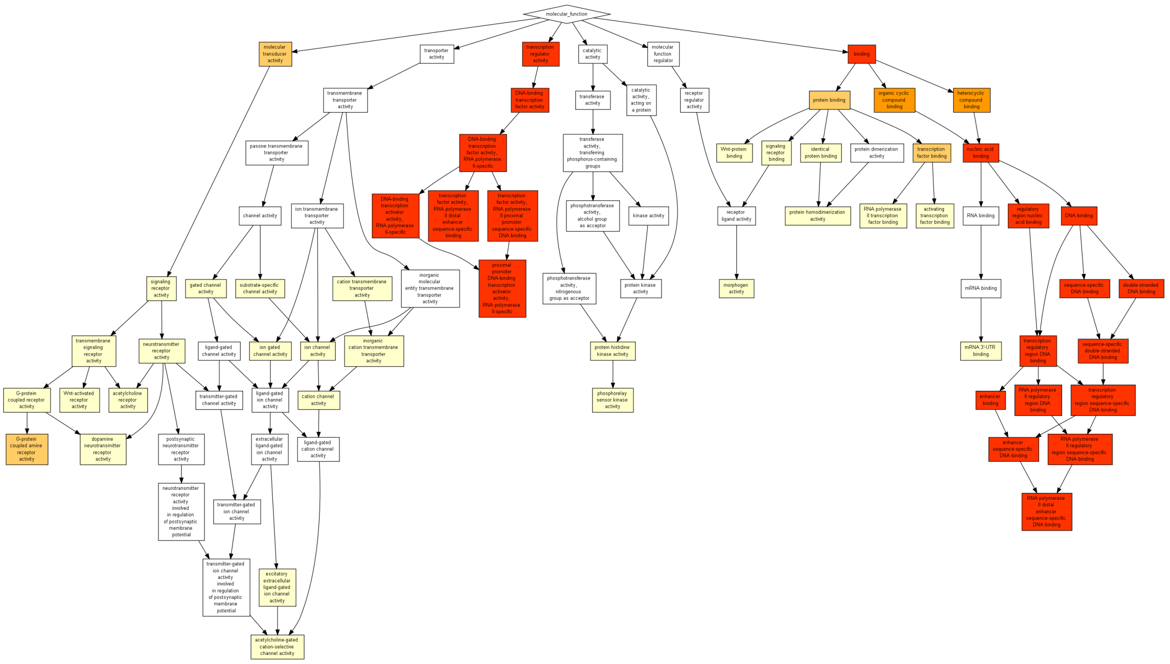

CPREdictor_T2017Schwartz_M2003_GTGT

Significantly enriched terms: 108

Name	p-value
Protein binding	1.180000E-42
DNA-binding T.F. activity	2.950000E-40
Transcription regulator activity	2.780000E-36
Seq.-spec. DNA binding	2.460000E-34
Binding	4.090000E-32
DNA-binding T.F. activity, RNA pol. II-specific	4.440000E-28
DNA binding	2.940000E-25
T.F. activity, RNA pol. II prox. promoter seq.-spec. DNA binding	8.710000E-18
Nucleosomal DNA binding	1.620000E-17
RNA pol. II reg. region seq.-spec. DNA binding	4.310000E-15
RNA pol. II reg. region DNA binding	5.790000E-15
Protein dimerization activity	1.530000E-14
Reg. region nucleic acid binding	3.000000E-14
Transcription reg. region DNA binding	3.000000E-14
Heterocyclic compound binding	3.240000E-14
Organic cyclic compound binding	5.340000E-14
Nucleic acid binding	2.770000E-13
Nucleosome binding	1.170000E-12
Seq.-spec. double-stranded DNA binding	1.320000E-12
Transcription reg. region seq.-spec. DNA binding	2.320000E-12
DNA-binding transcription repressor activity, RNA pol. II-specific	2.510000E-12
DNA-binding transcription activator activity, RNA pol. II-specific	1.800000E-11
Prox. promoter DNA-binding transcription repressor activity, RNA pol. II-specific	9.690000E-11
Double-stranded DNA binding	1.070000E-10
Protein kinase activity	2.940000E-10
T.F. binding	4.680000E-10
Prox. promoter DNA-binding transcription activator activity, RNA pol. II-specific	7.920000E-10
Protein-containing complex binding	9.260000E-10
Protein heterodimerization activity	1.400000E-09
Protein serine/threonine kinase activity	3.480000E-09
T.F. activity, RNA pol. II distal enhancer seq.-spec. binding	3.510000E-09
Kinase activity	5.950000E-09
Identical protein binding	1.010000E-08
Phosphotransferase activity, alcohol group as acceptor	1.790000E-08
Steroid hormone receptor activity	3.780000E-08
Molecular transducer activity	4.420000E-08
Chromatin binding	5.250000E-08
Protein homodimerization activity	5.390000E-08
Signaling receptor binding	1.360000E-07
Voltage-gated cation channel activity	2.090000E-07
T.F. activity, direct ligand regulated seq.-spec. DNA binding	2.750000E-07
Nuclear receptor activity	2.750000E-07
Actin binding	3.330000E-07
Enzyme binding	3.540000E-07
Cell adhesion molecule binding	6.280000E-07
Signaling receptor activity	6.690000E-07
Enhancer binding	9.340000E-07
Chromatin DNA binding	9.820000E-07
Enhancer seq.-spec. DNA binding	9.890000E-07
Repressing T.F. binding	1.050000E-06
Cation channel activity	1.160000E-06
Voltage-gated ion channel activity	2.360000E-06
Voltage-gated channel activity	2.360000E-06
Cytoskeletal protein binding	2.460000E-06
Rho GTPase binding	4.340000E-06
3',5'-cyclic-nucleotide phosphodiesterase activity	7.450000E-06
MRNA 3'-UTR binding	9.470000E-06
Calcium ion binding	1.240000E-05

Rho guanyl-nucleotide exchange factor activity	1.630000E-05
Potassium ion transmembrane transporter activity	1.740000E-05
G-protein coupled amine receptor activity	1.790000E-05
Cyclic-nucleotide phosphodiesterase activity	2.240000E-05
Ion binding	4.090000E-05
Transferase activity, transferring phosphorus-containing groups	4.500000E-05
Passive transmembrane transporter activity	6.340000E-05
Channel activity	6.340000E-05
MRNA binding	6.560000E-05
Protein kinase binding	7.040000E-05
Metal ion transmembrane transporter activity	8.000000E-05
RNA pol. II distal enhancer seq.-spec. DNA binding	9.740000E-05
Ion channel activity	1.180000E-04
Ribonucleotide binding	1.390000E-04
Substrate-specific channel activity	1.470000E-04
Kinase binding	1.560000E-04
Channel regulator activity	1.710000E-04
3',5'-cyclic-AMP phosphodiesterase activity	1.720000E-04
Ras guanyl-nucleotide exchange factor activity	1.920000E-04
Guanyl-nucleotide exchange factor activity	1.940000E-04
RNA pol. II prox. promoter seq.-spec. DNA binding	2.130000E-04
Purine ribonucleotide binding	2.280000E-04
Adenyl ribonucleotide binding	2.330000E-04
Translation repressor activity	2.340000E-04
ATP binding	2.440000E-04
Molecular function regulator	2.620000E-04
Purine ribonucleoside triphosphate binding	2.930000E-04
Adenyl nucleotide binding	2.960000E-04
Ligand-gated cation channel activity	3.260000E-04
Actin filament binding	3.370000E-04
Ion gated channel activity	3.370000E-04
Voltage-gated calcium channel activity	3.370000E-04
Purine nucleotide binding	3.680000E-04
Transmembrane signaling receptor activity	3.690000E-04
Translation regulator activity	4.090000E-04
Prox. promoter seq.-spec. DNA binding	4.140000E-04
Gated channel activity	5.010000E-04
Ras GTPase binding	5.240000E-04
GTPase binding	5.690000E-04
Calcium channel activity	5.770000E-04
Ion channel regulator activity	6.190000E-04
Mitogen-activated protein kinase kinase binding	6.860000E-04
Microtubule binding	6.980000E-04
Small GTPase binding	7.510000E-04
Potassium channel activity	7.550000E-04
Extracellular matrix binding	7.790000E-04
Cytokine binding	7.790000E-04
Collagen binding	7.790000E-04
3',5'-cyclic-GMP phosphodiesterase activity	7.790000E-04
Calcium ion transmembrane transporter activity	8.790000E-04

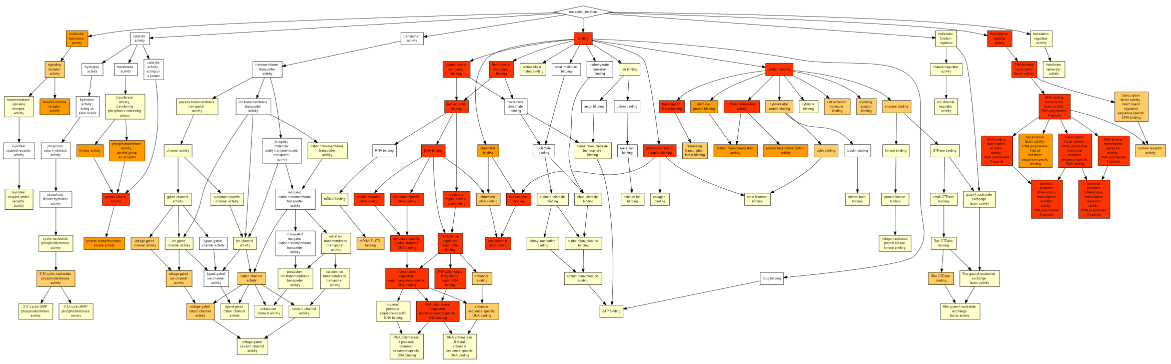

SVMMOCCA_T2017SchwartzMC_M2003_GTGT

Significantly enriched terms: 81

Name	p-value
DNA-binding T.F. activity	3.880000E-23
DNA-binding T.F. activity, RNA pol. II-specific	1.990000E-20
Seq.-spec. DNA binding	3.270000E-20
Protein binding	1.450000E-19
Transcription regulator activity	7.210000E-18
T.F. activity, RNA pol. II distal enhancer seq.-spec. binding	1.210000E-13
Binding	1.360000E-13
RNA pol. II reg. region seq.-spec. DNA binding	8.130000E-12
RNA pol. II reg. region DNA binding	9.340000E-12
Calcium ion binding	1.840000E-11
T.F. activity, RNA pol. II prox. promoter seq.-spec. DNA binding	1.520000E-10
Potassium ion transmembrane transporter activity	1.520000E-10
Reg. region nucleic acid binding	3.920000E-10
Transcription reg. region DNA binding	3.920000E-10
Cytoskeletal protein binding	1.050000E-09
Signaling receptor activity	2.370000E-09
Actin binding	3.710000E-09
Cell adhesion molecule binding	3.980000E-09
Protein kinase activity	4.190000E-09
DNA-binding transcription activator activity, RNA pol. II-specific	4.650000E-09
Transcription reg. region seq.-spec. DNA binding	4.940000E-09
Kinase activity	8.630000E-09
Molecular transducer activity	1.410000E-08
Metal ion transmembrane transporter activity	1.980000E-08
Enhancer binding	4.580000E-08
Seq.-spec. double-stranded DNA binding	4.600000E-08
Enhancer seq.-spec. DNA binding	6.730000E-08
DNA binding	1.290000E-07
Voltage-gated cation channel activity	1.380000E-07
Protein serine/threonine kinase activity	1.990000E-07
Cation channel activity	2.750000E-07
Signaling receptor binding	3.810000E-07
Phosphotransferase activity, alcohol group as acceptor	4.200000E-07
Double-stranded DNA binding	5.180000E-07
Cation transmembrane transporter activity	6.630000E-07
Ion transmembrane transporter activity	8.350000E-07
Voltage-gated ion channel activity	1.010000E-06
Voltage-gated channel activity	1.010000E-06
T.F. binding	1.390000E-06
G-protein coupled receptor activity	1.650000E-06
Calcium ion transmembrane transporter activity	1.850000E-06
Prox. promoter DNA-binding transcription activator activity, RNA pol. II-specific	2.050000E-06

Transmembrane signaling receptor activity	2.160000E-06
Transferase activity, transferring phosphorus-containing groups	2.710000E-06
Potassium channel activity	3.380000E-06
Ion channel activity	3.680000E-06
G-protein coupled amine receptor activity	4.040000E-06
DNA-binding transcription repressor activity, RNA pol. II-specific	4.270000E-06
Passive transmembrane transporter activity	4.950000E-06
Channel activity	4.950000E-06
Inorganic cation transmembrane transporter activity	5.270000E-06
Substrate-specific channel activity	5.710000E-06
RNA pol. II distal enhancer seq.-spec. DNA binding	6.540000E-06
Gated channel activity	7.610000E-06
Prox. promoter DNA-binding transcription repressor activity, RNA pol. II-specific	7.890000E-06
Calcium channel activity	1.010000E-05
Inorganic molecular entity transmembrane transporter activity	1.030000E-05
Voltage-gated potassium channel activity	1.170000E-05
Steroid hormone receptor activity	1.320000E-05
T.F. activity, direct ligand regulated seq.-spec. DNA binding	1.320000E-05
Nuclear receptor activity	1.320000E-05
Rho guanyl-nucleotide exchange factor activity	3.630000E-05
Ion gated channel activity	3.790000E-05
Ion binding	1.060000E-04
Protein homodimerization activity	1.290000E-04
Identical protein binding	1.320000E-04
Microtubule binding	1.400000E-04
Ras guanyl-nucleotide exchange factor activity	1.470000E-04
Actin filament binding	1.610000E-04
Ligand-gated cation channel activity	2.150000E-04
Neurotransmitter receptor activity	3.130000E-04
Cation binding	3.650000E-04
Cadherin binding	4.110000E-04
Metal ion binding	4.460000E-04
MRNA 3'-UTR binding	4.730000E-04
Rho GTPase binding	4.980000E-04
Guanyl-nucleotide exchange factor activity	6.740000E-04
Tubulin binding	6.980000E-04
Repressing T.F. binding	7.430000E-04
Transmembrane transporter activity	8.010000E-04
Neuropeptide receptor activity	8.740000E-04

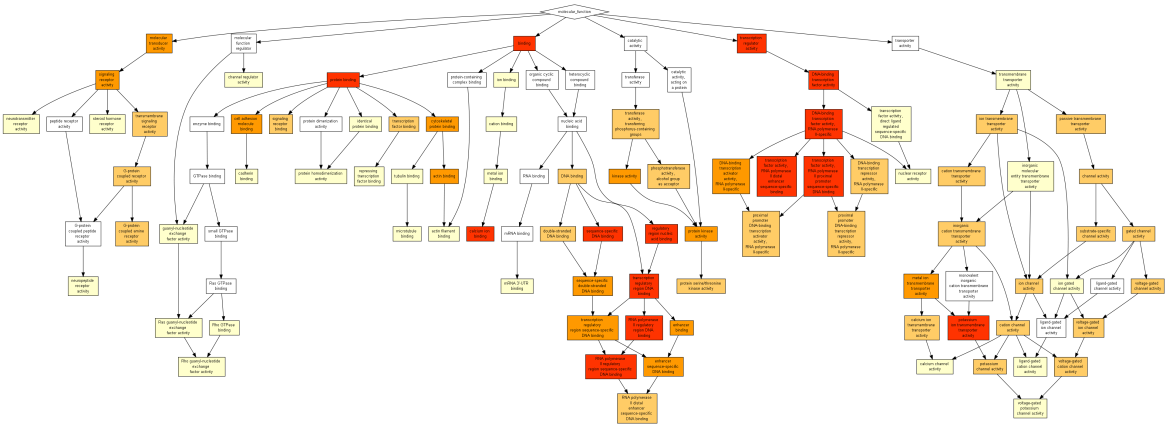

Gene ontology - Article

Set name:	SVMMOCCA_T2017SchwartzMC_M2003_GTGT
Significantly enriched terms:	81

Function (top 20)	p-value
DNA-binding T.F. activity	3.880000E-23
DNA-binding T.F. activity, RNA pol. II-specific	1.990000E-20
Seq.-spec. DNA binding	3.270000E-20
Protein binding	1.450000E-19
Transcription regulator activity	7.210000E-18
T.F. activity, RNA pol. II distal enhancer seq.-spec. binding	1.210000E-13
Binding	1.360000E-13
RNA pol. II reg. region seq.-spec. DNA binding	8.130000E-12
RNA pol. II reg. region DNA binding	9.340000E-12
Calcium ion binding	1.840000E-11
T.F. activity, RNA pol. II prox. promoter seq.-spec. DNA binding	1.520000E-10
Potassium ion transmembrane transporter activity	1.520000E-10
Reg. region nucleic acid binding	3.920000E-10
Transcription reg. region DNA binding	3.920000E-10
Cytoskeletal protein binding	1.050000E-09
Signaling receptor activity	2.370000E-09
Actin binding	3.710000E-09
Cell adhesion molecule binding	3.980000E-09
Protein kinase activity	4.190000E-09
DNA-binding transcription activator activity, RNA pol. II-specific	4.650000E-09

Gene ontology - Article - CPREdictor T2017 M2003+GTGT (term in ? experimental/prediction sets) - Top 20

Set name:	CPREdictor_T2017Schwartz_M2003_GTGT
Significantly enriched terms:	108

Function (top 20)	p-value	Validated *	Predicted **
Protein binding	1.180000E-42	1/3 sets	3/3 sets
DNA-binding T.F. activity	2.950000E-40	3/3 sets	3/3 sets
Transcription regulator activity	2.780000E-36	3/3 sets	3/3 sets
Seq.-spec. DNA binding	2.460000E-34	3/3 sets	3/3 sets
Binding	4.090000E-32	2/3 sets	3/3 sets
DNA-binding T.F. activity, RNA pol. II-specific	4.440000E-28	3/3 sets	3/3 sets
DNA binding	2.940000E-25	3/3 sets	3/3 sets
T.F. activity, RNA pol. II prox. promoter seq.-spec. DNA binding	8.710000E-18	3/3 sets	3/3 sets
Nucleosomal DNA binding	1.620000E-17	0/3 sets	0/3 sets
RNA pol. II reg. region seq.-spec. DNA binding	4.310000E-15	3/3 sets	3/3 sets
RNA pol. II reg. region DNA binding	5.790000E-15	3/3 sets	3/3 sets
Protein dimerization activity	1.530000E-14	1/3 sets	0/3 sets
Reg. region nucleic acid binding	3.000000E-14	3/3 sets	3/3 sets
Transcription reg. region DNA binding	3.000000E-14	3/3 sets	3/3 sets
Heterocyclic compound binding	3.240000E-14	2/3 sets	3/3 sets
Organic cyclic compound binding	5.340000E-14	2/3 sets	3/3 sets
Nucleic acid binding	2.770000E-13	2/3 sets	3/3 sets
Nucleosome binding	1.170000E-12	0/3 sets	0/3 sets
Seq.-spec. double-stranded DNA binding	1.320000E-12	3/3 sets	3/3 sets
Transcription reg. region seq.-spec. DNA binding	2.320000E-12	3/3 sets	3/3 sets

* Schwartz *et al.* (2010) HC Class I/II, Enderle *et al.* 2011
* Published predictions: PREdictor (2003), EpiPredictor Basic(2012), EpiPredictor CG (2012)

Gene ontology - Article - CPREdictor T2017 M2003+GTGT (term in ? experimental/prediction sets) - All

Set name:	CPREdictor_T2017Schwartz_M2003_GTGT
Significantly enriched terms:	108

Function (all)	p-value	Validated *	Predicted **
Protein binding	1.180000E-42	1/3 sets	3/3 sets
DNA-binding T.F. activity	2.950000E-40	3/3 sets	3/3 sets
Transcription regulator activity	2.780000E-36	3/3 sets	3/3 sets
Seq.-spec. DNA binding	2.460000E-34	3/3 sets	3/3 sets
Binding	4.090000E-32	2/3 sets	3/3 sets
DNA-binding T.F. activity, RNA pol. II-specific	4.440000E-28	3/3 sets	3/3 sets
DNA binding	2.940000E-25	3/3 sets	3/3 sets
T.F. activity, RNA pol. II prox. promoter seq.-spec. DNA binding	8.710000E-18	3/3 sets	3/3 sets
Nucleosomal DNA binding	1.620000E-17	0/3 sets	0/3 sets
RNA pol. II reg. region seq.-spec. DNA binding	4.310000E-15	3/3 sets	3/3 sets
RNA pol. II reg. region DNA binding	5.790000E-15	3/3 sets	3/3 sets
Protein dimerization activity	1.530000E-14	1/3 sets	0/3 sets
Reg. region nucleic acid binding	3.000000E-14	3/3 sets	3/3 sets
Transcription reg. region DNA binding	3.000000E-14	3/3 sets	3/3 sets
Heterocyclic compound binding	3.240000E-14	2/3 sets	3/3 sets
Organic cyclic compound binding	5.340000E-14	2/3 sets	3/3 sets
Nucleic acid binding	2.770000E-13	2/3 sets	3/3 sets
Nucleosome binding	1.170000E-12	0/3 sets	0/3 sets
Seq.-spec. double-stranded DNA binding	1.320000E-12	3/3 sets	3/3 sets
Transcription reg. region seq.-spec. DNA binding	2.320000E-12	3/3 sets	3/3 sets
DNA-binding transcription repressor activity, RNA pol. II-specific	2.510000E-12	2/3 sets	0/3 sets
DNA-binding transcription activator activity, RNA pol. II-specific	1.800000E-11	3/3 sets	3/3 sets
Prox. promoter DNA-binding transcription repressor activity, RNA pol. II-specific	9.690000E-11	2/3 sets	0/3 sets
Double-stranded DNA binding	1.070000E-10	3/3 sets	3/3 sets
Protein kinase activity	2.940000E-10	0/3 sets	0/3 sets
T.F. binding	4.680000E-10	2/3 sets	3/3 sets
Prox. promoter DNA-binding transcription activator activity, RNA pol. II-specific	7.920000E-10	2/3 sets	3/3 sets
Protein-containing complex binding	9.260000E-10	0/3 sets	0/3 sets
Protein heterodimerization activity	1.400000E-09	0/3 sets	0/3 sets
Protein serine/threonine kinase activity	3.480000E-09	0/3 sets	0/3 sets
T.F. activity, RNA pol. II distal enhancer seq.-spec. binding	3.510000E-09	3/3 sets	3/3 sets
Kinase activity	5.950000E-09	1/3 sets	0/3 sets
Identical protein binding	1.010000E-08	1/3 sets	1/3 sets
Phosphotransferase activity, alcohol group as acceptor	1.790000E-08	0/3 sets	0/3 sets
Steroid hormone receptor activity	3.780000E-08	1/3 sets	0/3 sets
Molecular transducer activity	4.420000E-08	0/3 sets	2/3 sets
Chromatin binding	5.250000E-08	0/3 sets	0/3 sets
Protein homodimerization activity	5.390000E-08	2/3 sets	1/3 sets
Signaling receptor binding	1.360000E-07	1/3 sets	1/3 sets
Voltage-gated cation channel activity	2.090000E-07	0/3 sets	0/3 sets
T.F. activity, direct ligand regulated seq.-spec. DNA binding	2.750000E-07	1/3 sets	0/3 sets
Nuclear receptor activity	2.750000E-07	1/3 sets	0/3 sets
Actin binding	3.330000E-07	1/3 sets	0/3 sets
Enzyme binding	3.540000E-07	1/3 sets	0/3 sets
Cell adhesion molecule binding	6.280000E-07	0/3 sets	0/3 sets
Signaling receptor activity	6.690000E-07	0/3 sets	2/3 sets
Enhancer binding	9.340000E-07	2/3 sets	3/3 sets
Chromatin DNA binding	9.820000E-07	0/3 sets	0/3 sets
Enhancer seq.-spec. DNA binding	9.890000E-07	2/3 sets	3/3 sets
Repressing T.F. binding	1.050000E-06	2/3 sets	0/3 sets
Cation channel activity	1.160000E-06	0/3 sets	2/3 sets

Voltage-gated ion channel activity	2.360000E-06	0/3 sets	0/3 sets
Voltage-gated channel activity	2.360000E-06	0/3 sets	0/3 sets
Cytoskeletal protein binding	2.460000E-06	1/3 sets	0/3 sets
Rho GTPase binding	4.340000E-06	0/3 sets	0/3 sets
3',5'-cyclic-nucleotide phosphodiesterase activity	7.450000E-06	0/3 sets	0/3 sets
MRNA 3'-UTR binding	9.470000E-06	1/3 sets	1/3 sets
Calcium ion binding	1.240000E-05	0/3 sets	0/3 sets
Rho guanyl-nucleotide exchange factor activity	1.630000E-05	0/3 sets	0/3 sets
Potassium ion transmembrane transporter activity	1.740000E-05	0/3 sets	0/3 sets
G-protein coupled amine receptor activity	1.790000E-05	0/3 sets	1/3 sets
Cyclic-nucleotide phosphodiesterase activity	2.240000E-05	0/3 sets	0/3 sets
Ion binding	4.090000E-05	1/3 sets	0/3 sets
Transferase activity, transferring phosphorus-containing groups	4.500000E-05	0/3 sets	0/3 sets
Passive transmembrane transporter activity	6.340000E-05	0/3 sets	1/3 sets
Channel activity	6.340000E-05	0/3 sets	1/3 sets
MRNA binding	6.560000E-05	1/3 sets	0/3 sets
Protein kinase binding	7.040000E-05	1/3 sets	0/3 sets
Metal ion transmembrane transporter activity	8.000000E-05	0/3 sets	0/3 sets
RNA pol. II distal enhancer seq.-spec. DNA binding	9.740000E-05	2/3 sets	3/3 sets
Ion channel activity	1.180000E-04	0/3 sets	2/3 sets
Ribonucleotide binding	1.390000E-04	1/3 sets	0/3 sets
Substrate-specific channel activity	1.470000E-04	0/3 sets	2/3 sets
Kinase binding	1.560000E-04	1/3 sets	0/3 sets
Channel regulator activity	1.710000E-04	0/3 sets	0/3 sets
3',5'-cyclic-AMP phosphodiesterase activity	1.720000E-04	0/3 sets	0/3 sets
Ras guanyl-nucleotide exchange factor activity	1.920000E-04	0/3 sets	0/3 sets
Guanyl-nucleotide exchange factor activity	1.940000E-04	0/3 sets	0/3 sets
RNA pol. II prox. promoter seq.-spec. DNA binding	2.130000E-04	2/3 sets	0/3 sets
Purine ribonucleotide binding	2.280000E-04	1/3 sets	0/3 sets
Adenyl ribonucleotide binding	2.330000E-04	0/3 sets	0/3 sets
Translation repressor activity	2.340000E-04	0/3 sets	0/3 sets
ATP binding	2.440000E-04	1/3 sets	0/3 sets
Molecular function regulator	2.620000E-04	0/3 sets	0/3 sets
Purine ribonucleoside triphosphate binding	2.930000E-04	1/3 sets	0/3 sets
Adenyl nucleotide binding	2.960000E-04	1/3 sets	0/3 sets
Ligand-gated cation channel activity	3.260000E-04	0/3 sets	1/3 sets
Actin filament binding	3.370000E-04	0/3 sets	0/3 sets
Ion gated channel activity	3.370000E-04	0/3 sets	2/3 sets
Voltage-gated calcium channel activity	3.370000E-04	0/3 sets	0/3 sets
Purine nucleotide binding	3.680000E-04	1/3 sets	0/3 sets
Transmembrane signaling receptor activity	3.690000E-04	0/3 sets	2/3 sets
Translation regulator activity	4.090000E-04	0/3 sets	0/3 sets
Prox. promoter seq.-spec. DNA binding	4.140000E-04	2/3 sets	0/3 sets
Gated channel activity	5.010000E-04	0/3 sets	2/3 sets
Ras GTPase binding	5.240000E-04	0/3 sets	0/3 sets
GTPase binding	5.690000E-04	0/3 sets	0/3 sets
Calcium channel activity	5.770000E-04	0/3 sets	0/3 sets
Ion channel regulator activity	6.190000E-04	0/3 sets	0/3 sets
Mitogen-activated protein kinase kinase binding	6.860000E-04	0/3 sets	0/3 sets
Microtubule binding	6.980000E-04	0/3 sets	0/3 sets
Small GTPase binding	7.510000E-04	0/3 sets	0/3 sets
Potassium channel activity	7.550000E-04	0/3 sets	0/3 sets
Extracellular matrix binding	7.790000E-04	0/3 sets	0/3 sets
Cytokine binding	7.790000E-04	0/3 sets	1/3 sets
Collagen binding	7.790000E-04	0/3 sets	0/3 sets
3',5'-cyclic-GMP phosphodiesterase activity	7.790000E-04	0/3 sets	0/3 sets
Calcium ion transmembrane transporter activity	8.790000E-04	0/3 sets	0/3 sets

* Schwartz *et al.* (2010) HC Class I/II, Enderle *et al.* 2011
* Published predictions: PREdictor (2003), EpiPredictor Basic(2012), EpiPredictor CG (2012)

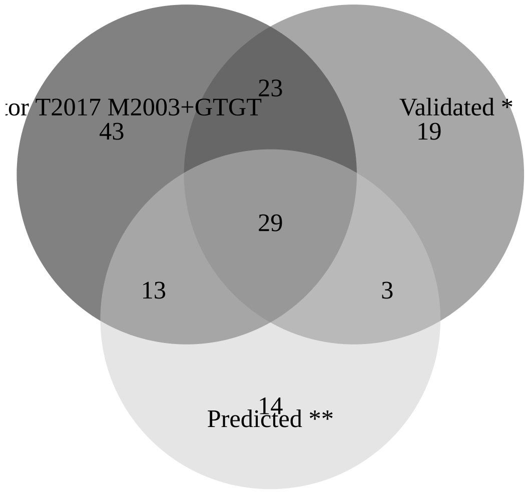

Gene ontology - Article - SVM-MOCCA (term in ? experimental/prediction sets) - Top 20

Set name:	SVMMOCCA_T2017SchwartzMC_M2003_GTGT
Significantly enriched terms:	81

Function (top 20)	p-value	Validated *	Predicted **
DNA-binding T.F. activity	3.880000E-23	3/3 sets	3/3 sets
DNA-binding T.F. activity, RNA pol. II-specific	1.990000E-20	3/3 sets	3/3 sets
Seq.-spec. DNA binding	3.270000E-20	3/3 sets	3/3 sets
Protein binding	1.450000E-19	1/3 sets	3/3 sets
Transcription regulator activity	7.210000E-18	3/3 sets	3/3 sets
T.F. activity, RNA pol. II distal enhancer seq.-spec. binding	1.210000E-13	3/3 sets	3/3 sets
Binding	1.360000E-13	2/3 sets	3/3 sets
RNA pol. II reg. region seq.-spec. DNA binding	8.130000E-12	3/3 sets	3/3 sets
RNA pol. II reg. region DNA binding	9.340000E-12	3/3 sets	3/3 sets
Calcium ion binding	1.840000E-11	0/3 sets	0/3 sets
T.F. activity, RNA pol. II prox. promoter seq.-spec. DNA binding	1.520000E-10	3/3 sets	3/3 sets
Potassium ion transmembrane transporter activity	1.520000E-10	0/3 sets	0/3 sets
Reg. region nucleic acid binding	3.920000E-10	3/3 sets	3/3 sets
Transcription reg. region DNA binding	3.920000E-10	3/3 sets	3/3 sets
Cytoskeletal protein binding	1.050000E-09	1/3 sets	0/3 sets
Signaling receptor activity	2.370000E-09	0/3 sets	2/3 sets
Actin binding	3.710000E-09	1/3 sets	0/3 sets
Cell adhesion molecule binding	3.980000E-09	0/3 sets	0/3 sets
Protein kinase activity	4.190000E-09	0/3 sets	0/3 sets
DNA-binding transcription activator activity, RNA pol. II-specific	4.650000E-09	3/3 sets	3/3 sets

* Schwartz *et al.* (2010) HC Class I/II, Enderle *et al.* 2011
* Published predictions: PREdictor (2003), EpiPredictor Basic(2012), EpiPredictor CG (2012)

Gene ontology - Article - SVM-MOCCA (term in ? experimental/prediction sets) - All

Set name:	SVMMOCCA_T2017SchwartzMC_M2003_GTGT
Significantly enriched terms:	81

Function (all)	p-value	Validated *	Predicted **
DNA-binding T.F. activity	3.880000E-23	3/3 sets	3/3 sets
DNA-binding T.F. activity, RNA pol. II-specific	1.990000E-20	3/3 sets	3/3 sets
Seq.-spec. DNA binding	3.270000E-20	3/3 sets	3/3 sets
Protein binding	1.450000E-19	1/3 sets	3/3 sets
Transcription regulator activity	7.210000E-18	3/3 sets	3/3 sets
T.F. activity, RNA pol. II distal enhancer seq.-spec. binding	1.210000E-13	3/3 sets	3/3 sets
Binding	1.360000E-13	2/3 sets	3/3 sets
RNA pol. II reg. region seq.-spec. DNA binding	8.130000E-12	3/3 sets	3/3 sets
RNA pol. II reg. region DNA binding	9.340000E-12	3/3 sets	3/3 sets
Calcium ion binding	1.840000E-11	0/3 sets	0/3 sets
T.F. activity, RNA pol. II prox. promoter seq.-spec. DNA binding	1.520000E-10	3/3 sets	3/3 sets
Potassium ion transmembrane transporter activity	1.520000E-10	0/3 sets	0/3 sets
Reg. region nucleic acid binding	3.920000E-10	3/3 sets	3/3 sets
Transcription reg. region DNA binding	3.920000E-10	3/3 sets	3/3 sets
Cytoskeletal protein binding	1.050000E-09	1/3 sets	0/3 sets
Signaling receptor activity	2.370000E-09	0/3 sets	2/3 sets
Actin binding	3.710000E-09	1/3 sets	0/3 sets
Cell adhesion molecule binding	3.980000E-09	0/3 sets	0/3 sets
Protein kinase activity	4.190000E-09	0/3 sets	0/3 sets
DNA-binding transcription activator activity, RNA pol. II-specific	4.650000E-09	3/3 sets	3/3 sets
Transcription reg. region seq.-spec. DNA binding	4.940000E-09	3/3 sets	3/3 sets
Kinase activity	8.630000E-09	1/3 sets	0/3 sets
Molecular transducer activity	1.410000E-08	0/3 sets	2/3 sets
Metal ion transmembrane transporter activity	1.980000E-08	0/3 sets	0/3 sets
Enhancer binding	4.580000E-08	2/3 sets	3/3 sets
Seq.-spec. double-stranded DNA binding	4.600000E-08	3/3 sets	3/3 sets
Enhancer seq.-spec. DNA binding	6.730000E-08	2/3 sets	3/3 sets
DNA binding	1.290000E-07	3/3 sets	3/3 sets
Voltage-gated cation channel activity	1.380000E-07	0/3 sets	0/3 sets
Protein serine/threonine kinase activity	1.990000E-07	0/3 sets	0/3 sets
Cation channel activity	2.750000E-07	0/3 sets	2/3 sets
Signaling receptor binding	3.810000E-07	1/3 sets	1/3 sets
Phosphotransferase activity, alcohol group as acceptor	4.200000E-07	0/3 sets	0/3 sets
Double-stranded DNA binding	5.180000E-07	3/3 sets	3/3 sets
Cation transmembrane transporter activity	6.630000E-07	0/3 sets	1/3 sets
Ion transmembrane transporter activity	8.350000E-07	0/3 sets	0/3 sets
Voltage-gated ion channel activity	1.010000E-06	0/3 sets	0/3 sets
Voltage-gated channel activity	1.010000E-06	0/3 sets	0/3 sets
T.F. binding	1.390000E-06	2/3 sets	3/3 sets
G-protein coupled receptor activity	1.650000E-06	0/3 sets	1/3 sets
Calcium ion transmembrane transporter activity	1.850000E-06	0/3 sets	0/3 sets
Prox. promoter DNA-binding transcription activator activity, RNA pol. II-specific	2.050000E-06	2/3 sets	3/3 sets
Transmembrane signaling receptor activity	2.160000E-06	0/3 sets	2/3 sets
Transferase activity, transferring phosphorus-containing groups	2.710000E-06	0/3 sets	0/3 sets
Potassium channel activity	3.380000E-06	0/3 sets	0/3 sets
Ion channel activity	3.680000E-06	0/3 sets	2/3 sets
G-protein coupled amine receptor activity	4.040000E-06	0/3 sets	1/3 sets
DNA-binding transcription repressor activity, RNA pol. II-specific	4.270000E-06	2/3 sets	0/3 sets
Passive transmembrane transporter activity	4.950000E-06	0/3 sets	1/3 sets
Channel activity	4.950000E-06	0/3 sets	1/3 sets
Inorganic cation transmembrane transporter activity	5.270000E-06	0/3 sets	1/3 sets

Substrate-specific channel activity	5.710000E-06	0/3 sets	2/3 sets
RNA pol. II distal enhancer seq.-spec. DNA binding	6.540000E-06	2/3 sets	3/3 sets
Gated channel activity	7.610000E-06	0/3 sets	2/3 sets
Prox. promoter DNA-binding transcription repressor activity, RNA pol. II-specific	7.890000E-06	2/3 sets	0/3 sets
Calcium channel activity	1.010000E-05	0/3 sets	0/3 sets
Inorganic molecular entity transmembrane transporter activity	1.030000E-05	0/3 sets	0/3 sets
Voltage-gated potassium channel activity	1.170000E-05	0/3 sets	0/3 sets
Steroid hormone receptor activity	1.320000E-05	1/3 sets	0/3 sets
T.F. activity, direct ligand regulated seq.-spec. DNA binding	1.320000E-05	1/3 sets	0/3 sets
Nuclear receptor activity	1.320000E-05	1/3 sets	0/3 sets
Rho guanyl-nucleotide exchange factor activity	3.630000E-05	0/3 sets	0/3 sets
Ion gated channel activity	3.790000E-05	0/3 sets	2/3 sets
Ion binding	1.060000E-04	1/3 sets	0/3 sets
Protein homodimerization activity	1.290000E-04	2/3 sets	1/3 sets
Identical protein binding	1.320000E-04	1/3 sets	1/3 sets
Microtubule binding	1.400000E-04	0/3 sets	0/3 sets
Ras guanyl-nucleotide exchange factor activity	1.470000E-04	0/3 sets	0/3 sets
Actin filament binding	1.610000E-04	0/3 sets	0/3 sets
Ligand-gated cation channel activity	2.150000E-04	0/3 sets	1/3 sets
Neurotransmitter receptor activity	3.130000E-04	0/3 sets	1/3 sets
Cation binding	3.650000E-04	0/3 sets	0/3 sets
Cadherin binding	4.110000E-04	0/3 sets	0/3 sets
Metal ion binding	4.460000E-04	0/3 sets	0/3 sets
MRNA 3'-UTR binding	4.730000E-04	1/3 sets	1/3 sets
Rho GTPase binding	4.980000E-04	0/3 sets	0/3 sets
Guanyl-nucleotide exchange factor activity	6.740000E-04	0/3 sets	0/3 sets
Tubulin binding	6.980000E-04	0/3 sets	0/3 sets
Repressing T.F. binding	7.430000E-04	2/3 sets	0/3 sets
Transmembrane transporter activity	8.010000E-04	0/3 sets	0/3 sets
Neuropeptide receptor activity	8.740000E-04	0/3 sets	0/3 sets

* Schwartz *et al.* (2010) HC Class I/II, Enderle *et al.* 2011

* Published predictions: PREdictor (2003), EpiPredictor Basic(2012), EpiPredictor CG (2012)

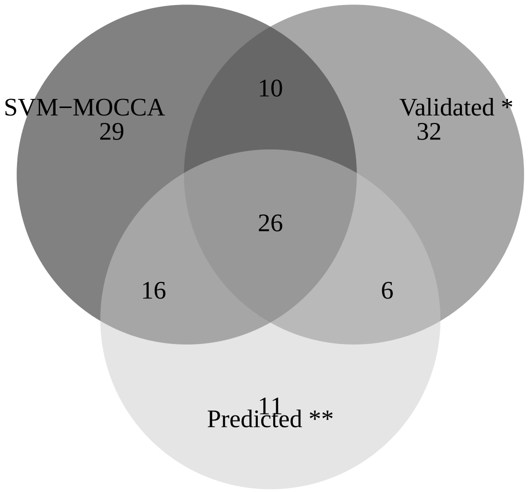

Supplement: gkz617_Supplemental_Files [file gkz617_supplemental_files.zip › SFile9_GeneOntologyAnalysis.pdf]
