# Supplementary material for: DNA sequence models of genome-wide Drosophila melanogaster Polycomb binding sites improve generalization to independent Polycomb Response Elements
Source: Nucleic Acids Res. 2019 Jul 24;47(15):7781–97. doi: 10.1093/nar/gkz617 (PMC6735708; doi:10.1093/nar/gkz617)
Supplement: gkz617_Supplemental_Files [file gkz617_supplemental_files.zip › SupplementaryMaterial_revision_2.pdf]

DNA sequence models of genome-wide *D. melanogaster* Polycomb binding sites improve generalization to independent Polycomb Response Elements

Supplementary Material

Bjørn André Bredesen and Marc Rehmsmeier, July 1, 2019

Supplementary Figure 1: Model distinction of PREs from coding sequence fragments

Classifiers used: CPREdictor and SVM-MOCCA
Motifs used: same as by Ringrose *et al.* (2003) + GTGT
PRE training set: Schwartz *et al.* (2010)—cross-validation training portion (110 sequences).
PRE test set: Schwartz *et al.* (2010)—cross-validation test portion, independent from training set (50 sequences).
Non-PRE test set: 5000 coding sequence fragments—CDS fragments used for training SVM-MOCCA in each cross-validation fold are excluded.

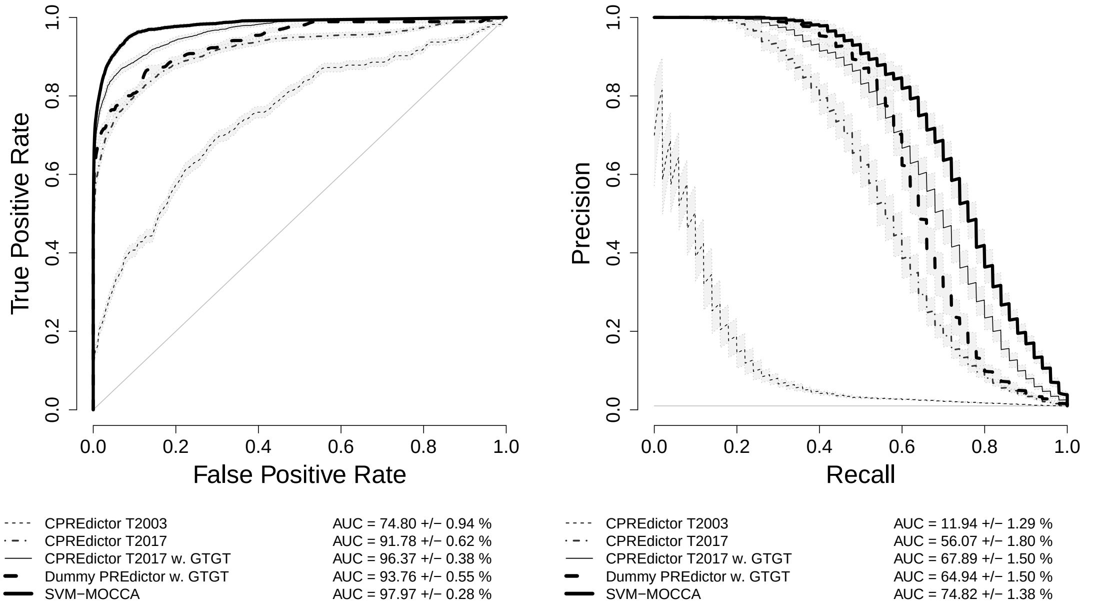

Supplementary Figure 2: Cross-validation generalization when training with different PRE sets

Classifiers used: CPREdictor and SVM-MOCCA
Motifs used: same as by Ringrose *et al.* (2003) (+GTGT for SVM-MOCCA and for CPREdictor T2017 w. GTGT)
PRE training set: As specified—cross-validation training portion (110 sequences).
PRE test set: As specified—cross-validation test portion (50 sequences), independent from training set.
Non-PRE test set: 5000 sequences randomly generated by a 4th-order Markov chain trained on specified PRE set.

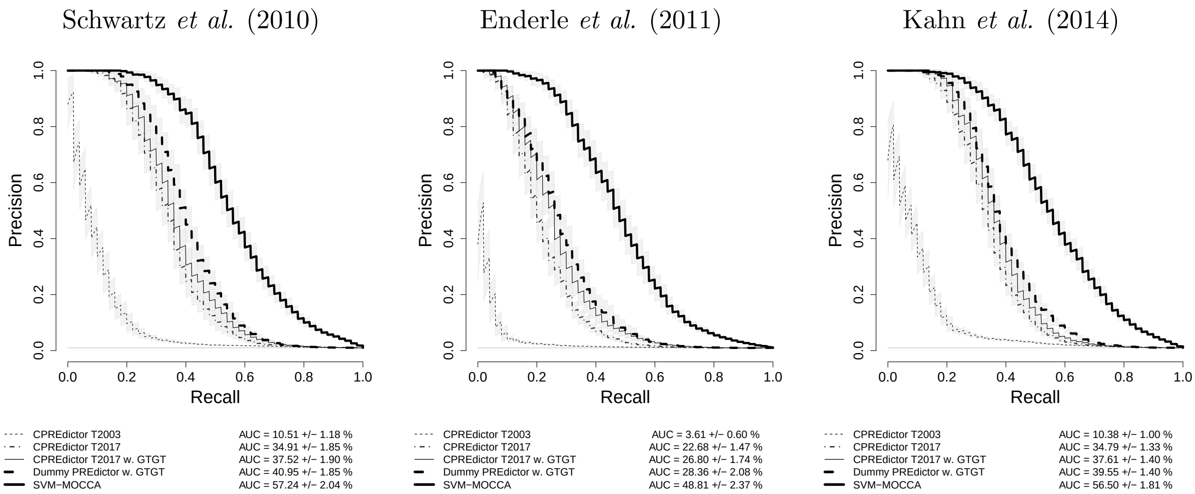

Supplementary Figure 3: Generalization when training models using Ringrose *et al.* (2003) PREs and Markov chain controls

PRE training set: Ringrose *et al.* (2003) (12 sequences).
Non-PRE training set: When "M.C." is included in the name, non-PRE training sequences were generated by a 4th-order Markov chain trained on the T2003 PREs (12 sequences, with lengths equal to the mean T2003 PRE length). Otherwise, the T2003 non-PREs were used.
PRE test set: Schwartz *et al.* (2010)—cross-validation test portion (50 sequences).
Non-PRE test set, left plot: 5000 sequences randomly generated by a 4th-order Markov chain trained genome-wide.
Non-PRE test set, right plot: 5000 sequences randomly generated by a 4th-order Markov chain trained on PREs determined by Schwartz *et al.* (2010).

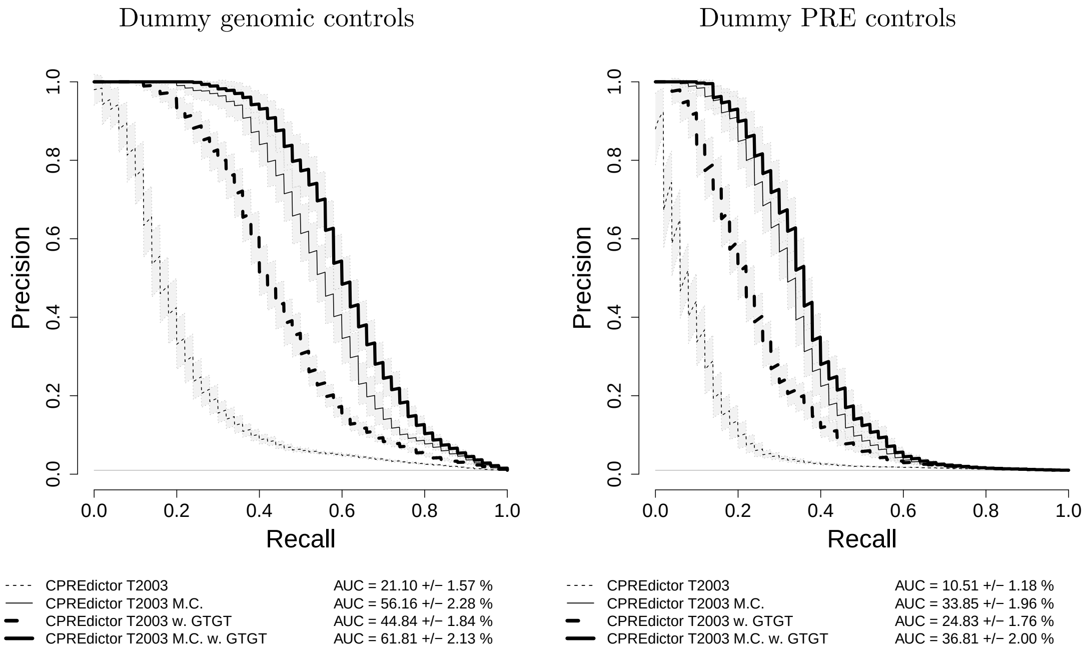

Supplementary Figure 4: Generalization with GTGT versus random 4-mers

Classifiers used: CPREdictor

PRE training set: Schwartz *et al.* (2010)—cross-validation training portion (110 sequences).

PRE test set: Schwartz *et al.* (2010)—cross-validation test portion (50 sequences), independent from training set.

Motifs used: same as by Ringrose *et al.* (2003) + the specified 4-mers.

Non-PRE test set, left plot: 5000 sequences randomly generated by a 4th-order Markov chain trained genome-wide.

Non-PRE test set, right plot: 5000 sequences randomly generated by a 4th-order Markov chain trained on PREs determined by Schwartz *et al.* (2010).

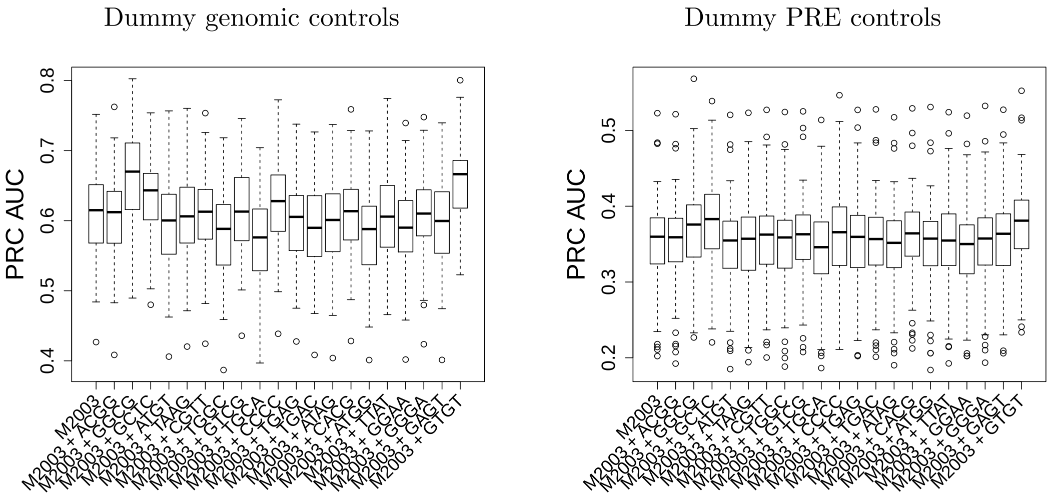

Supplementary Figure 5: Generalization for the addition of published PRE motifs

Classifier used: CPREdictor
Motifs used: same as by Ringrose *et al.* (2003) + motifs as shown
PRE training set: Schwartz *et al.* (2010)—cross-validation training portion (110 sequences).
PRE test set: Schwartz *et al.* (2010)—cross-validation test portion (50 sequences), independent from training set.
Non-PRE test set, left plot: 5000 sequences randomly generated by a 4th-order Markov chain trained genome-wide.
Non-PRE test set, right plot: 5000 sequences randomly generated by a 4th-order Markov chain trained on PREs determined by Schwartz *et al.* (2010).

Extra motifs:

Listed name	Factor name	Motif
Zeste2	Zeste	BGAGTGV
Sp1Klf	Sp1/KLF	RRGGYG
Dsp1	Dsp1	GAAAA
Grh1	Grainyhead	TGTTTTTT
Grh2	Grainyhead	WCHGGTT
SiteA	Site A	GAACNG

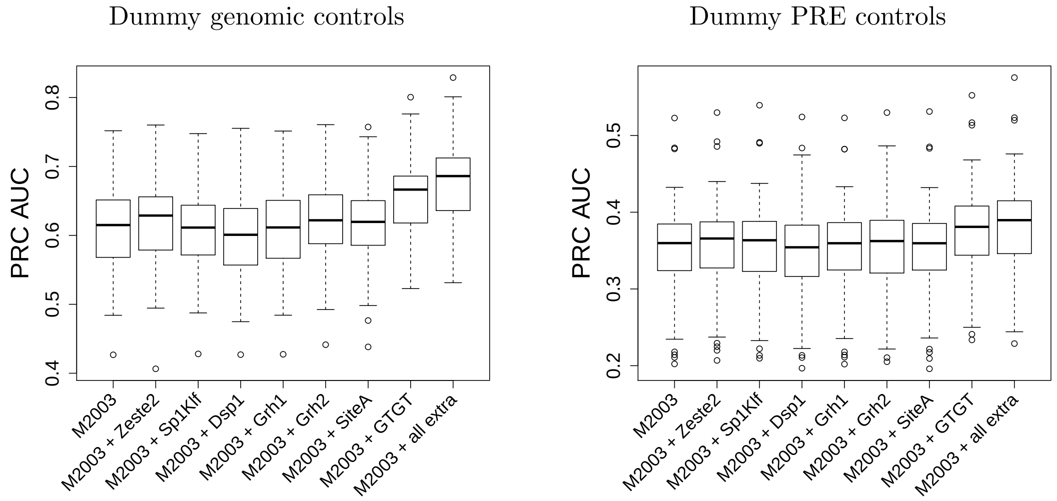

Supplementary Figure 6: CPREdictor motif pair weights

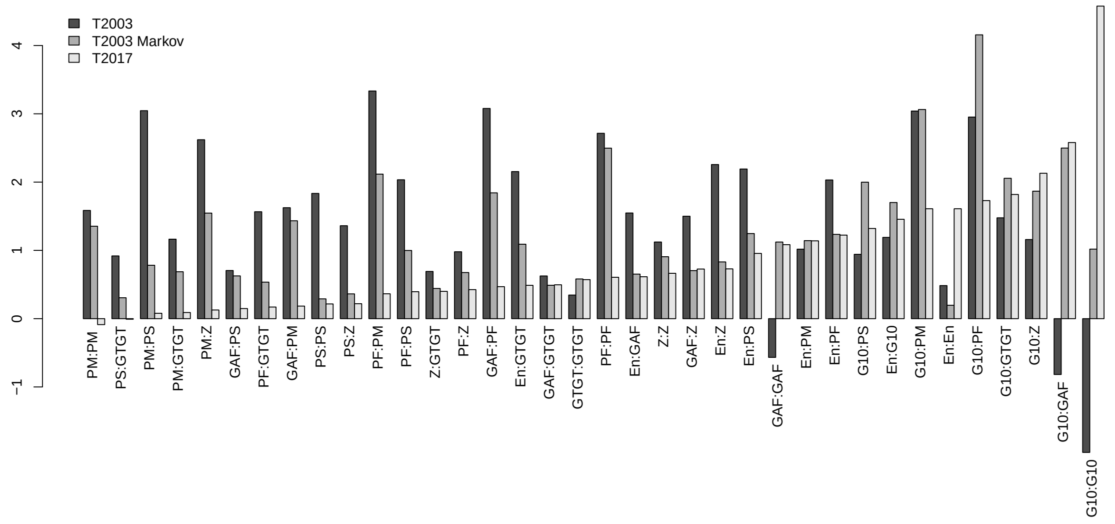

Models from cross-validation fold 1 are considered. T2003 and T2017 are as specified in the article, with T2017 using PcG targets from the Schwartz *et al.* (2010) set. T2003Markov uses PREs from T2003 and non-PREs generated by a 4th-order Markov chain trained on the T2003 PREs.

A	B	Pearson's correlation coefficient
T2003	T2017	-0.5102701
T2003	T2003Markov	0.3524476
T2003Markov	T2017	0.3769198

Supplementary Figure 7: Generalization performance of models trained on ChIP PcG target sites to Ringrose *et al.* (2003) PREs

Training set: Schwartz *et al.* (2010) PREs versus dummy PREs (generated by 4th-order Markov chain)
PRE training set: Schwartz *et al.* (2010)—cross-validation training portion (110 sequences).
PRE test set: As used for training by Ringrose *et al.* (2003) (12 sequences).
Non-PRE test set: As used for training by Ringrose *et al.* (2003) (16 sequences).

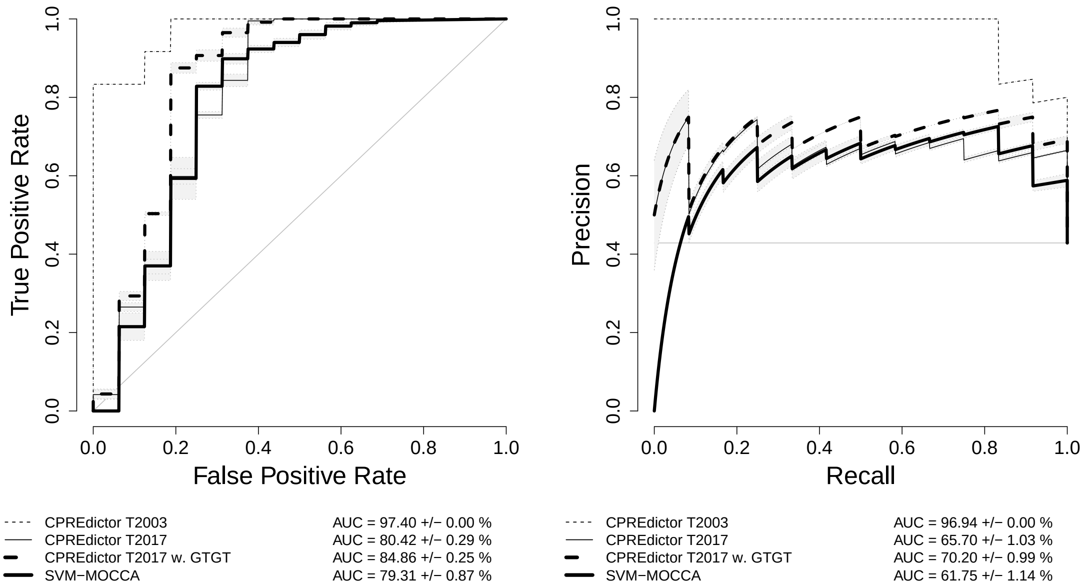

PRE test set: As used for training by Ringrose *et al.* (2003) (12 sequences).
Non-PRE test set: 16 randomly generated by a 4th-order Markov chain trained on the Ringrose *et al.* (2003) training set PREs.

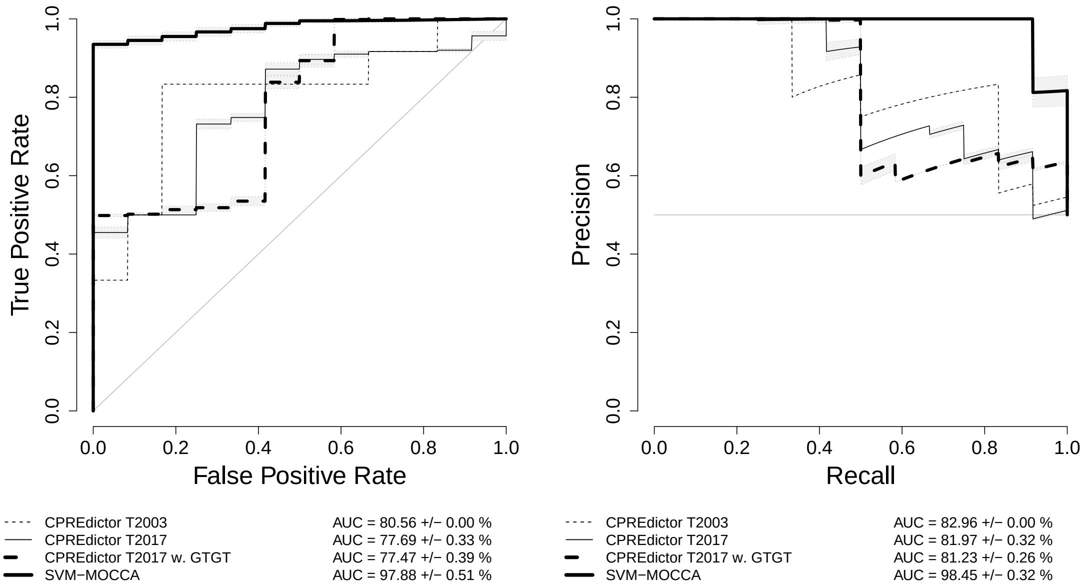

Supplementary Figure 8: Window size influence

Classifiers used: CPREdictor and SVM-MOCCA (linear kernel)
Motifs used: same as by Ringrose *et al.* (2003) + GTGT
PRE training set: Schwartz *et al.* (2010)—cross-validation training portion (110 sequences).
PRE test set: Schwartz *et al.* (2010)—cross-validation test portion (50 sequences), independent from training set.
Non-PRE test set, left plot: 5000 sequences randomly generated by a 4th-order Markov chain trained genome-wide.
Non-PRE test set, right plot: 5000 sequences randomly generated by a 4th-order Markov chain trained on PREs determined by Schwartz *et al.* (2010).

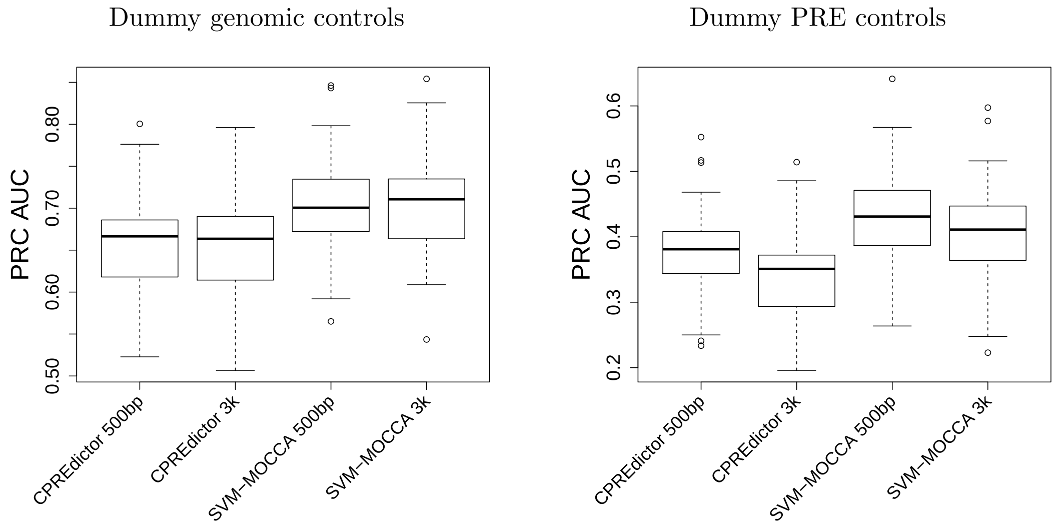

Supplementary Figure 9: SVM-MOCCA kernel function generalization performance

Classifiers used: SVM-MOCCA, 3kb window
Motifs used: same as by Ringrose *et al.* (2003) + GTGT
PRE training set: Schwartz *et al.* (2010)—cross-validation training portion (110 sequences).
PRE test set: Schwartz *et al.* (2010)—cross-validation test portion (50 sequences), independent from training set.
Non-PRE test set, left plot: 5000 sequences randomly generated by a 4th-order Markov chain trained genome-wide.
Non-PRE test set, right plot: 5000 sequences randomly generated by a 4th-order Markov chain trained on PREs determined by Schwartz *et al.* (2010).

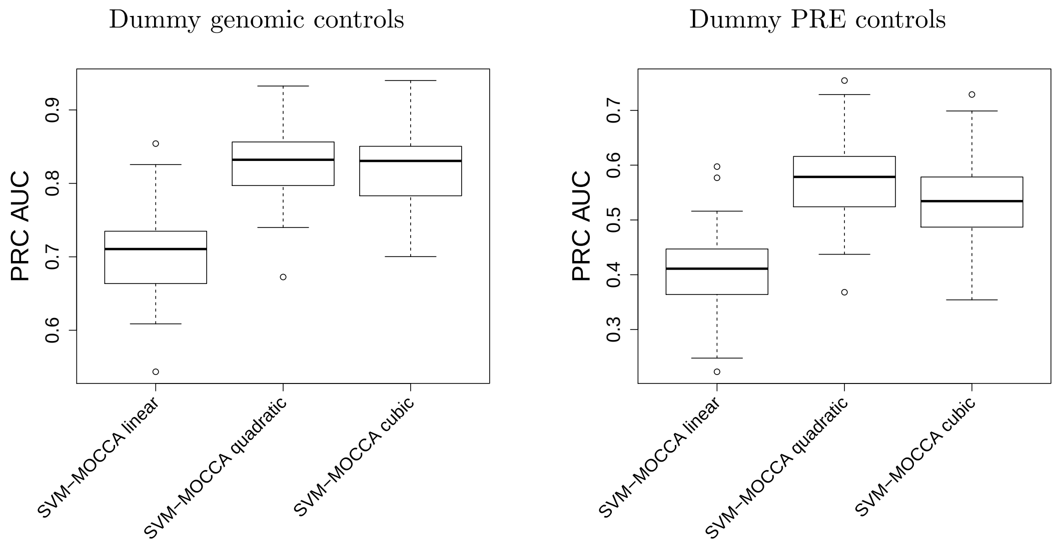

Supplementary Figure 10: Extended prediction overlap evaluation

Classifiers used: CPREdictor and SVM-MOCCA
Motifs used: same as by Ringrose *et al.* (2003) + GTGT
PRE training set: Schwartz *et al.* (2010)—cross-validation training portion (110 sequences).

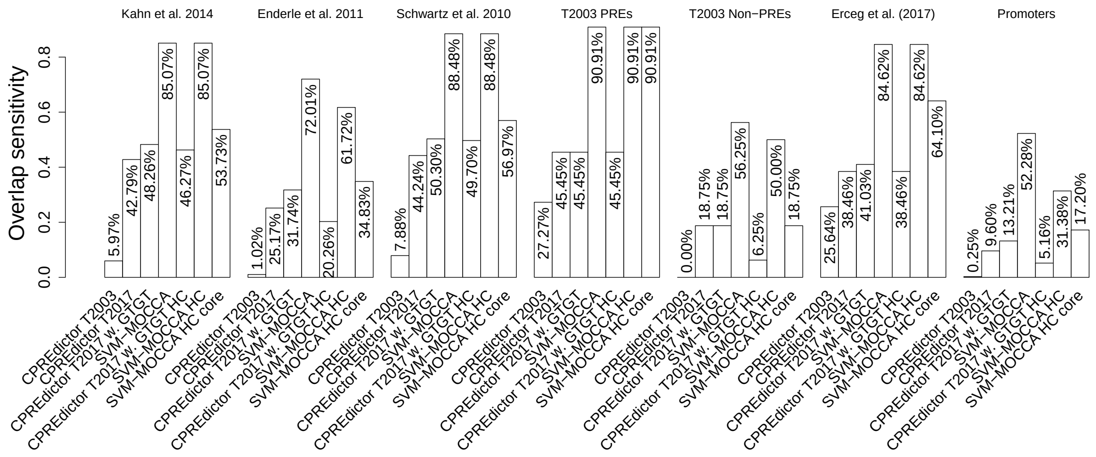

Supplementary Figure 11: Gene ontology analysis of PcG/TrxG target gene predictions made by SVM-MOCCA

Gene ontology analysis of PcG/TrxG target gene predictions made by SVM-MOCCA is highly enriched for regulatory functions. For the analysis, we used GOrilla, with target gene lists versus the rest of the annotated genes in FlyBase (Gramates *et al.* (2017)) Drosophila genome Release 6.04 as two unranked lists. The top 20 functional gene ontology terms are listed. We performed a similar analysis on experimentally determined PREs * (Schwartz *et al.* (2010) HC Class I/II, Enderle *et al.* (2011)) and published PRE prediction sets ** (PREdictor, Ringrose *et al.* (2003), EpiPredictor Basic and EpiPredictor CG, Zeng *et al.* (2012)). The Venn diagram shows how many terms are shared between SVM-MOCCA and any of the predicted and validated sets of genes.

Function (top 20)	p-value	Validated *	Predicted **
DNA-binding T.F. activity	3.880000E-23	3/3 sets	3/3 sets
DNA-binding T.F. activity, RNA pol. II-specific	1.990000E-20	3/3 sets	3/3 sets
Seq.-spec. DNA binding	3.270000E-20	3/3 sets	3/3 sets
Protein binding	1.450000E-19	1/3 sets	3/3 sets
Transcription regulator activity	7.210000E-18	3/3 sets	3/3 sets
T.F. activity, RNA pol. II distal enhancer seq.-spec. binding	1.210000E-13	3/3 sets	3/3 sets
Binding	1.360000E-13	2/3 sets	3/3 sets
RNA pol. II reg. region seq.-spec. DNA binding	8.130000E-12	3/3 sets	3/3 sets
RNA pol. II reg. region DNA binding	9.340000E-12	3/3 sets	3/3 sets
Calcium ion binding	1.840000E-11	0/3 sets	0/3 sets
T.F. activity, RNA pol. II prox. promoter seq.-spec. DNA binding	1.520000E-10	3/3 sets	3/3 sets
Potassium ion transmembrane transporter activity	1.520000E-10	0/3 sets	0/3 sets
Reg. region nucleic acid binding	3.920000E-10	3/3 sets	3/3 sets
Transcription reg. region DNA binding	3.920000E-10	3/3 sets	3/3 sets
Cytoskeletal protein binding	1.050000E-09	1/3 sets	0/3 sets
Signaling receptor activity	2.370000E-09	0/3 sets	2/3 sets
Actin binding	3.710000E-09	1/3 sets	0/3 sets
Cell adhesion molecule binding	3.980000E-09	0/3 sets	0/3 sets
Protein kinase activity	4.190000E-09	0/3 sets	0/3 sets
DNA-binding transcription activator activity, RNA pol. II-specific	4.650000E-09	3/3 sets	3/3 sets

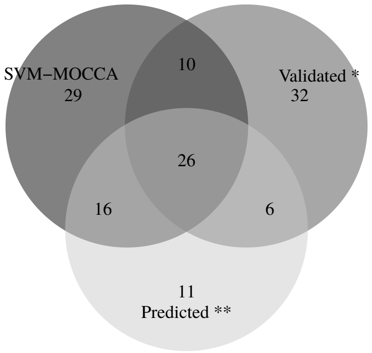

Supplementary Table 1: Full modENCODE FTP paths for the data sets considered

Base path: ftp://data.modencode.org/D.melanogaster

H3K27me3

- Histone-Modification/ChIP-chip/computed-peaks_gff3/H3K27me3:Developmental-Stage=Adult-Female: ChIP-chip:Rep-1::Dmel_r5.32:modENCODE_346.gff3.gz
- Histone-Modification/ChIP-chip/computed-peaks_gff3/H3K27me3:Developmental-Stage=Embryos-12-16-hr:ChIP-chip:Rep-1::Dmel_r5.32:modENCODE_767.gff3.gz
- Histone-Modification/ChIP-chip/computed-peaks_gff3/H3K27me3:Cell-Line=S2-DRSC# Developmental-Stage=Late-Embryonic-stage#Tissue=Embryo-derived-cell-line:ChIP-chip:Rep-1::Dmel_r5.32: modENCODE_298.gff3.gz
- Histone-Modification/ChIP-chip/computed-peaks_gff3/H3K27me3:Developmental-Stage=Larvae-L2-stage: ChIP-chip:Rep-1::Dmel_r5.32:modENCODE_371.gff3.gz
- Histone-Modification/ChIP-chip/computed-peaks_gff3/H3K27me3:Developmental-Stage=Embryos-20-24-hr:ChIP-chip:Rep-1::Dmel_r5.32:modENCODE_370.gff3.gz
- Histone-Modification/ChIP-chip/computed-peaks_gff3/H3K27me3:Developmental-Stage=Embryos-4-8-hr: ChIP-chip:Rep-1::Dmel_r5.32:modENCODE_768.gff3.gz
- Histone-Modification/ChIP-chip/computed-peaks_gff3/H3K27me3:Developmental-Stage=Embryos-0-12-hr: ChIP-chip:Rep-1::Dmel_r5.32:modENCODE_919.gff3.gz
- Histone-Modification/ChIP-chip/computed-peaks_gff3/H3K27me3:Developmental-Stage=Larvae-L1-stage: ChIP-chip:Rep-1::Dmel_r5.32:modENCODE_373.gff3.gz
- Histone-Modification/ChIP-chip/computed-peaks_gff3/H3K27me3:Developmental-Stage=Adult-Male: ChIP-chip:Rep-1::Dmel_r5.32:modENCODE_869.gff3.gz
- Histone-Modification/ChIP-chip/computed-peaks_gff3/H3K27me3:Developmental-Stage=Embryos-8-12-hr: ChIP-chip:Rep-1::Dmel_r5.32:modENCODE_868.gff3.gz
- Histone-Modification/ChIP-chip/computed-peaks_gff3/H3K27me3:Developmental-Stage=Embryos-0-4-hr# Strain=Y-cn-bw-sp:ChIP-chip:Rep-1::Dmel_r5.32:modENCODE_867.gff3.gz
- Histone-Modification/ChIP-chip/computed-peaks_gff3/H3K27me3:Developmental-Stage=Embryos-16-20-hr:ChIP-chip:Rep-1::Dmel_r5.32:modENCODE_376.gff3.gz
- Non-TF-Chromatin-binding-factor/ChIP-chip/computed-peaks_gff3/H3K27me3:Cell-Line=ML-DmBG3-c2# Developmental-Stage=Larvae-3rd-instar#RNAi-reagent=CG10159-RNAi#Tissue=CNS-derived-cell-line: ChIP-chip:Rep-1::Dmel_r5.32:modENCODE_3679.gff3.gz

H3K4me1

- Histone-Modification/ChIP-chip/computed-peaks_gff3/H3K4me1:Developmental-Stage=Adult-Female: ChIP-chip:Rep-1::Dmel_r5.32:modENCODE_432.gff3.gz
- Histone-Modification/ChIP-chip/computed-peaks_gff3/H3K4me1:Developmental-Stage=Embryos-12-16-hr: ChIP-chip:Rep-1::Dmel_r5.32:modENCODE_418.gff3.gz
- Histone-Modification/ChIP-chip/computed-peaks_gff3/H3K4me1:Developmental-Stage=Embryos-8-12-hr: ChIP-chip:Rep-1::Dmel_r5.32:modENCODE_864.gff3.gz
- Histone-Modification/ChIP-chip/computed-peaks_gff3/H3K4me1:Developmental-Stage=Adult-Male: ChIP-chip:Rep-1::Dmel_r5.32:modENCODE_428.gff3.gz
- Histone-Modification/ChIP-chip/computed-peaks_gff3/H3K4me1:Developmental-Stage=Embryos-4-8-hr: ChIP-chip:Rep-1::Dmel_r5.32:modENCODE_403.gff3.gz
- Histone-Modification/ChIP-chip/computed-peaks_gff3/H3K4me1:Developmental-Stage=Embryos-0-4-hr: ChIP-chip:Rep-1::Dmel_r5.32:modENCODE_423.gff3.gz
- Histone-Modification/ChIP-chip/computed-peaks_gff3/H3K4me1:Developmental-Stage=Embryos-20-24-hr: ChIP-chip:Rep-1::Dmel_r5.32:modENCODE_408.gff3.gz

- Histone-Modification/ChIP-seq/computed-peaks_gff3/H3K4me1:Developmental-Stage=Embryos-20-24-hr#Strain=Y-cn-bw-sp#threshold=37.3:ChIP-seq:Rep-1::Dmel_r5.32:modENCODE_782.gff3.gz
- Histone-Modification/ChIP-chip/computed-peaks_gff3/H3K4me1:Developmental-Stage=Embryos-16-20-hr:ChIP-chip:Rep-1::Dmel_r5.32:modENCODE_413.gff3.gz
- Histone-Modification/ChIP-seq/computed-peaks_gff3/H3K4me1:Developmental-Stage=Larvae-L3-stage:ChIP-seq:Rep-1::Dmel_r5.32:modENCODE_4986.gff3.gz

H3K4me3

- Histone-Modification/ChIP-chip/computed-peaks_gff3/H3K4me3:Developmental-Stage=Larvae-L2-stage:ChIP-chip:Rep-1::Dmel_r5.32:modENCODE_392.gff3.gz
- Histone-Modification/ChIP-chip/computed-peaks_gff3/H3K4me3:Developmental-Stage=Larvae-L1-stage:ChIP-chip:Rep-1::Dmel_r5.32:modENCODE_397.gff3.gz
- Histone-Modification/ChIP-chip/computed-peaks_gff3/H3K4me3:Developmental-Stage=Embryos-20-24-hr:ChIP-chip:Rep-1::Dmel_r5.32:modENCODE_407.gff3.gz
- Histone-Modification/ChIP-chip/computed-peaks_gff3/H3K4me3:Developmental-Stage=Embryos-16-20-hr:ChIP-chip:Rep-1::Dmel_r5.32:modENCODE_412.gff3.gz
- Histone-Modification/ChIP-chip/computed-peaks_gff3/H3K4me3:Developmental-Stage=Embryos-8-12-hr:ChIP-chip:Rep-1::Dmel_r5.32:modENCODE_865.gff3.gz
- Histone-Modification/ChIP-chip/computed-peaks_gff3/H3K4me3:Developmental-Stage=Embryos-4-8-hr:ChIP-chip:Rep-1::Dmel_r5.32:modENCODE_402.gff3.gz
- Histone-Modification/ChIP-chip/computed-peaks_gff3/H3K4me3:Developmental-Stage=Embryos-12-16-hr:ChIP-chip:Rep-1::Dmel_r5.32:modENCODE_417.gff3.gz
- Histone-Modification/ChIP-chip/computed-peaks_gff3/H3K4me3_Cell-Line=Kc167#Developmental-Stage=Late-Embryonic-stage#Tissue=Embryo-derived-cell-line:ChIP-chip:Rep-1::Dmel_r5.32:modENCODE_912.gff3.gz
- Histone-Modification/ChIP-chip/computed-peaks_gff3/H3K4me3_Cell-Line=Kc167#Developmental-Stage=Late-Embryonic-stage#Tissue=Embryo-derived-cell-line:ChIP-chip:Rep-1::Dmel_r5.32:modENCODE_5141.gff3.gz
- Histone-Modification/ChIP-chip/computed-peaks_gff3/H3K4me3:Developmental-Stage=Adult-Female:ChIP-chip:Rep-1::Dmel_r5.32:modENCODE_431.gff3.gz
- Histone-Modification/ChIP-chip/computed-peaks_gff3/H3K4me3:Developmental-Stage=Adult-Male:ChIP-chip:Rep-1::Dmel_r5.32:modENCODE_427.gff3.gz
- Histone-Modification/ChIP-chip/computed-peaks_gff3/H3K4me3:Developmental-Stage=Embryos-0-4-hr:ChIP-chip:Rep-1::Dmel_r5.32:modENCODE_771.gff3.gz
- Histone-Modification/ChIP-chip/computed-peaks_gff3/H3K4me3:Developmental-Stage=Embryos-0-12-hr#Tissue=Embryo:ChIP-chip:Rep-1::Dmel_r5.32:modENCODE_622.gff3.gz
- Non-TF-Chromatin-binding-factor/ChIP-chip/computed-peaks_gff3/H3K4me3_Cell-Line=ML-DmBG3-c2#Developmental-Stage=Larvae-3rd-instar#RNAi-reagent=CG8573-RNAi#Tissue=CNS-derived-cell-line:ChIP-chip:Rep-1::Dmel_r5.32:modENCODE_3691.gff3.gz

Pc

- Non-TF-Chromatin-binding-factor/ChIP-chip/computed-peaks_gff3/Pc:Developmental-Stage=Embryos-2-4-hr-OR#Strain=Oregon-R:ChIP-chip:Rep-1::Dmel_r5.32:modENCODE_5064.gff3.gz
- Non-TF-Chromatin-binding-factor/ChIP-chip/computed-peaks_gff3/Pc:Cell-Line=S2-DRSC#Developmental-Stage=Late-Embryonic-stage#Tissue=Embryo-derived-cell-line:ChIP-chip:Rep-1::Dmel_r5.32:modENCODE_326.gff3.gz
- Non-TF-Chromatin-binding-factor/ChIP-chip/computed-peaks_gff3/Pc:Cell-Line=S2-DRSC#Developmental-Stage=Late-Embryonic-stage#Tissue=Embryo-derived-cell-line:ChIP-chip:Rep-1::Dmel_r5.32:modENCODE_3791.gff3.gz

- Non-TF-Chromatin-binding-factor/ChIP-chip/computed-peaks_gff3/Pc:Cell-Line=ML-DmBG3-c2#Developmental-Stage=Larvae-3rd-instar#Tissue=CNS-derived-cell-line:ChIP-chip:Rep-1::Dmel_r5.32:modENCODE_325.gff3.gz
- Non-TF-Chromatin-binding-factor/ChIP-seq/computed-peaks_gff3/Pc:Developmental-Stage=Embryos-14-16-hr-OR#Strain=Oregon-R:ChIP-seq:Rep-1::Dmel_r5.32:modENCODE_3957:816.gff3.gz
- Non-TF-Chromatin-binding-factor/ChIP-seq/computed-peaks_gff3/Pc:Developmental-Stage=Embryos-14-16-hr-OR#Strain=Oregon-R:ChIP-seq:Rep-1::Dmel_r5.32:modENCODE_3957:948.gff3.gz

Psc

- Non-TF-Chromatin-binding-factor/ChIP-chip/computed-peaks_gff3/Psc:Cell-Line=ML-DmBG3-c2#Developmental-Stage=Larvae-3rd-instar#Tissue=CNS-derived-cell-line:ChIP-chip:Rep-1::Dmel_r5.32:modENCODE_3055.gff3.gz
- Non-TF-Chromatin-binding-factor/ChIP-chip/computed-peaks_gff3/Psc:Cell-Line=S2-DRSC#Developmental-Stage=Late-Embryonic-stage#Tissue=Embryo-derived-cell-line:ChIP-chip:Rep-1::Dmel_r5.32:modENCODE_3797.gff3.gz
- Non-TF-Chromatin-binding-factor/ChIP-chip/computed-peaks_gff3/Psc:Cell-Line=S2-DRSC#Developmental-Stage=Late-Embryonic-stage#Tissue=Embryo-derived-cell-line:ChIP-chip:Rep-1::Dmel_r5.32:modENCODE_3056.gff3.gz

dSFMBT

- Non-TF-Chromatin-binding-factor/ChIP-chip/computed-peaks_gff3/dSFMBT:Cell-Line=S2-DRSC#Developmental-Stage=Late-Embryonic-stage#Tissue=Embryo-derived-cell-line:ChIP-chip:Rep-1::Dmel_r5.32:modENCODE_3751.gff3.gz
- Non-TF-Chromatin-binding-factor/ChIP-chip/computed-peaks_gff3/dSFMBT:Cell-Line=ML-DmBG3-c2#Developmental-Stage=Larvae-3rd-instar#Tissue=CNS-derived-cell-line:ChIP-chip:Rep-1::Dmel_r5.32:modENCODE_2986.gff3.gz

Supplementary Table 2: Comparison of H3K4me1 and H3K4me3 overlaps

Number of high-confidence PRE candidates (PREs HC) that overlap with H3K4me1 and with H3K4me3 regions:

	total	H3K4me1	H3K4me3	both	none
PREs HC	2908	2412	2367	2129	258

Percent coverage of accessible DNA:

H3K4me1	60.84%
H3K4me3	56.98%

See Materials and Methods in main article for definitions of datasets.

Supplementary Text 1: SVM-MOCCA quadratic kernel, 4-class model weight analysis, unscaled

The decision function for an SVM with a polynomial kernel is given by

$$\hat{c}(f) = \sum_{v,c \in SV} c(\gamma f \circ v + c_0)^d - \rho$$

for a set SV of support vectors and their coefficients, kernel parameters c_0 and d, and bias ρ. We ignore feature scaling coefficients, as their application relates to mapping from the original feature space to the final decision value, whereas we are interested in how the SVM weights features relative to each other (post-scaling).

For the quadratic kernel function, $d = 2$. We also set $c_0 = 0$. This gives

$$\hat{c}(f) = \sum_{v,c \in SV} c\left(\gamma f \circ v\right)^2 - \rho = \sum_{v,c \in SV} \gamma^2 c \left(\sum_{i=1}^{|f|} f_i v_i\right)^2 - \rho = \sum_{v,c \in SV} \gamma^2 c \left(\sum_{i=1}^{|f|} f_i v_i\right)\left(\sum_{j=1}^{|f|} f_j v_j\right) - \rho$$

$$= \sum_{v,c \in SV} \gamma^2 c \left(\sum_{i=1}^{|f|}\sum_{j=1}^{|f|} f_i v_i f_j v_j\right) - \rho = \sum_{i=1}^{|f|}\sum_{j=1}^{|f|} f_i f_j \sum_{v,c \in SV} \gamma^2 c v_i v_j - \rho = \sum_{i=1}^{|f|}\sum_{j=1}^{|f|} f_i f_j w_{ij} - \rho,$$

where

$$w_{ij} = \sum_{v,c \in SV} \gamma^2 c v_i v_j.$$

Each w_{ij} is a weight for a pair of features i and j.

Class-merged positive-negative boundary feature weights, top 10

Motif classifier: GAF
Weight: 3.856949

Top 10 / 171 weights:

Feature	Weight
“AA” and “CC”	-0.648216
G10 and GAF	-0.513502
GAF and Z	-0.506031
“CC” and “CC”	0.501259
G10 and Z	-0.500820
GAF and GTGT	-0.452657
“AA” and “AA”	0.442378
“AG” and “GA”	0.438191
“AA” and “CA”	-0.437333
G10 and GTGT	-0.433605

Motif classifier: GTGT
Weight: 3.643022

Top 10 / 171 weights:

Feature	Weight
“AA” and “CC”	-1.654656
“AA” and “AA”	1.444052
“AA” and “CA”	-1.279718
“CC” and “CC”	1.165707
“AA” and “AC”	-0.980621
“AG” and “GA”	0.972684
“AA” and “AG”	-0.913557
“GA” and “GA”	0.865704
“AC” and “CA”	0.829551
“AA” and “GC”	-0.829348

Motif classifier: PM
Weight: 3.323236

Top 10 / 171 weights:

Feature	Weight
GAF and “CC”	-0.279807
“AG” and “GA”	0.273742
“AA” and “CC”	-0.238752
“AC” and “CA”	0.234695
G10 and “CC”	-0.218279
En and GAF	-0.217850
“AA” and “AA”	0.182362
En and G10	-0.179969
GAF and GTGT	-0.169561
En and GTGT	-0.165382

Motif classifier: PS
Weight: 3.135494

Top 10 / 171 weights:

Feature	Weight
“AA” and “CC”	-0.944007
“AA” and “AA”	0.716074
“AA” and “CA”	-0.653051
“AG” and “GA”	0.634035
“AA” and “AG”	-0.602515
“CC” and “CC”	0.565863
“AC” and “CA”	0.561941
“AG” and “AG”	0.521967
“AA” and “GC”	-0.479201
“CA” and “CA”	0.478400

Motif classifier: G10
Weight: 3.052891

Top 10 / 171 weights:

Feature	Weight
“AA” and “CC”	-0.168033
“CC” and “CC”	0.156412
Z and “AG”	-0.128443
G10 and “AG”	-0.127633
“AG” and “GA”	-0.127260
G10 and Z	-0.125859
G10 and “GA”	-0.106502
G10 and PF	-0.105953
GTGT and “AG”	-0.105943
GTGT and “GA”	-0.102145

Motif classifier: Z
Weight: 2.998110

Top 10 / 171 weights:

Feature	Weight
“AA” and “CC”	-0.569919
“AG” and “GA”	0.516107
“AA” and “AA”	0.461579
“AA” and “AG”	-0.459881
“CC” and “CC”	0.454201
GAF and Z	-0.432681
“AG” and “AG”	0.426442
G10 and Z	-0.423217
GAF and GTGT	-0.366505
G10 and GAF	-0.364575

Motif classifier: En
Weight: 0.727049

Top 10 / 171 weights:

Feature	Weight
GAF and “AG”	0.149872
G10 and “AA”	-0.140659
GAF and “AA”	-0.132831
G10 and “AG”	0.130865
Z and “AA”	-0.129052
Z and “AG”	0.109683
PM and “AA”	-0.109664
PF and “AA”	-0.108730
GTGT and “AA”	-0.108124
En and “AA”	-0.101853

Motif classifier: PF
Weight: 0.620457

Top 10 / 171 weights:

Feature	Weight
“AG” and “GA”	0.268646
“AC” and “CA”	0.255191
“AA” and “CC”	-0.243844
En and Z	-0.235083
“AA” and “AG”	-0.226225
G10 and Z	-0.214039
En and GAF	-0.198753
En and G10	-0.196129
GAF and “AA”	-0.195781
“AG” and “AG”	0.194196

Supplementary File 1: PRE predictions—CPREdictor T2017 w. GTGT

Supplementary File 2: PRE predictions—CPREdictor T2017 w. GTGT HC

Supplementary File 3: PRE predictions—SVM-MOCCA

Supplementary File 4: PRE predictions—SVM-MOCCA HC

Supplementary File 5: PRE predictions—SVM-MOCCA HC core

Supplementary File 6: PcG target gene predictions—CPREdictor T2017 w. GTGT

Supplementary File 7: PcG target gene predictions—SVM-MOCCA

Supplementary File 8: Genome-wide prediction analysis profile

Supplementary File 9: Gene ontology analysis profile

Supplementary File 10: PRE predictions—CPREdictor T2017 w. GTGT, H3K4me1-enriched

Supplementary File 11: PRE predictions—SVM-MOCCA, H3K4me1-enriched
